# Efficacy and safety of a 4-month quabodepistat, delamanid, and bedaquiline regimen for drug-susceptible pulmonary tuberculosis: a multicentre, open-label, randomised, proof-of-concept, non-inferiority, phase 2b/c trial

**DOI:** 10.1016/S1473-3099(26)00143-X

**Published:** 2026-10

**Authors:** Rodney Dawson, Andreas H Diacon, Ebrahim Variava, Tumelo Moloantoa, William Brumskine, Tlhago Ngwanto, Ntokozo Zuma-Gwala, Ayman Osman, Mohammed Rassool, Jaclyn Ann Bennet, Yongge Liu, Raymond Xu, Wei Li, Simbarashe Takuva, Jeffrey Hafkin

**Affiliations:** a Division of Pulmonology, Department of Medicine, University of Cape Town, Cape Town, South Africa; b University of Cape Town Lung Institute, Cape Town, South Africa; c TASK, Cape Town, South Africa; d Perinatal HIV Research Unit, Tshepong Hospital Complex, Klerksdorp, South Africa; e Perinatal HIV Research Unit, Soweto, South Africa; f The Aurum Institute NPC, Johannesburg, South Africa; g Department of Medicine, School of Medicine, Vanderbilt University, Nashville, TN, USA; h The Aurum Institute NPC, Gauteng, South Africa; i Setshaba Research Centre, Soshanguve, South Africa; j Perinatal HIV Research Unit, Department of Paediatrics and Child Health, School of Clinical Medicine, Faculty of Health Sciences, University of the Witwatersrand, Johannesburg, South Africa; k Clinical HIV Research Unit, Department of Internal Medicine, School of Clinical Medicine, Faculty of Health Sciences, University of the Witwatersrand, Johannesburg, South Africa; l Otsuka Pharmaceutical Development & Commercialization, Rockville, MD, USA; m Otsuka Novel Products, Munich, Germany

## Abstract

**Background:**

Combined therapy with delamanid, bedaquiline, and quabodepistat (DBQ) showed potent early bactericidal activity and was well tolerated in a phase 2a, 14-day early bactericidal activity trial in participants with drug-susceptible pulmonary tuberculosis. This subsequent proof-of-concept trial evaluated the efficacy and safety of a 4-month, three-dose level regimen of DBQ in participants with drug-susceptible pulmonary tuberculosis compared with 6 months of the standard of care.

**Methods:**

This open-label, randomised, proof-of-concept, non-inferiority, phase 2b/c trial was conducted at six clinical research sites in South Africa. Adults aged 18–65 years with newly diagnosed, drug-susceptible pulmonary tuberculosis were recruited by research sites from community tuberculosis clinics. Participants were randomly assigned (1:2:2:1) by balanced block randomisation (block size six) to receive oral delamanid (300 mg once a day) and bedaquiline (400 mg once a day for 2 weeks then 200 mg three times a week) plus quabodepistat at once-daily doses of 10 mg (DBQ10 group), 30 mg (DBQ30 group), or 90 mg (DBQ90 group) for 4 months; or 6 months of RHEZ (rifampicin, isoniazid, ethambutol, and pyrazinamide for 8 weeks followed by 18 weeks of rifampin and isoniazid). All drugs were administered orally. Masking procedures were not used, although microbiology laboratory staff were masked to treatment assignment. The primary endpoints were the proportion of participants reaching sputum culture conversion by the end of the treatment period in the modified intention-to-treat (mITT) analysis set and safety in the safety analysis set. Non-inferiority, with a margin of 12%, was assessed for the primary endpoint in the mITT analysis set, comparing the pooled DBQ group and the RHEZ group using a two-sided 80% CI. This study was registered at ClinicalTrials.gov, NCT05221502, and is complete.

**Findings:**

Between April 12, 2022, and May 19, 2024, 306 individuals were screened, of whom 122 were enrolled and randomly assigned: 20 to the DBQ10 group, 42 to the DBQ30 group, 39 to the DBQ90 group, and 21 to the RHEZ group. 121 participants received at least one dose of study drug and comprised the mITT analysis set. At the end of the treatment period, the proportion of participants with sputum culture conversion was 100·0% (95% CI 83·2–100·0, all 20 participants) in the DBQ10 group, 92·9% (80·5–98·5, 39 of 42 participants) in the DBQ30 group, 97·4% (86·2–99·9, 37 of 38 participants) in the DBQ90 group, 96·0% (90·1–98·9, 96 of 100 participants) in the pooled DBQ group, and 100·0% (83·9–100·0, all 21 participants) in the RHEZ group. The comparison between the pooled DBQ group and the RHEZ group met non-inferiority for the primary endpoint (difference −4·0% [80% CI −7·4 to 3·4]). Adverse events were mostly mild to moderate, with no serious adverse events or discontinuations attributed to trial medications. Rates of adverse events of grade 3 or higher were 20% (four of 20 participants) in the DBQ10 group, 14% (six of 42 participants) in the DBQ30 group, 11% (four of 38 participants) in the DBQ90 group, and 5% (one of 21 participants) in the RHEZ group. One participant (in the DBQ90 group) died after worsening pulmonary tuberculosis with pneumonia, which was assessed as not related to study treatment. 105 (87%) of 121 participants had at least one treatment-emergent adverse event: 17 (85%) of 20 in the DBQ10 group, 37 (88%) of 42 in the DBQ30 group, 30 (79%) of 38 in the DBQ90 group, and all 21 (100%) participants in the RHEZ group. The most common treatment-emergent adverse events in the total population of 121 participants were upper respiratory tract infection (n=36, 30%), headache (n=24, 20%), and diarrhoea (n=12, 10%).

**Interpretation:**

In this proof-of-concept study, 4 months of DBQ showed a promising end-of-treatment non-inferiority signal versus 6 months of RHEZ and was generally well tolerated. Our results support further studies of quabodepistat as a potential component of new treatment-shortening regimens for tuberculosis.

**Funding:**

Otsuka Pharmaceutical Development & Commercialization, with partial funding from the Gates Foundation.

**Research in context**

**Evidence before this study**

Antituberculosis drugs that have novel mechanisms of action and are well tolerated are urgently needed to shorten the duration of treatment. We searched PubMed for records published in English from database inception to Jan 26, 2026, using the terms “OPC-167832” and/or “Quabodepistat”. 16 records were identified. Quabodepistat (formerly OPC-167832), a 3,4-dihydrocarbostyril derivative, is being studied as a part of a regimen backbone for the treatment of drug-susceptible tuberculosis. Several preclinical studies found that quabodepistat was effective in vitro and in vivo against both drug-susceptible and drug-resistant strains of *Mycobacterium tuberculosis*. A 14-day early bactericidal activity trial in participants with drug-susceptible tuberculosis investigated quabodepistat both as monotherapy and in combination with delamanid and bedaquiline. Both monotherapy and combination therapy appeared to have an acceptable safety profile and be well tolerated. However, to our knowledge, quabodepistat has not been studied for treatment durations of longer than 14 days.

**Added value of this study**

This non-inferiority, proof-of-concept trial compared 4 months of treatment with an antituberculosis treatment regimen containing quabodepistat, delamanid, and bedaquiline with the current 6-month standard of care for drug-susceptible tuberculosis. The non-inferiority margin was met for the primary endpoint of sputum culture conversion at the end of treatment. Additionally, hypothesis-generating exploratory endpoints showed favourable long-term relapse rates. Our data support the potential of quabodepistat, delamanid, and bedaquiline as a component of treatment-shortening regimens.

**Implications of all the available evidence**

The data presented here, together with the previously identified similar potencies of quabodepistat on drug-susceptible and drug-resistant strains of tuberculosis, suggest that a quabodepistat-containing regimen could be a therapeutic option for drug-resistant tuberculosis. A phase 3 clinical trial (NCT07209761) is currently ongoing to test this hypothesis.

## Introduction

Tuberculosis, caused by the acid-fast bacillus *Mycobacterium tuberculosis*,^1^ remains one of the world's leading causes of death from a single infectious agent.^2^ Current therapies for drug-susceptible and drug-resistant tuberculosis are effective; however, the duration of treatment is long, and toxicity and tolerability are a challenge, especially in the treatment of drug-resistant tuberculosis.^2^, ^3^, ^4^, ^5^ There is an unmet need for the development of drugs that have novel mechanisms of action, have reduced toxicities, and are well tolerated, for inclusion in treatment-shortening regimens for drug-susceptible and drug-resistant tuberculosis.^6^

Quabodepistat (formerly OPC-167832) is an oral antituberculosis agent that inhibits decaprenylphosphoryl-β-d-ribose-2′-oxidase, an enzyme essential for cell-wall biosynthesis in *M tuberculosis*;^7^, ^8^ this mechanism differs from that of currently available anti-tuberculosis drugs. In non-clinical studies, quabodepistat had low minimum inhibitory concentrations (MICs) against both standard and clinically isolated strains of *M tuberculosis*, including those resistant to first-line and second-line agents.^7^ High bactericidal activity was observed in in-vitro and in-vivo studies.^7^ Quabodepistat has shown increased MICs in laboratory strains of *M tuberculosis* with *Rv0678* mutations, which are known to lead to bedaquiline and clofazimine resistance through the upregulation of efflux pumps; however, this increase is only approximately two to four-fold, and the MIC remains sufficiently low.^7^

In previous ascending-dose studies, quabodepistat doses ranging from 3 mg to 480 mg were well tolerated in healthy adults.^9^ In a phase 1/2 clinical trial in adults (aged 18–64 years) with drug-susceptible tuberculosis, monotherapy with quabodepistat doses ranging from 3 mg to 90 mg for 14 days had high early bactericidal activity and was considered well tolerated; however, no clear dose response was observed.^9^ Preliminary exposure–response modelling (Sasaki T et al, Otsuka, unpublished) identified an optimal dose of 30 mg quabodepistat administered once a day. Further, in participants with drug-susceptible tuberculosis, 30 mg quabodepistat in combination with delamanid and bedaquiline for 14 days showed a favourable safety profile and similar early bactericidal activity to that of RHEZ (rifampicin, isoniazid, ethambutol, and pyrazinamide), the standard-of-care treatment.^10^

A clinical development programme is under way with the aim to develop quabodepistat as part of an effective regimen with currently available antituberculosis drugs, including delamanid and bedaquiline, to not only shorten treatment duration but also be less toxic and better tolerated than current treatment regimens for patients with drug-resistant tuberculosis. In this current proof-of-concept trial, which expands on the knowledge gained in previous trials, we evaluate the efficacy and safety of a 4-month treatment with quabodepistat, delamanid, and bedaquiline (hereafter referred to as DBQ) in participants with drug-susceptible tuberculosis compared with 6 months of the standard of care (RHEZ). Because quabodepistat has a novel mechanism of action and shows in-vitro potency against both drug-susceptible and drug-resistant *M tuberculosis*, clinical efficacy data in drug-susceptible tuberculosis—together with non-clinical (ie, in-vitro, pharmacokinetic and pharmacodynamic, and animal) data^7^, ^9^, ^10^—provide support for the biological plausibility of activity in drug-resistant tuberculosis and can inform the design and feasibility of drug-resistant tuberculosis trials. As this study is of longer duration than those previously conducted, three dose levels of quabodepistat were used to provide ongoing determination of an optimal dose. We hypothesised that the pooled quabodepistat-containing regimens would show non-inferiority compared with the standard of care in the primary endpoint of proportion of participants having sputum culture conversion by the end of treatment (4 months for the quabodepistat-containing regimens, 6 months for RHEZ). The primary objective of the trial was to assess the safety and efficacy of DBQ administered for 4 months compared with RHEZ administered for 6 months in participants with drug-susceptible tuberculosis. Data obtained from this trial informed the development of a phase 3 clinical trial of quabodepistat-containing regimens in participants with drug-resistant tuberculosis.

## Methods

### Study design

This open-label, randomised, proof-of-concept, non-inferiority phase 2b/c trial was conducted at six clinical research sites in South Africa. Full details of the trial design have been published^11^ and are provided in the study protocol (appendix 1 pp 112–113, 126–128) and statistical analysis plan (appendix 1 pp 192–194). There was no patient or public involvement in the study design, conduct, or reporting.

The trial was conducted in accordance with US Food and Drug Administration regulations, the International Council for Harmonisation Good Clinical Practice Guideline, principles of the Declaration of Helsinki, and Council for International Organizations of Medical Science guidelines, and applicable local laws and regulations. An independent data monitoring committee, comprising two clinicians and one biostatistician, reviewed safety, efficacy, and pharmacokinetic data during the trial. An institutional review board or independent ethics committee at each trial site (appendix 1 p 19) reviewed and approved the protocol, informed-consent documents, and the trial.

### Participants

Participants were recruited by research sites from community tuberculosis clinics. Eligible participants were aged 18–65 years and had newly diagnosed pulmonary tuberculosis that was confirmed by sputum culture to be susceptible to rifampicin and isoniazid. Full inclusion and exclusion criteria are provided in appendix 1 (pp 2–3). In brief, eligible participants were aged 18–65 years at the time of screening and had newly diagnosed pulmonary drug-susceptible tuberculosis, confirmed by sputum culture to be susceptible to rifampicin and isoniazid, with no previous tuberculosis treatment and no clinically significant comorbidities that could compromise safety or study participation. Participants self-reported sex at birth and gender, with the options of male or female. All participants provided written informed consent before screening. The trial is registered with ClinicalTrials.gov, NCT05221502, and is complete.

### Randomisation and masking

Randomisation was done centrally via an interactive web response technology system (the study sites did not have access to the randomisation list). Balanced block randomisation was used with a block size of six. Participants were randomly assigned, in a 1:2:2:1 ratio, to one of three quabodepistat treatment groups, which received delamanid and bedaquiline plus either 10 mg (DBQ10 group), 30 mg (DBQ30 group), or 90 mg (DBQ90 group) quabodepistat, or to the control group, which received RHEZ (appendix 1 p 13). Because RHEZ is well characterised, the 1:2:2:1 ratio was chosen to allow more information—such as safety data—to be gathered regarding the experimental regimens, specifically the 30 mg and 90 mg doses of quabodepistat, which were considered to be of interest on the basis of previous data. Randomisation was to be stratified according to HIV status (yes or no) and presence of bilateral cavitation on screening chest x-ray (yes or no); however, no individuals living with HIV fulfilled the eligibility criteria. Except for the microbiology laboratory staff being masked to the treatment assignment of each participant, this trial did not use masking procedures, and the success of masking was not formally assessed.

### Procedures

The trial included a screening period of up to and including 14 days, a 4-month or 6-month treatment period, and a post-randomisation follow-up period until month 12 (appendix 1 p 14). The full schedule of study visits is provided in the protocol (appendix 1 pp 114–118).

At enrolment, *M tuberculosis* was identified by sputum microscopy or a WHO-recommended molecular rapid diagnostic test.^12^ Sputum samples were obtained at each study visit and processed using the Mycobacteria Growth Indicator Tube (MGIT) system. Microbiological relapse was determined through genotyping using whole-genome sequencing, and was defined as the same strain of *M tuberculosis* identified before treatment causing recurrence after a successful treatment (ie, reaching sputum culture conversion). *M tuberculosis* genotyping was conducted on a baseline culture (day –1 [the day before the first dose] and/or day 1 [the day of the first dose], or week 1, if necessary) and on the first of any subsequent positive culture during the post-treatment follow-up period. If genotyping identified that an unrelated strain caused the recurrence, this was considered a reinfection rather than a relapse.

Each quabodepistat treatment group received delamanid (300 mg once a day) and bedaquiline (400 mg once a day for 2 weeks, then 200 mg three times per week with at least 48 h between doses) plus quabodepistat at once-daily doses of 10 mg (DBQ10 group), 30 mg (DBQ30 group), or 90 mg (DBQ90 group) for 4 months (ie, 17 weeks). The control group received 8 weeks of RHEZ (a single, fixed-dose combination tablet of 150 mg rifampicin, 75 mg isoniazid, 275 mg ethambutol, and 400 mg pyrazinamide, once a day), then 18 weeks of rifampicin and isoniazid as per the local standard of care.

Participants in the DBQ groups took the study drugs orally after a meal; those in the RHEZ group took the study drug orally 1 h before or 2 h after a meal, all with approximately 240 mL of water. Locally accepted adherence-monitoring techniques were used, which included directly observed therapy, Wisepill (Wisepill Technologies, Cape Town, South Africa), video, or other adherence-monitoring techniques. All doses of study drugs given during trial visits were observed via the hand-and-mouth procedure. Adherence was calculated by dividing the number of doses missed during the treatment period by the number of doses expected to be taken during the treatment period.

Quabodepistat and delamanid were manufactured by Otsuka Pharmaceutical (Tokyo, Japan). Bedaquiline and RHEZ were locally sourced by the study sponsor.

### Outcomes

The alignment of the trial endpoints with the study objectives is reported in appendix 1 (p 4). In brief, the primary objective was to assess efficacy and safety of 4 months of treatment with DBQ compared with 6 months of treatment with RHEZ. The primary efficacy endpoint was the proportion of participants who had sputum culture conversion and no subsequent positive culture by the end of the treatment period. Sputum culture conversion was defined as occurring when the first of two sputum cultures at least 1 week apart was negative without a positive sputum culture result in between. Secondary efficacy endpoints reported here included the proportion of participants with sputum culture conversion in the MGIT system by week 8 of treatment, the proportion of participants who developed drug resistance, and the time to sputum culture conversion. The secondary efficacy endpoints of the proportion of participants who converted sputum lipoarabinomannan to negative by 8 weeks of treatment and by the end of treatment, and the time to detection of MGIT cultures, will be presented in a subsequent publication.

Exploratory efficacy endpoints reported here included the proportion of participants with a favourable outcome (appendix 1 p 110) at 12 months post-randomisation, the proportion of participants with a favourable outcome at 6 months after the end of treatment, and the proportion of participants with relapse at 12 months post-randomisation. The relapse rate 6 months after the end of treatment was assessed as a post-hoc analysis. 12 months post-randomisation is an 8-month follow-up period for the DBQ groups and a 6-month follow-up period for the RHEZ group. Because the DBQ groups have two additional months of follow-up, a post-hoc analysis was conducted 6 months after the end of treatment, so that a 6-month follow-up could be analysed for the DBQ groups and for the RHEZ group. A complete list of study endpoints can be found in the protocol (appendix 1 pp 124–125).

The primary safety endpoints were adverse events, clinical laboratory tests, vital signs, physical examination findings, and electrocardiographic results (done in triplicate, centrally read by board-certified cardiologists, and collected at screening, at baseline, and at weeks 2, 4, 6, 8, 10, 12, 13, 15, 17, 21, and 26 of the treatment period, as well as weeks 30, 34, 39, 43, 47, and 52 in the follow-up period); the proportion of participants with an adverse event of grade 3 or higher (based on the Division of AIDS criteria^13^); and the rate of all-cause treatment discontinuation. The total duration of follow-up for safety endpoints was 12 months post-randomisation.

### Statistical analysis

This Article presents data for individual groups and the pooled DBQ group. Because no major differences were observed between quabodepistat dose groups in previous trials,^9^, ^10^ the pooled DBQ group provided additional statistical power to draw comparisons and to avoid multiplicity. This trial used an 80% CI for the difference in proportion between treatment groups to compare with the non-inferiority margin. Assuming that 93% of participants in the RHEZ group and the same proportion in the pooled DBQ group would meet the primary efficacy endpoint, a sample size of 120 participants (100 in the pooled DBQ group and 20 in the RHEZ group) was estimated to provide 84% power to test the hypothesis that the 4-month regimen of DBQ would be non-inferior to the 6-month regimen of RHEZ. In this proof-of-concept study, we used a non-inferiority margin of 12% and a one-sided α of 0·1. Of note, a two-sided 80% CI corresponds to a two-sided α of 0·2, which is equivalent to a one-sided α of 0·1. The 12% non-inferiority margin is consistent with that of other trials that are ongoing.^14^, ^15^, ^16^ When pooling the three DBQ groups to compare with the control group, the allocation ratio became 5:1. The sample size calculation was therefore based on the allocation ratio of 5:1; however, the actual randomisation was based on a 1:2:2:1 ratio. Sample size calculations were conducted using PASS software (NCCS, Kaysville, UT, USA) with the procedure entitled non-inferiority tests for the difference between two proportions. Non-inferiority was shown if the lower limit of the two-sided 80% CI around the difference between the pooled DBQ group and the RHEZ group was larger than −0·12, as prespecified in the statistical analysis plan (appendix 1 pp 194–195). We assessed non-inferiority in sputum culture conversion at the end of treatment (4 months for DBQ groups and 6 months for the RHEZ group) to determine if the DBQ regimen had treatment-shortening potential. The Miettinen–Nurminen CI of common risk difference was used,^17^ stratified by bilateral cavitation status before randomisation. The same methods used for analysis of the primary endpoint were used for the analysis of the secondary efficacy endpoints of the proportions of participants with sputum culture conversion in the MGIT system by week 8 and the proportion of participants who developed drug resistance. Safety endpoints were summarised using descriptive statistics (ie, number, median, mean, SD, minimum, and maximum) for continuous values, and by frequency and percentage for discrete variables. Additional statistical methods can be found in the statistical analysis plan (appendix 1 p 199).

The safety analysis set included all randomly assigned participants who received any dose of study drug. The modified intention-to-treat (mITT) analysis set included all randomly assigned participants who took at least one dose of study drug (ie, delamanid, bedaquiline, or quabodepistat) or RHEZ, had at least one post-baseline culture result, and had positive culture results by 1 week and susceptibility to rifampicin and isoniazid (from the screening specimen). The per-protocol analysis set comprised participants from the mITT analysis set excluding those who met any of the following criteria: did not meet eligibility criteria; had adherence of 80% or less to any of the study drugs or discontinued any study drug for a reason other than death, tuberculosis or tuberculosis-related disease, or had major treatment adherence protocol violations; or had major protocol deviations that affect the primary efficacy analyses. All efficacy results presented here are from the mITT analysis set, with the exception of the primary endpoint, in which the results for the per-protocol analysis set are provided. The primary endpoint was analysed in both the mITT and per-protocol populations to assess the consistency of the treatment effect across analytical populations. Secondary endpoints were analysed in the mITT population only, as they were supportive and not intended for confirmatory inference.

Analyses were conducted using SAS version 9.4. There were no protocol, standard operating procedure, or method deviations in the conduct of this study (appendix 1 p 82).

### Role of the funding source

The study design and the collection, analysis, and interpretation of data were funded by Otsuka Pharmaceutical Development & Commercialization (sponsor), with partial funding from the Gates Foundation. The sponsor was involved in the development and preparation of this manuscript. The Gates Foundation had no role in study design, data collection, data analysis, data interpretation, or writing of the report.

## Results

The trial took place between April 12, 2022, and May 19, 2024, at six clinical research sites in South Africa. 306 individuals were screened, of whom 122 were enrolled and randomly assigned: 20 to the DBQ10 group, 42 to the DBQ30 group, 39 to the DBQ90 group, and 21 to the RHEZ group. 121 participants took at least one dose of DBQ or RHEZ and were included in the efficacy and safety analyses (figure). Study completion rates were high and were similar between groups. 79 (65%) of 121 participants for whom data were available were male and 42 (34%) were female. Most (83 [68%] of 122) participants were Black, with 39 (32%) self-reporting as another race. The mean age of participants was 33·7 years (SD 11·7, range 18–65), with a mean bodyweight of 55·7 kg (11·2, 39·0–129·0; table 1).

**Figure:**
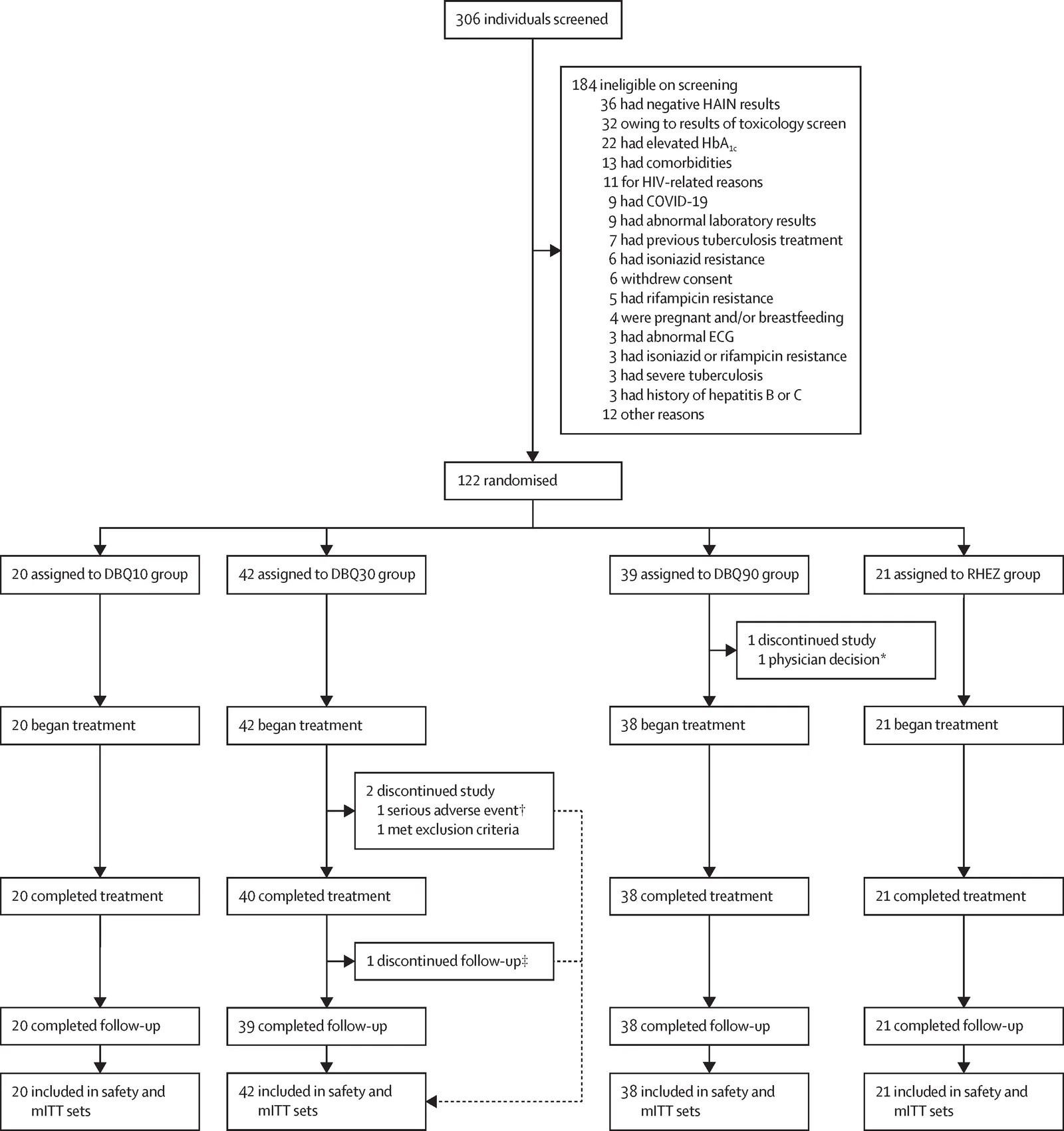
Trial profile DBQ=delamanid and bedaquiline plus quabodepistat; numbers refer to the dose in mg of quabodepistat. ECG=electrocardiogram. HAIN=HAINS Mycobacterium Tuberculosis First and Second Line Drug Resistance Test. HbA_1c_=glycated haemoglobin. mITT=modified intention-to-treat. RHEZ=rifampicin, isoniazid, ethambutol, and pyrazinamide. *Participant withdrew from study and did not begin treatment owing to borderline eligibility. †Participant discontinued owing to a serious adverse event of pneumonia and worsening tuberculosis. ‡One participant missed visit 39 onwards. This participant did not withdraw consent, and was reached via telephone.

**Table 1:** Demographics and clinical characteristics of participants at baseline

			**DBQ10 group (n=20)**	**DBQ30 group (n=42)**	**DBQ90 group (n=39)**	**Pooled DBQ group (n=101)**	**RHEZ group (n=21)**	**Total (N=122)**
Sex at birth*								
	Male		14 (70%)	27 (64%)	26 (67%)	67 (66%)	12 (57%)	79 (65%)
	Female		6 (30%)	14 (33%)	13 (33%)	33 (33%)	9 (43%)	42 (34%)
Mean age, years (SD, range)			37·7 (11·9, 21–62)	32·8 (11·9, 19–64)	33·2 (12·1, 18–65)	33·9 (12·0, 18–65)	32·7 (9·9, 18–56)	33·7 (11·7, 18–65)
Race								
	Black		13 (65%)	27 (64%)	29 (74%)	69 (68%)	14 (67%)	83 (68%)
	Other		7 (35%)	15 (36%)	10 (26%)	32 (32%)	7 (33%)	39 (32%)
Mean bodyweight, kg (SD, range)			54·1 (7·3, 42·0–75·0)	56·7 (14·8, 39·0–129·0)	56·0 (9·9, 39·0–81·0)	55·9 (11·7, 39·0–129·0)	54.4 (8·2, 39·0–74·0)	55·7 (11·2, 39·0–129·0)
Cavitation on chest x-ray								
	No		4 (20%)	14 (33%)	9 (23%)	27 (27%)	6 (29%)	33 (27%)
	Yes		16 (80%)	28 (67%)	30 (77%)	74 (73%)	15 (71%)	89 (73%)
		Unilateral	14 (88%)	22 (79%)	25 (83%)	61 (82%)	11 (73%)	72 (81%)
		Bilateral	2 (13%)	6 (21%)	5 (17%)	13 (18%)	4 (27%)	17 (19%)
Tuberculosis treatment history								
	Multidrug-resistant tuberculosis		1 (5%)	0	0	1 (1%)	0	1 (1%)
	Pre-extensively drug-resistant or extensively drug-resistant tuberculosis		0	0	0	0	0	0
	Drug-susceptible tuberculosis		3 (15%)	7 (17%)	4 (10%)	14 (14%)	6 (29%)	20 (16%)

Data are n (%) unless otherwise indicated. Data are provided for all randomly assigned participants. No participants were of Hispanic or Latino ethnicity. Percentages might not total 100 because of rounding. DBQ=delamanid and bedaquiline plus quabodepistat; numbers refer to the dose in mg of quabodepistat. RHEZ=rifampicin, isoniazid, ethambutol, and pyrazinamide.

* n=41 for the DBQ30 group, n=100 for the pooled DBQ group, and n=121 for total.

The proportion of participants who had sputum culture conversion by the end of the treatment period (mITT analysis set) was 100·0% (95% CI 83·2–100·0, all 20 participants) in the DBQ10 group, 92·9% (80·5–98·5, 39 of 42) in the DBQ30 group, 97·4% (86·2–99·9, 37 of 38) in the DBQ90 group, 96·0% (90·1–98·9, 96 of 100) in the pooled DBQ group, and 100·0% (83·9–100·0, all 21 participants) in the RHEZ group. The difference between the pooled DBQ group and the RHEZ group was −4·0 (80% CI −7·4 to 3·4); non-inferiority was therefore established (table 2). As prespecified, the three DBQ groups were pooled for the primary analysis to avoid multiplicity and to increase power, given the similar efficacy in each dose group. Sputum culture conversion rates were consistently high across doses (92·9–100·0%), and no pairwise dose comparisons were conducted. A global Cochran–Armitage trend test showed no evidence of a dose–response relationship (p=0·60). Similar results were observed for the per-protocol analysis set (table 2). Sputum culture conversion rates stratified by bilateral cavitation status are provided in appendix 1 (p 5), and suggest that participants with bilateral cavitation did not have poorer outcomes than those without.

**Table 2:** Proportion of participants with sputum culture conversion by the end of the treatment period

	**DBQ10 group (n=20)**	**DBQ30 group (n=42)**	**DBQ90 group (n=38)**	**Pooled DBQ group (n=100)**	**RHEZ group (n=21)**
**mITT analysis set**					
Proportion of participants with sputum culture conversion (95% CI)	100·0% (83·2–100·0), n=20	92·9% (80·5–98·5), n=39	97·4% (86·2–99·9), n=37	96·0% (90·1–98·9), n=96	100·0% (83·9–100·0), n=21
Difference (80% CI)*	..	..	..	−4·0 (−7·4 to 3·4)	..
Non-inferiority†	..	..	..	Yes	..
**Per-protocol analysis set**‡					
Proportion of participants with sputum culture conversion (95% CI)	100·0% (83·2–100·0), n=20	95·1% (83·5–99·4), n=39	97·4% (86·2–99·9), n=37	97·0% (91·4–99·4), n=96	100·0% (83·9–100·0), n=21
Difference (80% CI)*	..	..	..	−3·0 (−6·1 to 4·3)	..
Non-inferiority†	..	..	..	Yes	..

DBQ=delamanid and bedaquiline plus quabodepistat; numbers refer to the dose in mg of quabodepistat. mITT=modified intention-to-treat. RHEZ=rifampicin, isoniazid, ethambutol, and pyrazinamide.

* The difference was measured between the pooled DBQ group and the RHEZ group.

† Non-inferiority was assessed for the pooled DBQ group compared with the RHEZ group.

‡ For the per-protocol analysis set, n=41 for the DBQ30 group and n=99 for the pooled DBQ group.

The proportion of participants who had sputum culture conversion at 8 weeks was 40·0% (95% CI 19·1–63·9, eight of 20 participants) in the DBQ10 group, 57·1% (41·0–72·3, 24 of 42) in the DBQ30 group, 50·0% (33·4–66·6, 19 of 38) in the DBQ90 group, 51·0% (40·8–61·1, 51 of 100) in the pooled DBQ group, and 52·4% (29·8–74·3, 11 of 21) in the RHEZ group (table 3). Sputum culture conversion rates by study week are presented in appendix 1 (p 6).

**Table 3:** Secondary efficacy outcomes in the mITT population

	**DBQ10 group (n=20)**	**DBQ30 group (n=42)**	**DBQ90 group (n=38)**	**Pooled DBQ group (n=100)**	**RHEZ group (n=21)**
Proportion of participants with sputum culture conversion at 8 weeks (95% CI)	40·0% (19·1–63·9), n=8	57·1% (41·0–72·3), n=24	50·0% (33·4–66·6), n=19	51·0% (40·8–61·1), n=51	52·4% (29·8–74·3), n=11
Proportion of participants who developed drug resistance (95% CI)	0·0% (0·0–16·8), n=0	0·0% (0·0–8·4), n=0	0·0% (0·0–9·3), n=0	0·0% (0·0–3·7), n=0	0·0% (0·0–16·1), n=0

DBQ=delamanid and bedaquiline plus quabodepistat; numbers refer to the dose in mg of quabodepistat. mITT=modified intention-to-treat. RHEZ=rifampicin, isoniazid, ethambutol, and pyrazinamide.

Kaplan–Meier estimates of the time to sputum culture conversion by the end of treatment are shown in appendix 1 (p 15) by treatment group. None of the 121 participants, including those with relapse in the DBQ groups, acquired resistance during treatment or follow-up to delamanid, bedaquiline, or other tested agents (ie, rifampicin, ethambutol, pyrazinamide, streptomycin, and isoniazid), based on results from appropriate phenotypic drug-susceptibility testing (table 3). Determination of the MIC for quabodepistat is currently ongoing.

For the exploratory outcomes at 12 months post-randomisation, the proportion of participants with favourable outcome was 85·0% (95% CI 62·1–96·8, 17 of 20 participants) in the DBQ10 group, 81·0% (65·9–91·4, 34 of 42) in the DBQ30 group, 84·2% (68·7–94·0, 32 of 38) in the DBQ90 group, 83·0% (74·2–89·8, 83 of 100) in the pooled DBQ group, and 95·2% (76·2–99·9, 20 of 21) in the RHEZ group (table 4).

**Table 4:** Exploratory efficacy outcomes in the mITT population

	**DBQ10 group (n=20)**	**DBQ30 group (n=42)**	**DBQ90 group (n=38)**	**Pooled DBQ group (n=100)**	**RHEZ group (n=21)**
Favourable outcome*					
Proportion of participants with a favourable outcome at 12 months post-randomisation (95% CI)	85·0% (62·1–96·8), n=17	81·0% (65·9–91·4), n=34	84·2% (68·7–94·0), n=32	83·0% (74·2–89·8), n=83	95·2% (76·2–99·9), n=20
Proportion of participants with a favourable outcome 6 months after the end of treatment (95% CI)	85·0% (62·1–96·8), n=17	85·7% (71·5–94·6), n=36	81·6% (65·7–92·3), n=31	84·0% (75·3–90·6), n=84	90·5% (69·6–98·8), n=19
Not assessable, n (%)	0	1 (2%)	0	1 (1%)	0
**Unfavourable outcome**†					
Proportion of participants with an unfavourable outcome at 12 months post-randomisation (95% CI)	15·0% (3·2–37·9), n=3	16·7% (7·0–31·4), n=7	15·8% (6·0–31·3), n=6	16·0% (9·4–24·7), n=16	4·8% (0·1–23·8), n=1
Proportion of participants with an unfavourable outcome 6 months after the end of treatment (95% CI)	15·0% (3·2–37·9), n=3	11·9% (4·0–25·6), n=5	15·8% (6·0–31·3), n=6	14·0% (7·9–22·4), n=14	9·5% (1·2–30·4), n=2
Not assessable, n (%)	0	1 (2%)	1 (3%)	2 (2%)	0
**Relapse**					
Proportion of participants with relapse at 12 months post-randomisation (95% CI)	10·0% (1·2–31·7), n=2	11·9% (4·0–25·6), n=5	13·2% (4·4–28·1), n=5	12·0% (6·4–20·0), n=12	4·8% (0·1–23·8), n=1
Proportion of participants with relapse 6 months after the end of treatment (95% CI‡)	0·0% (0·0–16·8), n=0	7·1% (1·5–19·5), n=3	10·5% (2·9–24·8), n=4	7·0% (2·9–13·9), n=7	4·8% (0·1–23·8), n=1

95% CIs were calculated using the Clopper–Pearson Exact method. DBQ=delamanid and bedaquiline plus quabodepistat; numbers refer to the dose in mg of quabodepistat. mITT=modified intention-to-treat. RHEZ=rifampicin, isoniazid, ethambutol, and pyrazinamide.

* A participant was considered to have a favourable outcome if, during the post-randomisation evaluation period, the participant was alive, completed the post-randomisation evaluation, and otherwise did not meet the definition of an unfavourable outcome (appendix 1 p 110).

† A participant was considered to have an unfavourable outcome if one of the following occurred: (1) clinical progression of pulmonary disease during treatment or the need for an unanticipated surgical intervention for tuberculosis (eg, lobectomy); (2) any growth of *Mycobacterium tuberculosis* from an extrapulmonary site during the post-treatment period (excludes reinfection with a new strain of *M tuberculosis*); (3) signs and symptoms of active tuberculosis during the follow-up period (ie, after the end of treatment), including radiographic worsening compared with baseline findings, resulting in re-initiation of anti-mycobacterial therapy (excludes a re-initiation that is subsequently found to be due to a misdiagnosis and/or alternative diagnosis); (4) death during treatment or follow-up; (5) a sputum culture with growth of *M tuberculosis* at particular timepoints as follows: not reaching sputum culture conversion by the end of the treatment period resulting in a change in anti-mycobacterial therapy; microbiological relapse during the follow-up period (ie, after the end of treatment; excludes reinfection and requires more than one positive sputum culture with a new strain of *M tuberculosis*; appendix 1 p 110).

‡ This was a post-hoc analysis. Three participants were not assessable for the relapse outcome at 6 months after the end of treatment; these participants are included in the denominator for percentages.

6 months after the end of treatment, the favourable outcome rates were 85·0% (95% CI 62·1–96·8, 17 of 20 participants) in the DBQ10 group, 85·7% (71·5–94·6, 36 of 42) in the DBQ30 group, 81·6% (65·7–92·3, 31 of 38) in the DBQ90 group, 84·0% (75·3–90·6, 84 of 100) in the pooled DBQ group, and 90·5% (69·6–98·8, 19 of 21) in the RHEZ group (table 4).

10·0% (95% CI 1·2–31·7, two of 20) of participants in the DBQ10 group, 11·9% (4·0–25·6, five of 42) in the DBQ30 group, 13·2% (4·4–28·1, five of 38) in the DBQ90 group, 12·0% (6·4–20·0, 12 of 100) in the pooled DBQ group, and 4·8% (0·1–23·8, one of 21) in the RHEZ group had relapse at 12 months post-randomisation (table 4). 21 participants had genotyping for positive post-treatment cultures (appendix 1 p 7).

A post-hoc analysis assessed relapse at 6 months after the end of treatment. Rates were 0·0% (95% CI 0·0–16·8, zero of 20 participants) in the DBQ10 group, 7·1% (1·5–19·5, three of 42) in the DBQ30 group, 10·5% (2·9–24·8, four of 38) in the DBQ90 group, 7·0% (2·9–13·9, seven of 100) in the pooled DBQ group, and 4·8% (0·1–23·8, one of 21) in the RHEZ group (table 4).

Adherence was high, with participants receiving at least 98% of the planned drug exposure in all groups. Values were 100% in the DBQ10 group, 98% in the DBQ30 group, 100% in the DBQ90 group, and 98% in the RHEZ group.

105 (87%) of 121 participants had at least one treatment-emergent adverse event: 17 (85%) of 20 in the DBQ10 group, 37 (88%) of 42 in the DBQ30 group, 30 (79%) of 38 in the DBQ90 group, and all 21 (100%) participants in the RHEZ group (table 5). Details of the adverse events are provided in appendix 1 (pp 8–12). The proportions of participants who had an adverse event of at least grade 3 (a primary safety outcome) were 20% (four of 20) in the DBQ10 group, 14% (six of 42) in the DBQ30 group, 11% (four of 38) in the DBQ90 group, 14% (14 of 100) in the pooled DBQ group, and 5% (one of 21) in the RHEZ group. One (1%) of 121 participants, who was from the DBQ30 group, discontinued the trial owing to a treatment-emergent adverse event (pneumonia assessed as not related to the study drugs; table 5). There were no treatment discontinuations owing to an adverse event related to any of the study drugs or treatment tolerability. Five participants had seven serious treatment-emergent adverse events (two events in the DBQ10 group [appendicitis perforated and localised infection] and five events in the DBQ30 group [pneumonia, tuberculosis, hyperkalaemia, myelopathy, and haemoptysis]). All serious events were reported as recovered/resolved or recovered/resolved with sequelae, except for in one participant (in the DBQ30 group), who discontinued the trial at 8 weeks owing to worsening pulmonary tuberculosis complicated by severe bacterial pneumonia and was hospitalised and initiated on standard-of-care treatment. The participant died 24 days after hospitalisation. In this participant, baseline resistance testing showed susceptibility to all drugs tested (ie, isoniazid, rifampicin, delamanid, bedaquiline, ethambutol, streptomycin, and pyrazinamide), and quabodepistat MICs were within the expected range. None of the serious adverse events were deemed related to the study drugs (appendix 1 p 11).

**Table 5:** Safety analysis in the safety analysis set

	**DBQ10 group (n=20)**	**DBQ30 group (n=42)**	**DBQ90 group (n=38)**	**Pooled DBQ group (n=100)**	**RHEZ group (n=21)**	**Total (N=121)**
Treatment-emergent adverse events	17 (85%)	37 (88%)	30 (79%)	84 (84%)	21 (100%)	105 (87%)
Serious treatment-emergent adverse events	2 (10%)	3 (7%)	0	5 (5%)	0	5 (4%)
Severe treatment-emergent adverse events	3 (15%)	5 (12%)	2 (5%)	10 (10%)	1 (5%)	11 (9%)
Treatment-emergent adverse events related to study drugs	4 (20%)	8 (19%)	5 (13%)	17 (17%)	6 (29%)	23 (19%)
Grade ≥3 treatment-emergent adverse events*	4 (20%)	6 (14%)	4 (11%)	14 (14%)	1 (5%)	15 (12%)
Treatment-emergent adverse events leading to study discontinuation	0	1 (2%)	0	1 (1%)	0	1 (1%)
Treatment-emergent adverse events related to study drugs	4 (20%)	8 (19%)	5 (13%)	17 (17%)	6 (29%)	23 (19%)
Treatment-emergent adverse events related to study drugs leading to study discontinuation	0	0	0	0	0	0
Treatment-emergent adverse events leading to death	0	1 (2%)	0	1 (1%)	0	1 (1%)

Data are n (%). DBQ=delamanid and bedaquiline plus quabodepistat; numbers refer to the dose in mg of quabodepistat. RHEZ=rifampicin, isoniazid, ethambutol, and pyrazinamide.

* Based on the Division of AIDS criteria.

Overall, liver function tests in DBQ groups showed values within normal reference ranges, with no participants meeting Hy's law definition of hepatotoxicity (appendix 1 p 16). In the DBQ10, DBQ30, DBQ90, and pooled DBQ groups, mean QT intervals corrected by Fridericia's method (QTcF) were all below 480 ms for each visit. No quabodepistat dose-dependent effects on QTcF or dose-dependent changes from baseline were observed (appendix 1 pp 13, 17) and no participants required electrolyte corrections. A mild decrease in estimated glomerular filtration rate (eGFR) from baseline was observed in participants in the DBQ groups (appendix 1 p 18), which did not appear to be dependent on the dose of quabodepistat. Values remained within the normal reference range during treatment and returned to baseline levels for all participants after week 17.

## Discussion

For both drug-susceptible and drug-resistant tuberculosis, there is an unmet need to develop novel, well tolerated drugs for inclusion in treatment-shortening regimens.^6^ In this proof-of-concept trial, compared to the standard of care for drug-susceptible tuberculosis (ie, 6 months of RHEZ treatment), 4 months of treatment with DBQ met the non-inferiority margin for the primary endpoint (proportion of participants who had sputum culture conversion by end of treatment). Evaluations of long-term exploratory outcomes (relapse and favourable outcome rates) were not statistically powered, therefore no statistical inference can be concluded; however, the pooled DBQ group appeared to have high treatment success rates, albeit numerically lower than those of the RHEZ group. No safety concerns were attributed to DBQ, which was generally well tolerated. Overall, of the 100 participants in the pooled DBQ group, 14% had an adverse event of at least grade 3, 5% had a serious adverse event, and 1% discontinued treatment owing to adverse events not related to study drugs. Moreover, there was a higher proportion of treatment-emergent adverse events related to the study drug in the RHEZ group (29%) than in the pooled DBQ group (17%). These findings corroborate the promising antimycobacterial potential of quabodepistat given alongside delamanid and bedaquiline; however, a phase 3 study with a relapse-based primary endpoint is required to show durability.

Demonstrating non-inferiority to the standard of care with fewer months of therapy can be challenging.^18^ The phase 3 REMoxTB trial, which assessed two moxifloxacin-containing 4-month combination therapies in comparison with 6 months of RHEZ, initially showed a more rapid decline in bacterial load with the moxifloxacin-containing regimens.^19^ However, these findings did not predict the risk of relapse, and non-inferiority was not shown for the treatment-shortening regimens.^19^ In this current trial, the exploratory endpoints of favourable outcome and relapse rates were similar for both pooled DBQ and RHEZ groups at 6 months after the end of treatment, with an equal follow-up time for both groups. However, the RHEZ group had numerically more favourable outcomes and fewer relapses than the pooled DBQ group at 12 months post-randomisation (after 8 months follow-up for DBQ groups and 6 months follow-up for the RHEZ group). The findings regarding sputum culture conversion at the end of treatment showed that treatment with the three-drug DBQ backbone leads to bacterial clearance at 4 months; however, long-term outcomes suggest that optimisation of this backbone regimen is required for sustained cure. This optimisation could involve extending the treatment duration or the addition of a fourth drug.

A previous trial, Pan-TB (NCT05971602), assessed the efficacy, safety, optimal duration, and pharmacokinetics of delamanid, bedaquiline, quabodepistat, and sutezolid (DBQS) and pretomanid, bedaquiline, quabodepistat, and sutezolid (PBQS) in adult (aged 18–65 years) participants with drug-susceptible tuberculosis, for potential shortening of the treatment duration (to ≤3 months).^20^ Although DBQS and PBQS showed bactericidal activity, the study was stopped early as the sputum culture conversion rates at 2 months and 3 months for both regimens did not support the trial objective of identifying a treatment regimen with a treatment duration of up to 3 months. No safety concerns were identified for either investigational group.^20^ The proof-of-concept study presented here is different in that it did not seek to identify a regimen with a treatment duration of 3 months or less, but rather to identify a backbone that can be used in its current form or optimised further for evaluation in late phase studies. This three-drug backbone was administered for 4 months, with the primary endpoint of sputum culture conversion evaluated at the end of the 4-month treatment period for the experimental groups and at 6 months for the RHEZ group, all of which showed favourable results.

The results of this proof-of-concept trial can be interpreted in the context of other contemporary regimen-shortening efforts for the treatment of tuberculosis, including TRUNCATE-TB,^16^, ^21^ SimpliciTB,^14^ and PAN-TB/XBOS,^22^, ^23^ which differ substantially in terms of design and endpoints. TRUNCATE-TB evaluated a response-adaptive strategy to treatment shortening, with regimen duration individualised on the basis of early disease response and a long-term unfavourable outcome endpoint, rather than testing a fixed universal regimen. By contrast, SimpliciTB assessed predefined shortened regimens using a non-inferiority framework and relapse-free cure as the primary outcome. PAN-TB/XBOS used a platform design spanning drug-susceptible and drug-resistant tuberculosis, prioritising regimen scalability and safety across resistance profiles, with distinct microbiological and clinical endpoints. These methodological differences limit direct comparisons between trials and underscore that apparent similarities or differences in outcomes could reflect trial design and endpoint selection rather than the intrinsic performance of the regimen.

Reports from some phase 2 and 3 trials of antituberculosis drugs have described hepatotoxic adverse events leading to the discontinuation of treatment.^14^, ^24^ In the current study, which had a 12-month safety timeframe, serum aspartate aminotransferase and alanine aminotransferase concentrations remained similar to baseline values in participants in the DBQ groups, and were higher in the RHEZ group during most of the treatment period than at baseline, indicating that DBQ did not markedly affect liver function. In terms of renal parameters, mean eGFR and creatinine concentrations were generally within normal ranges throughout the trial in the DBQ and RHEZ groups; however, a mild decrease in the mean eGFR was observed in the DBQ groups. This decrease will be further evaluated in future trials. Although prolongation of the QTc interval has been reported with delamanid and bedaquiline,^25^, ^26^ no participants in the current study had a recorded maximum QTcF interval of 500 ms or longer. In addition, mean QTcF change from baseline was less than 60 ms at all study visits, and changes observed were not dependent on the dose of quabodepistat.

Although most non-clinical and all clinical studies for quabodepistat to date have been conducted with drug-susceptible strains of *M tuberculosis* and in participants with drug-susceptible disease, the eventual goal of the clinical development programme is to develop quabodepistat as part of an effective, shorter-duration, less-toxic, and well tolerated regimen for patients with drug-resistant tuberculosis, which has the highest unmet clinical need. We acknowledge that regimens might behave differently in individuals with drug-susceptible tuberculosis and individuals with drug-resistant tuberculosis, even when the individuals are fully susceptible to the regimen (ie, there is no microbiological difference). The phase 3 trial in participants with drug-resistant tuberculosis was therefore carefully designed to monitor treatment responses.

A limitation of this proof-of-concept trial was the use of sputum culture conversion as the primary endpoint, which can limit correlation with long-term outcomes—such as relapse and cure—and therefore not fully capture clinical efficacy. This study was not powered to compare long-term outcomes. Despite being conducted in participants with drug-susceptible tuberculosis, because this proof-of-concept study showed some promising results, a phase 3 study has begun that will test the concept of quabodepistat as the backbone of a treatment-shortening regimen for drug-resistant tuberculosis. Additional limitations include not assessing the single-drug contribution of quabodepistat to the treatment effect of the combination therapy with delamanid and bedaquiline. Such single-drug contribution trials are generally not conducted beyond the 14-day early bactericidal activity setting, and information regarding contribution might need to be obtained from non-clinical studies (as recognised by the regulators and used for approval of the bedaquiline–pretomanid–linezolid regimen^27^), which are ongoing with quabodepistat. The small sample size of the trial, especially in the RHEZ group, did not provide adequate power to conduct non-inferiority testing on the post-treatment follow-up exploratory endpoints (ie, relapse and favourable outcomes). Moreover, the relatively low number of participants in the RHEZ group could limit the precision of comparisons and the generalisability of the findings, and potentially weaken performance against external benchmarks. Similarly, the small number of participants with bilateral cavitation constrains the reliability and interpretability of results within that subgroup. Because this proof-of-concept trial was conducted exclusively in South Africa, in a predominately male population (65%), with a small sample size and restrictive inclusion criteria, the findings—including the generally favourable safety profile of the quabodepistat-containing regimen—might not be fully generalisable to larger or more diverse populations. Similarly, excluding older patients (aged >65 years) could underestimate the occurrence of adverse events in a wider population. For analysis of the effects on hepatic enzymes and QTcF, intensive monitoring and a small sample size (especially in the control group) could have created bias surrounding safety. Also, as an open-label trial, the subjective endpoints that form part of the unfavourable outcome criteria might have biased decisions regarding unsuccessful treatment against the experimental group. Finally, paediatric populations were excluded from participation and, although they were not ineligible, the trial did not enrol any participants who were living with HIV or who were pregnant and/or lactating. Many of the limitations of the proof-of-concept study will be expanded on in the planned phase 3 trial.

Further studies are needed to assess how quabodepistat-containing treatment regimens fit into the landscape of future antituberculosis treatments in patients with drug-resistant tuberculosis.^28^, ^29^ This trial established a promising end-of-treatment non-inferiority signal for the primary endpoint of the proportion of participants with sputum culture conversion with a 4-month, three-drug-backbone, quabodepistat-containing regimen. Using information gathered in this proof-of-concept trial, a phase 3 clinical trial (NCT07209761) with adequate power and broader populations will evaluate non-inferiority in the occurrence of long-term, clinically meaningful endpoints.

### Contributors

### Equitable Partnership Declaration

### Data sharing

The protocol and statistical analysis plan for this study can be found in appendix 1. Data will be available after marketing approval in global markets, or beginning 1–3 years after article publication. There is no end date to the availability of the data. To submit inquiries related to Otsuka clinical research, or to request access to individual participant data (IPD) associated with any Otsuka clinical trial, please visit https://clinical-trials.otsuka.com/. For all approved IPD access requests, Otsuka will share anonymised IPD on a remotely accessible data-sharing platform.

## Declaration of interests

RD, EV, TM, WB, TN, NZ-G, AO, MR, and JAB are investigators of this study and their institutions received funding support from Otsuka Pharmaceutical Development & Commercialization to conduct the study. AO is also an executive member and tuberculosis clinical research lead at Setshaba Research Center. MR is a member of an advisory board for Otsuka Pharmaceutical Development & Commercialization. YL, RX, WL, and JH are employees of Otsuka Pharmaceutical Development & Commercialization (USA). ST is an employee of Otsuka Novel Products (Germany). AHD declares no competing interests.

## Supplementary Materials

Supplementary appendix 1

Equitable Partnership Declaration
