# Supplementary material for: Efficacy and safety of a 4-month quabodepistat, delamanid, and bedaquiline regimen for drug-susceptible pulmonary tuberculosis: a multicentre, open-label, randomised, proof-of-concept, non-inferiority, phase 2b/c trial
Source: Lancet Infect Dis. 2026 Oct;26(10):1010–21. doi: 10.1016/S1473-3099(26)00143-X (PMC13601027; doi:10.1016/S1473-3099(26)00143-X)
Supplement: Supplementary file 1 [file mmc1.pdf]

THE LANCET
Infectious Diseases

Supplementary appendix 1

This appendix formed part of the original submission and has been peer reviewed. We post it as supplied by the authors.

Supplement to: Dawson R, Diacon AH, Variava E, et al. Efficacy and safety of a 4-month quabodepistat, delamanid, and bedaquiline regimen for drug-susceptible pulmonary tuberculosis: a multicentre, open-label, randomised, proof-of-concept, non-inferiority, phase 2b/c trial. *Lancet Infect Dis* 2026; published online May 29. https://doi.org/10.1016/S1473-3099(26)00143-X.

Supplemental Appendix

Table of contents

Supplemental Table 1. Full Selection Criteria Page 1

Supplemental Table 2. Selected Trial Objectives and Endpoints Page 3

Supplemental Table 3. Efficacy Outcomes by Bilateral Cavitation Status[a] Page 5

Supplemental Table 4. Proportion of Participants With Sputum Culture Conversion by Study Week[a,b] Page 6

Supplemental Table 5. Participants with Post-treatment Positive Cultures, Relapses, and Reinfections Page 7

Supplemental Table 6. Treatment Emergent Adverse Events by System Organ Class and Preferred Term (≥5% in Any Treatment Arm)[a] Page 8

Supplemental Table 7. Incidence of Serious Treatment-Emergent Adverse Events by Preferred Term[a] Page 11

Supplemental Table 8. Incidence of Grade ≥ 3 Treatment-emergent Adverse Events by DAIDS Toxicity Grade by System Organ Class and Preferred Term (Safety Analysis Set)[a] Page 12

Supplemental Table 9. QTcF Analysis[a] Page 13

Supplemental Figure 1. Study Design Page 14

Supplemental Figure 2. Time to Sputum Culture Conversion by End of Treatment (Modified Intent-to-Treat Population) Page 15

Supplemental Figure 3. Serum Alanine Aminotransferase (A) and Aspartate Transaminase (B) Levels[a] Page 16

Supplemental Figure 4. QT Interval Analysis[a] Page 17

Supplemental Figure 5. Renal Function[a] Page 18

List of IECs/IRBs Page 19

Trial Protocol Page 20

Trial Protocol Amendment Page 96

Statistical Analysis Plan Page 181

Tables

Supplemental Table 1. Full Selection Criteria

Inclusion Criteria
Participants were required to meet the following inclusion criteria when assessed:

1. Able to provide written, informed consent prior to initiation of any trial-related procedures or treatments, and able, in the opinion of the investigator, to comply with all the requirements of the trial.
2. Male or female participants between 18 and 65 years of age (inclusive) at the screening visit.
3. Body weight ≥35·0 kg at the screening visit.
4. Newly diagnosed, rifampicin and isoniazid susceptible (on the screening sample) pulmonary TB.
5. Able to spontaneously produce sputum.
6. Females of childbearing potential must agree to use 2 different approved methods of birth control or remain abstinent throughout the participation in the trial and for 12 weeks after the last dose of investigational medicinal product(s) or dose of RHEZ.
7. Male participants must agree to use 2 different approved methods of birth control or remain abstinent throughout the participation in the trial and for 12 weeks after the last dose of investigational medicinal product (s) or RHEZ.

Exclusion Criteria
Participants were excluded if they met any of the following exclusion criteria when assessed:

1. Participants are known or suspected of having resistance to rifampicin, isoniazid, ethambutol, pyrazinamide, DLM, or BDQ either confirmed by the laboratory, or based on epidemiological history, at screening.
2. Evidence of clinically significant metabolic (for example, ongoing or current hypokalemia [ie, potassium <3.5 mEq/dL at screening]), gastrointestinal, neurological, psychiatric, endocrine or liver (eg, hepatitis B and C) disease; malignancy; or other abnormalities (other than the indication being studied).
3. History of, or current, clinically relevant cardiovascular disorder such as heart failure, coronary heart disease, uncontrolled hypertension, arrhythmia or symptom strongly suggestive of such a problem (eg, syncope or palpitations), tachyarrhythmia or status after myocardial infarction.
4. Known bleeding disorders or family history of bleeding disorders.
5. Any diseases or conditions in which the use of DLM, BDQ, QBS, rifampicin, isoniazid, pyrazinamide, or ethambutol is contraindicated.
6. Any prior treatment for *M. tuberculosis* within the past 2 years.
7. Any treatment with a drug active against *M. tuberculosis* (eg, quinolones) within the 3 months prior to screening.
8. Clinical evidence of severe extrapulmonary TB (eg, miliary TB, abdominal TB, urogenital TB, osteoarthritic TB, TB meningitis).
9. Evidence of pulmonary silicosis, lung fibrosis, or other lung condition considered as severe by the investigator (other than TB). In particular, any underlying condition that could interfere with the assessment of x-ray images, sputum collection, or interpretation of sputum findings, or otherwise compromise the participant's participation in the trial.
10. Any renal impairment characterized by creatinine clearance/eGFR of <60 mL/min/1·73 m^2, or hepatic impairment characterized by alanine transaminase or aspartate transaminase >2·0 × upper limit of normal of the clinical laboratory reference range or bilirubin >2·0 × upper limit of normal of the clinical laboratory reference range, at screening.
11. Screening glucose (nonfasting) ≥200 mg/dL or HbA1c ≥6·5%.
12. QTcF >450 msec in male participants (>470 msec in female participants), atrioventricular block II or III, bi-fasicular block, at screening or current or history of clinically significant ventricular arrhythmias. Other ECG abnormalities, if considered clinically significant by the investigator.
13. Participants receiving any of the prohibited medications (selected cytochrome P450 3A4 inhibitors and P450 3A4 inducers) within the specified periods or who would be likely to require prohibited concomitant therapy during the trial. See study protocol for tables listing prohibited medications.
14. Female participants who are breast-feeding or who have a positive pregnancy test result prior to receiving the first dose of investigational medicinal product(s) or RHEZ on Day 1.
15. Current history of significant drug and/or alcohol abuse that is likely to result in poor adherence to trial requirements or that would pose a risk to the participant's well-being during the course of the trial.
16. History of current hepatitis or carriers of HBsAg and/or anti HCV.
17. Participants who test positive for cocaine or other drugs of abuse (excluding known prescription stimulants and other prescribed medications and marijuana) at screening are excluded. Detectable levels of alcohol, marijuana, barbiturates, or opiates in the drug screen are not exclusionary if, in the investigator's documented opinion, the participant does not meet Diagnostic and Statistical Manual of Mental Disorders, Fifth Edition criteria for moderate to severe substance use disorder and the positive test does not signal a clinical condition that would impact the safety of the participant or interpretation of the trial results, and participation is agreed to by the medical monitor prior to treatment.
18. History of having taken an investigational drug within 30 days preceding trial entry.
19. A history of difficulty in donating blood.
20. Donation of blood or plasma within 30 days prior to dosing.
21. History of serious mental disorders that, in the opinion of the investigator, would exclude the participant from participating in this trial.
22. Any known prior exposure to QBS, DLM, or BDQ.
23. Participants with significant medical comorbidities that in the opinion of the investigator should not participate in the trial.
24. Participants with Karnofsky score <60 will be excluded from the trial.
25. Participants testing positive for active SARS-CoV-2 infection at screening.
26. Participants with HIV co-infection not on a stable anti-retroviral regimen consisting of tenofovir, emtricitabine/lamivudine, dolutegravir (ie, >3 months), or who have a detectable viral load, or who have a CD4 count <350 cells/mm^3 were excluded from the trial.

BDQ=bedaquiline; DLM=delamanid; ECG=electrocardiogram; eGFR=estimated glomerular filtration rate; HbA1c=glycosylated hemoglobin; HBsAg=hepatitis B surface antigen; HCV=hepatitis C virus; HIV=human

immunodeficiency virus; QBS=quabodepistat; QTcF=QT interval corrected by Fridericia's method; RHEZ=rifampicin, isoniazid, ethambutol, and pyrazinamide; SARS-CoV-2=severe acute respiratory syndrome coronavirus 2; TB=tuberculosis.

Supplemental Table 2. Selected Trial Objectives and Endpoints

Objectives	Endpoints
Primary	
• To assess the safety and efficacy of QBS combined with DLM and BDQ in participants with DS-TB administered for 4 months compared to RHEZ administered for 6 months	**Safety:**<br>• AEs, clinical laboratory tests, vital signs, physical examinations, ECGs<br>• The proportion of participants with a grade 3 or higher AE<br>• The rate of all-cause treatment discontinuation<br>**Efficacy:**<br>• The proportion of participants achieving SCC in MGIT by the end of the treatment period
Secondary	
• To evaluate efficacy in sputum MGIT culture for QBS combined with DLM and BDQ administered for 4 months as compared to RHEZ<br>• To evaluate the development of resistance to anti-TB drugs in all treatment arms<br>• To inform dose selection of QBS combined with DLM and BDQ	**Efficacy:**<br>• The proportion of participants who achieve SCC in MGIT by 8 weeks of treatment<br>• Proportion of participants who develop drug resistance<br>• Time to SCC of QBS, DLM, and BDQ, as well as RHEZ
Exploratory	
• To evaluate the efficacy of experimental arms in comparison to RHEZ through the end of the post-treatment follow-up period	**Efficacy:**<br>• The proportion of participants with favorable outcome at 12 months post-randomization<br>• The proportion of participants with relapse at 12 months post-randomization

AE=adverse event; BDQ=bedaquiline; DLM=delamanid; DS=drug-susceptible; ECG=electrocardiogram; MGIT=Mycobacteria Growth Indicator Tube®; QBS=quabodepistat; RHEZ=rifampicin, isoniazid, ethambutol, and pyrazinamide; SCC=sputum culture conversion; TB=tuberculosis.

Supplemental Table 3. Efficacy Outcomes by Bilateral Cavitation Status[a]

	**DBQ10 (n=20)**		**DBQ30 (n=42)**		**DBQ90 (n=38)**		**Pooled DBQ Arms (n=100)**		**RHEZ (n=21)**	
Bilateral cavitation status	With (n = 2)	Without (n = 18)	With (n = 6)	Without (n = 36)	With (n = 5)	Without (n = 33)	With (n = 13)	Without (n = 87)	With (n = 4)	Without (n = 17)
Participants with SCC at end of treatment[b], n (%) [95% CI][c]	1 (50) [1.2–98.7]	18 (100) [81·5–100·0]	6 (100) [54·1–100·0]	32 (89) [73·9–96·9]	5 (100) [47.8–100]	31 (94) [79·8–99·3]	12 (92) [64·0–99·8]	81 (93) [85·6–97·4]	4 (100) [39.7–100]	17 (100) [80·5–100·0]
Participants with SCC at 8 weeks[c], n (%) [95% CI][c]	0 (0) [0–84.1]	8 (44) [21·5–69·2]	3 (50) [11·8–88·2]	21 (58) [40·8–74·5][d]	5 (100) [47.8–100]	14 (42) [25·5–60·8]	8 (62) [31·6–86·1]	43 (49.4) [38·5–60·4][e]	1 (25) [0.6–80.5]	10 (59) [32·9–81·6]

[a]The mITT analysis set included all randomized participants who took at least one dose of investigational medicinal product (ie, delamanid, bedaquiline, or quabodepistat) or RHEZ, had at least one post-baseline culture result, and demonstrated positive culture results by 1 week and susceptibility to rifampicin and isoniazid (from the screening specimen). [b]SCC occurred when a participant had the first of two visits of at least 1 week apart with sputum cultures negative and without a positive sputum culture result in between. [c]95% CIs are Clopper Pearson CI. [d]n=35; [e]n=86.

CI=confidence interval; DBQ=delamanid + bedaquiline + quabodepistat; mITT=modified intent-to-treat; RHEZ=rifampicin, isoniazid, ethambutol, and pyrazinamide; SCC=sputum culture conversion.

Supplemental Table 4. Proportion of Participants With Sputum Culture Conversion by Study Week[a,b]

Participants with SCC, n (%)	**DBQ10 (n=20)**	**DBQ30 (n=42)**	**DBQ90 (n=38)**	**RHEZ (n=21)**
Week 4	2 (10)	2 (5)	3 (8)	1 (5)
Week 8	8 (40)	24 (57)	19 (50)	11 (52)
Week 10	13 (65)	33 (79)	26 (68)	17 (81)
Week 12	17 (85)	35 (83)	33 (87)	19 (90)
Week 13	20 (100)	37 (88)	36 (95)	20 (95)
Week 15	19 (95)	37 (88)	35 (92)	20 (95)
Week 17	20 (100)	39 (93)	37 (97)	21 (100)

[a]The mITT analysis set included all randomized participants who took at least one dose of investigational medicinal product (ie, delamanid, bedaquiline, or quabodepistat) or RHEZ, had at least one post-baseline culture result, and demonstrated positive culture results by 1 week and susceptibility to rifampicin and isoniazid (from the screening specimen). [b]SCC occurred when a participant had the first of two visits of at least 1 week apart with sputum cultures negative and without a positive sputum culture result in between.

DBQ=delamanid + bedaquiline + quabodepistat; mITT=modified intent-to-treat; RHEZ=rifampicin, isoniazid, ethambutol, and pyrazinamide; SCC=sputum culture conversion.

Supplemental Table 5. Participants with Post-treatment Positive Cultures, Relapses, And Reinfections

	DBQ10 (n=20)	DBQ30 (n=42)	DBQ90 (n=38)	Pooled DBQ Arms (n=100)	RHEZ (n=21)
Post-treatment positive cultures, n (%)	5 (25)	6 (14)	7 (18)	18 (18)	2 (10)
Relapses	2	5	5	12	1
Reinfections	3	1	2	6	1

DBQ=delamanid + bedaquiline + quabodepistat; RHEZ=rifampicin, isoniazid, ethambutol, and pyrazinamide.

Supplemental Table 6. Treatment Emergent Adverse Events by System Organ Class and Preferred Term (≥5% in Any Treatment Arm)[a]

n (%)	DBQ10 (n=20)	DBQ30 (n=42)	DBQ90 (n=38)	RHEZ (n=21)	Overall (N=121)
**Cardiac disorders**					
Atrioventricular block first degree	0	2 (5)	1 (3)	0	3 (2)
**Eye disorders**					
Conjunctival hemorrhage	1 (5)	0	0	0	1 (1)
Conjunctivitis allergic	0	1 (2)	2 (5)	0	3 (2)
Eye irritation	1 (5)	0	0	0	1 (1)
Eye pain	0	2 (5)	0	0	2 (2)
Visual acuity reduced	1 (5)	0	1 (3)	0	2 (2)
**Gastrointestinal disorders**					
Abdominal pain	2 (10)	1 (2)	1 (3)	1 (5)	5 (4)
Abdominal tenderness	1 (5)	0	0	0	1 (1)
Constipation	1 (5)	3 (7)	0	0	4 (3)
Dental caries	0	0	2 (5)	0	2 (2)
Diarrhea	2 (10)	5 (12)	2 (5)	3 (14)	12 (10)
Dyspepsia	1 (5)	3 (7)	2 (5)	0	6 (5)
Gastritis	0	0	2 (5)	0	2 (2)
Gastroesophageal reflux disease	0	2 (5)	0	0	2 (2)
Hemorrhoids	0	0	0	1 (5)	1 (1)
Nausea	0	0	3 (8)	3 (14)	6 (5)
Noninfective gingivitis	1 (5)	0	0	0	1 (1)
Stomatitis	1 (5)	0	0	0	1 (1)
Toothache	0	2 (5)	0	1 (5)	3 (2)
Vomiting	0	4 (10)	2 (5)	1 (5)	7 (6)
**General disorders and administration site conditions**					
Chest pain	0	4 (10)	1 (3)	1 (5)	6 (5)
Chills	1 (5)	0	0	0	1 (1)
Influenza like illness	0	4 (10)	2 (5)	0	6 (5)
Non-cardiac chest pain	0	0	1 (3)	1 (5)	2 (2)
Pain	1 (5)	2 (5)	0	0	3 (2)
Pyrexia	0	1 (2)	1 (3)	1 (5)	3 (2)
**Hepatobiliary disorders**					
Hepatitis alcoholic	1 (5)	0	0	0	1 (1)
**Immune system disorders**					
Food allergy	0	0	0	1 (5)	1 (1)
Hypersensitivity	1 (5)	0	1 (3)	1 (5)	3 (2)
Seasonal allergy	0	1 (2)	2 (5)	0	3 (2)
**Infections and infestations**					
Abscess limb	1 (5)	1 (2)	1 (3)	0	3 (2)
Appendicitis perforated	1 (5)	0	0	0	1 (1)
Body tinea	1 (5)	2 (5)	0	0	3 (2)
Cellulitis	1 (5)	0	0	0	1 (1)
Conjunctivitis	0	2 (5)	1 (3)	0	3 (2)
Gastroenteritis	1 (5)	3 (7)	2 (5)	0	6 (5)
HIV infection	1 (5)	0	0	0	1 (1)
Hemophilus infection	1 (5)	0	0	0	1 (1)
Influenza	0	2 (5)	0	1 (5)	3 (2)
Localized infection	1 (5)	0	0	0	1 (1)
Lower respiratory tract infection	2 (10)	0	2 (5)	3 (14)	7 (6)
Nasopharyngitis	1 (5)	1 (2)	0	1 (5)	3 (2)
Otitis externa	0	0	0	1 (5)	1 (1)
Pneumonia	0	1 (2)	1 (3)	1 (5)	3 (2)
Respiratory tract infection	0	0	2 (5)	0	2 (2)

Sexually transmitted disease	1 (5)	3 (7)	0	0	4 (3)
Subcutaneous abscess	1 (5)	0	2 (5)	0	3 (2)
Upper respiratory tract infection	3 (15)	13 (31)	12 (32)	8 (38)	36 (30)
Urinary tract infection	1 (5)	1 (2)	2 (5)	1 (5)	5 (4)
Vulvovaginal candidiasis	0	0	0	1 (5)	1 (1)
**Injury, poisoning, and procedural complications**					
Ligament sprain	0	1 (2)	0	1 (5)	2 (2)
Limb injury	0	2 (5)	1 (3)	0	3 (2)
Skin laceration	0	2 (5)	1 (3)	0	3 (2)
Soft tissue injury	0	1 (2)	0	2 (10)	3 (2)
Thermal burn	1 (5)	0	1 (3)	1 (5)	3 (2)
**Investigation**					
Alanine aminotransferase increased	0	3 (7)	1 (3)	1 (5)	5 (4)
Aspartate aminotransferase increased	0	1 (2)	1 (3)	2 (10)	4 (3)
Blood pressure increased	1 (5)	1 (2)	2 (5)	1 (5)	5 (4)
Electrocardiogram QT prolonged	2 (10)	2 (5)	0	0	4 (3)
Hemoglobin decreased	1 (5)	0	0	0	1 (1)
**Metabolism and nutrition disorders**					
Abnormal loss of weight	2 (10)	0	0	0	2 (2)
Decreased appetite	1 (5)	2 (5)	0	0	3 (2)
**Musculoskeletal and connective tissue disorders**					
Arthralgia	3 (15)	2 (5)	0	4 (19)	9 (7)
Back pain	2 (10)	5 (12)	2 (5)	0	9 (7)
Joint swelling	0	0	0	1 (5)	1 (1)
Muscle spasms	1 (5)	2 (5)	0	0	3 (2)
Myalgia	0	0	2 (5)	0	2 (2)
Neck pain	0	0	1 (3)	1 (5)	2 23)
Pain in extremity	0	2 (5)	1 (3)	1 (5)	4 (3)
Temporomandibular pain and dysfunction syndrome	1 (5)	0	0	0	1 (1)
**Nervous system disorders**					
Headache	3 (15)	8 (19)	7 (18)	6 (29)	24 (20)
Neuropathy peripheral	0	0	0	2 (10)	2 (2)
Paresthesia	0	1 (2)	0	1 (5)	2 (2)
**Psychiatric disorders**					
Insomnia	1 (5)	1 (2)	1 (3)	0	3 (2)
Stress	0	2 (5)	0	0	2 (2)
**Renal and urinary disorders**					
Dysuria	1 (5)	1 (2)	0	0	2 (2)
Urinary hesitation	1 (5)	0	0	0	1 (1)
**Reproductive system and breast disorders**					
Breast discharge	0	0	0	1 (5)	1 (1)
Dysmenorrhea	0	0	0	1 (5)	1 (1)
Erectile dysfunction	0	3 (7)	0	0	3 (2)
Heavy menstrual bleeding	0	0	1 (3)	1 (5)	2 (2)
Intermenstrual bleeding	1 (5)	0	0	0	1 (1)
Uterine cyst	0	0	0	1 (5)	1 (1)
**Respiratory, thoracic, and mediastinal disorders**					
Bronchiectasis	1 (5)	0	0	0	1 (1)
Cough	1 (5)	3 (7)	0	0	4 (3)
Hemoptysis	2 (10)	1 (2)	1 (3)	1 (5)	5 (4)
Pleuritic pain	1 (5)	0	0	0	1 (1)
**Skin and subcutaneous tissue disorders**					
Dermatitis allergic	2 (10)	0	0	0	2 (2)
Dermatitis contact	1 (5)	0	0	0	1 (1)
Drug eruption	0	0	1 (3)	1 (5)	2 (2)
Eczema weeping	1 (5)	0	0	0	1 (1)
Night sweats	1 (5)	1 (2)	1 (3)	0	3 (2)

Papule	0	0	0	1 (5)	1 (1)
Pruritus	2 (10)	1 (2)	0	0	3 (2)
Rash	0	2 (5)	1 (3)	1 (5)	4 (3)
Rash maculo-papular	0	0	0	1 (5)	1 (1)
Rash papular	0	1 (2)	0	1 (5)	2 (2)
**Vascular disorders**					
Hypertension	2 (10)	2 (5)	2 (5)	1 (5)	7 (6)

[a]The safety analysis set included all randomized participants who received at least one dose of investigational medicinal product (ie, delamanid, bedaquiline, or quabodepistat) or RHEZ.

DBQ=delamanid + bedaquiline + quabodepistat; HIV=human immunodeficiency virus; RHEZ=rifampicin, isoniazid, ethambutol, and pyrazinamide.

Supplemental Table 7. Incidence of Serious Treatment-Emergent Adverse Events by Preferred Term[a]

n (%)	**DBQ10 (n=20)**	**DBQ30 (n=42)**	**DBQ90 (n=38)**	**RHEZ (n=21)**	**Overall (N=121)**
**Number of participants with serious TEAE**	2 (10)	3 (7)	0	0	5 (4)
Appendicitis perforated	1 (5)	0	0	0	1 (1)
Localized infection	1 (5)	0	0	0	1 (1)
Pneumonia	0	1 (2)	0	0	1 (1)
Tuberculosis	0	1 (2)	0	0	1 (1)
Hyperkalemia	0	1 (2)	0	0	1 (1)
Myelopathy	0	1 (2)	0	0	1 (1)
Hemoptysis	0	1 (2)	0	0	1 (1)

[a]The safety analysis set included all randomized participants who received at least one dose of investigational medicinal product (ie, delamanid, bedaquiline, or quabodepistat) or RHEZ.

DBQ=delamanid + bedaquiline + quabodepistat; RHEZ=rifampicin, isoniazid, ethambutol, and pyrazinamide; TEAE=treatment-emergent adverse event.

Supplemental Table 8. Incidence of Grade ≥ 3 Treatment-emergent Adverse Events by DAIDS Toxicity Grade by System Organ Class and Preferred Term (Safety Analysis Set)[a]

System Organ Class Adverse Event (MedDRA Preferred Term), n (%)	DAIDS Grade	DBQ10 (n=20)	DBQ30 (n=42)	DBQ90 (n=38)	RHEZ (n=21)	Overall (N=121)
Total DAIDS Grade 3		4 (20)	4 (10)	4 (11)	1 (5)	13 (11)
Total DAIDS Grade 4		0	1 (2)	0	0	1 (1)
Total DAIDS Grade 5		0	1 (2)	0	0	1 (1)
Hepatobiliary disorders	Grade 3	1 (5)	0	0	0	1 (1)
Hepatitis alcoholic	Grade 3	1 (5)	0	0	0	1 (1)
Infections and infestations	Grade 3	1 (5)	0	0	0	1 (1)
	Grade 5	0	1 (2)	0	0	1 (1)
Appendicitis perforated	Grade 3	1 (5)	0	0	0	1 (1)
Pneumonia	Grade 3	0	1 (2)	0	0	1 (1)
Tuberculosis	Grade 5	0	1 (2)	0	0	1 (1)
Investigations	Grade 3	1 (5)	2 (5)	4 (11)	1 (5)	8 (7)
Alanine aminotransferase increased	Grade 3	0	1 (2)	0	1 (5)	2 (2)
Aspartate aminotransferase increased	Grade 3	0	1 (2)	1 (3)	1 (5)	3 (2)
Glomerular filtration rate decreased	Grade 3	0	1 (2)	1 (3)	0	2 (2)
Hemoglobin decreased	Grade 3	1 (5)	0	0	0	1 (1)
Hepatic enzyme increased	Grade 3	0	0	1 (3)	0	1 (1)
Neutrophil count decreased	Grade 3	0	0	1 (3)	0	1 (1)
Metabolism and nutrition disorders	Grade 3	1 (5)	0	0	0	1 (1)
	Grade 4	0	1 (2)	0	0	1 (1)
Abnormal loss of weight	Grade 3	1 (5)	0	0	0	1 (1)
Hyperkaliemia	Grade 4	0	1 (2)	0	0	1 (1)
Hypoglycemia	Grade 3	0	1 (2)	0	0	1 (1)
Musculoskeletal and connective tissue disorders	Grade 3	0	1 (2)	0	0	1 (1)
Muscular weakness	Grade 3	0	1 (2)	0	0	1 (1)
Respiratory, thoracic and mediastinal disorders	Grade 3	0	2 (5)	0	0	2 (2)
Dyspnea	Grade 3	0	1 (2)	0	0	1 (1)
Hemoptysis	Grade 3	0	1 (2)	0	0	1 (1)
Vascular disorders	Grade 3	1 (5)	0	0	0	1 (1)
Hypertension	Grade 3	1 (5)	0	0	0	1 (1)

[a]The safety analysis set included all randomized participants who received at least one dose of investigational medicinal product (i.e., delamanid, bedaquiline, or quabodepistat) or RHEZ. Participants are counted once per term, for the greatest grade of multiple occurrences of a specific MedDRA preferred term and system organ class.

DBQ=delamanid + bedaquiline + quabodepistat; MedDRA=Medical Dictionary for Regulatory Activities; RHEZ=rifampicin, isoniazid, ethambutol, and pyrazinamide; TEAE=treatment-emergent adverse event.

Supplemental Table 9. QTcF Analysis[a]

	DBQ10 (n=20)	DBQ30 (n=42)	DBQ90 (n=38)	RHEZ (n=21)	Overall (N=121)
**Maximum QTcF, no. (%)**					
450–479 msec	5 (25)	8 (19)	4 (11)	1 (5)	18 (15)
480–499 msec	0	0	0	0	0
≥500 msec	0	0	0	0	0
**QTcF increase from baseline, no. (%)**					
30–60 msec	13 (65)	28 (67)	27 (71)	11 (52)	79 (65)
>60 msec	0	5 (12)	2 (5)	1 (5)	8 (7)

[a]The safety analysis set included all randomized participants who received at least one dose of investigational medicinal product (ie, delamanid, bedaquiline, or quabodepistat) or RHEZ.

DBQ=delamanid + bedaquiline + quabodepistat; QTcF=QT interval corrected by Fridericia's method; RHEZ=rifampicin, isoniazid, ethambutol, and pyrazinamide.

Figures

Supplemental Figure 1. Study Design

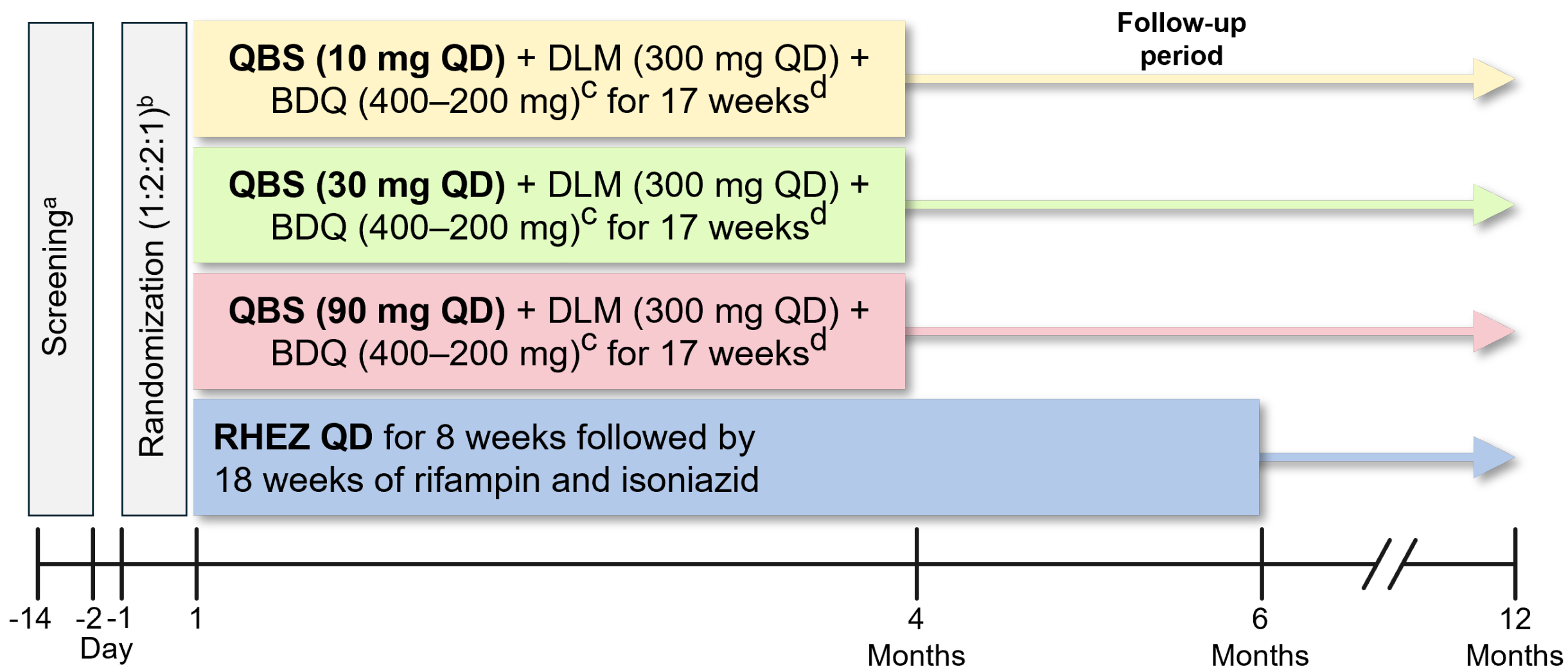

[a]Screening occurred for up to 14 days but was to be completed in as few days as possible. [b]Randomization on day −1 or pre-dose day 1. Baseline = day −1/day 1. [c]BDQ dose 400 mg QD for 2 weeks, then 200 mg three times per week. [d]Rescue treatment consisting of the standard of care treatment, ie, RHEZ, will be provided to participants in the QBS treatment arms in the following circumstances: 1) if a participant is sputum culture positive for *M. tuberculosis* at the end of treatment; 2) if a participant has a microbiologic relapse subsequent to the end of treatment; or, 3) if, in the opinion of the investigator, a participant is clinically deteriorating, based on any combination of signs, symptoms, or chest radiograph data. Participants in the RHEZ arm will be managed according to local guidelines if not responding to therapy.

BDQ=bedaquiline; DBQ, DLM+BDQ+QBS; DLM=delamanid; QBS=quabodepistat; QD=once daily; RHEZ=rifampicin, isoniazid, ethambutol, and pyrazinamide.

Supplemental Figure 2. Time to Sputum Culture Conversion by End of Treatment[a]

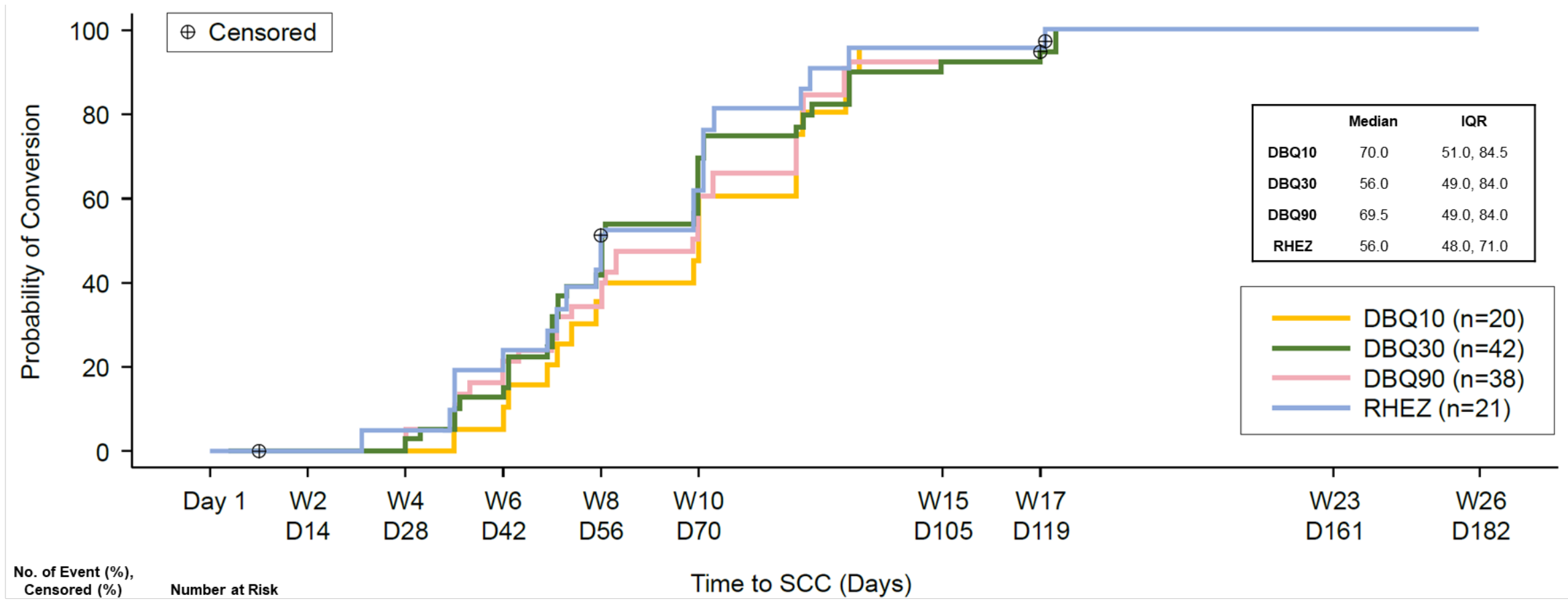

	No. of Event (%), Censored (%)	Number at Risk								
		Day 1	W2	W4	W6	W8	W10	W15	W17	W23
DBQ10	20 (100), 0 (0.0)	20	20	20	19	13	11	1	1	
DBQ30	39 (92.9), 3 (7.1)	42	41	41	36	24	17	4	3	
DBQ90	37 (97.4) 1 (2.6)	38	38	38	32	25	19	3	2	
RHEZ	21 (100), 0 (0.0)	21	21	20	17	12	8	1	1	0

[a]The mITT analysis set included all randomized participants who took at least one dose of investigational medicinal product (ie, delamanid, bedaquiline, or quabodepistat) or RHEZ, had at least one post-baseline culture result, and demonstrated positive culture results by 1 week and susceptibility to rifampicin and isoniazid (from the screening specimen). Time to SCC is one of the secondary endpoints and sample size was not powered for comparison on this endpoint; therefore, statistical comparison was not prespecified in the Statistical Analysis Plan. Only summary statistics for median and inter quartile range of time to SCC were prespecified in the Statistical Analysis Plan.

D=day; DBQ=delamanid + bedaquiline + quabodepistat; mITT=(Modified Intent-to-Treat Population); RHEZ=rifampicin, isoniazid, ethambutol, and pyrazinamide; SCC=sputum culture conversion; W=week.

Supplemental Figure 3. Serum Alanine Aminotransferase (A) and Aspartate Aminotransferase (B) Levels[a]

A

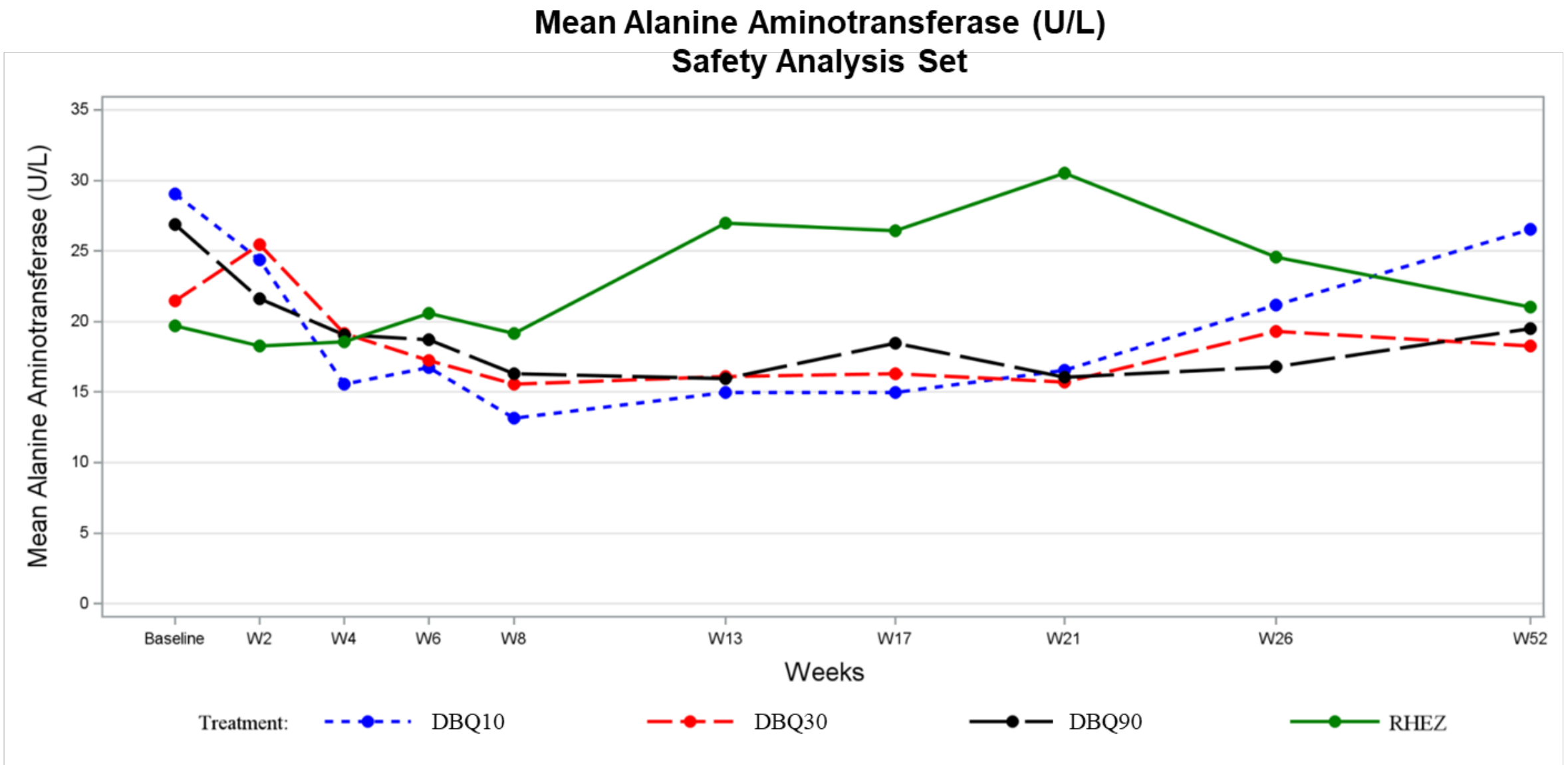

B

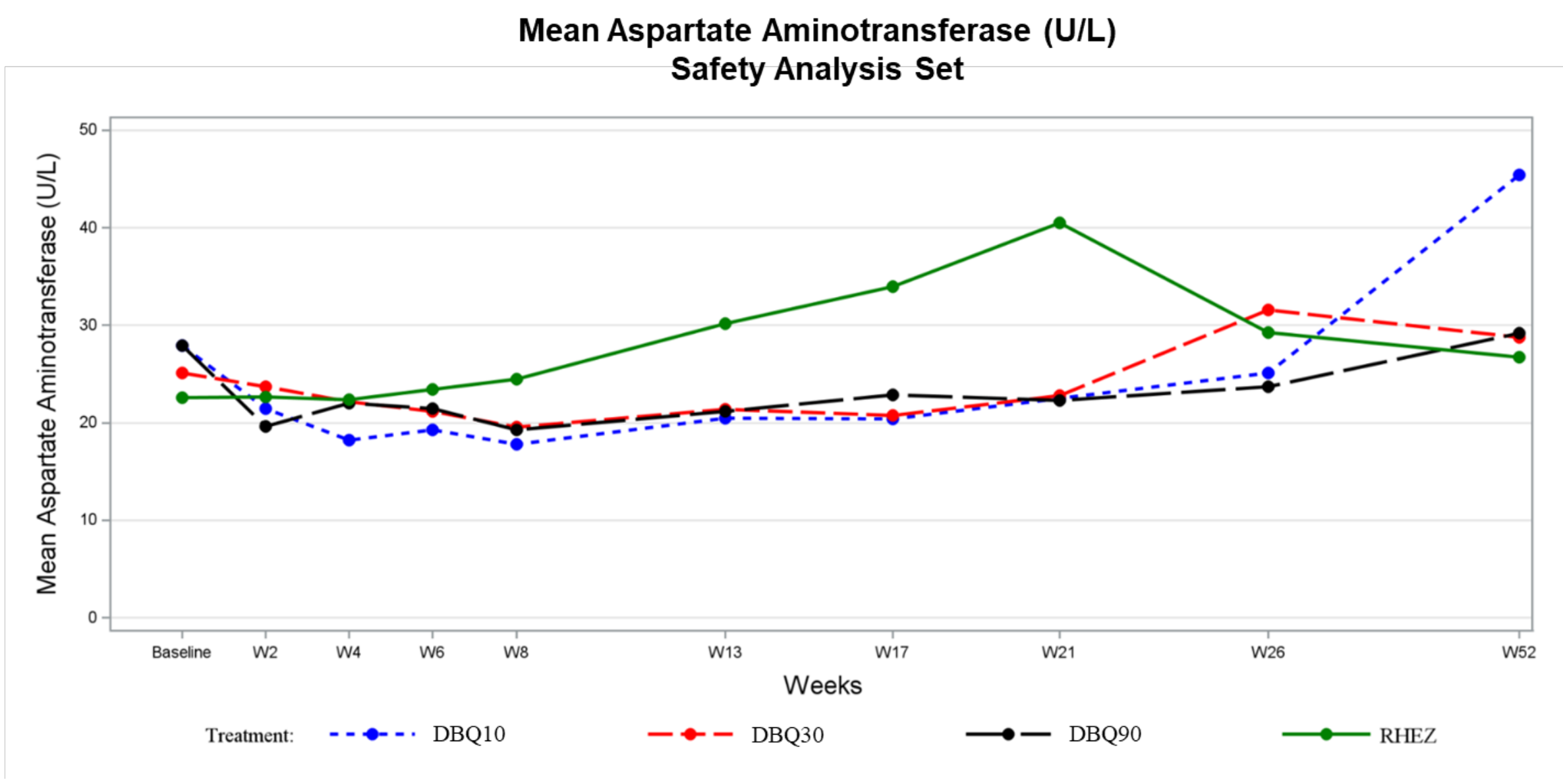

[a]The safety analysis set included all randomized participants who received at least one dose of investigational medicinal product (ie, delamanid, bedaquiline, or quabodepistat) or RHEZ. Mean values in participants with available data at the given visit.

DBQ=delamanid + bedaquiline + quabodepistat; RHEZ=rifampicin, isoniazid, ethambutol, and pyrazinamide; W=week.

Supplemental Figure 4. QT Interval Analysis[a]

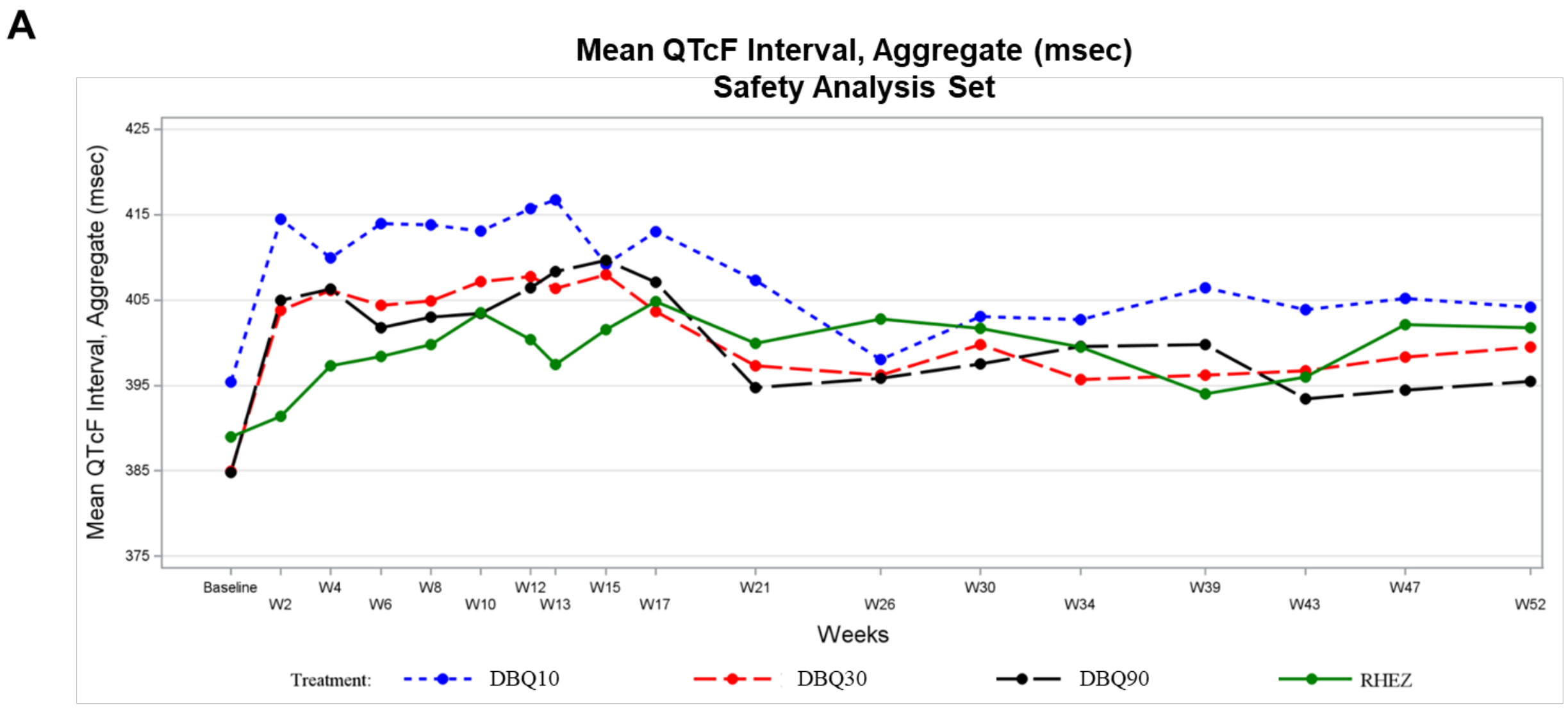

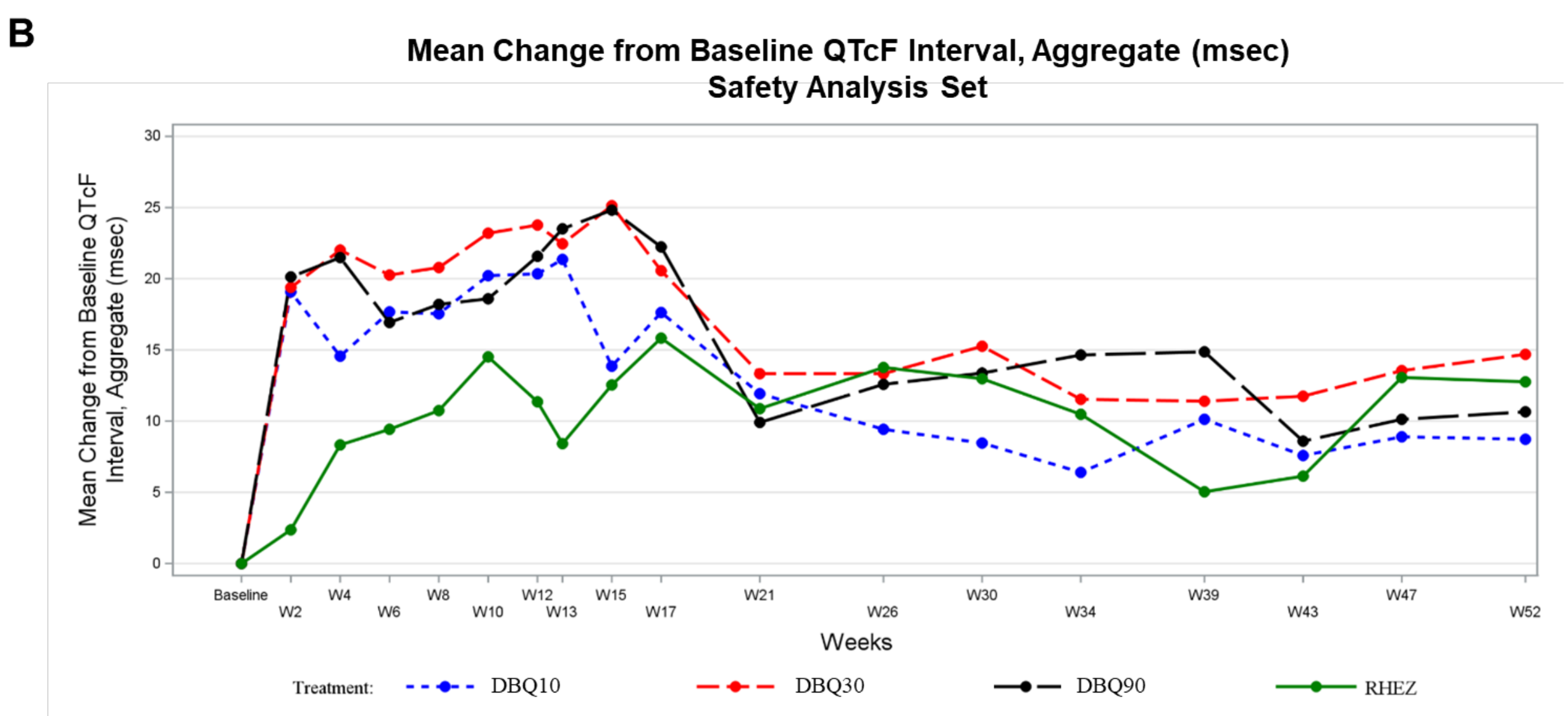

[a]The safety analysis set included all randomized participants who received at least one dose of investigational medicinal product (ie, delamanid, bedaquiline, or quabodepistat) or RHEZ. Mean values in participants with available data at the given visit.

DBQ=delamanid + bedaquiline + quabodepistat; QTcF=QT corrected for heart rate by Fridericia's cube root formula; RHEZ=rifampicin, isoniazid, ethambutol, and pyrazinamide; W=week.

Supplemental Figure 5. Renal Function[a]

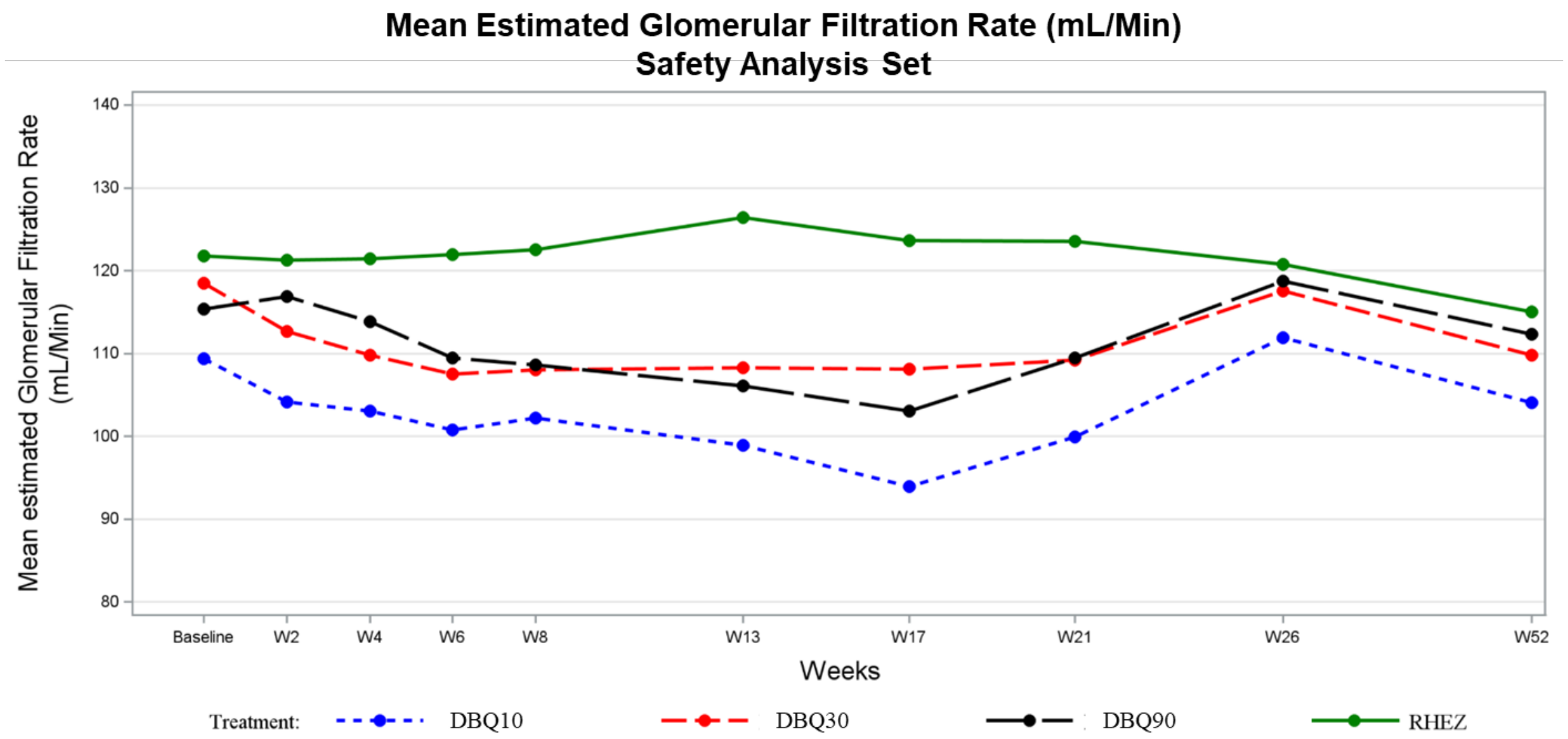

[a]The safety analysis set included all randomized participants who received at least one dose of investigational medicinal product (ie, delamanid, bedaquiline, or quabodepistat) or RHEZ. Mean values in participants with available data at the given visit.

DBQ=delamanid + bedaquiline + quabodepistat; RHEZ=rifampicin, isoniazid, ethambutol, and pyrazinamide; W=week.

Additional Information: List of IECs or IRBs

Site Number	Site Name	IEC/IRB Name	Approval Number
001	Setshaba Research Centre<br>2088 Block H Soshanguve<br>0152, South Africa	Pharma-Ethics Independent Research Ethics Committee	211024251
002	University of Cape Town Lung Institute (Pty) Ltd<br>George Street, Mowbray, 7700, Cape Town, South Africa	University of Cape Town Faculty of Health Sciences Human Research Ethics Committee	629/2021
003	TASK Applied Science, Brooklyn Chest Hospital Premises, Stanberry Road, Ysterplaat, 7405, Cape Town South Africa	Pharma-Ethics Independent Research Ethics Committee	211024251
004	Desmond Tutu Health Foundation - Masiphumelele Research Office Guinea Fowl Road, Sunnydale<br>Fish Hoek, Cape Town,<br>South Africa, 7975	University of Cape Town Faculty of Health Sciences Human Research Ethics Committee	630/2021
006	University of Witwatersrand<br>Clinical HIV Research Unit (CHRU)<br>Themba Lethu Clinic<br>Helen Joseph Hospital<br>Perth Road, Westdene, 2092 Johannesburg, South Africa	University of Witwatersrand Human Research Ethics Committee (Medical)	211004B
007	Klerksdorp/Tshepong Hospital Complex Benji Oliphant Road<br>Jouberton, Klerksdorp, 2569	University of Witwatersrand Human Research Ethics Committee (Medical)	211004B
008	The Aurum Institute: Tembisa Clinical Resesarch Centre cnr Rev RTJ Namane and Flint Mazibuko Drive<br>Hospital View Tembisa,<br>Gauteng South Africa 1632	University of Witwatersrand Human Research Ethics Committee (Medical)	211004B
009	The Aurum Institute: Rustenburg Clinical Research Centre Heritage Building<br>178 Beyers Naude Drive Rustenburg,<br>Northwest South Africa, 0029	Pharma-Ethics Independent Research Ethics Committee	211024251

IEC=Independent Ethics Committee; IRBs=Institutional Review Board.

Otsuka Pharmaceutical
Development & Commercialization, Inc.

Investigational Medicinal Product

OPC-167832

CLINICAL PROTOCOL

A Multicenter, Phase 2b/c, Open-label, Randomized, Dose-finding Trial to Evaluate the Safety and Efficacy of a 4-month Regimen of OPC-167832 in Combination with Delamanid and Bedaquiline in Subjects with Drug-susceptible Pulmonary Tuberculosis in Comparison with Standard Treatment

Protocol No. 323-201-00006
IND No. 129303

CONFIDENTIAL — PROPRIETARY INFORMATION

Clinical Development Phase:	Phase 2b/c
Sponsor:	Otsuka Pharmaceutical Development & Commercialization, Inc.
Immediately Reportable Event<br>[redacted]	Clindev Pty Ltd<br>[redacted]

Table of Contents

Table of Contents **2**

List of In-text Tables **7**

List of In-text Figures **8**

1 Protocol Summary **9**

1.1 Synopsis 9

1.2 Schema 16

1.3 Schedule of Assessments 18

2 Introduction **22**

2.1 Trial Rationale 22

2.2 Background 23

2.3 Known and Potential Risks and Benefits 24

2.3.1 OPC-167832 24

2.3.2 Delamanid 26

2.3.3 Bedaquiline 27

2.3.4 Rifampin, Isoniazid, Ethambutol, and Pyrazinamide 27

3 Objectives and Endpoints **27**

4 Trial Design **29**

4.1 Type/Design of Trial 29

4.2 Scientific Rationale for Trial Design 29

4.3 Dosing Rationale 30

4.4 End of Trial Definition 31

4.5 Definition of Completed Subjects 31

5 Trial Population **31**

5.1 Subject Selection and Numbering 31

5.2 Eligibility Criteria 32

5.2.1 Inclusion Criteria 32

5.2.2 Exclusion Criteria 32

5.3 Lifestyle Considerations 34

5.3.1 Meals and Dietary Restrictions 34

5.3.2 Caffeine, Alcohol, and Tobacco 34

5.4 Screen Failures 34

6 Trial Treatments 35

6.1 Trial Treatments Administered 35

6.1.1 Medical Devices 36

6.2 Management of Investigational Medicinal Product 36

6.2.1 Packaging and Labeling 36

6.2.2 Storage 37

6.2.3 Accountability 37

6.2.4 Returns and Destruction 37

6.2.5 Reporting of Product Quality Complaints 37

6.2.5.1 Eliciting and Reporting Product Quality Complaints 38

6.2.5.2 Information Required for Reporting Purposes 38

6.2.5.3 Return Process 38

6.2.5.4 Assessment/Evaluation 39

6.3 Measures to Minimize/Avoid Bias 39

6.4 Subject Compliance 39

6.5 Concomitant Medications or Therapies 39

6.5.1 Prohibited Medications or Therapies 39

6.5.2 Permitted Medications or Therapies 40

6.5.3 Rescue Medications 41

6.6 Intervention After the End of the Trial 41

7 Stopping Rules, Withdrawal Criteria, and Procedures 41

7.1 Entire Trial or Treatment 41

7.2 Individual Site 41

7.3 Individual Subject Discontinuation 41

7.3.1 Treatment Interruption 42

7.3.2 Treatment Discontinuation 42

7.3.3 Documenting Reasons for Treatment Interruption or Discontinuation 42

7.3.4 Withdrawal of Consent 43

7.3.5 Procedures to Encourage Continued Trial Participation 44

7.4 Definition of Subjects Lost to Follow-up 44

8 Trial Procedures 45

8.1 Efficacy Assessments 45
8.1.1 Sputum Sampling and Microbiology Laboratory Assessments....45
8.1.1.1 Sputum for Eligibility Assessments 45
8.1.1.2 Sputum for Efficacy Assessments....45
8.1.2 Signs and Symptoms of Tuberculosis....46
8.2 Pharmacokinetic Assessments....47
8.2.1 Pharmacokinetic Blood/Serum/Plasma Samples 47
8.3 Pharmacodynamic Assessments....48
8.3.1 Pharmacodynamic Laboratory Tests 48
8.4 Pharmacogenomic Assessments....48
[redacted]
8.7 Safety Assessments 49
8.7.1 Clinical Laboratory Assessments 49
8.7.2 Physical Examination 49
8.7.3 Vital Signs 49
8.7.4 Electrocardiogram....50
8.7.5 Suicidality Monitoring....50
8.7.6 Other Safety Variables....50
8.8 Adverse Events....50
8.8.1 Definitions 50
8.8.2 Eliciting and Reporting Adverse Events....52
8.8.3 Immediately Reportable Events....53
8.8.4 Adverse Events of Special Interest 53
8.8.5 Potential Serious Hepatotoxicity 53
8.8.6 Procedure for Breaking the Blind 54
8.8.7 Follow-up of Adverse Events 54
8.8.7.1 Follow-up of Nonserious Adverse Events 54
8.8.7.2 Follow-up of Immediately Reportable Events 54
8.8.7.3 Follow-up and Reporting of Immediately Reportable Events Occurring After Last Scheduled Contact 55

8.9 Treatment of Overdose 55

8.10 Subject Assessment Recording 55

8.11 Other Assessments 55

9 Statistical Considerations 55

9.1 Sample Size 55

9.2 Datasets for Analysis 56

9.3 Handling of Missing Data for Primary and Secondary Endpoint Analysis 56

9.4 Statistical Analyses 56

9.4.1 Efficacy Analyses 56

9.4.1.1 Primary Efficacy Endpoint Analysis 56

9.4.1.2 Secondary Efficacy Endpoint Analysis 57

9.4.1.3 Control of Experiment-wise Type 1 Error 57

9.4.1.4 Other Efficacy Endpoint Analysis 57

9.4.2 Safety Analysis 57

9.4.2.1 Adverse Events 58

9.4.2.2 Clinical Laboratory Data 58

9.4.2.3 Physical Examination and Vital Signs Data 58

9.4.2.4 Electrocardiogram Data 58

9.4.3 Other Analyses 59

9.4.3.1 Analysis of Demographic and Baseline Characteristics 59

9.4.3.2 Pharmacokinetic Analysis 59

9.4.3.3 Pharmacodynamic Analysis 59

9.4.3.4 Exposure-Response Analysis 59

9.4.3.5 Pharmacogenomic Analysis 59

[redacted]

9.5 Interim Analysis and Adaptive Design 60

9.5.1 Data Monitoring Committee 60

10 Supporting Documentation and Operational Considerations 61

10.1 Appendix 1: Regulatory, Ethical, and Trial Oversight Considerations 61

10.1.1 Ethics and Responsibility 61

10.1.2 Informed Consent 61

10.1.3 Confidentiality 62

10.1.4 Quality Control and Quality Assurance 62
10.1.4.1 Monitoring 62
10.1.4.2 Auditing 63
10.1.5 Protocol Deviations 63
10.1.6 Records Management 63
10.1.6.1 Source Documents 63
10.1.6.2 Data Collection 64
10.1.6.3 File Management at the Trial Site 64
10.1.6.4 Records Retention at the Trial Site 65
10.1.6.5 Publication Authorship Requirements 65
10.2 Appendix 2: Clinical Laboratory Tests 67
10.3 Appendix 3: Contraceptive Guidance and Collection of Pregnancy Information 68
10.4 Appendix 4: List of Abbreviations 70
10.5 Appendix 5: Schedule of Sputum Collections for Each Treatment Arm 72
10.6 Appendix 6: Protocol Amendments 73
10.6.1 Protocol Amendment(s)/Administrative Change(s) 74
11 References 75

List of In-text Tables

Table 1.3-1 Schedule of Assessments - All Subjects 18
Table 3-1 Trial Objectives and Endpoints 27
Table 6.5.1-1 List of Selected Cytochrome P450 3A4 Inhibitors Prohibited During the Trial 40
Table 6.5.1-2 List of Selected Cytochrome P450 3A4 Inducers Prohibited During the Trial 40

List of In-text Figures

Figure 1.2-1 Trial Design Schematic..16

1 Protocol Summary

1.1 Synopsis

Name of Sponsor: Otsuka Pharmaceutical Development & Commercialization, Inc.

Name of Investigational Medicinal Product: OPC-167832, bedaquiline (BDQ), and delamanid (DLM)

Protocol No.: 323-201-00006

IND No.: 129303

Protocol Title: A Multicenter, Phase 2b/c, Open-label, Randomized, Dose-finding Trial to Evaluate the Safety and Efficacy of a 4-month Regimen of OPC-167832 in Combination with Delamanid and Bedaquiline in Subjects with Drug-susceptible Pulmonary Tuberculosis in Comparison with Standard Treatment

Clinical Phase: Phase 2b/c, Therapeutic use

Treatment/Indication: Drug-susceptible pulmonary tuberculosis (DS-TB)

Objectives and Endpoints:

Objectives	Endpoints
Primary:<br>• To assess the safety and efficacy of OPC-167832 combined with DLM and BDQ in subjects with DS-TB administered for 4 months compared to rifampin, isoniazid, ethambutol, pyrazinamide (RHEZ) administered for 6 months	Safety:<br>• Adverse events (AEs), clinical laboratory tests, vital signs, physical examinations, electrocardiograms (ECGs)<br>• The proportion of subjects with a grade 3 or higher AE<br>• The rate of all-cause treatment discontinuation<br><br>Efficacy:<br>• The proportion of subjects achieving sputum culture conversion ([SCC] in Mycobacteria Growth Indicator Tube® [MGIT]) by the end of the treatment period

Objectives	Endpoints
Secondary:<br>• To evaluate efficacy in sputum MGIT culture and lipoarabinomannan (LAM) for OPC-167832 combined with DLM and BDQ administered for 4 months as compared to RHEZ<br>• To evaluate the development of resistance to anti-tuberculosis (TB) drugs in all treatment arms<br>• To compare time to SCC of OPC-167832, DLM, and BDQ to RHEZ<br><br>Pharmacokinetics (PK):<br>• To evaluate the PK of OPC-167832 combined with DLM and BDQ in subjects with DS-TB	Efficacy:<br>• The proportion of subjects who achieve SCC in MGIT by 8 weeks of treatment<br>• Time to detection of MGIT cultures<br>• The proportion of subjects who convert sputum LAM to negative by 8 weeks of treatment and by end of treatment<br>• Proportion of subjects who develop drug resistance<br>• Time to SCC<br><br>PK:<br>• The concentrations of OPC-167832, DLM and its metabolite DM-6705, and BDQ and its metabolite M2
[redacted]	[redacted]

Trial Design:

Trial 323-201-00006 is a multicenter, phase 2b/c, open-label, randomized, dose-finding trial in subjects with DS-TB. The microbiology laboratory will be blinded to treatment assignments.

Following a screening period of up to 14 days, eligible subjects will be randomized on Day −1 or predose Day 1 in a ratio of 1:2:2:1 to one of the following treatment arms:

- Arm 1: DLM (300 mg once daily [QD]) + BDQ (400 mg QD x 2 weeks, then 200 mg thrice-weekly [TIW]) + OPC-167832 (10 mg QD) for 17 weeks
- Arm 2: DLM (300 mg QD) + BDQ (400 mg QD x 2 weeks, then 200 mg TIW) + OPC-167832 (30 mg QD) for 17 weeks
- Arm 3: DLM (300 mg once daily [QD]) + BDQ (400 mg QD x 2 weeks, then 200 mg TIW) + OPC-167832 (90 mg QD) for 17 weeks
- Arm 4: RHEZ for 8 weeks followed by 18 weeks of rifampin and isoniazid (for a total of 26 weeks)

After the end of the treatment period, subjects will be followed until 12 months postrandomization.

Trial Population:

The trial population will consist of subjects who are ≥ 18 years to ≤ 65 years at screening with body weight of ≥ 35.0 kg and who have newly diagnosed pulmonary DS-TB. Approximately 120 subjects will be enrolled at sites in South Africa.

Key Inclusion/Exclusion Criteria:

Key inclusion criteria are noted above, under Trial Population.

Key exclusion criteria are as follows:

- Subjects are known or suspected of having resistance to rifampin, isoniazid, ethambutol, pyrazinamide, DLM, or BDQ either confirmed by the laboratory, or based on epidemiological history, at screening.
- Evidence of clinically significant metabolic (including ongoing or current hypokalemia [ie, potassium < 3.5 mEq/dL at screening]), gastrointestinal, neurological, psychiatric, endocrine or liver (eg, hepatitis B and C) disease; malignancy; or other abnormalities (other than the indication being studied).
- History of, or current, clinically relevant cardiovascular disorder such as heart failure, coronary heart disease, uncontrolled hypertension, arrhythmia or symptom strongly suggestive of such a problem (for example, syncope or palpitations), tachyarrhythmia or status after myocardial infarction.
- Known bleeding disorders or family history of bleeding disorders.
- Any diseases or conditions in which the use of DLM, BDQ, OPC-167832, rifampin, isoniazid, pyrazinamide, or ethambutol is contraindicated.
- Clinical evidence of severe extrapulmonary TB (eg, miliary TB, abdominal TB, urogenital TB, osteoarthritic TB, TB meningitis).

- Evidence of pulmonary silicosis, lung fibrosis, or other lung condition considered as severe by the investigator (other than TB). In particular, any underlying condition that could interfere with the assessment of x-ray images, sputum collection, or interpretation of sputum findings, or otherwise compromise the subject's participation in the trial.
- Any renal impairment characterized by creatinine clearance/estimated glomerular filtration rate of < 60 mL/min, hepatic impairment characterized by alanine transaminase or aspartate transaminase > 2.0 × upper limit of normal of the clinical laboratory reference range or bilirubin > 2.0 × upper limit of normal of the clinical laboratory reference range, at screening.
- Screening glucose (nonfasting) ≥ 200 mg/dL or glycosylated hemoglobin ≥ 6.5%.
- QT interval corrected by Fridericia's method > 450 msec in male subjects (> 470 msec in female subjects), atrioventricular block II or III, bi-fasicular block, at screening or current or history of clinically significant ventricular arrhythmias. Other ECG abnormalities, if considered clinically significant by the investigator.
- Subjects receiving any of the prohibited medications within the specified periods or who would be likely to require prohibited concomitant therapy during the trial.
- Current history of significant drug and/or alcohol abuse that is likely to result in poor adherence to trial requirements or that would pose a risk to the subject's well-being during the course of the trial.
- Subjects who test positive for cocaine or other drugs of abuse (excluding known prescription stimulants and other prescribed medications and marijuana) at screening are excluded. Detectable levels of alcohol, marijuana, barbiturates, or opiates in the drug screen are not exclusionary if, in the investigator's documented opinion, the subject does not meet Diagnostic and Statistical Manual of Mental Disorders, Fifth Edition criteria for moderate to severe substance use disorder and the positive test does not signal a clinical condition that would impact the safety of the subject or interpretation of the trial results, and participation is agreed to by the medical monitor prior to treatment.
- History of having taken an investigational drug within 30 days preceding trial entry.
- Subjects with significant medical comorbidities that in the opinion of the investigator, should not participate in the trial.
- Subjects with Karnofsky score < 60 will be excluded from the trial.

Trial Site(s):

This trial will be conducted at approximately 6 sites in South Africa.

Investigational Medicinal Product(s), Dose, Dosage Regimen, Treatment Duration, Formulation, Mode of Administration:

All investigational medicinal product (IMP) (ie, OPC-167832, DLM, or BDQ) must be administered after the completion of a meal.

Bedaquiline should be administered as follows: 400 mg QD for the first 2 weeks of treatment and 200 mg TIW thereafter with at least 48 hours between doses for the remainder of the trial (ie, a total of 17 weeks [Arm 1 or Arm 2 or Arm 3] of treatment).

Subjects randomized to treatment Arm 4 will receive RHEZ orally QD for 8 weeks followed by 18 weeks of rifampin and isoniazid per the local standard of care.

Trial treatments (IMP or RHEZ) will be administered using the locally accepted method of directly observed therapy (DOT). In addition, all doses of IMP or RHEZ given during trial visits will be observed via the hand-and-mouth procedure. All trial treatments will be administered with approximately 240 mL of still (noncarbonated) water.

Trial Assessments:

Assessments for Efficacy: Sputum for acid fast bacilli smear microscopy, MGIT culture, LAM concentration drug susceptibility testing, and *Mycobacterium tuberculosis* strain typing (as necessary); sputum for RS ratio; assessment of clinical signs and symptoms of TB; PET/CT scan; chest radiograph; and whole blood biomarkers.

Assessments for Safety: AE reporting; clinical laboratory tests (serum chemistry, hematology, urinalysis, and coagulation); physical examination; vital signs; ECGs; and concomitant medications.

Screening (excluding safety assessments mentioned above)/Other: demographics, medical history, prior medications; height and weight; visual assessment; sputum for confirmation of DS-TB; viral hepatitis serology (hepatitis B surface antigen [HBsAg] and hepatitis C virus [HCV]) and human immunodeficiency virus (HIV) screening; urine pregnancy testing; urine or blood alcohol, and urine drug screen; severe acute respiratory syndrome coronavirus (SARS-CoV-2) testing.

Assessments for PK: PK samples will be used to analyze concentrations of OPC-167832, DLM and its metabolite DM-6705, and BDQ and its metabolite M2.

[redacted]

Data Monitoring Committee:

An independent Data Monitoring Committee (DMC) will be contracted by Otsuka to review safety, efficacy, and PK data during the conduct of the trial. The DMC will convene and operate based on an established charter. The DMC will be permitted to seek additional independent expertise, as needed. The primary responsibility of the DMC will be to monitor safety and to advise the sponsor of the clinical project regarding whether or

not the safety concerns merit stopping the trial. Towards this objective the DMC will meet on a predetermined schedule to review safety and available efficacy and PK data.

Statistical Methods: Primary: Safety endpoints will be summarized using descriptive statistics (number, median, mean, standard deviation, minimum, maximum) for continuous values, and by frequency and percentage for discrete variables. Percentage and frequency of adverse events and clinical laboratory test results by grades will be analyzed.

The modified intent-to-treat (mITT) analysis set includes all randomized subjects who take at least 1 dose of IMP or RHEZ, have at least 1 postbaseline culture result, and demonstrate positive culture results by Week 1 and susceptibility to rifampin and isoniazid (from the screening specimen).

The primary efficacy endpoint of the trial is the proportion of subjects who have SCC and no subsequent positive culture at the end of treatment. Sputum culture conversion occurs when a subject has 2 visits of at least 28 days apart (± 4 days) with sputum cultures negative and without a positive sputum culture result in between.

The 3 DLM + BDQ + OPC-167832 arms will be pooled, and noninferiority with RHEZ will be established if the lower limit of 80% confidence interval (CI) for the difference of proportion of subjects with SCC between the pooled DLM + BDQ + OPC-167832 group is larger than -0.12. The Miettinen and Nurminen CI of common risk difference stratified by HIV status and cavitation status prior to randomization will be used.

The 80% CI of the proportion difference between each dose level of DLM + BDQ + OPC-167832 arm and RHEZ will be provided.

In exploratory analysis, the difference between proportion of subjects with favorable outcome between each DLM + BDQ + OPC-167832 group and [redacted] group will be studied.

Definition of favorable and unfavorable outcome:

A subject is considered to have a **favorable** outcome if, during the postrandomization evaluation period, the subject is alive, completed the postrandomization evaluation, and otherwise did not meet the definition of an unfavorable outcome.

A subject is considered to have an **unfavorable** outcome if one of the following occurs:

1) Clinical progression of pulmonary disease during treatment or the need for an unanticipated surgical intervention for TB (eg, lobectomy)
2) Any growth of *M tuberculosis* from an extrapulmonary site during the trial

3) Signs and symptoms of active tuberculosis during the follow-up period (ie, after the end of treatment), including radiographic worsening compared to baseline findings, resulting in re-initiation of anti-mycobacterial therapy (Note: excludes a re-initiation that is subsequently found to be due to a misdiagnosis and/or alternative diagnosis)
4) Death during treatment or follow-up
5) A sputum culture with growth of *M tuberculosis* at certain time points as follows:
   - Failure to achieve no growth of *M tuberculosis* on consecutive sputum cultures at the end of the treatment period that result in a change in anti-mycobacterial therapy.
   - Microbiologic relapse during the follow-up period (ie, after the end of treatment) (Note: Excludes reinfection and requires more than 1 positive sputum culture).

Pharmacokinetics/pharmacodynamics and/or exposure-response analysis will be used to analyze the relationship of OPC-167832 plasma exposure alone or combined with DLM and BDQ with the efficacy and safety endpoints, as applicable.

Trial Duration:

Each subject in this trial is expected to participate in the following periods of the trial (approximate durations listed):

- Eligibility screening period (up 14 days)
- Treatment period (Arm 1, Arm 2, and Arm 3: 17 weeks; or Arm 4: 26 weeks)
- Post-treatment follow-up period (12 months postrandomization)

Overall, the per-patient trial duration from signing of the first informed consent form to the final subject assessment is expected to be approximately 12 months.

1.2 Schema

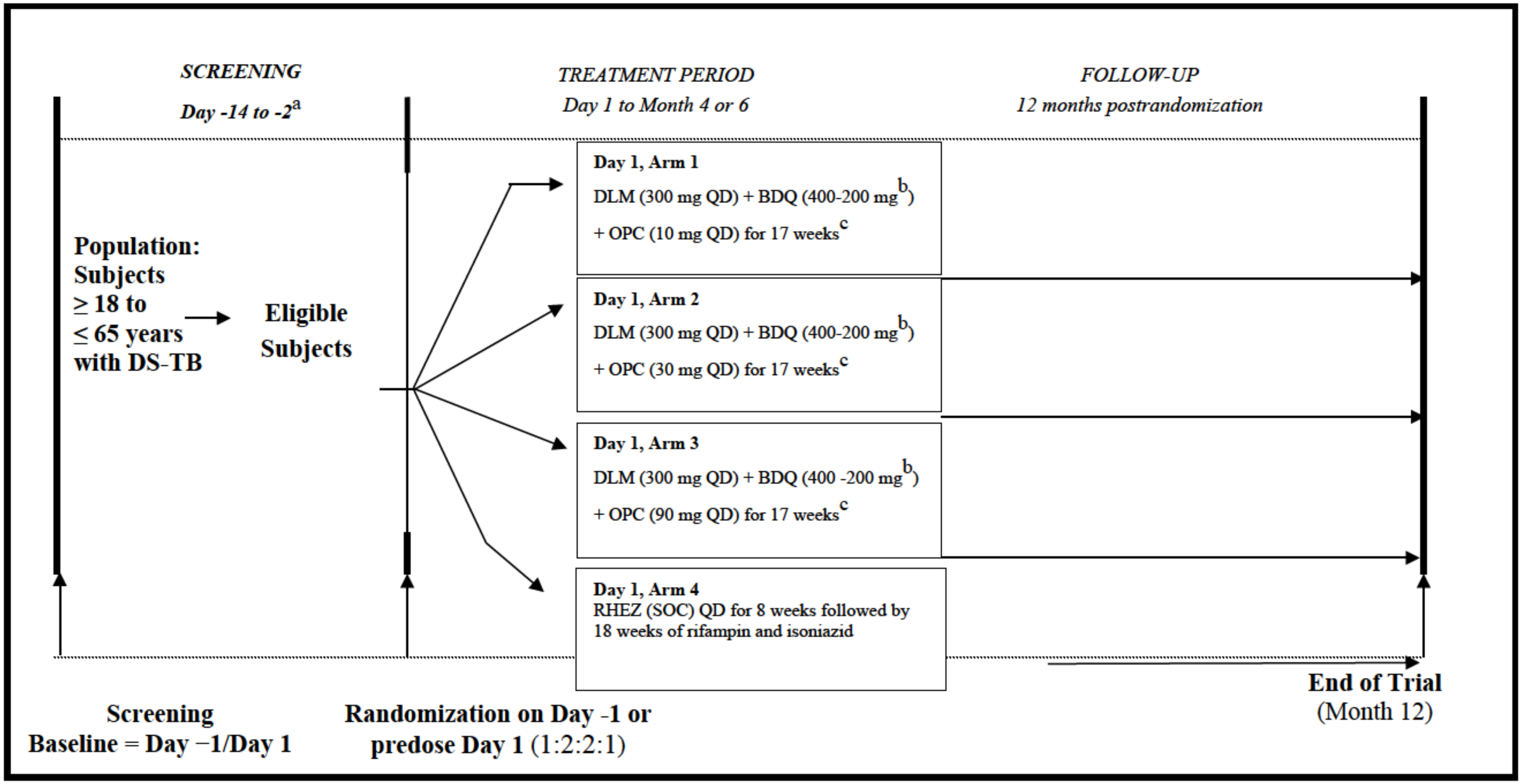

Figure 1.2-1 Trial Design Schematic

SOC = standard-of-care.

[a]Screening can occur for up to 14 days but should be completed in as few days as possible.

[b]BDQ dose 400 mg QD x 2 weeks, then 200 mg TIW.

[c]Rescue treatment consisting of the standard of care treatment, ie, RHEZ, will be provided to subjects in Arm 1, or Arm 2, or Arm 3 in the following circumstances: 1) If a subject is sputum culture positive for *M tuberculosis* at the end of treatment; 2) If a subject has a microbiologic relapse subsequent to the end of treatment; or, 3) If, in the opinion of the investigator, a subject is clinically deteriorating, based on any combination of signs, symptoms, or chest radiograph data. Subjects in Arm 4 will be managed according to local guidelines if not responding to therapy.

1.3 Schedule of Assessments

Table 1.3-1 Schedule of Assessments - All Subjects

Period	Pre-treatment Period		Treatment Period																		Follow-up Period								
	SCR	BSL																											
Visit	1	2	3	4	5	6	7	8	9	10	11	12	13	14	15	16	17	18	19	20	21	22	23	24	25	26	27	ET	Notes
	Day			Week (± 2 days)																			Week (± 7 days)						
Day/ Week	−14 to −2	−1	1	1	2	3	4	5	6	7	8	10	12	13	15	17	19	21	23	26[a]	28	30	34	39	43	47	52	N/A	
Informed consent	X																												Section 10.1.2
Demography	X																												Section 5.1
Concomitant medications	X	X	X	X	X	X	X	X	X	X	X	X	X	X	X	X	X	X	X	X	X	X	X	X	X	X	X	X	Section 6.5
Medical history	X																												Section 5.2
TB treatment history	X																												Section 5.2
Inclusion/ exclusion criteria	X	X																											Section 5.2
Chest radiograph[b]	X															X b				X[b]							X	X	Section 8.1.2
Random-ization		X																											
Physical examination	X	X[c]			X c		X[c]		X c		X[c]	X[c]	X[c]	X[c]	X c	X[c]		X[c]		X[c]		X[c]	X[c]	X[c]	X[c]	X[c]	X	X[c]	Section 8.7.2
Vital signs[d]	X	X			X		X		X		X	X	X	X	X	X		X		X	X	X	X	X	X	X	X	X	Section 8.7.3

Table 1.3-1 Schedule of Assessments - All Subjects

Period	Pre-treatment Period		Treatment Period																		Follow-up Period								
	SCR	BSL																											
**Visit**	1	2	3	4	5	6	7	8	9	10	11	12	13	14	15	16	17	18	19	20	21	22	23	24	25	26	27	ET	Notes
	Day			Week (± 2 days)																			Week (± 7 days)						
**Day/ Week**	−14 to −2	−1	1	1	2	3	4	5	6	7	8	10	12	13	15	17	19	21	23	26[a]	28	30	34	39	43	47	52	N/A	
Visual assessment	X																												Section 8.7.2
PET/CT scan[e]		X[e]					X[e]									X[e]				X[e]									Section 8.7.6
Standard 12-lead ECG	X	X			X		X		X		X	X	X	X	X	X		X		X		X	X	X	X	X	X	X	Section 8.7.4
Signs and symptoms of TB	X	X			X		X		X		X			X		X		X		X		X	X	X	X	X	X	X	Section 8.1.2
Hematology, serum chemistry, and urinalysis	X	X			X		X		X		X			X		X		X		X							X	X	Section 8.7.1
Coagulation	X																												Section 8.7.1
Urine drug screen	X																												Section 8.7.1
Urine pregnancy test FOCBP	X																										X	X	Section 10.3
HIV, HBsAg, anti-HCV	X																												Section 8.7.1
Testing for SARS-CoV-2	X																												Section 5.2.2
Sputum for eligibility[f]	X	X																											Section 8.1.1.1

Table 1.3-1 Schedule of Assessments - All Subjects

Period	Pre-treatment Period		Treatment Period																		Follow-up Period									
	SCR	BSL																												
Visit	1	2	3	4	5	6	7	8	9	10	11	12	13	14	15	16	17	18	19	20	21	22	23	24	25	26	27	ET	Notes
	Day			Week (± 2 days)																			Week (± 7 days)							
Day/ Week	−14 to −2	−1	1	1	2	3	4	5	6	7	8	10	12	13	15	17	19	21	23	26[a]	28	30	34	39	43	47	52	N/A	
Sputum for smear, MGIT culture/ sputum LAM		X	X g	X	X	X	X	X	X	X	X	X	X	X	X	X	X	X	X	X	X	X	X	X	X	X	X	X	Section 8.1.1
Sputum for RS ratio		X	X g	X	X	X	X	X	X	X	X	X	X	X	X	X	X	X	X	X	X	X	X	X	X	X	X	X	Section 8.1.1
M tuberculosis strain genotyping[h]		X	X	X												X	X	X	X	X	X	X	X	X	X	X	X	X	Section 8.1.1
Paxgene tube for transcrip-tomics	X	X			X		X		X		X			X		X		X		X							X	X	Section 8.5
IMP admin-istration[i]			X	X	X	X	X	X	X	X	X	X	X	X	X	X	X	X	X	X	X								Section 6
Compliance check of IMP[i]			X	X	X	X	X	X	X	X	X	X	X	X	X	X	X	X	X	X	X								Section 6.4
Sparse PK[j]			X		X		X				X			X		X												X	Section 8.2
AEs and IREs	X	X	X	X	X	X	X	X	X	X	X	X	X	X	X	X	X	X	X	X	X	X	X	X	X	X	X	X	Section 8.8

BSL= Baseline; ET = early termination; FOCBP = female of childbearing potential; IRE = immediately reportable event; SCR=Screening.

[a]The Week 26 visit will be performed only for subjects randomized to Arm 4.

[b]An end of treatment chest radiograph will be taken at Week 17 (for subjects in Arm 1, Arm 2, and Arm 3) or Week 26 (for subjects in Arm 4) and at Month 12.

[c]A targeted physical examination is a review that focuses on subject driven issues eg, the investigator enquires if the subject has any complaints, pains or disturbances and this would lead to further evaluation of the problematic area. At a minimum, an assessment of general appearance and a chest examination should be performed.

[d]At screening, blood pressure and heart rate will be taken with the subject in the supine (performed first) and sitting position after remaining for ≥ 3 minutes in each position and temperature and respiratory rate will be taken with the subject in the supine position. At all other time points, vital signs will be obtained after the subject has been at rest in the supine position for at least 1 minute.

[e]The PET/CT scan will be done fasting within 1 week prior to starting treatment, Week 4 (± 4 days), and at the end of treatment visit (± 4 days) for each group (ie, Week 17 for Arm 1, Arm 2, and Arm 3, and Week 26 for Arm 4).

[f]A sputum sample will be collected during the screening period (Day -14 to Day -2) and baseline Day -1.

[g]The Day 1 sputum will be collected prior to the first dose of IMP.

[h]*M tuberculosis* genotyping will be performed on a baseline culture (Day -1 and/or Day 1, or Week 1 if necessary) and the first of any subsequent positive culture during the posttreatment follow-up period to determine if the subject has relapsed or has new infection of *M tuberculosis*. In addition, the sponsor may request genotyping as needed.

[i]Subjects in Arm 1, Arm 2, and Arm 3 will receive DLM and OPC-167832 QD for 17 weeks combined with BDQ QD for 2 weeks, then BDQ TIW. Subjects in Arm 4 will receive RHEZ QD for 8 weeks followed by rifampin and isoniazid for 18 weeks per the local standard of care.

[j]For subjects randomized to Arm 1, Arm 2, and Arm 3, PK samples will be taken on Day 1 (predose and postdose), and then Week 2 (predose), Week 4 (predose), Week 8 (predose and postdose), Week 13 (predose), and Week 17/ET (predose and postdose) (9 samples per subject). The PK samples will be drawn based on the DLM dosing for Arm 1, Arm 2, and Arm 3.

2 Introduction

Tuberculosis (TB) remains a major global health problem and is the leading cause of death from an infectious disease worldwide.[1] Progress has been made in the research and development of new anti-tuberculosis drugs to help overcome the current TB treatment situation. In recent years, bedaquiline (BDQ) (Sirturo™) and the sponsor's delamanid (DLM) (Deltyba™) have been used as part of a combination therapy in adults with pulmonary multidrug-resistant tuberculosis (MDR-TB). These 2 new drugs offer the opportunity to form the backbone for a novel oral anti-TB drug regimen that can shorten the treatment for both drug-susceptible tuberculosis (DS-TB) and MDR-TB.[1,2,3] Thus, there is an urgent need for the development of new potent anti-TB agents with low toxicity that are effective against drug-susceptible and drug-resistant strains of *Mycobacterium tuberculosis* and can be combined with BDQ and DLM.[4]

Otsuka Pharmaceutical Co, Ltd (Otsuka) initiated a program to screen for new anti-TB agents that can be used in combination with DLM. As a result of research efforts, Otsuka discovered OPC-167832, a novel 3, 4 dihydrocarbostyril derivative. OPC-167832 is an inhibitor of decaprenylphosphoryl-β-D-ribose 2'-oxidase (DprE1), an essential enzyme for *M tuberculosis* cell wall biosynthesis. The minimum inhibitory concentrations (MIC) of OPC-167832 for *M tuberculosis* on clinically isolated strains, including MDR-/extensively drug-resistant-TB, ranged from 0.00024 to 0.002 µg/mL. It has bactericidal activity against both growing and intracellular bacilli. The frequency of spontaneous resistance of OPC-167832 was 2.60×10^{-9} to 1.52×10^{-7} for *M tuberculosis* H37Rv at 16 × MIC.

Please refer to the OPC-167832 Investigator's Brochure (IB) for more detailed information.[5]

2.1 Trial Rationale

In vitro and in vivo studies have shown that OPC-167832 has potent bactericidal activity.[5] Further, OPC-167832 in combination with DLM or BDQ may have additive effects on bactericidal activity. No antagonistic activity has been shown with OPC-167832 and DLM or OPC-167832 and BDQ. Because of OPC-167832's high bactericidal activity, and novel mechanism of action (different from all other TB drugs currently available for human use), it is likely a good candidate to be included in a regimen to possibly improve efficacy and prevent resistance development to concomitant TB drugs.[5]

Single oral doses up to 480 mg OPC-167832 were well tolerated in healthy subjects; there were no serious or severe adverse events (AEs) and no subjects were discontinued from the trial due to a treatment emergent AE. One subject did decide to withdraw consent after occurrence of the nonserious and mild AEs of arthralgia, pain in extremity, and ecchymosis.

The safety, tolerability, pharmacokinetics (PK), and efficacy of OPC-167832 are being evaluated in adult subjects with uncomplicated, smear-positive, pulmonary DS-TB, in an ongoing, multiple-dose trial. Stage 1, which was testing multiple ascending doses of OPC-167832 has been completed. Clinically meaningful early bactericidal activity as measured by change from baseline at 14 days in Log10 colony-forming unit (CFU) was seen across all doses of OPC-167832 tested. OPC-167832 demonstrated an average decline of 0.101, 0.138, 0.151, and 0.149 Log10 CFU/mL per day in sputum specimens obtained from subjects administered with 3 mg, 10 mg, 30 mg, and 90 mg per day, respectively, versus 0.200 Log10 CFU/mL per day for the rifampin, isoniazid, ethambutol, and pyrazinamide (RHEZ) arm. There was a single serious adverse event (SAE) of haemoptysis that led to discontinuation of investigational medicinal product (IMP) (3 mg dose); however, this SAE was not considered to be related to the IMP.

The proposed treatment regimen in this trial has not yet been evaluated in a clinical trial but is supported by the clinical and nonclinical data on DLM, BDQ, and OPC-167832. In a mouse chronic TB model, the 3-drug combination of OPC-167832, DLM, and BDQ significantly killed *M tuberculosis* faster than the standard care of treatment regimen of RHEZ[4] and is more efficacious in a hollow fiber system of tuberculosis (HFS-TB). In the HFS-TB, the 3-drug combination sterilized the system much faster than the standard treatment regimen and suggested a potential 3 to 4 months treatment regimen.

This trial will evaluate the safety, tolerability, pharmacokinetics (PK), and efficacy of 2 doses of OPC-167832 combined with DLM and BDQ in subjects who are ≥ 18 to ≤ 65 years old with pulmonary DS-TB. Standard-of-care treatment with RHEZ will be included for comparison.[6]

2.2 Background

Delamanid and BDQ are 2 of the most recently developed anti-TB drugs that have been extensively studied in patients with MDR-TB, and have received regulatory approval and World Health Organization endorsement to be used as part of an optimal background regimen for patients with MDR-TB. Delamanid is a nitro-dihydro-imidazooxazole derivative that inhibits the synthesis of mycolic acid, an important component of the *M tuberculosis* cell wall. Bedaquiline is a diarylquinoline derivative that inhibits

M tuberculosis adenosine triphosphate synthase. Both DLM and BDQ have been studied together as part of a National Institutes of Health-sponsored combination drug-drug interaction/safety trial (NCT02583048).

OPC-167832 is a newly discovered 3,4-dihydrocarbostyril derivative that is an inhibitor of DprE1, an essential enzyme for *M tuberculosis* to synthesize lipoarabinomannan (LAM) and arabinogalactan (key components of the *M tuberculosis* cell wall). OPC-167832 is currently under clinical development: a single ascending dose trial in healthy subjects (Trial 323-201-00001) has been completed, and a multiple, ascending-dose and early bactericidal activity trial in subjects with DS-TB has completed Stage 1 and is ongoing (Trial 323-201-00003).

These 3 drugs have distinct mechanisms of actions against *M tuberculosis* that are also distinct from other approved TB drugs (one notable exception is pretomanid, which has a similar mechanism of action and likely near complete cross-resistance to DLM).

2.3 Known and Potential Risks and Benefits

2.3.1 OPC-167832

In a 1-week repeated-dose oral toxicity study in rats, slightly suppressed body weight gain and slightly decreased food consumption were observed. In blood biochemistry, increases in total cholesterol and phospholipids and decreases in creatinine were observed.

In 1-, 4-, and 26-week toxicity studies in rats, no drug-related deaths occurred in either males or females at any dose tested. Suppressed body weight gain and/or decreased food consumption were observed and increases in total cholesterol and phospholipids and decreases in creatinine were observed at 1 week. In the 4-week repeated-dose oral toxicity study in rats, suppressed body weight gain and decreased food consumption were observed. These changes showed reversibility or a tendency for reversibility. The no observed adverse effect level (NOAEL) was estimated to be 2000 mg/kg/day for males and 200 mg/kg/day for females. At the NOAEL, the maximum (peak) plasma concentration (C_{max}) and area under the concentration-time curve from zero to 24 hours (AUC_{24h}) of OPC-167832 in Week 4 (on Day 28) were respectively 6.863 μg/mL and 96.91 μg·h/mL in males and 9.205 μg/mL and 130.7 μg·h/mL in females. In a 1-week repeated-dose oral toxicity study in dogs, decreased body weight or suppressed body weight gain, or both, as well as relatively low food consumption were observed.

In 1-, 4-, 13- and 39-week toxicity studies in dogs, no treatment related deaths occurred in either males or females at any dose tested. Increases in creatinine kinase, alanine

aminotransferase (ALT), aspartate aminotransferase (AST), lactate dehydrogenase, and potassium were observed in the 4-week toxicity study at the highest dose (2000 mg/kg/day). Increases in total cholesterol and phospholipids were also observed in the 4- and 13-week toxicity studies at doses of 100 mg/kg/day and higher. In the 4-week toxicity study, slight prolongation of QT interval and QT interval corrected (QTc) was observed at Week 4 in both sexes at 2000 mg/kg/day. However, no prolongation of QT interval and QTc was noted in the 13-week toxicity study in either sex at up to 100 mg/kg.

In 4- and 13-week repeated-dose oral toxicity studies in dogs, decreased body weight or suppressed body weight gain, or both, and decreased food consumption were observed. In blood biochemistry, changes in creatinine kinase, lactate dehydrogenase, alanine aminotransferase, aspartate aminotransferase, total cholesterol, phospholipids, α_1-globulin content and ratio, potassium, and myoglobin were observed. In electrocardiography, a slight prolongation of QT interval and QTc was observed only at the highest dose in the 4-week repeated-dose oral toxicity study. In histopathological examination, changes in the muscle fibers were observed. Stress-related changes secondary to body weight loss were observed in the thymus and adrenal grand. These changes showed reversibility or a tendency for reversibility. The NOAEL was 3 mg/kg in beagle dogs in the 13-week toxicity study.[5]

Single oral doses up to 480 mg OPC-167832 were well tolerated during Trial 323-201-00001; there were no serious or severe AEs but 1 subject withdrew consent after occurrence of the nonserious and mild AEs of arthralgia, pain in extremity, and ecchymosis.[5] In the single ascending dose trial of OPC-167832 (Trial 323-201-00001), where single oral doses up to 480 mg were administered to healthy subjects, a slight negative correlation was observed between changes in QT interval corrected by Fridericia's method (QTcF) from baseline and placebo, and OPC-167832 plasma concentrations.

The safety, tolerability, PK, and efficacy of OPC-167832 are being evaluated in adult subjects with uncomplicated, smear-positive, DS-TB, in an ongoing, multiple-dose trial (Trial 323-201-00003). Stage 1, which was testing multiple ascending doses of OPC-167832 has been completed. Clinically meaningful early bactericidal activity as measured by change from baseline at 14 days in Log10 CFU was seen across all doses of OPC-167832 tested. There was a single SAE of haemoptysis that led to discontinuation of IMP (3 mg dose); however, this SAE was not considered to be related to the IMP.

Trial sites will receive updated versions of the IB, when available, and trial sites should refer to the most current version as needed.

2.3.2 Delamanid

The first marketing approval for DELTYBA® (DLM) was granted on 28 Apr 2014 in Europe (Austria, Belgium, Bulgaria, Croatia, Cyprus, Czech Republic, Denmark, Estonia, Finland, France, Germany, Greece, Hungary, Iceland, Ireland, Italy, Latvia, Liechtenstein, Lithuania, Luxembourg, Malta, Netherlands, Norway, Poland, Portugal, Romania, Slovakia, Slovenia, Spain, Sweden), and United Kingdom, followed by approvals in Japan (04 Jul 2014), South Korea (29 Oct 2014), Hong Kong (29 Mar 2016), Turkey (31 May 2017), India (24 Aug 2017), the Philippines (08 Sep 2017), China (12 Mar 2018), Mongolia (05 Dec 2018), Ukraine (30 Jan 2019), South Africa (25 Mar 2019), Peru (01 Jun 2019), Kazakhstan (08 Aug 2019), Turkmenistan (22 Sep 2019), and Russia (08 May 2020). In all completed and ongoing clinical trials of DLM, as of 31 Jan 2019, including healthy subjects and subjects with TB (uncomplicated DS-TB, MDR-TB, and MDR TB refractory to treatment), 1419 subjects received at least 1 dose of DLM.

In the 14 completed trials in healthy subjects, the treatment-emergent adverse events (TEAEs) that led to the discontinuation of the IMP (ie, DLM) in more than 1 subject in the Any DLM group were rash generalised (12 subjects), haematochezia (3 subjects), and headache (3 subjects). There were 2 serious TEAEs, delirium and ischaemic colitis, both occurring in Trial 242-07-209. No deaths were reported in any of the completed trials in healthy subjects.

In the 2 completed trials of subjects with uncomplicated DS-TB treated with DLM, there were no deaths, and no subjects discontinued IMP due to TEAEs. There were 5 serious TEAEs (3 events of prolonged electrocardiogram (ECG) QT interval and 2 events of increased transaminases), all occurring in Trial 242-06-101.

In the 4 completed trials of subjects with MDR-TB, the only TEAE that led to the discontinuation of IMP in more than 1 subject in the DLM + optimized background regimen (OBR) group was prolonged ECG QT interval (7 subjects in the DLM + OBR group and 2 subjects in the placebo + OBR group). The serious TEAEs that occurred in at least 1% of subjects in the DLM + OBR group and at a higher incidence than in the placebo + OBR group were prolonged ECG QT interval (3.4% DLM + OBR, 1.2% placebo + OBR), TB (2.1% DLM + OBR, 0.9% placebo + OBR), and hypokalaemia (1.6% DLM + OBR, 0.9% placebo + OBR).

Additional information regarding the safety and the potential risks and benefits of DLM is provided in the IB.[3]

2.3.3 Bedaquiline

Bedaquiline (SIRTURO®) is a diarylquinoline antimycobacterial drug indicated as part of combination therapy in adult and pediatric patients (≥ 5 years and weighing ≥ 15 kg) with pulmonary MDR-TB. For the known and potential risks of SIRTURO, please see the package insert for SIRTURO (BDQ) tablets.[2]

2.3.4 Rifampin, Isoniazid, Ethambutol, and Pyrazinamide

RIFAFOUR® tablets are a combination of four first line agents (RHEZ) used in the treatment of TB. Rifampin is a semi-synthetic, broad-spectrum bactericidal antibiotic. Isoniazid is a synthetic, antitubercular agent which is bacteriostatic against semi-dormant bacilli and bactericidal against actively dividing mycobacteria. Pyrazinamide may be bactericidal or bacteriostatic, depending on its concentration and the susceptibility of the organism. Ethambutol is a synthetic, bacteriostatic antitubercular agent. All agents are readily absorbed following oral administration, with wide distribution to most tissues and fluids including cerebrospinal fluid. For the complete list of known and potential risks of RHEZ, please see the package insert for RIFAFOUR single-dose combination tablets.[6]

3 Objectives and Endpoints

Table 3-1 Trial Objectives and Endpoints

Objectives	Endpoints
Primary:<br>• To assess the safety and efficacy of OPC-167832 combined with DLM and BDQ in subjects with DS-TB administered for 4 months compared to RHEZ administered for 6 months	Safety:<br>• AEs, clinical laboratory tests, vital signs, physical examinations, ECGs<br>• The proportion of subjects with a grade 3 or higher AE<br>• The rate of all-cause treatment discontinuation<br><br>Efficacy:<br>• The proportion of subjects achieving sputum culture conversion (SCC) in Mycobacteria Growth Indicator Tube® (MGIT) by the end of the treatment period

Table 3-1 Trial Objectives and Endpoints

Objectives	Endpoints
Secondary:<br>• To evaluate efficacy in sputum MGIT culture and LAM for OPC-167832 combined with DLM and BDQ administered for 4 months as compared to RHEZ<br>• To evaluate the development of resistance to anti-TB drugs in all treatment arms<br>• To compare time to SCC of OPC-167832, DLM, and BDQ to RHEZ<br><br>PK:<br>• To evaluate the PK of OPC-167832 combined with DLM and BDQ in subjects with DS-TB	Efficacy:<br>• The proportion of subjects who achieve SCC in MGIT by 8 weeks of treatment<br>• Time to detection of MGIT cultures<br>• The proportion of subjects who convert sputum LAM to negative by 8 weeks of treatment and by end of treatment<br>• Proportion of subjects who develop drug resistance<br>• Time to SCC<br><br>PK:<br>• The concentrations of OPC-167832, DLM and its metabolite DM-6705, and BDQ and its metabolite M2
[redacted]	[redacted]

Section 9.4 describes the statistical analysis of the endpoints.

4 Trial Design

4.1 Type/Design of Trial

Trial 323-201-00006 is a multicenter, phase 2b/c, open-label, randomized, dose-finding trial in subjects with DS-TB. The microbiology laboratory will be blinded to treatment assignments.

Following a screening period of up to 14 days, eligible subjects will be randomized on Day −1 or predose Day 1 in a ratio of 1:2:2:1 to one of the following treatment arms:

- Arm 1: DLM (300 mg once daily [QD]) + BDQ (400 mg QD x 2 weeks, then 200 mg thrice-weekly [TIW]) + OPC-167832 (10 mg QD) for 17 weeks
- Arm 2: DLM (300 mg QD) + BDQ (400 mg QD x 2 weeks, then 200 mg TIW) + OPC-167832 (30 mg QD) for 17 weeks
- Arm 3: DLM (300 mg QD) + BDQ (400 mg QD x 2 weeks, then 200 mg TIW) + OPC-167832 (90 mg QD) for 17 weeks
- Arm 4: RHEZ for 8 weeks followed by 18 weeks of rifampin and isoniazid (for a total of 26 weeks)

At the end of the treatment period, subjects will be followed until 12 months postrandomization.

4.2 Scientific Rationale for Trial Design

Treatment of TB requires multiple drugs with different mechanisms of action and the ability to kill *M tuberculosis* bacilli within heterogeneous TB lesions (ie, different pathophysiologic environments) in order to ensure rapid sterilization of lesions, a durable relapse-free cure, and prevent the development of resistance. Development of novel TB treatment regimens that are comprised of potent new TB drugs that can shorten the treatment duration to 4-months or less will simplify TB treatment, enhance compliance, and potentially improve treatment outcomes.

This trial will be conducted to evaluate a new proposed treatment regimen that is supported by the clinical and nonclinical data on DLM, BDQ, and OPC-167832. In vitro and in vivo nonclinical data demonstrate that the proposed 3-drug combination is more efficacious than the current 6-month standard treatment in a mouse model and a HFS-TB. Importantly, HFS-TB data suggest that the 3-drug combination has the potential to cure TB in 2 to 3 months. Furthermore, the drug exposures used in these studies are generally in accordance with those that can be achieved with the proposed human doses.

Using the HFS-TB model, the 3-drug combination of DLM+BDQ+OPC-167832 killed TB bacilli faster than any of the 2-drug combinations as well as the standard therapy (RHEZ), in either the low pH or log-phase culture conditions. Unexpectedly, the DLM and BDQ 2-drug combination not only did not kill bacteria but paradoxically appeared to enhance bacterial growth (this phenomenon was confirmed in separate experiments). Currently, there is no explanation for this observation, but is likely unique to the HFS-TB, since the DLM and BDQ combination was additive in a mouse study and indifferent to additive in the in vitro checkerboard assay. Nevertheless, when OPC-167832 was added to the combination of DLM and BDQ, the combination became very effective. Using a model developed to convert the HFS-TB data to TB treatment duration in patients, the 3-drug regimen is predicted to cure DS-TB patients in 2 to 3 months.

This trial will evaluate the safety, tolerability, PK, and efficacy of OPC-167832 combined with DLM and BDQ in subjects with DS-TB. Standard-of-care treatment with RHEZ will be included for comparison.

4.3 Dosing Rationale

A key goal of the trial is to determine if the combination of DLM, OPC-167832, and BDQ is efficacious, safe and can potentially shorten the treatment duration in patients with DS-TB. In order to maximize the chances of success in achieving this goal, doses were chosen such that they can provide the highest possible exposures of the drugs without compromising patient safety. Since OPC-167832 has only been evaluated in the 14-day early bactericidal activity (EBA) trial in subjects with TB, and long-term efficacy and safety are not available, 2 doses of this compound will be evaluated in this trial. Both doses are selected to achieve near maximal efficacy based on the pharmacokinetics/pharmacodynamics (PK/PD) analyses of the EBA trial. Delamanid QD dosing regimen in combination with QD dosing of OPC-167832 and BDQ is being proposed in this trial due to the need to facilitate patient adherence.

Delamanid exposure-response modeling using DLM steady state area under the concentration-time curve (AUC) and change from baseline in CFU found cumulative fraction of response (CFR) of 96 to 98% and 89 to 96% following the approved DLM dose of 100 mg twice daily (BID) (from Trial 242-07-204) and the 200 mg QD dose regimen (from Trial 242-09-213), respectively. Following dosing of DLM 300 mg QD (from Trials 242-06-201 and 242-08-211), DLM AUC were about 20 to 25% higher than those obtained following 200 mg QD and hence correspondingly, the CFR also would be higher than that of the 200 mg QD dose. From a safety perspective, the exposures following 300 mg QD were lower than that following the approved 100 mg BID dosing,

indicating that the 300 mg QD dose would likely have less of an effect on a key safety issue, QT interval prolongation, compared to the approved 100 mg BID dose. Hence the dose of 300 mg QD DLM has been selected for the trial.

In the current multiple ascending dose (MAD)/EBA trial of OPC-167832 (Trial 323-201-00003), doses of 3 mg, 10 mg, 30 mg, and 90 mg have been studied. An exposure-response analysis of OPC-167832 of data obtained to date from the MAD/EBA trial indicated that the effect of 10 mg, 30 mg and 90 mg doses on CFU reduction were similar while the effect from the 3 mg dose is slightly less (ie, CFU count). All 4 doses were efficacious and well tolerated with no significant safety concerns. Because the efficacy and safety of long-term use in combinations with other TB drugs of OPC-167832 are unknown, in order to maximize exposures, and at the same time have a reasonable safety margin, the 10 mg, 30 mg, and 90 mg doses of OPC-167832 were chosen for the trial.

4.4 End of Trial Definition

The end of trial date is defined as the last date of contact or the date of final contact attempt from the post-treatment follow-up electronic case report form (eCRF) page for the last subject completing or withdrawing from the trial.

4.5 Definition of Completed Subjects

The treatment period is defined as the time period during which subjects are evaluated for primary and/or secondary objectives of the trial irrespective of whether or not the subject actually consumes all doses of the IMP. Subjects who are evaluated at the last scheduled visit during the treatment period will be defined as treatment completers (ie, they completed treatment). For purposes of this trial, subjects who complete Week 17 (for Arm 1, Arm 2, and Arm 3) or Week 26 (for Arm 4) visit will be defined as trial completers.

5 Trial Population

The trial population will consist of subjects who are ≥ 18 years to ≤ 65 years at screening and who have newly diagnosed pulmonary DS-TB. Approximately 120 subjects will be enrolled at sites in South Africa.

5.1 Subject Selection and Numbering

All subjects will be given a unique subject identifier (identification [ID]; site number [3 digits] + subject number [‘S’ + 5 digits] upon providing consent). The site number will

be designated by the sponsor. Subject numbers will be assigned sequentially across the trial from S00001.

Demographic information (collection date, date of birth, sex, childbearing potential, race, ethnicity, country) and medical history will be recorded in the eCRF at the screening visit.

5.2 Eligibility Criteria

Exceptions for eligibility criteria will not be permitted during the trial, neither by the investigator nor by the medical monitor.

5.2.1 Inclusion Criteria

Subjects are required to meet the following inclusion criteria when assessed:

1) Able to provide written, informed consent prior to initiation of any trial-related procedures or treatments, and able, in the opinion of the investigator, to comply with all the requirements of the trial.
2) Male or female subjects between 18 and 65 years of age (inclusive) at the screening visit.
3) Body weight ≥ 35.0 kg at the screening visit.
4) Newly diagnosed, rifampin and isoniazid susceptible (on the screening sample) pulmonary TB.
5) Able to spontaneously produce sputum.
6) Negative human immunodeficiency virus (HIV) status at screening.
7) Females of childbearing potential (FOCBP) must agree to use 2 different approved methods of birth control or remain abstinent throughout the participation in the trial and for 12 weeks after the last dose of IMP or dose of RHEZ.
8) Male subjects must agree to use 2 different approved methods of birth control or remain abstinent throughout the participation in the trial and for 12 weeks after the last dose of IMP or RHEZ.

5.2.2 Exclusion Criteria

Subjects will be excluded if they meet any of the following exclusion criteria when assessed:

1) Subjects are known or suspected of having resistance to rifampin, isoniazid, ethambutol, pyrazinamide, DLM, or BDQ either confirmed by the laboratory, or based on epidemiological history, at screening.
2) Evidence of clinically significant metabolic (including ongoing or current hypokalemia [ie, potassium <3.5 mEq/dL at screening]), gastrointestinal,

neurological, psychiatric, endocrine or liver (eg, hepatitis B and C) disease; malignancy; or other abnormalities (other than the indication being studied).

3) History of, or current, clinically relevant cardiovascular disorder such as heart failure, coronary heart disease, uncontrolled hypertension, arrhythmia or symptom strongly suggestive of such a problem (for example, syncope or palpitations), tachyarrhythmia or status after myocardial infarction.
4) Known bleeding disorders or family history of bleeding disorders.
5) Any diseases or conditions in which the use of DLM, BDQ, OPC-167832, rifampin, isoniazid, pyrazinamide, or ethambutol is contraindicated.
6) Any prior treatment for *M tuberculosis* within the past 2 years.
7) Any treatment with a drug active against *M tuberculosis* (eg, quinolones) within the 3 months prior to screening.
8) Clinical evidence of severe extrapulmonary TB (eg, miliary TB, abdominal TB, urogenital TB, osteoarthritic TB, TB meningitis).
9) Evidence of pulmonary silicosis, lung fibrosis, or other lung condition considered as severe by the investigator (other than TB). In particular, any underlying condition that could interfere with the assessment of x-ray images, sputum collection, or interpretation of sputum findings, or otherwise compromise the subject's participation in the trial.
10) Any renal impairment characterized by creatinine clearance/estimated glomerular filtration rate (eGFR) of < 60 mL/min, or hepatic impairment characterized by alanine transaminase or aspartate transaminase > 2.0 × upper limit of normal of the clinical laboratory reference range or bilirubin > 2.0 × upper limit of normal of the clinical laboratory reference range, at screening.
11) Screening glucose (nonfasting) ≥ 200 mg/dL or glycosylated hemoglobin (HbA1c) ≥ 6.5%.
12) QTcF > 450 msec in male subjects (> 470 msec in female subjects), atrioventricular block II or III, bi-fasicular block, at screening or current or history of clinically significant ventricular arrhythmias. Other ECG abnormalities, if considered clinically significant by the investigator.
13) Subjects receiving any of the prohibited medications (see Section 6.5.1) within the specified periods or who would be likely to require prohibited concomitant therapy during the trial.
14) Female subjects who are breast-feeding or who have a positive pregnancy test result prior to receiving the first dose of IMP or RHEZ on Day 1.
15) Current history of significant drug and/or alcohol abuse that is likely to result in poor adherence to trial requirements or that would pose a risk to the subject's well-being during the course of the trial.
16) History of HIV or current hepatitis or carriers of hepatitis B surface antigen (HBsAg) and/or anti hepatitis C virus (HCV).
17) Subjects who test positive for cocaine or other drugs of abuse (excluding known prescription stimulants and other prescribed medications and marijuana) at screening are excluded. Detectable levels of alcohol, marijuana, barbiturates, or

opiates in the drug screen are not exclusionary if, in the investigator's documented opinion, the subject does not meet Diagnostic and Statistical Manual of Mental Disorders, Fifth Edition criteria for moderate to severe substance use disorder and the positive test does not signal a clinical condition that would impact the safety of the subject or interpretation of the trial results, and participation is agreed to by the medical monitor prior to treatment.

18) History of having taken an investigational drug within 30 days preceding trial entry.
19) A history of difficulty in donating blood.
20) Donation of blood or plasma within 30 days prior to dosing.
21) History of serious mental disorders that, in the opinion of the investigator, would exclude the subject from participating in this trial.
22) Any known prior exposure to OPC-167832, DLM, or BDQ.
23) Subjects with significant medical comorbidities that in the opinion of the investigator, should not participate in the trial.
24) Subjects with Karnofsky score < 60 will be excluded from the trial.
25) Subjects testing positive for active severe acute respiratory syndrome coronavirus (SARS-CoV-2) infection at screening.

A definition of childbearing potential can be found in Section 10.3.

Subjects must agree to restrictions to lifestyle and medications described in Section 5.3 and Section 6.5.1, respectively.

5.3 Lifestyle Considerations

Not applicable.

5.3.1 Meals and Dietary Restrictions

Consumption of grapefruit, grapefruit products, Seville oranges, or Seville orange products within 72 hours prior to the first dose of IMP (ie, DLM, OPC-167832, or BDQ) or RHEZ and during the trial is prohibited.

5.3.2 Caffeine, Alcohol, and Tobacco

Subjects will be encouraged to refrain from drinking alcoholic beverages or using illicit drugs during participation in the trial.

5.4 Screen Failures

Subjects who sign an informed consent form (ICF) but who are not started on treatment are permitted to be rescreened. In the event that the subject is rescreened for trial participation, and the rescreening was not completed within the original screening window, a new ICF must be signed. Subjects excluded for positive drug/alcohol screen

are not eligible to be rescreened for participation in the trial. However, subjects excluded for other reasons may be rescreened at any time if the exclusion characteristic has changed. In the event that the subject is rescreened a new ICF must be signed, a new screening number assigned, and all screening procedures repeated.

A screen failure is a subject from whom informed consent is obtained and is documented in writing (ie, subject signs an ICF), but who is not randomized or assigned trial treatment. All AEs must be reported after subject informed consent has been obtained, including screening failures due to AEs, irrespectively of IMP administration.

If the subject meets the definition of a screen failure in this trial, the following information will be recorded in the eCRF:

- Date of informed consent
- Visit date (screening visit)
- Demographics (collection date, birth date, sex, race, ethnicity, country)
- Screen failure date
- Reason for screen failure

6 Trial Treatments

6.1 Trial Treatments Administered

Treatment Arms 1, 2, and 3:

All IMP (ie, OPC-167832, DLM, or BDQ) must be administered after the completion of a meal.

Bedaquiline should be administered as follows: 400 mg QD for the first 2 weeks of treatment and 200 mg TIW thereafter with at least 48 hours between doses for the remainder of the trial (ie, a total of 17 weeks [Arm 1 or Arm 2 or Arm 3] of treatment).

IMP will be administered using the locally accepted method of directly observed therapy (DOT). In addition, all doses of IMP given during trial visits will be observed via the hand-and-mouth procedure. IMP will be administered with approximately 240 mL of still (noncarbonated) water.

Treatment Arm 4:

Subjects randomized to treatment Arm 4 will receive RHEZ orally QD for 8 weeks followed by 18 weeks of rifampin and isoniazid per the local standard of care.

RHEZ will be administered using the locally accepted method of DOT. In addition, all doses of RHEZ given during trial visits will be observed via the hand-and-mouth

procedure. RHEZ will be administered with approximately 240 mL of still (noncarbonated) water. The RHEZ will be taken 1 hour prior to or 2 hours after a meal.

For information regarding the dose regimen and treatment period(s), including any follow-up period(s) for each treatment group/arm of the trial, see Section 4.1.

6.1.1 Medical Devices

Not applicable.

6.2 Management of Investigational Medicinal Product

The IMPs to be administered during this trial include OPC-167832, DLM, and BDQ.

The reference product to be administered is RHEZ (RIFAFOUR e-275 tablets) and rifampin/isoniazid.

For full details on IMP management, please refer to the OPC-167832 and DLM IBs and BDQ prescribing information.

6.2.1 Packaging and Labeling

Investigational medicinal product will be provided by the sponsor or designated agent to the investigators and the persons designated by the investigator(s) or institution(s). The IMP will be supplied as packages. OPC-167832 or DLM may not be repackaged by the trial site.

OPC-167832 and DLM will be provided to the investigator(s) and the persons designated by the investigator(s) or institutions by the sponsor or designated agent.

OPC-167832 and DLM will be supplied as follows:

- OPC-167832 will be supplied as 10-mg or 30-mg immediate-release tablets
- DLM will be supplied as 50-mg tablets

Each bottle or carton of OPC-167832 or DLM provided by the sponsor will be labeled to clearly disclose the compound identity, protocol number, the sponsor's name and address, instructions for use, route of administration, appropriate precautionary statements, and other information required by local regulatory authorities.

Both BDQ and RHEZ (RIFAFOUR single dose combination tablets) will be sourced in country.

6.2.2 Storage

The IMP will be stored in a securely locked cabinet or enclosure. Access will be limited to investigators and their designees. Neither the investigator(s) nor any designees may provide IMP or RHEZ to any subject not participating in this protocol.

The IMP and RHEZ will be stored according to the storage conditions indicated on the clinical labels.

The clinical trial site staff will maintain a temperature log in the drug storage area recording the temperature at least once each working day.

6.2.3 Accountability

The investigator or designee must maintain an inventory record of IMP and RHEZ received, dispensed, administered, and returned.

6.2.4 Returns and Destruction

Upon completion or termination of the trial, all used IMP containers, unused IMPs, and partially-used IMPs must be destroyed at the trial site(s).

The assigned trial monitor will facilitate the destruction of used IMP containers, unused IMPs, and partially-used IMPs. The trial site(s) may utilize qualified third-party vendors for IMP destruction. A certificate of destruction should be filed within the IMP accountability.

6.2.5 Reporting of Product Quality Complaints

A Product Quality Complaint (PQC) is any written, electronic, or oral communication provided by a healthcare professional, clinical trial subject, medical representative, regulatory agency, partner, or other third party that alleges deficiencies related to the identity, quality, durability, reliability, safety, or performance of a medical device or medicinal product after it is released for distribution.

Examples include, but are not limited to, communications involving:

- Failure of a product to meet any of its specifications.
- Packaging defects (eg, damaged, dirty, crushed, missing product or component, incorrect or missing labeling).
- Product defects (eg, odor, chipped, broken, damaged, crushed, embossing illegible, under-filled bottle, over-filled bottle, empty bottle, no safety seal).
- Loss or theft of product.

6.2.5.1 Eliciting and Reporting Product Quality Complaints

The investigator or designee must record each PQC identified through any means from the receipt of the sponsor IMP (ie, DLM or OPC-167832) from the sponsor or sponsor's designee, through and including reconciliation and up to destruction, including subject dosing. The investigator or designee must notify the sponsor (or sponsor's designee) by email within 24 hours of becoming aware of the PQC according to the procedure outlined below.

- Send PQC reporting information to the Otsuka Pharmaceutical Development & Commercialization, Inc. IMP complaints mailbox email: IMP-PQC@otsuka-us.com.

Also indicate whether or not the complaint sample is available for return.

Identification of a PQC by the subject should be reported to the site investigator, who should then follow the reporting mechanism above.

The investigator should report PQCs in accordance with respective manufacturing authorization holders for nonOtsuka products (per package label).

6.2.5.2 Information Required for Reporting Purposes

- Description of complaint
- Reporter ID (eg, investigator, site, etc)
- Reporter contact information (eg, address, phone number, email address)
- Subject number
- Clinical site number
- ID of material (product/compound name, lot/batch number, shipment number, expiry date)
- Clinical protocol reference (number and/or trial name)
- Dosage form/strength (if known)
- Pictures of complaint sample (if available)
- Availability of complaint sample for return

6.2.5.3 Return Process

Indicate during the report of the PQC if the complaint sample is available for return. If the complaint sample is available for return, the sponsor will provide return instructions, when applicable.

If the complaint sample is available but not at the clinical site, please instruct the subject to bring the complaint sample to their next site visit.

It must be documented in the site accountability record that the complaint sample has been forwarded to the sponsor for complaint investigation.

6.2.5.4 Assessment/Evaluation

Assessment and evaluation of PQCs will be handled by the sponsor. The investigator should report PQCs in accordance with the respective manufacturing authorization holders for nonOtsuka products (per package label).

6.3 Measures to Minimize/Avoid Bias

This is an open-label trial; however, the microbiology laboratory will be blinded to treatment assignments. The Miettinen and Nurminen confidence interval (CI) of common risk difference stratified by HIV status and cavitation status prior to randomization will be used to reduce bias in subject allocation to treatment groups.[7]

6.4 Subject Compliance

The time and dose of IMP and RHEZ administration during the trial will be recorded on the eCRF. Information regarding any inappropriately administered doses will also be documented on the eCRF. A Medication Event Reminder Monitor System will also be used to measure compliance.

6.5 Concomitant Medications or Therapies

The investigator will record all medications (including prescription medications, over-the-counter medications, herbal remedies, etc) and therapies taken by the subject from 30 days prior to signing of informed consent through the end of the evaluation period (defined as the time period during which subjects are evaluated for primary and/or secondary objectives) in the eCRF. The investigator will also record all medications and therapies taken by the subject for treatment of an AE or which caused an AE until the end of the trial (defined as the last date of contact or date of final contact attempt) in the eCRF.

For concomitant medications, the following will be recorded in the eCRF: medication, indication, dose, frequency, route, start date and end date.

6.5.1 Prohibited Medications or Therapies

Quinolones should not be used within 3 months prior to the screening visit and during the screening period. Subjects should not be taking or start antiretroviral therapy within 2 weeks of the completion of the trial.

The selected cytochrome P450 (CYP)3A4 inhibitors and inducers presented in Table 6.5.1-1 and Table 6.5.1-2, respectively should not be used from Day −2 until the end of treatment.

Table 6.5.1-1 List of Selected Cytochrome P450 3A4 Inhibitors Prohibited During the Trial

**Potent CYP3A4 Inhibitors:**	
Viekira PAK2	Danoprevir
Indinavir	Elvitegravir
Tipranavir	Saquinavir
Ritonavir	Lopinavir
Cobicistat	Itraconazole
Ketoconazole	Voriconazole
Troleandomycin	Mibefradil
Telaprevir	Clarithromycin
Posaconazole	Nelfinavir
Telithromycin	Nefazodone
Grapefruit juice	Idelalisib
Conivaptan	Boceprevir
**Moderate CYP3A Inhibitors:**	
Erythromycin	Faldaprevir
Fluconazole	Imatinib
Atazanavir	Verapamil
Diltiazem	Netupitant
Darunavir	Nilotinib
Dronedarone	Tofisopam
Crizotinib	Cyclosporine
Atazanavir	Ciprofloxacin
Aprepitant	Isavuconazole
Casopitant	Cimetidine
Amprenavir	

Note: Please communicate with the sponsor's medical monitor if the subject needs to receive any medication (other than IMP or RHEZ) that is not on this list.

Table 6.5.1-2 List of Selected Cytochrome P450 3A4 Inducers Prohibited During the Trial

**Potent CYP3A4 Inducers:**	
Carbamazepine	Enzalutamide
Mitotane	Phenytoin
St. John's wort	
**Moderate CYP3A Inducers:**	
Bosentan	Efavirenz
Etravirine	Modafinil

Note: Please communicate with the sponsor's medical monitor if the subject needs to receive any medication (other than IMP or RHEZ) that is not on this list.

6.5.2 Permitted Medications or Therapies

Not applicable.

6.5.3 Rescue Medications

Rescue treatment consisting of the standard of care treatment, ie, RHEZ, will be provided in the following circumstances:

1) If a subject is sputum culture positive for *M tuberculosis* at the end of treatment,
2) If a subject has a microbiologic relapse subsequent to the end of treatment, or
3) If, in the opinion of the investigator, a subject is clinically deteriorating, based on any combination of signs, symptoms, or chest radiograph data.

Note: Standardized rescue treatment consisting of RHEZ is to be used for Arm 1, Arm 2, and Arm 3 not responding to therapy. However, subjects in Arm 4 will receive individualized care according to local guidelines. All subjects who require rescue treatment will be considered treatment failures for the purposes of the trial.

6.6 Intervention After the End of the Trial

Not applicable.

7 Stopping Rules, Withdrawal Criteria, and Procedures

7.1 Entire Trial or Treatment

If the sponsor terminates or suspends the trial for any reason, prompt notification will be given to investigators, Independent Ethics Committee (IECs)/Institutional Review Boards (IRBs), and regulatory authorities in accordance with regulatory requirements.

7.2 Individual Site

Individual trial site participation may be discontinued by the sponsor, the investigator, or the IEC if judged to be necessary for medical, safety, regulatory, ethical or other reasons consistent with applicable laws, regulations, and Good Clinical Practice (GCP). The investigator will notify the sponsor promptly if the trial is terminated by the investigator or the IEC at the site.

7.3 Individual Subject Discontinuation

After the first dose of IMP or RHEZ on Day 1, a subject may stop treatment permanently for a variety of reasons. Treatment discontinuations may be initiated by a subject or may become medically necessary due to AEs, required treatment with a disallowed medication or therapy, or other reasons, as determined by the investigator. If a subject discontinues treatment, their participation in the trial will be discontinued. Discontinued subjects

should be encouraged to complete all early termination (ET) and follow-up assessments with ET assessments conducted as soon as possible after the subject is withdrawn.

If during the trial the sponsor finds any unacceptable safety concerns on the safety of a high dose DLM + BDQ + OPC-167832 group, the enrollment of new subjects in the high dose group can be discontinued or reduced, and allocated to the remaining sample size for other dose level(s).

7.3.1 Treatment Interruption

Not applicable.

7.3.2 Treatment Discontinuation

After treatment assignment, a subject may stop treatment permanently for a variety of reasons. Treatment discontinuations may be initiated by a subject or may become medically necessary due to AEs, required treatment with a disallowed medication or therapy, or other issues, as determined by the investigator. However, each investigator must comprehensively review the circumstances and offer the subject options for continued treatment to the degree possible as described in Section 7.3.5. Discontinued subjects should be encouraged to complete all ET and follow up assessments with ET assessments conducted as soon as possible after the subject is withdrawn.

7.3.3 Documenting Reasons for Treatment Interruption or Discontinuation

A subject may discontinue IMP for the reasons listed below:

- Adverse event
  - Subject decides to discontinue because of annoyance or discomfort due to a nonserious AE which is not otherwise determined to be an undue hazard
  - Continuing IMP places the subject at undue risk as determined by the investigator (eg, a safety concern that is possibly, probably, or likely related to IMP)
    - SAE
    - Other potentially IMP-related safety concerns or AEs
- Death
- Disease relapse
- Failure to meet randomization criteria
- Lack of efficacy
- Lost to follow-up
- Noncompliance with IMP
- Physician decision

- Pregnancy (see Section 10.3)
- Progressive disease
- Protocol deviation
- Protocol violation
- Protocol-specific withdrawal criterion met
- Randomized by mistake
- Randomized by mistake with trial treatment
- Site terminated by sponsor
- Trial terminated by sponsor
- Withdrawal by subject

If the subject discontinues IMP due to an AE, the investigator, or other trial personnel, will make every effort to follow the event until it has resolved or stabilized. Follow-up procedures in Section 7.3.2 must be followed.

7.3.4 Withdrawal of Consent

Each subject has the right to withdraw their consent from further participation in the trial at any time without prejudice. Subjects can withdraw consent for use of data which has not previously been anonymously transferred into trial data sets collected as part of the trial and can only withdraw consent for future participation. The investigator can also discontinue a subject's participation in the trial at any time if medically necessary. Unless the subject provides their written withdrawal of consent or there is other written documentation by the investigator confirming the subject's verbal intent to completely withdraw from the trial, subjects should be followed for all protocol-specified evaluations and assessments, if possible.

Complete withdrawal of consent requires a subject's refusal of ALL of the following methods of follow-up:

- Participation in all follow-up procedures specified in the protocol (whether in-clinic, by telephone, or by a home visit).
- Participation in a subset of protocol specified follow-up procedures (by a frequency schedule and method, as agreed by subject and trial site staff).
- Contact of the subject by trial personnel, even if only by telephone, to assess current medical condition, and obtain necessary medical or laboratory reports relevant to the trial's objectives.
- Contact of alternative person(s) who have been designated in source records as being available to discuss the subject's medical condition, even if only by telephone, mail, or email (eg, family, spouse, partner, legal representative, friend, neighbor, or physician).

- Access to medical information from alternative sources (eg, hospital/clinic medical records, referring doctor's notes, public records, dialysis, transplantation or vital registries, social media sources).

Withdrawal of consent is a critical trial event and, therefore, should be approached with the same degree of importance and care as is used in initially obtaining informed consent. The reasons for a subject's intended withdrawal need to be completely understood, documented, and managed to protect the rights of the subject and the integrity of the trial. A subject may initially express their desire to discontinue IMP administration, which is not equivalent to a complete withdrawal of consent for further participation (see Section 7.3.2). A subject may, however, indicate that further trial participation is creating a burden on their work, school, or social schedule. Therefore, the investigator should follow the procedures outlined in Section 7.3.3 to determine if the subject can continue participation in the trial if modifications to his/her treatment and/or schedule of assessments can be accommodated. Only subjects who withdraw their permission for all of the above methods of follow-up are considered to have completely withdrawn their consent to participate in the trial.

[redacted]

7.3.5 Procedures to Encourage Continued Trial Participation

In all cases of impending IMP discontinuation or consent withdrawal, investigators will be instructed to meet and discuss (without undue coercion) with the subject their options of continuing in the trial, preferably on therapy. The investigator should ensure understanding and documentation of the reasons for the subject's desire to withdraw consent. Refer to Section 6.5.3 regarding rescue medications.

7.4 Definition of Subjects Lost to Follow-up

Subjects who cannot be contacted on or before the trial visits at Weeks 17 (for Arm 1, Arm 2, and Arm 3) and Week 26 (for Arm 4) during the treatment period, who do not have a known reason for discontinuation (eg, withdrew consent or AE), and for whom a survival status at the end of the trial cannot be determined will be classified as "lost to follow-up". Survival status can be determined from a variety of sources, either by obtaining acceptable documentation for death (ie, death certificate, medical records, public records, statement by a family member or primary care physician) or acceptable documentation for life (ie, direct contact with the subject, medical records, successful telephone contact with the subject, statement by a family member or primary care physician, or public records).

The site will make 3 documented attempts to contact the subject by telephone and in the event the site is unable to reach the subject by telephone, the site will attempt to contact the subject via certified mail or an alternative similar method, where appropriate, before assigning a “lost to follow-up” status.

If the subject was classified as “lost to follow-up”, “Were you able to contact the subject?”, “Date of contact/Date of final contact attempt” and “Contact method” will be recorded in the eCRF.

8 Trial Procedures

The assessments to be conducted during the trial are summarized in Table 1.3-1.

8.1 Efficacy Assessments

8.1.1 Sputum Sampling and Microbiology Laboratory Assessments

Refer to Section 10.5 (Appendix 5) for a detailed collection schedule of sputum samples for each treatment arm.

8.1.1.1 Sputum for Eligibility Assessments

A sputum sample will be collected spontaneously (ie, not induced) by the site staff during the screening period (Day -14 to Day -2) and baseline Day -1 to confirm the presence of *M tuberculosis* that is susceptible to isoniazid and rifampin. The specimen will be tested by a molecular assay that can rapidly detect drug resistance to these 2 drugs.

8.1.1.2 Sputum for Efficacy Assessments

Sputum samples will be collected as per Table 1.3-1 for processing.

Up to 3 sputum samples will be tested at each scheduled visit except for Day −1 and Day 1 (on these days, site staff will collect 1 sputum sample for culture, and 1 sputum for ribosomal ribonucleic acid synthesis ratio [RS ratio] on each day). The Day 1 sputum will be collected prior to the first dose of IMP. For subsequent visits, specimen number 1 will be collected by the subject early in the morning, preferably prior to the morning meal. Specimen number 2 will be collected at the site by the trial site staff. Sputum specimens 1 and 2 will be processed using the Mycobacteria Growth Indicator Tube® (MGIT) liquid culture system. Culture growth will be confirmed as *M tuberculosis* complex using a rapid commercial test (immunochromatographic or molecular). An immunoassay to measure sputum LAM concentration will also be performed at each visit.

If a subject (in Arms, 1, 2, and 3) is unable (RHEZ) to spontaneously expectorate sputum number 2 during site collection, an attempt will be made to induce sputum production by aerosol inhalation. Trial sites will use their local procedure for sputum induction. If

neither sputum number 1 nor sputum number 2 is collected on the visit date, subjects should return to the site to attempt to produce a sample(s) once over the next 72 hours, if necessary. Sputum samples will be defined as unobtainable if no sputum can be obtained during this 72-hour period after an attempt at sputum induction.

If the subject is able to spontaneously produce a subsequent specimen during the visit, specimen number 3 will be collected directly into ribonucleic acid preservation media and frozen for batch measuring of the RS ratio. If this sputum cannot be collected, this will not be considered a protocol deviation.

Refer to the trial operations manual for further guidance on proper collection of sputum specimens at each scheduled visit.

Susceptibility testing for anti-TB medications will be performed as necessary. Susceptibility testing and/or MIC of DLM, BDQ, and OPC-167832 will also be performed on positive *M tuberculosis* isolates from one of Day -1 or Day 1 cultures (if both are positive), and at later time points as necessary.

To determine if the subject has relapsed or has a new infection of *M tuberculosis*, genotyping will be performed on the baseline culture (Day −1 and/or Day 1, or Week 1, if necessary) and the first of any subsequent positive culture during the post treatment follow-up period to determine if the subject has relapsed or has new infection of *M tuberculosis*. In addition, the sponsor may request genotyping as needed.

Positive *M tuberculosis* isolates will be stored long-term for confirmatory testing and potential future characterization of the bacteria, as necessary.

8.1.2 Signs and Symptoms of Tuberculosis

An aggregate of clinical signs and symptoms of pulmonary TB will be assessed at screening and throughout the trial. Signs and symptoms will be assessed by the investigator (cough, hemoptysis, dyspnea, chest pain, loss of appetite, feeling feverish, and night sweats); from physical examination (weight, body mass index, body temperature, respiratory rate and heart rate); from clinical laboratory tests (hemoglobin, hematocrit, c-reactive protein, and serum albumin); and from chest radiograph. A chest radiograph must be taken during the screening period, and an official interpretation must be available, and the assessment of its compatibility with pulmonary TB must be made, prior to randomization. An end of treatment chest radiograph will be taken at Week 17 (for subjects in Arms 1, 2, and 3) or Week 26 (for subjects in Arm 4) and at Month 12.

8.2 Pharmacokinetic Assessments

Sparse sampling will be performed; population PK analysis will be used to analyze PK characteristics of DLM and metabolite DM-6705, BDQ and its metabolite M2, and OPC-167832, as applicable.

For subjects randomized to Arm 1, Arm 2, and Arm 3, PK samples will be taken on Day 1 (predose to DLM and postdose), and then Week 2 (predose), Week 4 (predose), Week 8 (predose and postdose), Week 13 (predose), and Week 17/ET (predose and postdose) (9 samples per subject). One or 2 blood samples for OPC-167832, DLM and its metabolite DM-6705, and BDQ and its metabolite M2 will be taken from each subject at each PK sampling timepoint. (The predose blood draw should be drawn 15 minutes prior to the daily dose of OPC-167832/DLM/BDQ. The postdose blood draw should be done at a flexible time approximately 2-8 hours postdose). If a PK blood sample cannot be drawn at the designated time, a window of ± 15 minutes for each blood draw is acceptable, with the exact time recorded.

Subjects randomized to treatment Arm 4 will not have PK sampling.

8.2.1 Pharmacokinetic Blood/Serum/Plasma Samples

Blood samples will be collected to determine the concentrations of DLM and its metabolite DM-6705, and for OPC 167832. Blood samples also will be collected to determine the concentrations of BDQ and its metabolite M2.

The concentrations of additional metabolites that are not identified in the protocol may also be determined, if needed. In addition, PK samples may be used for the investigation of a bioanalytical method, if needed.

Blood samples for PK analysis will be collected at the time points as shown in Table 1.3-1 (schedule of assessments). These samples should be collected within 15 minutes (ie, ± 15 minutes) of the nominal sampling window. Predose samples should be collected within 15 minutes before IMP administration.

When vital signs or ECGs are scheduled at the same nominal time as PK sample collections, vital signs should be measured and ECGs should be performed before PK samples are collected.

The actual date and time of the PK sample collection will be recorded in the eCRF.

After processing into plasma, aliquots will be placed into appropriately labeled tubes and will be placed in a freezer set at −70°C.

All plasma samples will be shipped to the central repository. Additional information will be provided in the Operations Manual.

8.3 Pharmacodynamic Assessments

8.3.1 Pharmacodynamic Laboratory Tests

Sputum culture using MGIT: culture conversion and time-to-detection.

8.4 Pharmacogenomic Assessments

8.4.1 Pharmacogenomic Samples

Not applicable.

8.5 Biomarker Assessments

Sputum LAM concentration will be determined using an immunoassay to evaluate the correlation of LAM to culture results.

Additional biomarkers include positron emission tomography/computerized axial tomography (PET/CT) imaging, RS ratio, and transcriptomic signatures from whole blood. The type of PET/CT in this trial is positron emission tomography with 2-[^{18}F]-fluoro-2-deoxy-D-glucose.

Whole blood samples to longitudinally assess transcriptomic signatures previously associated with TB cure will be collected using Paxgene ribonucleic acid tubes at the time points described in the schedule of assessments (Table 1.3-1). Genomic ribonucleic acid will be extracted from a whole blood sample and stored until shipped to the designated laboratory for analysis. Additional information will be provided in the Laboratory Manual.

[redacted]

[redacted]

[redacted] diseases and/or their therapeutic treatments.

[redacted]

8.7 Safety Assessments

Details pertaining to the definitions, collection, reporting, and follow-up of AEs are described in Section 8.8.

8.7.1 Clinical Laboratory Assessments

Clinical laboratory samples will be collected at the time points described in the schedule of assessments (Table 1.3-1) to perform the clinical laboratory assessments described in Section 10.2. Refer to Section 8.8.5 for potential serious hepatotoxicity. The Division of AIDS (DAIDS) table criteria will be used for identifying laboratory values of potential clinical relevance.[8] The total volume of blood to be collected during the trial will be documented in the ICF form.

8.7.2 Physical Examination

Either a complete or targeted physical examination will be performed at the time points described in the schedule of assessments (Table 1.3-1). Visual assessments to be completed during screening will consist of Snellen and Ishihara testing and will be repeated during the trial if the investigator suspects abnormalities. Neurological examination and psychiatric assessment to be performed with each physical examination. Please refer to the trial operations manual for details on the neurological and psychiatric assessments and the targeted physical examination.

8.7.3 Vital Signs

Vital signs include systolic and diastolic blood pressure (mmHg), heart rate (bpm), respiratory rate (rpm), body temperature (C), height (cm) (only at screening) and weight. Vital signs will be obtained prior to PK blood draws and ECGs at the nominal time points, where applicable. At screening, blood pressure and heart rate will be taken with the subject in the supine (performed first) and sitting position after remaining for ≥ 3 minutes in each position and temperature and respiratory rate will be taken with the

subject in the supine position. At all other time points, vital signs will be obtained after the subject has been at rest in the supine position for at least 1 minute.

Vital signs will be collected at the trial visits described in the schedule of assessments (Table 1.3-1). Subjects should be monitored for potentially clinically significant vital signs values. The DAIDS table criteria will be used for identifying vital signs of potential clinical relevance.[8]

8.7.4 Electrocardiogram

One standard 12-lead ECG will be performed during screening, plus 3 ECGs at baseline (Day −1) spaced 5 to 10 minutes apart, and 3 ECGs at all subsequent visits during the treatment and follow up periods. Where multiple ECGs are required in a visit, the subject should be recumbent for 10 minutes prior, if possible, and ECGs should be collected 5 to 10 minutes apart after the dose of IMP and prior to PK blood draws.

Electrocardiograms will be performed at the trial visits described in the schedule of assessments (Table 1.3-1). Subjects should be monitored for potentially clinically significant ECG results. The DAIDS table criteria will be used for identifying ECGs of potential clinical relevance.[8]

8.7.5 Suicidality Monitoring

Not applicable.

8.7.6 Other Safety Variables

A PET/CT scan will be performed at the trial visits described in the schedule of assessments (Table 1.3-1). If the PET/CT scan is unavailable, the subject is permitted to continue in the trial.

8.8 Adverse Events

8.8.1 Definitions

An AE is defined as any untoward medical occurrence in a clinical trial subject administered an IMP or trial treatment and which does not necessarily have a causal relationship with this treatment. Adverse events would not include information recorded as medical history at screening for preplanned procedures for which the underlying condition was known and no worsening occurred. An adverse reaction is any untoward and unintended response to an IMP related to any dose administered.

A suspected adverse reaction is any AE for which there is a reasonable possibility that the IMP caused the AE. For the purpose of Investigational New Drug (IND) safety reporting,

"reasonable possibility" means there is evidence to suggest a causal relationship between the IMP or trial treatment and the AE. Suspected adverse reaction implies a lesser degree of certainty about causality.

TEAEs are defined as AEs with an onset date on or after the start of treatment. In more detail, TEAEs are all AEs which started after the start of IMP treatment; or if the event was continuous from baseline and was worsening, serious, IMP related, or resulted in death, discontinuation, interruption, or reduction of IMP.

<u>An SAE</u> includes any event that results in any of the following outcomes:

- Death
- Life-threatening; ie, the subject was, in the opinion of the investigator, at immediate risk of death from the event as it occurred. It does not include an event that, had it occurred in a more severe form, might have caused death.
- Persistent or significant incapacity/disability or substantial disruption of the ability to conduct normal life functions.
- Requires inpatient hospitalization or prolongs hospitalization.
  - Hospitalization itself should not be reported as an SAE; whenever possible the reason for the hospitalization should be reported.
  - Hospitalizations or prolonged hospitalizations for social admissions (ie, those required for reasons of convenience or other nonmedical need) are not considered SAEs.
  - Prescheduled hospitalization to address a condition that has existed prior to the signing of the ICF should not be considered an SAE.
- Congenital anomaly/birth defect.
- Other medically significant events that, based upon appropriate medical judgment, may jeopardize the subject and may require medical or surgical intervention to prevent one of the outcomes listed above; eg, allergic bronchospasm requiring intensive treatment in an emergency room or home, blood dyscrasias or convulsions that do not result in hospitalization, or the development of drug dependency or drug abuse.

<u>Nonserious AEs</u> are all AEs that do not meet the criteria for a "serious" AE.

<u>Adverse Events of Special Interest (AESIs)</u>: A noteworthy event for the particular product/IMP or class of products that a sponsor may wish to monitor carefully. All AESIs are to be reported as immediately reportable event (IREs). No AESIs have been identified for the IMP to be administered during this trial. There are no AESIs in this trial.

<u>Immediately Reportable Event (IRE):</u>

- Any SAE.
- Any AE related to occupational exposure.

- Potential serious hepatotoxicity (see Section 8.8.5).
- Pregnancies are also defined as IREs. Although normal pregnancy is not an AE, it will mandate IMP discontinuation and must be reported on an IRE form and the Clinical Trial Pregnancy and Breastfeeding Form(s) to the sponsor. This includes pregnancy of the subject or the partner of the subject. Pregnancy will only be documented on the AE eCRF if the pregnancy occurs in a female subject and there is an abnormality or complication.

Clinical Laboratory Test Value Changes: It is the investigator's responsibility to review the results of laboratory tests for each individual subject as they become available. This review will be documented by the investigator's dated signature on the laboratory report. The investigator may repeat the laboratory test or request additional tests to verify the results of the original laboratory tests. If this laboratory value is considered medically relevant (ie, clinically significant) by the investigator (subject is symptomatic, requiring corrective treatment or further evaluation), or if the laboratory value leads to discontinuation, and/or fulfills a seriousness criterion, this is considered an AE.

Severity: Adverse events will be graded on a 3-point scale and reported as indicated in the eCRF. The severity of an adverse experience is defined as follows:

**1 = Mild:**	Discomfort noticed, but no disruption to daily activity.
**2 = Moderate:**	Discomfort sufficient to reduce or affect normal daily activity.
**3 = Severe:**	Inability to work or perform normal daily activity.

Note: In addition, all AEs will be graded on a 5-point scale according to the "DAIDS Table for Grading the Severity of Adult and Pediatric AEs".[8]

IMP Causality: Assessment of causal relationship of an AE to the use of the IMP is defined as follows:

**Related**:	There is a reasonable possibility of a temporal and causal relationship between the IMP and the AE.
**Not Related**:	There is no temporal or causal relationship between the IMP and the AE.

8.8.2 Eliciting and Reporting Adverse Events

The investigator will regularly assess subjects for the occurrence of AEs. To avoid bias in eliciting AEs, subjects should be asked the nonleading question: "How have you felt since your last visit?" All AEs (serious and nonserious) reported by the subject must be recorded on the source documents and eCRF provided by the sponsor. Adverse event collection will begin after a subject signs the ICF, and will continue until the follow up visit 12 months postrandomization. All AEs must be reported after subject informed

consent has been obtained, including screening failures due to AEs, irrespective of IMP administration.

Medical terminology should be used for AE reporting. Adverse events should be reported as a single unifying diagnosis whenever possible or, in the absence of a unifying diagnosis, as individual signs or symptoms.

Exacerbation or disease progression should be reported as an AE only if there are unusual or severe clinical features that were not present, or experienced earlier, or not expected based on the course of the condition.

In addition, the sponsor must be notified immediately by telephone, fax, or email of any IREs according to the procedure outlined below, in Section 8.8.3. Special attention should be paid to recording hospitalization and concomitant medications.

The adverse event, start date, end date, seriousness, severity, relationship to trial treatment (IMP causality), action taken with trial treatment, and outcome will be recorded on the source documents and in the eCRF.

8.8.3 Immediately Reportable Events

The investigator must immediately report (within 24 hours), using an IRE form, after he/she or site personnel become aware of any IRE (SAE, AE related to occupational exposure, potential serious hepatotoxicity, or confirmed pregnancy), by telephone, fax, or email to the sponsor or designee using the contact information on the cover page of this protocol (please note that the IRE form is NOT the AE eCRF). Subject confidentiality must be protected and contact information such as name, address, phone number or any other protected health information as determined by applicable local regulation must be redacted when forwarding Safety Information and supporting documentation. Details regarding the follow-up of IREs are included in Section 8.8.7.2.

8.8.4 Adverse Events of Special Interest

Not applicable.

8.8.5 Potential Serious Hepatotoxicity

For a subject who experiences an elevation in AST or ALT that is ≥ 3 times the upper limit of normal (ULN), a total bilirubin level should also be evaluated. If the total bilirubin is ≥ 2 times the ULN, complete an IRE form with all values listed and also report as an AE in the eCRF.

8.8.6 Procedure for Breaking the Blind

Except for the microbiology laboratory being blinded to the treatment assignment of each subject, this trial does not use blinding procedures.

8.8.7 Follow-up of Adverse Events

8.8.7.1 Follow-up of Nonserious Adverse Events

Nonserious AEs that are identified at any time during the trial must be recorded on the AE eCRF with the current status (ongoing or resolved/recovered) noted. All nonserious events (that are not IREs) that are ongoing at the last scheduled contact will be recorded as ongoing in the eCRF. For any AE having been identified throughout the trial, during analysis, additional relevant medical history information may be requested by the sponsor to further ascertain causality (including, but not limited to, information such as risk-related behavior, family history, and occupation).

8.8.7.2 Follow-up of Immediately Reportable Events

This trial requires that subjects be actively monitored for IREs up to 12 months after randomization, or the last scheduled visit.

Immediately reportable events that are **identified or ongoing at the last scheduled contact** must be recorded as such on the AE eCRF page and the IRE form. If updated information (eg, resolved status) on IRE status becomes available after a subject's last scheduled contact (up to last in-clinic visit for the entire trial), this must be reported to the sponsor and recorded on the AE eCRF page and the IRE form, according to the appropriate reporting procedures described in Section 8.8.3.

It is expected that the investigator will provide or arrange appropriate supportive care for the subject and will provide prompt updates on the subject's status to the sponsor. The investigator will follow IREs until the events are:

- Resolved,
- Recovered/Resolved with sequelae
- The subject is lost to follow-up, or
- Has died.

Resolution means that the subject has returned to the baseline state of health and stabilized means that the investigator does not expect any further improvement or worsening of the subject's condition. The investigator will continue to report any significant follow-up information to the sponsor up to the point the event has resolved or stabilized, or the subject is lost to follow-up, or has died.

Refer to Section 10.3 for additional information regarding the follow-up period for subjects that become pregnant or for pregnant partners of male subjects.

8.8.7.3 Follow-up and Reporting of Immediately Reportable Events Occurring After Last Scheduled Contact

Any new IREs reported to the investigator which occur after the last scheduled contact and are determined by the investigator to be reasonably associated with the use of the IMP, should be reported to the sponsor according to the procedures outlined in Section 8.8.3.This may include IREs that are captured on follow-up telephone contact or at any other time point after the defined trial period and continue to report any significant follow-up information to the sponsor until the events are resolved or stabilized, or the subject is lost to follow-up or has died.

8.9 Treatment of Overdose

For treatment of overdose, please refer to the IB for DLM or OPC-167832, or the package insert for BDQ and RHEZ.

8.10 Subject Assessment Recording

Not applicable.

8.11 Other Assessments

Not applicable.

9 Statistical Considerations

9.1 Sample Size

It is planned to enroll enough subjects so there will be 20 subjects in DLM + BDQ + 90 mg OPC-167832, 40 subjects each in DLM + BDQ + 30 mg OPC-167832, and DLM + BDQ + 10 mg OPC-167832, and 20 subjects in RHEZ group. This is a pilot trial and exploratory in nature. Thus 80% CIs between proportion differences will be calculated to compare with noninferiority margin. Assuming that the sputum culture conversion (SCC) rate for the primary endpoint is 93% for the RHEZ group, and assuming the SCC rate of the investigational arms is identical to the RHEZ group, with a noninferiority margin of 12%, sample size of 120 subjects (100 for pooled DLM + BDQ + OPC-167832 and 20 for RHEZ) will have about 84% power to reject the null hypothesis with a one-sided alpha level of 0.1, ie, the lower limit of the two-sided 80% CI of the difference in proportions of sputum conversion between the pooled investigational arms and RHEZ is larger than -0.12. The Miettinen and Nurminen method to construct 80% CI was used in the sample size calculation.[7] Assuming 11% randomized

subjects will not demonstrate at least 1 positive culture result by Week 1 and susceptibility to rifampin and isoniazid, approximately 135 subjects will be randomized in 1:2:2:1 ratio to obtain 120 subjects in the modified intent-to-treat (mITT) analysis set.

9.2 Datasets for Analysis

The PK analysis set will include all subjects with adequate data for the population PK analysis.

The safety analysis set includes all subjects who receive any dose of IMP or RHEZ. Safety analysis will be conducted based on the safety analysis set.

The mITT analysis set includes all randomized subjects who take at least 1 dose of IMP or RHEZ, have at least 1 postbaseline culture result, and demonstrate positive culture results by Week 1 and susceptibility to rifampin and isoniazid (from the screening specimen).

9.3 Handling of Missing Data for Primary and Secondary Endpoint Analysis

Missing data caused by subject discontinuation will be handled by a composite variable strategy according to International Council for Harmonisation (ICH) E9 (R1) Addendum.[9] Subjects who discontinue early from the trial during the treatment period without an SCC outcome will be considered as no SCC.

9.4 Statistical Analyses

9.4.1 Efficacy Analyses

9.4.1.1 Primary Efficacy Endpoint Analysis

The primary efficacy endpoint of the trial is the proportion of subjects who have SCC and no subsequent positive culture by the end of treatment. Sputum culture conversion occurs when a subject has 2 visits of at least 28 days apart (±4 days) with sputum cultures negative and without a positive sputum culture result in between.

The 3 DLM + BDQ + OPC-167832 arms will be pooled, and noninferiority with RHEZ will be established if the lower limit of 80% CI for the difference of proportion of subjects with SCC between the pooled DLM + BDQ + OPC-167832 group is larger than -0.12. The Miettinen and Nurminen CI of common risk difference stratified by HIV status and cavitation status prior to randomization will be used.[7] The 80% CI of the proportion difference between each dose level of DLM + BDQ + OPC-167832 arm and RHEZ will be provided. In addition, Cochran-Armitage test will be used to detect trend in proportion of SCC with increasing OPC-167832 dose.

9.4.1.2 Secondary Efficacy Endpoint Analysis

The secondary efficacy endpoints of this protocol are:

- The proportion of subjects in each arm who achieve SCC in MGIT by 8 weeks of treatment.
- Time to detection of MGIT cultures.
- The proportion of subjects in each arm who convert sputum LAM to negative by 8 weeks of treatment and by end of treatment.
- Proportion of subjects who develop drug resistance.
- Time to SCC.

For a proportion endpoint, the same analysis methodology specified for the primary endpoint will be used. A 12% noninferiority margin will be used if the goal is to establish noninferiority.

The Kaplan-Meier curve of time to SCC will be provided for each group and relationship between time to SCC and PK parameters will be studied.

To identify the optimal dose of OPC-167832, a PK/PD analysis will be performed using AUC as the PK parameter, and bacterial load (MGIT time-to-detection) or proportion of culture conversion as the pharmacodynamic parameter.

9.4.1.3 Control of Experiment-wise Type 1 Error

Not applicable to this trial.

9.4.1.4 Other Efficacy Endpoint Analysis

Exploratory efficacy endpoints are:

- Longitudinally assess PET/CT imaging response over the course of treatment.
- Longitudinally evaluate the RS ratio decline in sputum.
- Longitudinally assess whole blood transcriptomic signatures previously associated with TB cure from serum.
- The proportion of subjects with favorable outcome in each experimental arm as compared to the RHEZ standard treatment arm at 6 months post end of treatment and at 12 months postrandomization.

Detailed analysis methods for these exploratory efficacy endpoints will be provided in the statistical analysis plan (SAP).

9.4.2 Safety Analysis

Safety endpoints will be summarized using descriptive statistics (number, median, mean, standard deviation, minimum, maximum) for continuous values, and by frequency and

percentage for discrete variables. Percentage and frequency of AEs and clinical laboratory test results by grades will be analyzed.

9.4.2.1 Adverse Events

All AEs will be coded by system organ class and Medical Dictionary for Regulatory Activities preferred term. The incidence of the following events will be summarized by treatment group:

- TEAEs
- TEAEs by severity
- TEAEs potentially causally related to the IMP or trial medication
- TEAEs with an outcome of death or trial medication
- Serious TEAEs
- TEAEs leading to discontinuation of the IMP or trial medication

In addition, TEAEs by DAIDS grades will be summarized.

9.4.2.2 Clinical Laboratory Data

For clinical laboratory tests data, baseline is defined as the last nonmissing value obtained at a scheduled visit on or before Day −1. If the Day −1 measurement is missing, the baseline value will be the last nonmissing measurement from the screening visit (the previous scheduled visit). Clinical laboratory test parameters and mean changes from baseline will be summarized by treatment. The incidence of potentially clinically significant changes and abnormalities in clinical laboratory test parameters will be summarized. Clinical laboratory test data will be presented in listings.

9.4.2.3 Physical Examination and Vital Signs Data

Physical examination data will be presented in listings.

Vital sign parameters and mean changes from baseline will be summarized by treatment arm. The incidence of potentially clinically significant changes and abnormalities in vital sign parameters will be summarized. Vital sign data will be presented in listings.

9.4.2.4 Electrocardiogram Data

Electrocardiogram measurements and mean changes from baseline will be summarized by treatment. The baseline ECG is defined as Day −1. If the measurement from Day −1 is missing, the screening ECG is taken as baseline. The incidence of potentially clinically significant changes and abnormalities in ECG evaluations will be summarized. Electrocardiogram data will be presented in listings.

9.4.3 Other Analyses

9.4.3.1 Analysis of Demographic and Baseline Characteristics

Summaries of demographics, TB medical history, TB disease severity, and sputum specimen results at baseline will be provided. Continuous variables will be summarized with mean, standard deviation, minimum, median, and maximum.

9.4.3.2 Pharmacokinetic Analysis

Plasma concentration of OPC-167832, DLM and its metabolite DM-6705, and BDQ and its metabolite M2 are planned to be utilized to perform population PK analyses, as applicable.

9.4.3.3 Pharmacodynamic Analysis

Not applicable.

9.4.3.4 Exposure-Response Analysis

Pharmacokinetics/PD and/or exposure-response analysis will be used to analyze the relationship of OPC-167832 plasma exposure alone or combined with DLM and BDQ with the efficacy and safety endpoints, as applicable. The efficacy endpoints including but not limited to LAM, proportion of subjects without SCC, time to detection and time to SCC may be explored.

9.4.3.5 Pharmacogenomic Analysis

Transcriptomic signatures previously associated with TB cure from serum will be longitudinally assessed. Details will be included in the approved SAP.

[redacted]

[redacted]

[redacted]

[redacted]

[redacted]

[redacted]

[redacted]

9.5 Interim Analysis and Adaptive Design

Interim PK/PD analyses will be performed after subjects have completed the end of treatment response time point (4 months of treatment in the OPC-167832 arm).

In addition, an early assessment of dose-response and toxicity may be conducted after the first 24 randomized subjects (ie, approximately 4:8:8:4 in each arm) complete the end of treatment response time point. If an OPC-167832 arm is dropped, subsequently enrolled subjects would then be allocated to the remaining OPC-167832 arms. Further details will be provided in the SAP.

9.5.1 Data Monitoring Committee

An independent Data Monitoring Committee (DMC) will be contracted by Otsuka to review safety, efficacy, and PK data during the conduct of the trial. The DMC will convene and operate based on an established charter. The DMC will be permitted to seek additional independent expertise, as needed. The primary responsibility of the DMC will be to monitor safety and to advise the sponsor of the clinical project regarding whether or not the safety concerns merit stopping the trial. Towards this objective the DMC will meet on a predetermined schedule to review safety and available efficacy and PK data.

10 Supporting Documentation and Operational Considerations

10.1 Appendix 1: Regulatory, Ethical, and Trial Oversight Considerations

10.1.1 Ethics and Responsibility

This trial must be conducted in compliance with the protocol, applicable ICH GCP guidance, international ethical principles derived from the Declaration of Helsinki and Council for International Organizations of Medical Science guidelines, and applicable local laws and regulations. Each trial site will seek approval/favorable opinion by an IRB or IEC according to regional requirements, and the investigator will provide that documentation to the sponsor. The IRB/IEC will evaluate the ethical, scientific, and medical appropriateness of the trial. Further, in preparing and handling the eCRF, IRE and any safety information, the investigator, subinvestigator, and their staff will take measures to ensure adequate care in protecting subject privacy. To this end, a subject ID will be used to identify each subject.

Financial aspects, subject insurance, and the publication policy for the trial will be documented in the agreement between the sponsor and the investigator.

10.1.2 Informed Consent

Informed consent will be freely obtained from all subjects (or their guardian or legally acceptable representative, as applicable for local laws). The ICF will be approved by the same IRB/IEC that approves this protocol.

Each ICF will comply with the ICH (International Council for Harmonisation) GCP Guidelines, and local regulatory requirements. The investigator will ensure that the sponsor reviews and authorizes any written site-specific ICF used in the trial before submission to the IRB/IEC.

Investigators may discuss trial availability and the possibility for entry with a potential subject without first obtaining consent. However, informed consent must be obtained and documented before initiation of any procedures that are performed solely for the purpose of determining eligibility for this trial, including withdrawal from current medication(s).

Potential subjects are free to refuse entry into the trial, or withdraw from the trial at any time, without justification, and there will be no consequences to their further care.

Once appropriate essential information has been provided and fully explained in layman's language to the subject by the investigator (or a qualified designee), and it has been documented that the subject has had the opportunity to ask questions and have those

questions answered, the IRB/IEC-approved paper ICF will be signed and dated by both the subject and the person obtaining consent (investigator or designee), as well as by any other parties required by the IRB/IEC. The subject will receive a copy of the signed ICF; the original shall be kept on file by the investigator. Subjects may be asked to sign additional ICFs if the protocol is amended and the changes to the protocol result in additional information that needs to be provided to the subjects, so that they can make a knowledgeable and voluntary decision on continued trial participation. Female partners of male subjects who become pregnant during the course of the trial may be asked to sign additional ICFs in order to collect additional information regarding the nonsubject partner and fetus.

[redacted]

10.1.3 Confidentiality

All information generated in this trial will be considered confidential and will not be disclosed to anyone not directly concerned with the trial without the sponsor's prior written permission. Subject confidentiality requirements of the region(s) where the trial is conducted will be met. However, authorized regulatory officials and sponsor personnel (or their representatives) may be allowed full access to inspect and copy the records, consistent with local requirements. All IMPs, subject bodily fluids, and/or other materials collected shall be used solely in accordance with this protocol, unless otherwise agreed to in writing by the sponsor.

Subjects will be identified only by unique subject ID in the eCRF. If further subject ID is required, subjects' full names may be made known to a regulatory agency or other authorized officials if necessary, subject to local regulations.

10.1.4 Quality Control and Quality Assurance

10.1.4.1 Monitoring

The sponsor has ethical, legal, and scientific obligations to follow this trial in accordance with established research principles, the applicable ICH GCP guidance, and applicable regulatory requirements and local laws. As part of a concerted effort to fulfill these obligations (maintain current personal knowledge of the progress of the trial), the sponsor's monitors will visit the site during the trial, as well as communicate frequently via telephone, email, and written communications. In addition, all investigators and trial site personnel will undergo initial and ongoing training for this particular trial, and this training will be clearly documented.

10.1.4.2 Auditing

The sponsor's Quality Assurance Unit (or representative) may conduct trial site audits. Audits will include, but are not limited to, IMP supply, presence of required documents, the informed consent process, site operations, delegation of authority and training, and a review of the eCRF with source documents, as applicable. The investigator will agree to cooperate and participate with audits.

Regulatory authorities may inspect the investigator site during or after the trial. The investigator will cooperate with such inspections and will contact the sponsor immediately if such an inspection occurs.

10.1.5 Protocol Deviations

In the event of a significant deviation from the protocol due to an emergency, accident, or mistake (eg, violation of informed consent process, IMP dispensing or subject dosing error, treatment assignment error, subject enrolled in violation of eligibility criteria or concomitant medication criteria), the investigator or designee will contact the sponsor or designee at the earliest possible time by telephone or via email. The investigator and sponsor (or designee) will come as quickly as possible to a joint decision regarding the subject's continuation in the trial. This decision will be documented by the investigator and the sponsor (or designee) and reviewed by the site monitor.

Any major protocol deviation will be recorded in the eCRF along with the start date and details of the deviation.

10.1.6 Records Management

10.1.6.1 Source Documents

Source documents are defined as the results of original observations and activities of a clinical investigation. Source documents will include but are not limited to medical records, electronic data, logs, and recorded data from automated instruments or applications. All source documents pertaining to this trial will be maintained by the investigators and made available for direct inspection by authorized persons.

Investigator(s)/institution(s) will permit trial-related monitoring, audits, IRB/IEC review, and regulatory inspection(s) by providing direct access to source data/documents by authorized persons as defined in the ICF. In all cases, subject confidentiality must be maintained in accordance with local regulatory requirements.

10.1.6.2 Data Collection

During each subject's visit to the site, an investigator or their designee participating in the trial will record information to document all significant observations. At a minimum, these notes will contain:

- Documentation of the informed consent process, including any revised consents;
- Documentation of the investigator's decision to enroll the subject into the trial, the review of all inclusion/exclusion criteria prior to IMP administration, and confirmation of the subject's actual participation in the trial;
- The date of the visit and the corresponding Visit or Day in the trial schedule;
- General subject status remarks, including any *significant* medical findings. The severity, frequency, duration, action taken, and outcome of any AEs and the investigator's assessment of relationship to IMP must also be recorded;
- Any changes in concomitant medications or dosages;
- A general reference to the procedures completed, including dosing and IMP compliance;
- The signature (or initials) and date of the investigator (or designee) who made an entry in the medical record.

In addition, any contact with the subject via telephone or other means that provides significant clinical information will also be documented in the progress notes as described above.

Information from medical records and other source documents will be entered by investigative site personnel onto eCRFs in the sponsor's electronic data capture (EDC) system that is 21 Code of Federal Regulations Part 11 compliant. Changes to the data will be captured by an automatic audit trail in the EDC system.

Electronic data not entered on eCRF, such as data received from central laboratories and central ECG readers, will be reconciled using key data fields by the sponsor or the clinical research organization with the eCRF data to ensure consistency.

10.1.6.3 File Management at the Trial Site

The investigator will ensure that the trial site file is maintained in accordance with applicable ICH GCP guidance and as required by applicable local regulations. The investigator/institution will take measures to prevent accidental or premature destruction of these documents.

10.1.6.4 Records Retention at the Trial Site

Regulatory requirements for the archival of records for this trial necessitate that participating investigators maintain detailed clinical data for the longest of the following 3 periods:

- A period of at least 2 years after the date on which approval to market the drug is obtained (or if IMP development is discontinued, the date regulatory authorities were notified of discontinuation); OR
- A period of at least 3 years after the sponsor notifies the investigator that the final report has been filed with regulatory authorities.
- Longer, region-specific storage requirements, if applicable.

The investigator must not dispose of any records relevant to this trial without either (1) written permission from the sponsor or (2) provision of an opportunity for the sponsor to collect such records. The investigator will be responsible to maintain adequate and accurate electronic or hard copy source documents of all observations and data generated during this trial including any data clarification forms received from the sponsor. Such documentation is subject to inspection by the sponsor and relevant regulatory authorities. If the investigator withdraws from the trial (eg, due to relocation or retirement), all trial-related records should be transferred to a mutually agreed-upon designee within a sponsor-specified timeframe. Notice of such transfer will be given to the sponsor in writing.

10.1.6.5 Publication Authorship Requirements

Authorship for any Otsuka-sponsored publications resulting from the conduct of this trial will be based on International Committee of Medical Journal Editors (ICMJE) authorship criteria (http://www.icmje.org/recommendations). According to ICMJE guidelines, one may be considered an author only if the following criteria are met:

1) Substantial contributions to the conception or design of the work; or the acquisition, analysis, or interpretation of data for the work; AND
2) Drafting the work or revising it critically for important intellectual content; AND
3) Final approval of the version to be published; AND
4) Agreement to be accountable for all aspects of the work in ensuring that questions related to the accuracy or integrity of any part of the work are appropriately investigated and resolved.

All authors must meet the above criteria, and all who qualify for authorship based on the above criteria should be listed as authors.

Investigators or other trial subjects who do not qualify for authorship may be acknowledged in publications resulting from the trial. By agreeing to participate in the trial, investigators or other trial subjects consent to such acknowledgement in any publications resulting from its conduct.

10.2 Appendix 2: Clinical Laboratory Tests

The tests detailed in Table 10.2-1 will be performed.

Table 10.2-1 Clinical Laboratory Assessments

Hematology:<br>Hemoglobin<br>Hematocrit<br>Mean corpuscular hemoglobin concentration<br>Mean corpuscular volume<br>Red blood cell (RBC) count<br>White blood cell (WBC) count (absolute and differential)<br>Platelets<br>Coagulation (prothrombin time [PT], partial thromboplastin time [PTT], international normalized ratio [INR])<br><br>Urinalysis:<br>Specimen appearance<br>Color<br>Occult blood<br>Glucose<br>Microscopic analysis, if indicated, WBC/RBC counts per high powered field<br>pH<br>Protein<br>Specific gravity<br><br>Additional Tests:<br>Urine pregnancy for FOCBP<br>Serology (hepatitis B surface antigen [HbsAg], hepatitis C antibodies [anti-HCV], HIV),<br>SARS-CoV-2 polymerase chain reaction test (where appropriate)	Serum Chemistry:<br>Alkaline phosphatase<br>Alanine aminotransferase<br>Aspartate aminotransferase<br>Bilirubin<br>Blood urea nitrogen<br>Calcium<br>Cholesterol<br>Creatinine/eGFR<br>Gamma glutamyl transferase<br>Glucose<br>HbA1c<br>Lactate dehydrogenase<br>Magnesium<br>Potassium<br>Protein<br>Sodium<br>Triglycerides<br><br>Drug Screen (all items in urine except where noted)<br>Alcohol (urine or blood test)<br>Amphetamines<br>Barbiturates<br>Benzodiazepines<br>Cannabinoids<br>Cocaine<br>Methadone<br>Opiates<br>Phencyclidine

10.3 Appendix 3: Contraceptive Guidance and Collection of Pregnancy Information

Females of childbearing potential (FOCBP) are females whose menstruation has started and who are not documented as sterile (eg, have had a bilateral oophorectomy, or hysterectomy, or who have been postmenopausal for at least 12 months). Females of nonchildbearing potential do not meet definition of FOCBP.

For males and FOCBP, or their partners, who are sexually active, there must be a documented agreement that the subject and their partner will take effective measures (ie, 2 different approved methods of birth control or abstinence) to prevent pregnancy during the course of the trial and for 12 weeks after the last dose of IMP or RHEZ. Unless the subject is sterile (ie, females who have had a bilateral oophorectomy, have had a hysterectomy, or have been postmenopausal for at least 12 consecutive months; or males who have had a bilateral orchiectomy) and for 12 weeks after the last dose of IMP of RHEZ, 2 of the following approved methods of birth control must be used: vasectomy, tubal ligation, intrauterine device, birth control pill, birth control implant, birth control depot injection, birth control patch, condom, sponge, or occlusive cap (vaginal diaphragm or cervical/vault cap). Any single method of birth control, including vasectomy and tubal ligation, may fail, leading to pregnancy. The contraceptive method will be documented in the eCRF or source document. Male subjects must also agree not to donate sperm from trial screening through 12 weeks after the last dose of IMP or RHEZ.

Before enrolling males and females in this clinical trial, investigators must review the below information about trial participation as part of the ICF process. The topics should generally include:

- General information
- Informed consent form
- Pregnancy prevention information
- Drug interactions with hormonal contraceptives
- Contraceptives in current use
- Follow-up of a reported pregnancy

Before trial enrollment, males and FOCBP must be advised of the importance of avoiding pregnancy during trial participation and the potential risk factors for an unintentional pregnancy. Subjects must sign the ICF confirming that the above-mentioned risk factors and the consequences were discussed.

A urine or serum pregnancy test for human chorionic gonadotropin will be performed at screening on all FOCBP and female subjects ≥ 12 years of age, if menstruation has

started. If a urine test is performed and is positive, the investigator will follow-up with a confirmatory serum test.

During the trial, all FOCBP should be instructed to contact the investigator immediately if they suspect they might be pregnant (eg, missed or late menstrual cycle). Male subjects must be instructed to contact the investigator immediately, during the trial, if their partner suspects that they might be pregnant (eg, missed or late menstrual cycle).

If a subject is suspected to be pregnant before she receives IMP or RHEZ, the IMP or RHEZ administration must be withheld until the results of serum pregnancy tests are available. If the pregnancy is confirmed, the subject must not receive the IMP or RHEZ and must not be enrolled in the trial. If pregnancy is suspected while the subject is taking IMP or RHEZ, the IMP or RHEZ must be withheld immediately (if reasonable, taking into consideration any potential withdrawal risks) until the result of the pregnancy test is known. If pregnancy is confirmed, the IMP or RHEZ will be permanently discontinued in an appropriate manner (eg, dose tapering if necessary for subject safety) and the subject will be withdrawn from the trial. Exceptions to trial discontinuation may be considered for life-threatening conditions only after consultations with the IRE contact (see the title page of this protocol for contact information).

The investigator must immediately notify the sponsor (within 24 hours) of any pregnancy associated with IMP or RHEZ exposure during the trial and for at least 12 weeks after the last dose of IMP or RHEZ, and record the event on the IRE form and forward it to the sponsor. The sponsor will forward the Clinical Trial Breastfeeding and Pregnancy form in addition to the IRE form to the investigator for monitoring the outcome of the pregnancy.

Protocol required procedures for trial discontinuation and follow-up must be performed on the subject unless contraindicated by pregnancy (eg, x-ray studies). Other appropriate pregnancy follow-up procedures should be considered if indicated. In addition, the investigator must report to the sponsor, on the Clinical Trial Breastfeeding and Pregnancy Form(s), follow-up information regarding the course of the pregnancy, including perinatal and neonatal outcome. Infants will be followed for a minimum of 6 months from the date of birth. Local regulatory requirements must be followed for follow-up and reporting on pregnancy cases and/or infants.

10.4 Appendix 4: List of Abbreviations

Abbreviation	Definition
AE	Adverse event
AESI	Adverse event of special interest
ALT	Alanine aminotransferase
Anti-HCV	Hepatitis C antibodies
AST	Aspartate aminotransferase
AUC	Area under the concentration-time curve
AUC_{24h}	Area under the concentration-time curve from zero to 24 hours
BDQ	Bedaquiline
BID	Twice daily
CFR	Cumulative fraction of response
CFU	Colony-forming unit
CI	Confidence interval
C_{max}	Maximum (peak) plasma concentration
CYP	Cytochrome P450
DAIDS	Division of AIDS
DprE1	Decaprenylphosphoryl-β-D-ribose 2'-oxidase
DLM	Delamanid
DMC	Data Monitoring Committee
DOT	Directly observed therapy
DS-TB	Drug-susceptible tuberculosis
EBA	Early bactericidal activity
ECG	Electrocardiogram
eCRF	Electronic case report form
EDC	Electronic data capture
eGFR	Estimated glomerular filtration rate
ET	Early termination
[redacted]	[redacted]
FOCBP	Female of childbearing potential
GCP	Good Clinical Practice
HbA1c	Glycosylated hemoglobin
HBsAg	Hepatitis B surface antigen
HCV	Hepatitis C virus
HFS-TB	Hollow fiber system of tuberculosis
HIV	Human immunodeficiency virus
IB	Investigator's Brochure
ICF	Informed consent form
ICH	International Council for Harmonisation
ICMJE	International Committee of Medical Journal Editors
ID	Identification
IEC	Independent Ethics Committee
IMP	Investigational medicinal product
IND	Investigational New Drug
INR	International normalized ratio

Abbreviation	Definition
IRB	Institutional Review Board
IRE	Immediately reportable event
LAM	Lipoarabinomannan
MAD	Multiple ascending dose
MDR-TB	Multidrug-resistant tuberculosis
MGIT	Mycobacteria Growth Indicator Tube®
MIC	Minimum inhibitory concentration
mITT	Modified intent-to-treat
NOAEL	No observed adverse effect level
OBR	Optimized background regimen
Otsuka	Otsuka Pharmaceutical Co, Ltd
PET/CT	Positron emission tomography/computerized axial tomography
PK	Pharmacokinetics
PK/PD	Pharmacokinetics/pharmacodynamics
PQC	Product quality complaint
PT	Prothrombin time
PTT	Partial thromboplastin time
QD	Once daily
QTc	QT interval corrected
QTcF	QT interval corrected by Fridericia's method
RBC	Red blood cell
RHEZ	Rifampin, isoniazid, ethambutol, and pyrazinamide
RS ratio	Ribosomal ribonucleic acid synthesis ratio
SAE	Serious adverse event
SAP	Statistical analysis plan
SARS-CoV-2	Severe acute respiratory syndrome coronavirus
SCC	Sputum culture conversion
TB	Tuberculosis
TEAE	Treatment-emergent adverse event
TIW	Thrice-weekly
ULN	Upper limit of normal
WBC	White blood cell

10.5 Appendix 5: Schedule of Sputum Collections for Each Treatment Arm

Sample:		Sputum - MGIT Culture/ LAM	Sputum - MGIT Culture/ LAM	Sputum - RS Ratio
Collected by:		Participant at home	Site staff	Site staff
Day	-14 to -2	-	X (eligibility assessment only)	-
	-1	-	X	X
	1	-	X	X
Week	1	X	X	X
	2	X	X	X
	3	X	X	X
	4	X	X	X
	5	X	X	X
	6	X	X	X
	7	X	X	X
	8	X	X	X
	10	X	X	X
	12	X	X	X
	13	X	X	X
	15	X	X	X
	17	X	X	X
	19	X	X	X
	21	X	X	X
	23	X	X	X
	26	X	X	X
	28	X	X	X
Week	30 to 52/ET	X	X	X

10.6 Appendix 6: Protocol Amendments

The investigator will not make any changes to this protocol without the sponsor's prior written consent and subsequent approval/favorable opinion by the IRB/IEC. Any permanent change to the protocol, whether an overall change or a change for specific trial site(s), must be handled as a protocol amendment. Any amendment will be written by the sponsor. Each amendment will be submitted to the IRB/IEC, as required by local regulations. Except for "administrative" or "nonsubstantial" amendments, investigators will wait for IRB/IEC approval/favorable opinion of the amended protocol before implementing the change(s). Administrative amendments are defined as having no effect on the safety of subjects, conduct or management of the trial, trial design, or the quality or safety of IMP(s) used in the trial. A protocol change intended to eliminate an apparent immediate hazard to subjects should be implemented immediately after agreement by the sponsor and investigator, followed by IRB/IEC notification within local applicable timelines. The sponsor will submit protocol amendments to the applicable regulatory agencies within local applicable timelines.

When the IRB/IEC, investigators, and/or the sponsor conclude that the protocol amendment substantially alters the trial design and/or increases the potential risk to the subject, the currently approved written ICF will require similar modification. In such cases, after approval/favorable opinion of the new ICF by the IRB/IEC, repeat written informed consent will be obtained from subjects enrolled in the trial before expecting continued participation and before the amendment-specified changes in the trial are implemented.

10.6.1 Protocol Amendment(s)/Administrative Change(s)

Not applicable.

11 References

1. World Health Organization. Global tuberculosis report 2019. Geneva: World Health Organization; 2020.
2. Sirturo (bedaquiline) [prescribing information]. Titusville, NJ: Janssen Therapeutics, Division of Janssen Products, LP; May 2020.
3. Otsuka Pharmaceutical Co., Ltd. OPC-67683 Investigator's Brochure, Edition 16. Otsuka Report, issued 07 Apr 2020.
4. Gumbo T, Chapagain M, Magombedze G, Srivastava S, Deshpande D, Pasipanodya JG, et al. Novel tuberculosis combination regimens of two and three-months therapy duration. Submitted, Mar 2020.
5. Otsuka Pharmaceutical Development and Commercialization, Inc. OPC-167832 Investigator's Brochure, Edition 6, Otsuka Report, issued 20 Apr 2021.
6. Aventis Pharma (Pty) Ltd. Rifafour e-275 tablets Package Insert. South African Electronic Package Inserts. Published 11 Feb 2002.
7. Miettinen O, Nurminen M. Comparative analysis of two rates. Stat Med. 1985;4(2):213–226.
8. United States Department of Health and Human Services, Division of AIDS, National Institute of Allergy and Infectious Diseases, National Institutes of Health. Division of AIDS (DAIDS) Table for Grading the Severity of Adult and Pediatric Adverse Events; 2017.
9. European Medicines Agency, Science Medicines Health. ICH E9 (R1) addendum on estimands and sensitivity analysis in clinical trials to the guideline on statistical principles for clinical trials. London, England: European Medicines Agency; 2017.

Agreement

I, the undersigned principal investigator, have read and understand the protocol (including the Investigator's Brochure) and agree that it contains all the ethical, legal and scientific information necessary to conduct this trial in accordance with the principles of Good Clinical Practices and as described herein and in the sponsor's (or designee's) Clinical Trial Agreement.

I will provide copies of the protocol to all physicians, nurses, and other professional personnel to whom I delegate trial responsibilities. I will discuss the protocol with them to ensure that they are sufficiently informed regarding the investigational new drug, OPC-167832, the concurrent medications, the efficacy and safety parameters and the conduct of the trial in general. I am aware that this protocol must be approved by the Institutional Review Board (IRB) or receive a favorable opinion by the Independent Ethics Committee (IEC) responsible for such matters in the clinical trial facility where OPC-167832 will be tested prior to commencement of this trial. I agree to adhere strictly to the attached protocol (unless amended in the manner set forth in the sponsor's Clinical Trial Agreement, at which time I agree to adhere strictly to the protocol as amended).

I understand that this IRB/IEC-approved protocol will be submitted to the appropriate regulatory authority/ies by the sponsor. I agree that clinical data entered on eCRF by me and my staff will be utilized by the sponsor in various ways, such as for submission to governmental regulatory authorities and/or in combination with clinical data gathered from other research sites, whenever applicable. I agree to allow sponsor and designee monitors and auditors full access to all medical records at the research facility for subjects screened or enrolled in the trial.

I agree to await IRB/IEC approval before implementation of any substantial amendments to this protocol. If, however, there is an immediate hazard to subjects, I will implement the amendment immediately, and provide the information to the IRB/IEC within the required local applicable timelines. Administrative changes to the protocol will be transmitted to the IRB/IEC for informational purposes only, if required by local regulations.

I agree to provide all subjects with informed consent forms, as required by the applicable regulations and by ICH guidelines. I agree to report to the sponsor any adverse experiences in accordance with the terms of the sponsor's Clinical Trial Agreement and the relevant regional regulation(s) and guideline(s). I further agree to provide all required information regarding financial certification or disclosure to the sponsor for all investigators and subinvestigators in accordance with the terms of the relevant regional regulation(s). I understand that participation in the protocol involves a commitment to publish the data from this trial in a cooperative publication before publication of efficacy and safety results on an individual basis may occur, and I consent to be acknowledged in any such cooperative publications that result.

______________________________	______________________________	____________
Principal Investigator Print Name	Signature	Date

Otsuka Pharmaceutical
Development & Commercialization, Inc.

Investigational Medicinal Product

OPC-167832

CLINICAL PROTOCOL

A Multicenter, Phase 2b/c, Open-label, Randomized, Dose-finding Trial to Evaluate the Safety and Efficacy of a 4-month Regimen of OPC-167832 in Combination with Delamanid and Bedaquiline in Participants with Drug-susceptible Pulmonary Tuberculosis in Comparison with Standard Treatment

Protocol No. 323-201-00006
IND No. 129303

CONFIDENTIAL — PROPRIETARY INFORMATION

Clinical Development Phase:	Phase 2b/c
Sponsor:	Otsuka Pharmaceutical Development & Commercialization, Inc.
Immediately Reportable Event	Clindev Pty Ltd [redacted]
[redacted]	
Amendment 2 Approval:	08 Oct 2021

Table of Contents

Table of Contents 2
List of In-text Tables 7
List of In-text Figures 8
1 Protocol Summary 9
1.1 Synopsis 9
1.2 Schema 17
1.3 Schedule of Assessments 19
2 Introduction 24
2.1 Trial Rationale 24
2.2 Background 25
2.3 Known and Potential Risks and Benefits 26
2.3.1 OPC-167832 26
2.3.2 Delamanid 28
2.3.3 Bedaquiline 29
2.3.4 Rifampin, Isoniazid, Ethambutol, and Pyrazinamide 29
3 Objectives and Endpoints 29
4 Trial Design 31
4.1 Type/Design of Trial 31
4.2 Scientific Rationale for Trial Design 31
4.3 Dosing Rationale 32
4.4 End of Trial Definition 33
4.5 Definition of Completed Participants 33
5 Trial Population 33
5.1 Participant Selection and Numbering 34
5.2 Eligibility Criteria 34
5.2.1 Inclusion Criteria 34
5.2.2 Exclusion Criteria 34
5.3 Lifestyle Considerations 36
5.3.1 Meals and Dietary Restrictions 36
5.3.2 Caffeine, Alcohol, and Tobacco 37

5.4 Screen Failures 37
6 Trial Treatments 37
6.1 Trial Treatments Administered 37
6.1.1 Medical Devices 38
6.2 Management of Investigational Medicinal Product 38
6.2.1 Packaging and Labeling....38
6.2.2 Storage 39
6.2.3 Accountability....39
6.2.4 Returns and Destruction 39
6.2.5 Reporting of Product Quality Complaints 39
6.2.5.1 Eliciting and Reporting Product Quality Complaints 40
6.2.5.2 Information Required for Reporting Purposes....40
6.2.5.3 Return Process....41
6.2.5.4 Assessment/Evaluation 41
6.3 Measures to Minimize/Avoid Bias....41
6.4 Participant Compliance 41
6.5 Concomitant Medications or Therapies 41
6.5.1 Prohibited Medications or Therapies....42
6.5.2 Permitted Medications or Therapies....43
6.5.3 Rescue Medications....43
6.6 Intervention After the End of the Trial....43
7 Stopping Rules, Withdrawal Criteria, and Procedures....43
7.1 Entire Trial or Treatment....43
7.2 Individual Site 44
7.3 Individual Participant Discontinuation....44
7.3.1 Treatment Interruption....44
7.3.2 Treatment Discontinuation 44
7.3.3 Documenting Reasons for Treatment Interruption or Discontinuation 45
7.3.4 Withdrawal of Consent....46
7.3.5 Procedures to Encourage Continued Trial Participation 47
7.4 Definition of Participants Lost to Follow-up 47
8 Trial Procedures....47

8.1 Efficacy Assessments 48
8.1.1 Sputum Sampling and Microbiology Laboratory Assessments 48
8.1.1.1 Sputum for Eligibility Assessments 48
8.1.1.2 Sputum for Efficacy Assessments 48
8.1.2 Signs and Symptoms of Tuberculosis 49
8.2 Pharmacokinetic Assessments 49
8.2.1 Pharmacokinetic Blood/Serum/Plasma Samples 50
8.3 Pharmacodynamic Assessments 50
8.3.1 Pharmacodynamic Laboratory Tests 50
8.4 Pharmacogenomic Assessments 51
CCI [redacted]
8.7 Safety Assessments 52
8.7.1 Clinical Laboratory Assessments 52
8.7.2 Physical Examination 52
8.7.3 Vital Signs 52
8.7.4 Electrocardiogram 53
8.7.5 Suicidality Monitoring 53
8.7.6 Other Safety Variables 53
8.8 Adverse Events 53
8.8.1 Definitions 53
8.8.2 Eliciting and Reporting Adverse Events 55
8.8.3 Immediately Reportable Events 56
8.8.4 Adverse Events of Special Interest 56
8.8.5 Potential Serious Hepatotoxicity 56
8.8.6 Procedure for Breaking the Blind 56
8.8.7 Follow-up of Adverse Events 57
8.8.7.1 Follow-up of Nonserious Adverse Events 57
8.8.7.2 Follow-up of Immediately Reportable Events 57
8.8.7.3 Follow-up and Reporting of Immediately Reportable Events Occurring After Last Scheduled Contact 58

8.9 Treatment of Overdose 58

8.10 Participant Assessment Recording 58

8.11 Other Assessments 58

9 Statistical Considerations 58

9.1 Sample Size 58

9.2 Datasets for Analysis 59

9.3 Handling of Missing Data for Primary and Secondary Endpoint Analysis 59

9.4 Statistical Analyses 59

9.4.1 Efficacy Analyses 59

9.4.1.1 Primary Efficacy Endpoint Analysis 59

9.4.1.2 Secondary Efficacy Endpoint Analysis 60

9.4.1.3 Exploratory Biomarker Endpoint Analysis 60

9.4.2 Safety Analysis 60

9.4.2.1 Adverse Events 61

9.4.2.2 Clinical Laboratory Data 61

9.4.2.3 Physical Examination and Vital Signs Data 61

9.4.2.4 Electrocardiogram Data 61

9.4.3 Other Analyses 62

9.4.3.1 Analysis of Demographic and Baseline Characteristics 62

9.4.3.2 Pharmacokinetic Analysis 62

9.4.3.3 Pharmacodynamic Analysis 62

9.4.3.4 Exposure-Response Analysis 62

9.4.3.5 Pharmacogenomic Analysis 62

CCI [redacted]

9.5 Interim Analysis and Adaptive Design 63

9.5.1 Data Monitoring Committee 64

10 Supporting Documentation and Operational Considerations 65

10.1 Appendix 1: Regulatory, Ethical, and Trial Oversight Considerations 65

10.1.1 Ethics and Responsibility 65

10.1.2 Informed Consent 65

10.1.3 Confidentiality 66

10.1.4 Quality Control and Quality Assurance 66

10.1.4.1 Monitoring 66
10.1.4.2 Auditing 67
10.1.5 Protocol Deviations 67
10.1.6 Records Management 67
10.1.6.1 Source Documents 67
10.1.6.2 Data Collection 68
10.1.6.3 File Management at the Trial Site 68
10.1.6.4 Records Retention at the Trial Site 69
10.1.6.5 Publication Authorship Requirements 69
10.2 Appendix 2: Clinical Laboratory Tests 71
10.3 Appendix 3: Contraceptive Guidance and Collection of Pregnancy Information 72
10.4 Appendix 4: List of Abbreviations 74
10.5 Appendix 5: Schedule of Sputum Collections for All Treatment Arms 76
10.6 Appendix 6: Completer Category Information 77
10.7 Appendix 7: Protocol Amendments 78
10.7.1 Protocol Amendment(s)/Administrative Change(s) 79
11 References 80

List of In-text Tables

Table 1.3-1	Schedule of Assessments - All Participants	19
Table 3-1	Trial Objectives and Endpoints	29
Table 6.5.1-1	List of Selected Cytochrome P450 3A4 Inhibitors Prohibited During the Trial	42
Table 6.5.1-2	List of Selected Cytochrome P450 3A4 Inducers Prohibited During the Trial	43

List of In-text Figures

Figure 1.2-1 Trial Design Schematic....17

1 Protocol Summary

1.1 Synopsis

Name of Sponsor: Otsuka Pharmaceutical Development & Commercialization, Inc.

Name of Investigational Medicinal Products: OPC-167832, bedaquiline (BDQ), and delamanid (DLM)

Protocol No.: 323-201-00006

IND No.: 129303

Protocol Title: A Multicenter, Phase 2b/c, Open-label, Randomized, Dose-finding Trial to Evaluate the Safety and Efficacy of a 4-month Regimen of OPC-167832 in Combination with Delamanid and Bedaquiline in Participants with Drug-susceptible Pulmonary Tuberculosis in Comparison with Standard Treatment

Clinical Phase: Phase 2b/c, Therapeutic use

Treatment/Indication: Drug-susceptible pulmonary tuberculosis (DS-TB)

Objectives and Endpoints:

Objectives	Endpoints
Primary:<br>• To assess the safety and efficacy of OPC-167832 combined with DLM and BDQ in participants with DS-TB administered for 4 months compared to rifampin, isoniazid, ethambutol, pyrazinamide (RHEZ) administered for 6 months	Safety:<br>• Adverse events (AEs), clinical laboratory tests, vital signs, physical examinations, electrocardiograms (ECGs)<br>• The proportion of participants with a grade 3 or higher AE<br>• The rate of all-cause treatment discontinuation<br><br>Efficacy:<br>• The proportion of participants achieving sputum culture conversion ([SCC] in Mycobacteria Growth Indicator Tube® [MGIT]) by the end of the treatment period

Objectives	Endpoints
Secondary:<br>• To evaluate efficacy in sputum MGIT culture and lipoarabinomannan (LAM) for OPC-167832 combined with DLM and BDQ administered for 4 months as compared to RHEZ<br>• To evaluate the development of resistance to anti-tuberculosis (TB) drugs in all treatment arms<br>• To inform dose selection of OPC-167832 combined with DLM and BDQ	Efficacy:<br>• The proportion of participants who achieve SCC in MGIT by 8 weeks of treatment<br>• Time to detection of MGIT cultures<br>• The proportion of participants who convert sputum LAM to negative by 8 weeks of treatment and by end of treatment<br>• Proportion of participants who develop drug resistance<br>• Time to SCC of OPC-167832, DLM, and BDQ, as well as RHEZ
Exploratory Biomarker Analysis:<br>• To evaluate various novel biomarkers for association with microbiological and clinical response	Efficacy:<br>• Longitudinally assess positron emission tomography/computerized axial tomography (PET/CT) imaging response over the course of treatment<br>• Longitudinally evaluate the ribosomal ribonucleic acid synthesis ratio (RS ratio) decline in sputum<br>• Longitudinally assess whole blood transcriptomic signatures previously associated with TB cure from serum

CCI [redacted]

Trial Design:

Trial 323-201-00006 is a multicenter, phase 2b/c, open-label, randomized, dose-finding trial in participants with DS-TB. The microbiology laboratory will be blinded to treatment assignments.

Following a screening period of up to 14 days, eligible participants will be randomized on Day −1 or predose Day 1 in a ratio of 1:2:2:1 to one of the following treatment arms:

- Arm 1: DLM (300 mg once daily [QD]) + BDQ (400 mg QD x 2 weeks, then 200 mg thrice-weekly [TIW]) + OPC-167832 (10 mg QD) for 17 weeks
- Arm 2: DLM (300 mg QD) + BDQ (400 mg QD x 2 weeks, then 200 mg TIW) + OPC-167832 (30 mg QD) for 17 weeks
- Arm 3: DLM (300 mg once daily [QD]) + BDQ (400 mg QD x 2 weeks, then 200 mg TIW) + OPC-167832 (90 mg QD) for 17 weeks
- Arm 4: RHEZ for 8 weeks followed by 18 weeks of rifampin and isoniazid (for a total of 26 weeks)

Randomization will be stratified by HIV status (yes or no) and presence of bilateral cavitation on screening chest x-ray (yes or no). After the end of the treatment period, participants will be followed until 12 months postrandomization.

Trial Population:

The trial population will consist of participants who are ≥ 18 years to ≤ 65 years at screening with body weight of ≥ 35.0 kg and who have newly diagnosed pulmonary DS-TB. Approximately 120 participants (including no more than 15% with HIV-coinfection) will be enrolled at 8 sites in South Africa.

Key Inclusion/Exclusion Criteria:

Key inclusion criteria are noted above, under Trial Population.

Key exclusion criteria are as follows:

- Participants are known or suspected of having resistance to rifampin, isoniazid, ethambutol, pyrazinamide, DLM, or BDQ either confirmed by the laboratory, or based on epidemiological history, at screening.
- Evidence of clinically significant metabolic (for example, including ongoing or current hypokalemia [ie, potassium < 3.5 mEq/dL at screening]), gastrointestinal, neurological, psychiatric, endocrine or liver (eg, hepatitis B and C) disease; malignancy; or other abnormalities (other than the indication being studied).
- History of, or current, clinically relevant cardiovascular disorder such as heart failure, coronary heart disease, uncontrolled hypertension, arrhythmia or symptom strongly suggestive of such a problem (for example, syncope or palpitations), tachyarrhythmia or status after myocardial infarction.
- Known bleeding disorders or family history of bleeding disorders.
- Any diseases or conditions in which the use of DLM, BDQ, OPC-167832, rifampin, isoniazid, pyrazinamide, or ethambutol is contraindicated.

- Clinical evidence of severe extrapulmonary TB (eg, miliary TB, abdominal TB, urogenital TB, osteoarthritic TB, TB meningitis).
- Evidence of pulmonary silicosis, lung fibrosis, or other lung condition considered as severe by the investigator (other than TB). In particular, any underlying condition that could interfere with the assessment of x-ray images, sputum collection, or interpretation of sputum findings, or otherwise compromise the subject's participation in the trial.
- Any renal impairment characterized by creatinine clearance/estimated glomerular filtration rate of < 60 mL/min/1.73 m^2, hepatic impairment characterized by alanine transaminase or aspartate transaminase > 2.0 × upper limit of normal of the clinical laboratory reference range or bilirubin > 2.0 × upper limit of normal of the clinical laboratory reference range, at screening.
- Screening glucose (nonfasting) ≥ 200 mg/dL or glycosylated hemoglobin ≥ 6.5%.
- QT interval corrected by Fridericia's method > 450 msec in male participants (> 470 msec in female participants), atrioventricular block II or III, bi-fasicular block, at screening or current or history of clinically significant ventricular arrhythmias. Other ECG abnormalities, if considered clinically significant by the investigator.
- Participants receiving any of the prohibited medications (see Section 6.5.1) within the specified periods or who would be likely to require prohibited concomitant therapy during the trial.
- Current history of significant drug and/or alcohol abuse that is likely to result in poor adherence to trial requirements or that would pose a risk to the participant's well-being during the course of the trial.
- Participants who test positive for cocaine or other drugs of abuse (excluding known prescription stimulants and other prescribed medications and marijuana) at screening are excluded. Detectable levels of alcohol, marijuana, barbiturates, or opiates in the drug screen are not exclusionary if, in the investigator's documented opinion, the participant does not meet Diagnostic and Statistical Manual of Mental Disorders, Fifth Edition criteria for moderate to severe substance use disorder and the positive test does not signal a clinical condition that would impact the safety of the participant or interpretation of the trial results, and participation is agreed to by the medical monitor prior to treatment.
- History of having taken an investigational drug within 30 days preceding trial entry.
- Participants with significant medical comorbidities that in the opinion of the investigator, should not participate in the trial.
- Participants with Karnofsky score < 60 will be excluded from the trial.
- Participants with HIV co-infection not on a stable anti-retroviral regime consisting of a combination of tenofovir, emtricitabine/ lamivudine, plus dolutegravir (ie > 3 months), or who have a detectable viral load, or who have a CD4 count < 350 cells/mm^3 will be excluded from the trial.

Trial Site(s):

This trial will be conducted at approximately 8 sites in South Africa.

Investigational Medicinal Product(s), Dose, Dosage Regimen, Treatment Duration, Formulation, Mode of Administration:

All investigational medicinal product (IMP) (ie, OPC-167832, DLM, or BDQ) must be administered after the completion of a meal.

Bedaquiline should be administered as follows: 400 mg QD for the first 2 weeks of treatment and 200 mg TIW thereafter with at least 48 hours between doses for the remainder of the trial (ie, a total of 17 weeks [Arm 1 or Arm 2 or Arm 3] of treatment).

Participants randomized to treatment Arm 4 will receive RHEZ orally QD for 8 weeks followed by 18 weeks of rifampin and isoniazid per the local standard of care.

Trial treatments (IMP or RHEZ) will be administered using the locally accepted adherence monitoring techniques which could include directly observed therapy (DOT), Wisepill, video DOTs, or other adherence monitoring techniques. In addition, all doses of IMP or RHEZ given during trial visits will be observed via the hand-and-mouth procedure. All trial treatments will be administered with approximately 240 mL of still (noncarbonated) water.

Trial Assessments:

Assessments for Efficacy: Sputum for MGIT culture, LAM concentration, drug susceptibility testing, and *Mycobacterium tuberculosis* strain typing (as necessary); sputum for RS ratio; assessment of clinical signs and symptoms of TB; PET/CT scan; chest radiograph; and whole blood biomarkers.

Assessments for Safety: AE reporting; clinical laboratory tests (serum chemistry, hematology, urinalysis, and coagulation); physical examination; vital signs; ECGs; and concomitant medications.

Screening (excluding safety assessments mentioned above)/Other: demographics, medical history, prior medications; height and weight; visual assessment; sputum for confirmation of DS-TB; viral hepatitis serology (hepatitis B surface antigen [HBsAg] and hepatitis C virus [HCV]) and HIV screening (and CD4 count and HIV viral load for those with a known history of HIV); urine pregnancy testing; urine or blood alcohol, and urine drug screen; severe acute respiratory syndrome coronavirus (SARS-CoV-2) testing.

Assessments for PK: PK samples will be used to analyze concentrations of OPC-167832, DLM and its metabolite DM-6705, and BDQ and its metabolite M2.

[redacted]

Data Monitoring Committee:

An independent Data Monitoring Committee (DMC) will be contracted by Otsuka to review safety, efficacy, and PK data during the conduct of the trial. The DMC will convene and operate based on an established charter. The DMC will be permitted to seek additional independent expertise, as needed. The primary responsibility of the DMC will be to monitor safety and to advise the sponsor of the clinical project regarding whether or not the safety concerns merit stopping the trial. Towards this objective the DMC will meet on a predetermined schedule to review safety and available efficacy and PK data.

Statistical Methods: Primary: Safety endpoints will be summarized using descriptive statistics (number, median, mean, standard deviation, minimum, maximum) for continuous values, and by frequency and percentage for discrete variables. Percentage and frequency of adverse events and clinical laboratory test results by grades will be analyzed.

The modified intent-to-treat (mITT) analysis set includes all randomized participants who take at least 1 dose of IMP or RHEZ, have at least 1 postbaseline culture result, and demonstrate positive culture results by 1 week and susceptibility to rifampin and isoniazid (from the screening specimen).

The primary efficacy endpoint of the trial is the proportion of participants who have SCC and no subsequent positive culture at the end of treatment. Sputum culture conversion occurs when a participant has the first of 2 visits of at least 1 week apart with sputum cultures negative and without a positive sputum culture result in between.

The 3 DLM + BDQ + OPC-167832 arms will be pooled, and noninferiority with RHEZ will be established if the lower limit of 80% confidence interval (CI) for the difference of proportion of participants with SCC between the pooled DLM + BDQ + OPC-167832 group is larger than -0.12. The Miettinen and Nurminen CI of common risk difference stratified by HIV status and cavitation status prior to randomization will be used. If there is a stratum of very small size, strata may be collapsed. The statistical analysis plan (SAP) will describe the details.

The 80% CI of the proportion difference between each dose level of DLM + BDQ + OPC-167832 arm and RHEZ will be provided.

In exploratory analysis, the difference between proportion of participants with favorable outcome between each DLM + BDQ + OPC-167832 group and [redacted] group will be studied.

Definition of favorable and **unfavorable** outcome:

A participant is considered to have a **favorable** outcome if, during the postrandomization evaluation period, the participant is alive, completed the postrandomization evaluation, and otherwise did not meet the definition of an unfavorable outcome.

A participant is considered to have an **unfavorable** outcome if one of the following occurs:

1) Clinical progression of pulmonary disease during treatment or the need for an unanticipated surgical intervention for TB (eg, lobectomy)
2) Any growth of *M tuberculosis* from an extrapulmonary site during the post-treatment period (Note: Excludes reinfection with a new strain of *M tuberculosis*).
3) Signs and symptoms of active tuberculosis during the follow-up period (ie, after the end of treatment), including radiographic worsening compared to baseline findings, resulting in re-initiation of anti-mycobacterial therapy (Note: excludes a re-initiation that is subsequently found to be due to a misdiagnosis and/or alternative diagnosis)
4) Death during treatment or follow-up
5) A sputum culture with growth of *M tuberculosis* at certain time points as follows:
    - Failure to achieve SCC by the end of the treatment period that results in a change in anti-mycobacterial therapy.
    - Microbiologic relapse during the follow-up period (ie, after the end of treatment) (Note: Excludes reinfection and requires more than 1 positive sputum culture with a new strain of *M tuberculosis*).

Pharmacokinetics/pharmacodynamics and/or exposure-response analysis will be used to analyze the relationship of OPC-167832 plasma exposure alone or combined with DLM and BDQ with the efficacy and safety endpoints, as applicable.

Trial Duration:

Each participant in this trial is expected to participate in the following periods of the trial (approximate durations listed):

- Eligibility screening period (up 14 days)
- Treatment period (Arm 1, Arm 2, and Arm 3: 17 weeks; or Arm 4: 26 weeks)
- Post-treatment follow-up period (12 months postrandomization)

Overall, the per-patient trial duration from signing of the first informed consent form to the final participant assessment is expected to be approximately 12 months.

1.2 Schema

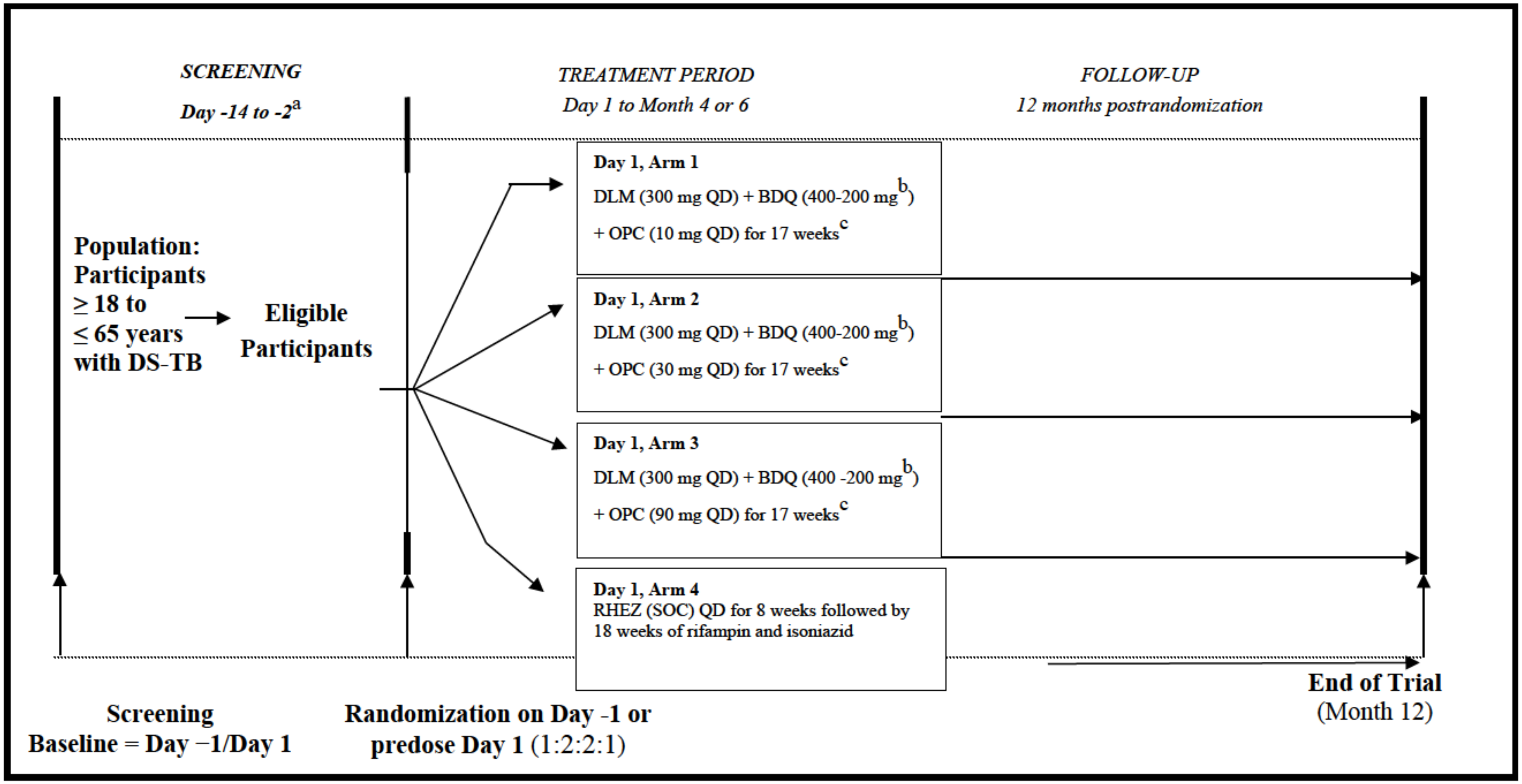

Figure 1.2-1 Trial Design Schematic

SOC = standard-of-care.

[a]Screening can occur for up to 14 days but should be completed in as few days as possible.

[b]BDQ dose 400 mg QD x 2 weeks, then 200 mg TIW.

[c]Rescue treatment consisting of the standard of care treatment, ie, RHEZ, will be provided to participants in Arm 1, or Arm 2, or Arm 3 in the following circumstances: 1) If a participant is sputum culture positive for *M tuberculosis* at the end of treatment; 2) If a participant has a microbiologic relapse subsequent to the end of treatment; or, 3) If, in the opinion of the investigator, a participant is clinically deteriorating, based on any combination of signs, symptoms, or chest radiograph data. Participants in Arm 4 will be managed according to local guidelines if not responding to therapy.

1.3 Schedule of Assessments

Table 1.3-1 Schedule of Assessments - All Participants

Period	Pre-treatment Period		Treatment Period																		Follow-up Period								
	SCR	BSL																										
**Visit**	**1**	**2**	**3**	**4**	**5**	**6**	**7**	**8**	**9**	**10**	**11**	**12**	**13**	**14**	**15**	**16**	**17**	**18**	**19**	**20**	**21**	**22**	**23**	**24**	**25**	**26**	**27**	**ET**	**Notes**
	**Day**			**Week (± 2 days)**																			**Week (± 7 days)**						
**Day/ Week**	**−14 to −2**	**−1**	**1**	**1**	**2**	**3**	**4**	**5**	**6**	**7**	**8**	**10**	**12**	**13**	**15**	**17**	**19**	**21**	**23**	**26**	**28**	**30**	**34**	**39**	**43**	**47**	**52**	**N/A**	
Informed consent	X																												Section 10.1.2
Demography	X																												Section 5.1
Concomitant medications	X	X	X	X	X	X	X	X	X	X	X	X	X	X	X	X	X	X	X	X	X	X	X	X	X	X	X	X	Section 6.5
Medical history	X																												Section 5.2
TB treatment history	X																												Section 5.2
Inclusion/ exclusion criteria	X	X																											Section 5.2
Chest radiograph[a]	X															X[a]				X[a]							X	X	Section 8.1.2
Randomization		X																											Section 4.1
Physical examination	X	X[b]			X[b]		X[b]		X[b]		X[b]	X[b]	X[b]	X[b]	X[b]	X[b]		X[b]		X[b]		X[b]	X[b]	X[b]	X[b]	X[b]	X	X[b]	Section 8.7.2
Karnofsky score	X																												Section 5.2.2
Vital signs[c]	X	X			X		X		X		X	X	X	X	X	X		X		X	X	X	X	X	X	X	X	X	Section 8.7.3

Table 1.3-1	Schedule of Assessments - All Participants																												
**Period**	**Pre-treatment Period**		**Treatment Period**																			**Follow-up Period**							
	**SCR**	**BSL**																											
**Visit**	**1**	**2**	**3**	**4**	**5**	**6**	**7**	**8**	**9**	**10**	**11**	**12**	**13**	**14**	**15**	**16**	**17**	**18**	**19**	**20**	**21**	**22**	**23**	**24**	**25**	**26**	**27**	**ET**	**Notes**
	**Day**			**Week (± 2 days)**																			**Week (± 7 days)**						
**Day/ Week**	**−14 to −2**	**−1**	**1**	**1**	**2**	**3**	**4**	**5**	**6**	**7**	**8**	**10**	**12**	**13**	**15**	**17**	**19**	**21**	**23**	**26**	**28**	**30**	**34**	**39**	**43**	**47**	**52**	**N/A**	
Visual assessment[d]	X															X				X									Section 8.7.2
PET/CT scan[e]		X[e]					X[e]									X[e]				X[e]									Section 8.7.6
Standard 12-lead ECG[f]	X	X			X		X		X		X	X	X	X	X	X		X		X		X	X	X	X	X	X	X	Section 8.7.4
Signs and symptoms of TB	X	X			X		X		X		X			X		X		X		X		X	X	X	X	X	X	X	Section 8.1.2
Hematology, serum chemistry, and urinalysis	X	X			X		X		X		X			X		X		X		X							X	X	Section 8.7.1 and 10.2
HbA1c[g]	X																												Section 10.2
Coagulation	X																												Section 8.7.1
Urine drug screen	X																												Section 8.7.1
Urine pregnancy test FOCBP	X																										X	X	Section 10.3
HIV[h], HBsAg, anti-HCV	X																												Section 8.7.1

Table 1.3-1 Schedule of Assessments - All Participants

Period	Pre-treatment Period		Treatment Period																		Follow-up Period								
	SCR	BSL																											
**Visit**	**1**	**2**	**3**	**4**	**5**	**6**	**7**	**8**	**9**	**10**	**11**	**12**	**13**	**14**	**15**	**16**	**17**	**18**	**19**	**20**	**21**	**22**	**23**	**24**	**25**	**26**	**27**	**ET**	**Notes**
	**Day**			**Week (± 2 days)**																			**Week (± 7 days)**						
**Day/ Week**	**−14 to −2**	**−1**	**1**	**1**	**2**	**3**	**4**	**5**	**6**	**7**	**8**	**10**	**12**	**13**	**15**	**17**	**19**	**21**	**23**	**26**	**28**	**30**	**34**	**39**	**43**	**47**	**52**	**N/A**	
Testing for SARS-CoV-2	X																												Section 5.2.2
Sputum for eligibility	X																												Section 8.1.1.1
Sputum MGIT culture/ sputum LAM		X	X[i]	X	X	X	X	X	X	X	X	X	X	X	X	X	X	X	X	X	X	X	X	X	X	X	X	X	Section 8.1.1
Acid fast bacilli smear microscopy		X																											
Susceptibility testing[j]		X	X														X									X			Section 8.1.1.2
Sputum for RS ratio[k]		X	X	X	X	X	X	X	X	X	X	X	X	X	X	X	X	X	X	X	X	X	X	X	X	X	X	X	Section 8.1.1
M tuberculosis strain genotyping[l]		X	X	X												X	X	X	X	X	X	X	X	X	X	X	X	X	Section 8.1.1
Paxgene tube for transcrip-tomics	X	X			X		X		X		X			X		X		X		X							X	X	Section 8.5
IMP admin-istration[m]			X	X	X	X	X	X	X	X	X	X	X	X	X	X	X	X	X	X	X								Section 6

Table 1.3-1 Schedule of Assessments - All Participants

Period	Pre-treatment Period		Treatment Period																		Follow-up Period								
	SCR	BSL																											
**Visit**	1	2	3	4	5	6	7	8	9	10	11	12	13	14	15	16	17	18	19	20	21	22	23	24	25	26	27	ET	Notes
	Day			Week (± 2 days)																			Week (± 7 days)						
**Day/ Week**	−14 to −2	−1	1	1	2	3	4	5	6	7	8	10	12	13	15	17	19	21	23	26	28	30	34	39	43	47	52	N/A	
Compliance check of IMP[m]			X	X	X	X	X	X	X	X	X	X	X	X	X	X	X	X	X	X	X								Section 6.4
Sparse PK[n]			X		X		X				X			X		X												X	Section 8.2
HIV viral load, CD4 count[o]	X																			X							X	X	Section 10.2
AEs and IREs	X	X	X	X	X	X	X	X	X	X	X	X	X	X	X	X	X	X	X	X	X	X	X	X	X	X	X	X	Section 8.8

BSL= Baseline; ET = early termination; FOCBP = female of childbearing potential; HbA1c = glycosylated haemoglobin; IRE = immediately reportable event; SCR=Screening.

[a]An end of treatment chest radiograph will be taken at Week 17 (for participants in Arm 1, Arm 2, and Arm 3) or Week 26 (for participants in Arm 4) and at Month 12.

[b]A full physical examination will be conducted at screening and Week 52. A targeted physical examination is a review that focuses on participant driven issues eg, the investigator enquires if the participant has any complaints, pains or disturbances and this would lead to further evaluation of the problematic area. At a minimum, an assessment of general appearance and a chest examination should be performed. Note, visual assessments to be completed during screening will consist of Snellen and Ishihara testing and will be repeated during the trial if the investigator suspects abnormalities.

[c]At screening, blood pressure and heart rate will be taken with the participant in the supine (performed first) and sitting position after remaining for ≥ 3 minutes in each position and temperature and respiratory rate will be taken with the participant in the supine position. At all other time points, vital signs will be obtained after the participant has been at rest in the supine position for at least 1 minute. Height will only be captured at screening. Weight, as well as BMI will also be captured at screening and other applicable time-points.

[d]Visual assessments will occur at screening, Week 17 (for participants in Arm 1, Arm 2, and Arm 3), or Week 26 (for participants in Arm 4). Note, visual assessments to be completed during screening will consist of Snellen and Ishihara testing and will be repeated during the trial if the investigator suspects abnormalities.

[e]Only for participants consenting to this trial procedure. The PET/CT scan will be done fasting within 1 week prior to starting treatment, Week 4 (± 7 days), and at the end of treatment visit (± 7 days) for each group (ie, Week 17 for Arm 1, Arm 2, and Arm 3, and Week 26 for Arm 4).

[f]One standard 12-lead ECG will be performed during screening, plus 3 ECGs at baseline (Day −1) spaced 5 to 10 minutes apart after the dose of IMP and prior to PK blood draws. Triplicate ECGs will be performed at subsequent visits during the treatment and follow up periods.

[g]In diabetics, HbA1c should be repeated every 3 months.

[h]If not already known to be HIV-infected. This HIV test can be repeated if clinically indicated.

[i]The Day 1 sputum will be collected prior to the first dose of IMP.

[j]Drug susceptibility testing for anti-tuberculosis drugs will be performed on Day -1 (or Day 1 if Day -1 is not useable) and on the end of treatment visit, if positive for *M tuberculosis* (Week 17 for Arm 1, Arm 2, and Arm 3, and Week 26 for Arm 4).

[k]If the participant is willing and able to spontaneously produce a subsequent specimen during the visit, specimen number 3 will be collected directly into ribonucleic acid preservation media and frozen for batch measuring of the RS ratio. If this sputum cannot be collected, this will not be considered a protocol deviation.

[l]*M tuberculosis* genotyping will be performed on a baseline culture (Day -1 and/or Day 1, or Week 1 if necessary) and the first of any subsequent positive culture during the posttreatment follow-up period to determine if the participant has relapsed or has new infection of *M tuberculosis*. In addition, the sponsor may request genotyping as needed.

[m]Participants in Arm 1, Arm 2, and Arm 3 will receive DLM and OPC-167832 QD for 17 weeks combined with BDQ QD for 2 weeks, then BDQ TIW. Participants in Arm 4 will receive RHEZ QD for 8 weeks followed by rifampin and isoniazid for 18 weeks per the local standard of care.

[n]For participants randomized to Arm 1, Arm 2, and Arm 3, PK samples will be taken on Day 1 (predose and postdose), and then Week 2 (predose), Week 4 (predose), Week 8 (predose and postdose), Week 13 (predose), and Week 17/ET (predose and postdose) (9 samples per participant). The PK samples will be drawn based on the DLM dosing for Arm 1, Arm 2, and Arm 3.

[o]For HIV positive participants who are included, HIV testing does not need to be repeated at screening if the HIV positive status has been documented prior to screening (within the last year) and the documentation can be kept with the trial documentation for the required duration. For participants who are HIV positive, blood samples are to be drawn for CD4 and viral load assessment at the local laboratory.

2 Introduction

Tuberculosis (TB) remains a major global health problem and is a leading cause of death from an infectious disease worldwide.[1] Progress has been made in the research and development of new anti-tuberculosis drugs to help overcome the current TB treatment situation. In recent years, bedaquiline (BDQ) (Sirturo™) and the sponsor's delamanid (DLM) (Deltyba™) have been used as part of a combination therapy in adults with pulmonary multidrug-resistant tuberculosis (MDR-TB). These 2 new drugs offer the opportunity to form the backbone for a novel oral anti-TB drug regimen that can shorten the treatment for both drug-susceptible tuberculosis (DS-TB) and MDR-TB.[1,2,3] Thus, there is an urgent need for the development of new potent anti-TB agents with low toxicity that are effective against drug-susceptible and drug-resistant strains of *Mycobacterium tuberculosis* and can be combined with BDQ and DLM.[4]

Otsuka Pharmaceutical Co, Ltd (Otsuka) initiated a program to screen for new anti-TB agents that can be used in combination with DLM. As a result of research efforts, Otsuka discovered OPC-167832, a novel 3, 4 dihydrocarbostyril derivative. OPC-167832 is an inhibitor of decaprenylphosphoryl-β-D-ribose 2'-oxidase (DprE1), an essential enzyme for *M tuberculosis* cell wall biosynthesis. The minimum inhibitory concentrations (MIC) of OPC-167832 for *M tuberculosis* on clinically isolated strains, including MDR-/extensively drug-resistant-TB, ranged from 0.00024 to 0.002 µg/mL. It has bactericidal activity against both growing and intracellular bacilli. The frequency of spontaneous resistance of OPC-167832 was 2.60×10^{-9} to 1.52×10^{-7} for *M tuberculosis* H37Rv at 16 × MIC.

Please refer to the OPC-167832 Investigator's Brochure (IB) for more detailed information.[5]

2.1 Trial Rationale

In vitro and in vivo studies have shown that OPC-167832 has potent bactericidal activity.[5] Further, OPC-167832 in combination with DLM or BDQ may have additive effects on bactericidal activity. No antagonistic activity has been shown with OPC-167832 and DLM or OPC-167832 and BDQ. Because of OPC-167832's high bactericidal activity, and novel mechanism of action (different from all other TB drugs currently available for human use), it is likely a good candidate to be included in a regimen to possibly improve efficacy and prevent resistance development to concomitant TB drugs.[5]

Single oral doses up to 480 mg OPC-167832 were well tolerated in healthy participants; there were no serious or severe adverse events (AEs) and no participants were discontinued from the trial due to a treatment emergent AE. One participant did decide to withdraw consent after occurrence of the nonserious and mild AEs of arthralgia, pain in extremity, and ecchymosis.

The safety, tolerability, pharmacokinetics (PK), and efficacy of OPC-167832 are being evaluated in adult participants with uncomplicated, smear-positive, pulmonary DS-TB, in an ongoing, multiple-dose trial. Stage 1, which was testing multiple ascending doses of OPC-167832 has been completed. Clinically meaningful early bactericidal activity as measured by change from baseline at 14 days in Log10 colony-forming unit (CFU) was seen across all doses of OPC-167832 tested. OPC-167832 demonstrated an average decline of 0.101, 0.138, 0.151, and 0.149 Log10 CFU/mL per day in sputum specimens obtained from participants administered with 3 mg, 10 mg, 30 mg, and 90 mg per day, respectively, versus 0.200 Log10 CFU/mL per day for the rifampin, isoniazid, ethambutol, and pyrazinamide (RHEZ) arm. There was a single serious adverse event (SAE) of haemoptysis that led to discontinuation of investigational medicinal product (IMP) (3 mg dose); however, this SAE was not considered to be related to the IMP.

The proposed treatment regimen in this trial has not yet been evaluated in a clinical trial but is supported by the clinical and nonclinical data on DLM, BDQ, and OPC-167832. In a mouse chronic TB model, the 3-drug combination of OPC-167832, DLM, and BDQ significantly killed *M tuberculosis* faster than the standard care of treatment regimen of RHEZ[4] and is more efficacious in a hollow fiber system of tuberculosis (HFS-TB). In the HFS-TB, the 3-drug combination sterilized the system much faster than the standard treatment regimen and suggested a potential 3 to 4 months treatment regimen.

This trial will evaluate the safety, tolerability, pharmacokinetics (PK), and efficacy of 2 doses of OPC-167832 combined with DLM and BDQ in participants who are ≥ 18 to ≤ 65 years old with pulmonary DS-TB. Standard-of-care treatment with RHEZ will be included for comparison.[6]

2.2 Background

Delamanid and BDQ are 2 of the most recently developed anti-TB drugs that have been extensively studied in patients with MDR-TB, and have received regulatory approval and World Health Organization endorsement to be used as part of an optimal background regimen for patients with MDR-TB. Delamanid is a nitro-dihydro-imidazooxazole derivative that inhibits the synthesis of mycolic acid, an important component of the *M tuberculosis* cell wall. Bedaquiline is a diarylquinoline derivative that inhibits

M tuberculosis adenosine triphosphate synthase. Both DLM and BDQ have been studied together as part of a National Institutes of Health-sponsored combination drug-drug interaction/safety trial (NCT02583048).

OPC-167832 is a newly discovered 3,4-dihydrocarbostyril derivative that is an inhibitor of DprE1, an essential enzyme for *M tuberculosis* to synthesize lipoarabinomannan (LAM) and arabinogalactan (key components of the *M tuberculosis* cell wall). OPC-167832 is currently under clinical development: a single ascending dose trial in healthy participants (Trial 323-201-00001) has been completed, and a multiple, ascending-dose and early bactericidal activity trial in participants with DS-TB has completed Stage 1 and is ongoing (Trial 323-201-00003).

These 3 drugs have distinct mechanisms of actions against *M tuberculosis* that are also distinct from other approved TB drugs (one notable exception is pretomanid, which has a similar mechanism of action and likely near complete cross-resistance to DLM).

2.3 Known and Potential Risks and Benefits

2.3.1 OPC-167832

In a 1-week repeated-dose oral toxicity study in rats, slightly suppressed body weight gain and slightly decreased food consumption were observed. In blood biochemistry, increases in total cholesterol and phospholipids and decreases in creatinine were observed.

In 1-, 4-, and 26-week toxicity studies in rats, no drug-related deaths occurred in either males or females at any dose tested. Suppressed body weight gain and/or decreased food consumption were observed and increases in total cholesterol and phospholipids and decreases in creatinine were observed at 1 week. In the 4-week repeated-dose oral toxicity study in rats, suppressed body weight gain and decreased food consumption were observed. These changes showed reversibility or a tendency for reversibility. The no observed adverse effect level (NOAEL) was estimated to be 2000 mg/kg/day for males and 200 mg/kg/day for females. At the NOAEL, the maximum (peak) plasma concentration (C_{max}) and area under the concentration-time curve from zero to 24 hours (AUC_{24h}) of OPC-167832 in Week 4 (on Day 28) were respectively 6.863 µg/mL and 96.91 µg·h/mL in males and 9.205 µg/mL and 130.7 µg·h/mL in females. In a 1-week repeated-dose oral toxicity study in dogs, decreased body weight or suppressed body weight gain, or both, as well as relatively low food consumption were observed.

In 1-, 4-, 13- and 39-week toxicity studies in dogs, no treatment related deaths occurred in either males or females at any dose tested. Increases in creatinine kinase, alanine

aminotransferase (ALT), aspartate aminotransferase (AST), lactate dehydrogenase, and potassium were observed in the 4-week toxicity study at the highest dose (2000 mg/kg/day). Increases in total cholesterol and phospholipids were also observed in the 4- and 13-week toxicity studies at doses of 100 mg/kg/day and higher. In the 4-week toxicity study, slight prolongation of QT interval and QT interval corrected (QTc) was observed at Week 4 in both sexes at 2000 mg/kg/day. However, no prolongation of QT interval and QTc was noted in the 13-week toxicity study in either sex at up to 100 mg/kg.

In 4- and 13-week repeated-dose oral toxicity studies in dogs, decreased body weight or suppressed body weight gain, or both, and decreased food consumption were observed. In blood biochemistry, changes in creatinine kinase, lactate dehydrogenase, alanine aminotransferase, aspartate aminotransferase, total cholesterol, phospholipids, α_1-globulin content and ratio, potassium, and myoglobin were observed. In electrocardiography, a slight prolongation of QT interval and QTc was observed only at the highest dose in the 4-week repeated-dose oral toxicity study. In histopathological examination, changes in the muscle fibers were observed. Stress-related changes secondary to body weight loss were observed in the thymus and adrenal grand. These changes showed reversibility or a tendency for reversibility. The NOAEL was 3 mg/kg in beagle dogs in the 13-week toxicity study.[5]

Single oral doses up to 480 mg OPC-167832 were well tolerated during Trial 323-201-00001; there were no serious or severe AEs but 1 participant withdrew consent after occurrence of the nonserious and mild AEs of arthralgia, pain in extremity, and ecchymosis.[5] In the single ascending dose trial of OPC-167832 (Trial 323-201-00001), where single oral doses up to 480 mg were administered to healthy participants, a slight negative correlation was observed between changes in QT interval corrected by Fridericia's method (QTcF) from baseline and placebo, and OPC-167832 plasma concentrations.

The safety, tolerability, PK, and efficacy of OPC-167832 are being evaluated in adult participants with uncomplicated, smear-positive, DS-TB, in an ongoing, multiple-dose trial (Trial 323-201-00003). Stage 1, which was testing multiple ascending doses of OPC-167832 has been completed. Clinically meaningful early bactericidal activity as measured by change from baseline at 14 days in Log10 CFU was seen across all doses of OPC-167832 tested. There was a single SAE of haemoptysis that led to discontinuation of IMP (3 mg dose); however, this SAE was not considered to be related to the IMP.

Trial sites will receive updated versions of the IB, when available, and trial sites should refer to the most current version as needed.

2.3.2 Delamanid

The first marketing approval for DELTYBA® (DLM) was granted on 28 Apr 2014 in Europe (Austria, Belgium, Bulgaria, Croatia, Cyprus, Czech Republic, Denmark, Estonia, Finland, France, Germany, Greece, Hungary, Iceland, Ireland, Italy, Latvia, Liechtenstein, Lithuania, Luxembourg, Malta, Netherlands, Norway, Poland, Portugal, Romania, Slovakia, Slovenia, Spain, Sweden), and United Kingdom, followed by approvals in Japan (04 Jul 2014), South Korea (29 Oct 2014), Hong Kong (29 Mar 2016), Turkey (31 May 2017), India (24 Aug 2017), the Philippines (08 Sep 2017), China (12 Mar 2018), Mongolia (05 Dec 2018), Ukraine (30 Jan 2019), South Africa (25 Mar 2019), Indonesia (05 Apr 2019), Peru (01 Jun 2019), Kazakhstan (08 Aug 2019), Turkmenistan (22 Sep 2019), Russia (08 May 2020), and Azerbaijan (26 Jan 2021). In all completed and ongoing clinical trials of DLM, as of 31 Jan 2019, including healthy participants and participants with TB (uncomplicated DS-TB, MDR-TB, and MDR TB refractory to treatment), 1419 participants received at least 1 dose of DLM.

In the 14 completed trials in healthy participants, the treatment-emergent adverse events (TEAEs) that led to the discontinuation of the IMP (ie, DLM) in more than 1 participant in the Any DLM group were rash generalised (12 participants), haematochezia (3 participants), and headache (3 participants). There were 2 serious TEAEs, delirium and ischaemic colitis, both occurring in Trial 242-07-209. No deaths were reported in any of the completed trials in healthy participants.

In the 2 completed trials of participants with uncomplicated DS-TB treated with DLM, there were no deaths, and no participants discontinued IMP due to TEAEs. There were 5 serious TEAEs (3 events of prolonged electrocardiogram (ECG) QT interval and 2 events of increased transaminases), all occurring in Trial 242-06-101.

In the 4 completed trials of participants with MDR-TB, the only TEAE that led to the discontinuation of IMP in more than 1 participant in the DLM + optimized background regimen (OBR) group was prolonged ECG QT interval (7 participants in the DLM + OBR group and 2 participants in the placebo + OBR group). The serious TEAEs that occurred in at least 1% of participants in the DLM + OBR group and at a higher incidence than in the placebo + OBR group were prolonged ECG QT interval (3.4% DLM + OBR, 1.2% placebo + OBR), TB (2.1% DLM + OBR, 0.9% placebo + OBR), and hypokalaemia (1.6% DLM + OBR, 0.9% placebo + OBR).

Additional information regarding the safety and the potential risks and benefits of DLM is provided in the IB.[3]

2.3.3 Bedaquiline

Bedaquiline (SIRTURO®) is a diarylquinoline antimycobacterial drug indicated as part of combination therapy in adult and pediatric patients (≥ 5 years and weighing ≥ 15 kg) with pulmonary MDR-TB. For the known and potential risks of SIRTURO, please see the package insert for SIRTURO (BDQ) tablets.[2]

2.3.4 Rifampin, Isoniazid, Ethambutol, and Pyrazinamide

RIFAFOUR® tablets are a combination of four first line agents (RHEZ) used in the treatment of TB. Rifampin is a semi-synthetic, broad-spectrum bactericidal antibiotic. Isoniazid is a synthetic, antitubercular agent which is bacteriostatic against semi-dormant bacilli and bactericidal against actively dividing mycobacteria. Pyrazinamide may be bactericidal or bacteriostatic, depending on its concentration and the susceptibility of the organism. Ethambutol is a synthetic, bacteriostatic antitubercular agent. All agents are readily absorbed following oral administration, with wide distribution to most tissues and fluids including cerebrospinal fluid. For the complete list of known and potential risks of RHEZ, please see the package insert for RIFAFOUR single-dose combination tablets.[6]

3 Objectives and Endpoints

Table 3-1 Trial Objectives and Endpoints

Objectives	Endpoints
Primary:<br>• To assess the safety and efficacy of OPC-167832 combined with DLM and BDQ in participants with DS-TB administered for 4 months compared to RHEZ administered for 6 months	Safety:<br>• AEs, clinical laboratory tests, vital signs, physical examinations, ECGs<br>• The proportion of participants with a grade 3 or higher AE<br>• The rate of all-cause treatment discontinuation<br><br>Efficacy:<br>• The proportion of participants achieving sputum culture conversion (SCC) in Mycobacteria Growth Indicator Tube® (MGIT) by the end of the treatment period

Table 3-1 Trial Objectives and Endpoints

Objectives	Endpoints
Secondary:<br>• To evaluate efficacy in sputum MGIT culture and LAM for OPC-167832 combined with DLM and BDQ administered for 4 months as compared to RHEZ<br>• To evaluate the development of resistance to anti-TB drugs in all treatment arms<br>• To inform dose selection of OPC-167832 combined with DLM and BDQ	Efficacy:<br>• The proportion of participants who achieve SCC in MGIT by 8 weeks of treatment<br>• Time to detection of MGIT cultures<br>• The proportion of participants who convert sputum LAM to negative by 8 weeks of treatment and by end of treatment<br>• Proportion of participants who develop drug resistance<br>• Time to SCC of OPC-167832, DLM, and BDQ, as well as RHEZ
Exploratory Biomarker Analysis:<br>• To evaluate various novel biomarkers for association with microbiological and clinical response	Efficacy:<br>• Longitudinally assess positron emission tomography/computerized axial tomography (PET/CT) imaging response over the course of treatment<br>• Longitudinally evaluate the ribosomal ribonucleic acid synthesis ratio (RS ratio) decline in sputum<br>• Longitudinally assess whole blood transcriptomic signatures previously associated with TB cure from serum
CCI [redacted]	

Section 9.4 describes the statistical analysis of the endpoints.

4 Trial Design

4.1 Type/Design of Trial

Trial 323-201-00006 is a multicenter, phase 2b/c, open-label, randomized, dose-finding trial in participants with DS-TB. The microbiology laboratory will be blinded to treatment assignments.

Following a screening period of up to 14 days, eligible participants will be randomized on Day −1 or predose Day 1 in a ratio of 1:2:2:1 to one of the following treatment arms:

- Arm 1: DLM (300 mg once daily [QD]) + BDQ (400 mg QD x 2 weeks, then 200 mg thrice-weekly [TIW]) + OPC-167832 (10 mg QD) for 17 weeks
- Arm 2: DLM (300 mg QD) + BDQ (400 mg QD x 2 weeks, then 200 mg TIW) + OPC-167832 (30 mg QD) for 17 weeks
- Arm 3: DLM (300 mg QD) + BDQ (400 mg QD x 2 weeks, then 200 mg TIW) + OPC-167832 (90 mg QD) for 17 weeks
- Arm 4: RHEZ for 8 weeks followed by 18 weeks of rifampin and isoniazid (for a total of 26 weeks)

Randomization will be stratified by HIV status (yes or no) and presence of bilateral cavitation on screening chest x-ray (yes or no). At the end of the treatment period, participants will be followed until 12 months postrandomization.

4.2 Scientific Rationale for Trial Design

Treatment of TB requires multiple drugs with different mechanisms of action and the ability to kill *M tuberculosis* bacilli within heterogeneous TB lesions (ie, different pathophysiologic environments) in order to ensure rapid sterilization of lesions, a durable relapse-free cure, and prevent the development of resistance. Development of novel TB treatment regimens that are comprised of potent new TB drugs that can shorten the treatment duration to 4-months or less will simplify TB treatment, enhance compliance, and potentially improve treatment outcomes.

This trial will be conducted to evaluate a new proposed treatment regimen that is supported by the clinical and nonclinical data on DLM, BDQ, and OPC-167832. In vitro and in vivo nonclinical data demonstrate that the proposed 3-drug combination is more efficacious than the current 6-month standard treatment in a mouse model and a HFS-TB. Importantly, HFS-TB data suggest that the 3-drug combination has the potential to cure TB in 2 to 3 months. Furthermore, the drug exposures used in these studies are generally in accordance with those that can be achieved with the proposed human doses.

Using the HFS-TB model, the 3-drug combination of DLM+BDQ+OPC-167832 killed TB bacilli faster than any of the 2-drug combinations as well as the standard therapy (RHEZ), in either the low pH or log-phase culture conditions. Unexpectedly, the DLM and BDQ 2-drug combination not only did not kill bacteria but paradoxically appeared to enhance bacterial growth (this phenomenon was confirmed in separate experiments). Currently, there is no explanation for this observation, but is likely unique to the HFS-TB, since the DLM and BDQ combination was additive in a mouse study and indifferent to additive in the in vitro checkerboard assay. Nevertheless, when OPC-167832 was added to the combination of DLM and BDQ, the combination became very effective. Using a model developed to convert the HFS-TB data to TB treatment duration in patients, the 3-drug regimen is predicted to cure DS-TB patients in 2 to 3 months.

This trial will evaluate the safety, tolerability, PK, and efficacy of OPC-167832 combined with DLM and BDQ in participants with DS-TB. Standard-of-care treatment with RHEZ will be included for comparison.

4.3 Dosing Rationale

A key goal of the trial is to determine if the combination of DLM, OPC-167832, and BDQ is efficacious, safe and can potentially shorten the treatment duration in patients with DS-TB. In order to maximize the chances of success in achieving this goal, doses were chosen such that they can provide the highest possible exposures of the drugs without compromising patient safety. Since OPC-167832 has only been evaluated in the 14-day early bactericidal activity (EBA) trial in participants with TB, and long-term efficacy and safety are not available, 3 doses of this compound will be evaluated in this trial. Both doses are selected to achieve near maximal efficacy based on the pharmacokinetics/pharmacodynamics (PK/PD) analyses of the EBA trial. Delamanid QD dosing regimen in combination with QD dosing of OPC-167832 and BDQ is being proposed in this trial due to the need to facilitate patient adherence.

Delamanid exposure-response modeling using DLM steady state area under the concentration-time curve (AUC) and change from baseline in CFU found cumulative fraction of response (CFR) of 96 to 98% and 89 to 96% following the approved DLM dose of 100 mg twice daily (BID) (from Trial 242-07-204) and the 200 mg QD dose regimen (from Trial 242-09-213), respectively. Following dosing of DLM 300 mg QD (from Trials 242-06-201 and 242-08-211), DLM AUC were about 20 to 25% higher than those obtained following 200 mg QD and hence correspondingly, the CFR also would be higher than that of the 200 mg QD dose. From a safety perspective, the exposures following 300 mg QD were lower than that following the approved 100 mg BID dosing,

indicating that the 300 mg QD dose would likely have less of an effect on a key safety issue, QT interval prolongation, compared to the approved 100 mg BID dose. Hence the dose of 300 mg QD DLM has been selected for the trial.

In the current multiple ascending dose (MAD)/EBA trial of OPC-167832 (Trial 323-201-00003), doses of 3 mg, 10 mg, 30 mg, and 90 mg have been studied. An exposure-response analysis of OPC-167832 of data obtained to date from the MAD/EBA trial indicated that the effect of 10 mg, 30 mg and 90 mg doses on CFU reduction were similar while the effect from the 3 mg dose is slightly less (ie, CFU count). All 4 doses were efficacious and well tolerated with no significant safety concerns. Because the efficacy and safety of long-term use in combinations with other TB drugs of OPC-167832 are unknown, in order to maximize exposures, and at the same time have a reasonable safety margin, the 10 mg, 30 mg, and 90 mg doses of OPC-167832 were chosen for the trial.

4.4 End of Trial Definition

The end of trial date is defined as the last date of contact or the date of final contact attempt from the post-treatment follow-up electronic case report form (eCRF) page for the last participant completing or withdrawing from the trial.

4.5 Definition of Completed Participants

The treatment period is defined as the time period during which participants are evaluated for primary and/or secondary objectives of the trial irrespective of whether or not the participant actually took all doses of the IMP. For the purpose of this trial, treatment completers will be randomized participants who take IMP through either Week 17 (for Arm 1, Arm2, and Arm 3) or Week 26 (for Arm 4) and complete at least some of the end-of-treatment assessments. Trial completers will be randomized participants who remain on the study till the 12 months post-randomization visit.

Completer categories are as follows: (1) Treatment completer and trial completer (2) Treatment non-completer and trial completer (3) Treatment completer, trial non-completer and (4) Treatment non-completer and trial non-completer.

Further details can be found in Section 10.6.

5 Trial Population

The trial population will consist of participants who are ≥ 18 years to ≤ 65 years at screening and who have newly diagnosed pulmonary DS-TB. Approximately 120 participants (including no more than 15% with HIV-coinfection) will be enrolled at approximately 8 sites in South Africa.

5.1 Participant Selection and Numbering

All participants will be given a unique participant identifier (identification [ID]; site number [3 digits] + participant number ['S' + 5 digits] upon providing consent). The site number will be designated by the sponsor. Participant's numbers will be assigned sequentially across the trial from S00001.

Demographic information (collection date, date of birth, sex, childbearing potential, race, ethnicity, country) and medical history will be recorded in the eCRF at the screening visit.

5.2 Eligibility Criteria

Exceptions for eligibility criteria will not be permitted during the trial, neither by the investigator nor by the medical monitor.

5.2.1 Inclusion Criteria

Participants are required to meet the following inclusion criteria when assessed:

1) Able to provide written, informed consent prior to initiation of any trial-related procedures or treatments, and able, in the opinion of the investigator, to comply with all the requirements of the trial.
2) Male or female participants between 18 and 65 years of age (inclusive) at the screening visit.
3) Body weight ≥ 35.0 kg at the screening visit.
4) Newly diagnosed, rifampin and isoniazid susceptible (on the screening sample) pulmonary TB.
5) Able to spontaneously produce sputum.
6) Females of childbearing potential (FOCBP) must agree to use 2 different approved methods of birth control or remain abstinent throughout the participation in the trial and for 12 weeks after the last dose of IMP or dose of RHEZ.
7) Male participants must agree to use 2 different approved methods of birth control or remain abstinent throughout the participation in the trial and for 12 weeks after the last dose of IMP or RHEZ.

5.2.2 Exclusion Criteria

Participants will be excluded if they meet any of the following exclusion criteria when assessed:

1) Participants are known or suspected of having resistance to rifampin, isoniazid, ethambutol, pyrazinamide, DLM, or BDQ either confirmed by the laboratory, or based on epidemiological history, at screening.

2) Evidence of clinically significant metabolic (for example, including ongoing or current hypokalemia [ie, potassium <3.5 mEq/dL at screening]), gastrointestinal, neurological, psychiatric, endocrine or liver (eg, hepatitis B and C) disease; malignancy; or other abnormalities (other than the indication being studied).
3) History of, or current, clinically relevant cardiovascular disorder such as heart failure, coronary heart disease, uncontrolled hypertension, arrhythmia or symptom strongly suggestive of such a problem (for example, syncope or palpitations), tachyarrhythmia or status after myocardial infarction.
4) Known bleeding disorders or family history of bleeding disorders.
5) Any diseases or conditions in which the use of DLM, BDQ, OPC-167832, rifampin, isoniazid, pyrazinamide, or ethambutol is contraindicated.
6) Any prior treatment for *M tuberculosis* within the past 2 years.
7) Any treatment with a drug active against *M tuberculosis* (eg, quinolones) within the 3 months prior to screening.
8) Clinical evidence of severe extrapulmonary TB (eg, miliary TB, abdominal TB, urogenital TB, osteoarthritic TB, TB meningitis).
9) Evidence of pulmonary silicosis, lung fibrosis, or other lung condition considered as severe by the investigator (other than TB). In particular, any underlying condition that could interfere with the assessment of x-ray images, sputum collection, or interpretation of sputum findings, or otherwise compromise the subject's participation in the trial.
10) Any renal impairment characterized by creatinine clearance/estimated glomerular filtration rate (eGFR) of < 60 mL/min/1.73 m^2, or hepatic impairment characterized by alanine transaminase or aspartate transaminase > 2.0 × upper limit of normal of the clinical laboratory reference range or bilirubin > 2.0 × upper limit of normal of the clinical laboratory reference range, at screening.
11) Screening glucose (nonfasting) ≥ 200 mg/dL or glycosylated hemoglobin (HbA1c) ≥ 6.5%.
12) QTcF > 450 msec in male participants (> 470 msec in female participants), atrioventricular block II or III, bi-fasicular block, at screening or current or history of clinically significant ventricular arrhythmias. Other ECG abnormalities, if considered clinically significant by the investigator.
13) Participants receiving any of the prohibited medications (see Section 6.5.1) within the specified periods or who would be likely to require prohibited concomitant therapy during the trial.
14) Female participants who are breast-feeding or who have a positive pregnancy test result prior to receiving the first dose of IMP or RHEZ on Day 1.
15) Current history of significant drug and/or alcohol abuse that is likely to result in poor adherence to trial requirements or that would pose a risk to the participant's well-being during the course of the trial.
16) History of current hepatitis or carriers of hepatitis B surface antigen (HBsAg) and/or anti hepatitis C virus (HCV).

17) Participants who test positive for cocaine or other drugs of abuse (excluding known prescription stimulants and other prescribed medications and marijuana) at screening are excluded. Detectable levels of alcohol, marijuana, barbiturates, or opiates in the drug screen are not exclusionary if, in the investigator's documented opinion, the participant does not meet Diagnostic and Statistical Manual of Mental Disorders, Fifth Edition criteria for moderate to severe substance use disorder and the positive test does not signal a clinical condition that would impact the safety of the participant or interpretation of the trial results, and participation is agreed to by the medical monitor prior to treatment.
18) History of having taken an investigational drug within 30 days preceding trial entry.
19) A history of difficulty in donating blood.
20) Donation of blood or plasma within 30 days prior to dosing.
21) History of serious mental disorders that, in the opinion of the investigator, would exclude the participant from participating in this trial.
22) Any known prior exposure to OPC-167832, DLM, or BDQ.
23) Participants with significant medical comorbidities that in the opinion of the investigator, should not participate in the trial.
24) Participants with Karnofsky score < 60 will be excluded from the trial.
25) Participants testing positive for active severe acute respiratory syndrome coronavirus (SARS-CoV-2) infection at screening.
26) Participants with HIV co-infection not on a stable anti-retroviral regimen consisting of tenofovir, emtricitabine/ lamivudine, dolutegravir (ie > 3 months), or who have a detectable viral load, or who have a CD4 count < 350 cells/mm^3 will be excluded from the trial.

A definition of childbearing potential can be found in Section 10.3.

Participants must agree to restrictions to lifestyle and medications described in Section 5.3 and Section 6.5.1, respectively.

5.3 Lifestyle Considerations

Not applicable.

5.3.1 Meals and Dietary Restrictions

Consumption of grapefruit, grapefruit products, Seville oranges, or Seville orange products within 72 hours prior to the first dose of IMP (ie, DLM, OPC-167832, or BDQ) or RHEZ and during the trial is prohibited.

5.3.2 Caffeine, Alcohol, and Tobacco

Participants will be encouraged to refrain from drinking alcoholic beverages or using illicit drugs during participation in the trial.

5.4 Screen Failures

Participants who sign an informed consent form (ICF) but who are not started on treatment are permitted to be rescreened. In the event that the participant is rescreened for trial participation, and the rescreening was not completed within the original screening window, a new ICF must be signed. Participants excluded for positive drug/alcohol screen are not eligible to be rescreened for participation in the trial. However, participants excluded for other reasons may be rescreened at any time if the exclusion characteristic has changed. In the event that the participant is rescreened a new ICF must be signed, a new screening number assigned, and all screening procedures repeated.

A screen failure is a participant from whom informed consent is obtained and is documented in writing (ie, participant signs an ICF), but who is not randomized or assigned trial treatment. All AEs must be reported after participant informed consent has been obtained, including screening failures due to AEs, irrespectively of IMP administration.

If the participant meets the definition of a screen failure in this trial, the following information will be recorded in the eCRF:

- Date of informed consent
- Visit date (screening visit)
- Demographics (collection date, birth date, sex, race, ethnicity, country)
- Screen failure date
- Reason for screen failure

6 Trial Treatments

6.1 Trial Treatments Administered

Treatment Arms 1, 2, and 3:

All IMP (ie, OPC-167832, DLM, or BDQ) must be administered after the completion of a meal.

Bedaquiline should be administered as follows: 400 mg QD for the first 2 weeks of treatment and 200 mg TIW thereafter with at least 48 hours between doses for the remainder of the trial (ie, a total of 17 weeks [Arm 1 or Arm 2 or Arm 3] of treatment).

IMP will be administered using the locally accepted adherence monitoring techniques which could include directly observed therapy (DOT), Wisepill, video DOTs, or other adherence monitoring techniques. In addition, all doses of IMP given during trial visits will be observed via the hand-and-mouth procedure. IMP will be administered with approximately 240 mL of still (noncarbonated) water.

Treatment Arm 4:

Participants randomized to treatment Arm 4 will receive RHEZ orally QD for 8 weeks followed by 18 weeks of rifampin and isoniazid per the local standard of care.

RHEZ will be administered using the locally accepted adherence monitoring techniques which could include DOT, Wisepill, video DOTs, or other adherence monitoring techniques. In addition, all doses of RHEZ given during trial visits will be observed via the hand-and-mouth procedure. RHEZ will be administered with approximately 240 mL of still (noncarbonated) water. The RHEZ will be taken 1 hour prior to or 2 hours after a meal.

For information regarding the dose regimen and treatment period(s), including any follow-up period(s) for each treatment group/arm of the trial, see Section 4.1.

6.1.1 Medical Devices

Not applicable.

6.2 Management of Investigational Medicinal Product

The IMPs to be administered during this trial include OPC-167832, DLM, and BDQ.

The reference product to be administered is RHEZ (RIFAFOUR e-275 tablets) and rifampin/isoniazid.

For full details on IMP management, please refer to the OPC-167832 and DLM IBs and BDQ prescribing information.

6.2.1 Packaging and Labeling

Investigational medicinal product will be provided by the sponsor or designated agent to the investigators and the persons designated by the investigator(s) or institution(s). The IMP will be supplied as packages. OPC-167832 or DLM may not be repackaged by the trial site.

OPC-167832 and DLM will be provided to the investigator(s) and the persons designated by the investigator(s) or institutions by the sponsor or designated agent.

OPC-167832 and DLM will be supplied as follows:

- OPC-167832 will be supplied as 10-mg or 30-mg immediate-release tablets
- DLM will be supplied as 50-mg film-coated tablets

Each bottle or carton of OPC-167832 or DLM provided by the sponsor will be labeled to clearly disclose the compound identity, protocol number, the sponsor's name and address, instructions for use, route of administration, appropriate precautionary statements, and other information required by local regulatory authorities.

Both BDQ and RHEZ (RIFAFOUR single dose combination tablets) will be sourced in country.

6.2.2 Storage

The IMP will be stored in a securely locked cabinet or enclosure. Access will be limited to investigators and their designees. Neither the investigator(s) nor any designees may provide IMP or RHEZ to any participant not participating in this protocol.

The IMP and RHEZ will be stored according to the storage conditions indicated on the clinical labels.

The clinical trial site staff will maintain a temperature log in the drug storage area recording the temperature at least once each working day.

6.2.3 Accountability

The investigator or designee must maintain an inventory record of IMP and RHEZ received, dispensed, administered, and returned.

6.2.4 Returns and Destruction

Upon completion or termination of the trial, all used IMP containers, unused IMPs, and partially-used IMPs must be destroyed at the trial site(s).

The assigned trial monitor will facilitate the destruction of used IMP containers, unused IMPs, and partially-used IMPs. The trial site(s) may utilize qualified third-party vendors for IMP destruction. A certificate of destruction should be filed within the IMP accountability.

6.2.5 Reporting of Product Quality Complaints

A Product Quality Complaint (PQC) is any written, electronic, or oral communication provided by a healthcare professional, clinical trial participant, medical representative, regulatory agency, partner, or other third party that alleges deficiencies related to the

identity, quality, durability, reliability, safety, or performance of a medical device or medicinal product after it is released for distribution.

Examples include, but are not limited to, communications involving:

- Failure of a product to meet any of its specifications.
- Packaging defects (eg, damaged, dirty, crushed, missing product or component, incorrect or missing labeling).
- Product defects (eg, odor, chipped, broken, damaged, crushed, embossing illegible, under-filled bottle, over-filled bottle, empty bottle, no safety seal).
- Loss or theft of product.

6.2.5.1 Eliciting and Reporting Product Quality Complaints

The investigator or designee must record each PQC identified through any means from the receipt of the sponsor IMP (ie, DLM or OPC-167832) from the sponsor or sponsor's designee, through and including reconciliation and up to destruction, including participant dosing. The investigator or designee must notify the sponsor (or sponsor's designee) by email within 24 hours of becoming aware of the PQC according to the procedure outlined below.

- Send PQC reporting information to the Otsuka Pharmaceutical Development & Commercialization, Inc. IMP complaints mailbox email: IMP-PQC@otsuka-us.com.

Also indicate whether or not the complaint sample is available for return.

Identification of a PQC by the participant should be reported to the site investigator, who should then follow the reporting mechanism above.

The investigator should report PQCs in accordance with respective manufacturing authorization holders for nonOtsuka products (per package label).

6.2.5.2 Information Required for Reporting Purposes

- Description of complaint
- Reporter ID (eg, investigator, site, etc)
- Reporter contact information (eg, address, phone number, email address)
- Subject number
- Clinical site number
- ID of material (product/compound name, lot/batch number, shipment number, expiry date)
- Clinical protocol reference (number and/or trial name)
- Dosage form/strength (if known)

- Pictures of complaint sample (if available)
- Availability of complaint sample for return

6.2.5.3 Return Process

Indicate during the report of the PQC if the complaint sample is available for return. If the complaint sample is available for return, the sponsor will provide return instructions, when applicable.

If the complaint sample is available but not at the clinical site, please instruct the participant to bring the complaint sample to their next site visit.

It must be documented in the site accountability record that the complaint sample has been forwarded to the sponsor for complaint investigation.

6.2.5.4 Assessment/Evaluation

Assessment and evaluation of PQCs will be handled by the sponsor. The investigator should report PQCs in accordance with the respective manufacturing authorization holders for nonOtsuka products (per package label).

6.3 Measures to Minimize/Avoid Bias

This is an open-label trial; however, the microbiology laboratory will be blinded to treatment assignments. The Miettinen and Nurminen confidence interval (CI) of common risk difference stratified by HIV status and cavitation status prior to randomization will be used to reduce bias in participant allocation to treatment groups.[7]

6.4 Participant Compliance

The time and dose of IMP and RHEZ administration during the trial will be recorded on the eCRF. Information regarding any inappropriately administered doses will also be documented on the eCRF. A Medication Event Reminder Monitor System will also be used to measure compliance.

6.5 Concomitant Medications or Therapies

The investigator will record all medications (including prescription medications, vaccines, over-the-counter medications, herbal remedies, etc) and therapies taken by the participant from 30 days prior to signing of informed consent through the end of the evaluation period (defined as the time period during which participants are evaluated for primary and/or secondary objectives) in the eCRF. The investigator will also record all medications and therapies taken by the participant for treatment of an AE or which

caused an AE until the end of the trial (defined as the last date of contact or date of final contact attempt) in the eCRF.

For concomitant medications, the following will be recorded in the eCRF: medication, indication, dose, frequency, route, start date and end date.

6.5.1 Prohibited Medications or Therapies

Quinolones should not be used within 3 months prior to the screening visit and during the screening period. The only antiretroviral therapy allowed for this trial is tenofovir, emtricitabine/ lamivudine, dolutegravir. Furthermore, participants must be on a stable antiretroviral regimen for at least 3 months prior to screening.

The selected cytochrome P450 (CYP)3A4 inhibitors and inducers presented in Table 6.5.1-1 and Table 6.5.1-2, respectively should not be used from Day −2 until the end of treatment.

Table 6.5.1-1 List of Selected Cytochrome P450 3A4 Inhibitors Prohibited During the Trial

**Potent CYP3A4 Inhibitors:**	
Viekira PAK2	Itraconazole
Tipranavir	Mibefradil
Ritonavir	Clarithromycin
Cobicistat	Nelfinavir
Ketoconazole	Nefazodone
Troleandomycin	Idelalisib
Telaprevir	Boceprevir
Posaconazole	Voriconazole
Telithromycin	
Grapefruit juice	
Conivaptan	
**Moderate CYP3A4 Inhibitors:**	
Erythromycin	Faldaprevir
Fluconazole	Imatinib
Atazanavir	Verapamil
Diltiazem	Netupitant
Darunavir	Nilotinib
Dronedarone	Tofisopam
Crizotinib	Cyclosporine
Atazanavir	Ciprofloxacin
Aprepitant	Isavuconazole
Casopitant	Cimetidine
Amprenavir	

Note: Please communicate with the sponsor's medical monitor if the participant needs to receive any medication (other than IMP or RHEZ) that is not on this list.

Table 6.5.1-2 List of Selected Cytochrome P450 3A4 Inducers Prohibited During the Trial	
**Potent CYP3A4 Inducers:**	
Carbamazepine	Enzalutamide
Mitotane	Phenytoin
St. John's wort	
**Moderate CYP3A4 Inducers:**	
Bosentan	Efavirenz
Etravirine	Modafinil

Note: Please communicate with the sponsor's medical monitor if the participant needs to receive any medication (other than IMP or RHEZ) that is not on this list.

6.5.2 Permitted Medications or Therapies

Not applicable.

6.5.3 Rescue Medications

Rescue treatment consisting of the standard of care treatment, ie, RHEZ, will be provided in the following circumstances:

1) If a participant is sputum culture positive for *M tuberculosis* at the end of treatment,
2) If a participant has a microbiologic relapse subsequent to the end of treatment, or
3) If, in the opinion of the investigator, a participant is clinically deteriorating, based on any combination of signs, symptoms, or chest radiograph data.

Note: Standardized rescue treatment consisting of RHEZ is to be used for Arm 1, Arm 2, and Arm 3 not responding to therapy. However, participants in Arm 4 will receive individualized care according to local guidelines. All participants who require rescue treatment will be considered treatment failures for the purposes of the trial.

6.6 Intervention After the End of the Trial

Not applicable.

7 Stopping Rules, Withdrawal Criteria, and Procedures

7.1 Entire Trial or Treatment

If the sponsor terminates or suspends the trial for any reason, prompt notification will be given to investigators, Independent Ethics Committee (IECs)/Institutional Review Boards (IRBs), and regulatory authorities in accordance with regulatory requirements.

7.2 Individual Site

Individual trial site participation may be discontinued by the sponsor, the investigator, or the IEC if judged to be necessary for medical, safety, regulatory, ethical or other reasons consistent with applicable laws, regulations, and Good Clinical Practice (GCP). The investigator will notify the sponsor promptly if the trial is terminated by the investigator or the IEC at the site.

7.3 Individual Participant Discontinuation

After the first dose of IMP or RHEZ on Day 1, a participant may stop treatment permanently for a variety of reasons. Treatment discontinuations may be initiated by a participant or may become medically necessary due to AEs, required treatment with a disallowed medication or therapy, or other reasons, as determined by the investigator. However, each investigator must comprehensively review the circumstance and offer the participant options for continued treatment to the degree possible as described in Section 7.3.5.

A participant who discontinues treatment will have an end of treatment visit, which should be scheduled as soon as possible after the participant's last dose of IMP to conduct the assessments defined in Table 1.3-1.

After the end of treatment visit, off-treatment completers may remain in the trial to complete some or all of the remaining visit assessments as defined in Table 1.3-1.

Participants whose consents are completely withdrawn will be discontinued from the trial as trial non-completers.

If during the trial the sponsor finds any unacceptable safety concerns on the safety of a high dose DLM + BDQ + OPC-167832 group, the enrollment of new participants in the high dose group can be discontinued or reduced, and allocated to the remaining sample size for other dose level(s).

7.3.1 Treatment Interruption

Not applicable.

7.3.2 Treatment Discontinuation

After treatment assignment, a participant may stop treatment permanently for a variety of reasons. Treatment discontinuations may be initiated by a participant or may become medically necessary due to AEs, required treatment with a disallowed medication or therapy, or other issues, as determined by the investigator. However, each investigator

must comprehensively review the circumstances and offer the participant options for continued treatment to the degree possible as described in Section 7.3.5.

7.3.3 Documenting Reasons for Treatment Interruption or Discontinuation

A participant may discontinue IMP for the reasons listed below:

- Adverse event
  - Subject decides to discontinue because of annoyance or discomfort due to a nonserious AE which is not otherwise determined to be an undue hazard
  - Continuing IMP places the participant at undue risk as determined by the investigator (eg, a safety concern that is possibly, probably, or likely related to IMP)
    - SAE
    - Other potentially IMP-related safety concerns or AEs
- Death
- Disease relapse
- Failure to meet randomization criteria
- Lack of efficacy
- Lost to follow-up
- Noncompliance with IMP
- Physician decision
- Pregnancy (see Section 10.3)
- Progressive disease
- Protocol deviation
- Protocol violation
- Protocol-specific withdrawal criterion met
- Randomized by mistake
- Randomized by mistake with trial treatment
- Site terminated by sponsor
- Trial terminated by sponsor
- Withdrawal by participant

If the participant discontinues IMP due to an AE, the investigator, or other trial personnel, will make every effort to follow the event until it has resolved or stabilized. Follow-up procedures in Section 7.3.2 must be followed.

7.3.4 Withdrawal of Consent

Each participant has the right to withdraw their consent from further participation in the trial at any time without prejudice. Participants can withdraw consent for use of data which has not previously been anonymously transferred into trial data sets collected as part of the trial and can only withdraw consent for future participation. The investigator can also discontinue a participant's participation in the trial at any time if medically necessary. Unless the participant provides their written withdrawal of consent or there is other written documentation by the investigator confirming the participant's verbal intent to completely withdraw from the trial, participants should be followed for all protocol-specified evaluations and assessments, if possible.

Complete withdrawal of consent requires a participant's refusal of ALL of the following methods of follow-up:

- Participation in all follow-up procedures specified in the protocol (whether in-clinic, by telephone, or by a home visit).
- Participation in a subset of protocol specified follow-up procedures (by a frequency schedule and method, as agreed by participant and trial site staff).
- Contact of the participant by trial personnel, even if only by telephone, to assess current medical condition, and obtain necessary medical or laboratory reports relevant to the trial's objectives.
- Contact of alternative person(s) who have been designated in source records as being available to discuss the participant's medical condition, even if only by telephone, mail, or email (eg, family, spouse, partner, legal representative, friend, neighbor, or physician).
- Access to medical information from alternative sources (eg, hospital/clinic medical records, referring doctor's notes, public records, dialysis, transplantation or vital registries, social media sources).

Withdrawal of consent is a critical trial event and, therefore, should be approached with the same degree of importance and care as is used in initially obtaining informed consent. The reasons for a participant's intended withdrawal need to be completely understood, documented, and managed to protect the rights of the participant and the integrity of the trial. A participant may initially express their desire to discontinue IMP administration, which is not equivalent to a complete withdrawal of consent for further participation (see Section 7.3.2). A participant may, however, indicate that further trial participation is creating a burden on their work, school, or social schedule. Therefore, the investigator should follow the procedures outlined in Section 7.3.3 to determine if the participant can continue participation in the trial if modifications to his/her treatment and/or schedule of assessments can be accommodated. Only participants who withdraw their permission for

all of the above methods of follow-up are considered to have completely withdrawn their consent to participate in the trial.

CCI [redacted]

7.3.5 Procedures to Encourage Continued Trial Participation

In all cases of impending IMP discontinuation or consent withdrawal, investigators will be instructed to meet and discuss (without undue coercion) with the participant their options of continuing in the trial, preferably on therapy. The investigator should ensure understanding and documentation of the reasons for the participant's desire to withdraw consent. Refer to Section 6.5.3 regarding rescue medications.

7.4 Definition of Participants Lost to Follow-up

Participants who cannot be contacted on or before the trial visits at Weeks 17 (for Arm 1, Arm 2, and Arm 3) and Week 26 (for Arm 4) during the treatment period, who do not have a known reason for discontinuation (eg, withdrew consent or AE), and for whom a survival status at the end of the trial cannot be determined will be classified as "lost to follow-up". Survival status can be determined from a variety of sources, either by obtaining acceptable documentation for death (ie, death certificate, medical records, public records, statement by a family member or primary care physician) or acceptable documentation for life (ie, direct contact with the participant, medical records, successful telephone contact with the participant, statement by a family member or primary care physician, or public records).

The site will make 3 documented attempts to contact the participant by telephone and in the event the site is unable to reach the participant by telephone, the site will attempt to contact the participant via certified mail or an alternative similar method, where appropriate, before assigning a "lost to follow-up" status.

If the participant was classified as "lost to follow-up", "Were you able to contact the participant?", "Date of contact/Date of final contact attempt" and "Contact method" will be recorded in the eCRF.

8 Trial Procedures

The assessments to be conducted during the trial are summarized in Table 1.3-1.

8.1 Efficacy Assessments

8.1.1 Sputum Sampling and Microbiology Laboratory Assessments

Refer to Section 10.5 (Appendix 5) for a detailed collection schedule of sputum samples for each treatment arm.

8.1.1.1 Sputum for Eligibility Assessments

A sputum sample will be collected spontaneously (ie, not induced) by the site staff during the screening period (Day -14 to Day -2) to confirm the presence of *M tuberculosis* that is susceptible to isoniazid and rifampin. The specimen will be tested by a molecular assay that can rapidly detect drug resistance to these 2 drugs.

8.1.1.2 Sputum for Efficacy Assessments

Sputum samples will be collected as per Table 1.3-1 for processing.

Up to 3 sputum samples will be tested at each scheduled visit except for Day −1 and Day 1 (on these days, site staff will collect 1 sputum sample for culture, and 1 sputum for ribosomal ribonucleic acid synthesis ratio [RS ratio] on each day). The Day 1 sputum will be collected prior to the first dose of IMP. For subsequent visits, specimen number 1 will be collected by the participant early in the morning, preferably prior to the morning meal. On Day -1 only, sputum specimen 1 will be tested for acid-fast bacilli smear microscopy to assess baseline bacterial burden, in addition to the assays below.

Specimen number 2 will be collected at the site by the trial site staff. Sputum specimens 1 and 2 from all study visits will be processed using the Mycobacteria Growth Indicator Tube® (MGIT) liquid culture system. Culture growth will be confirmed as *M tuberculosis* complex using a rapid commercial test (immunochromatographic or molecular). An immunoassay to measure sputum LAM concentration will also be performed at each visit. Lowenstein-Jensen cultures may be performed on sputum as necessary.

If a participant (in Arms, 1, 2, and 3) is unable (RHEZ) to spontaneously expectorate sputum number 2 during site collection, an attempt will be made to induce sputum production by aerosol inhalation. Trial sites will use their local procedure for sputum induction. If neither sputum number 1 nor sputum number 2 is collected on the visit date, participants should return to the site to attempt to produce a sample(s) once over the next 72 hours, if necessary. Sputum samples will be defined as unobtainable if no sputum can be obtained during this 72-hour period after an attempt at sputum induction.

If the participant is able to spontaneously produce a subsequent specimen during the visit, specimen number 3 will be collected directly into ribonucleic acid preservation media

and frozen for batch measuring of the RS ratio. If this sputum cannot be collected, this will not be considered a protocol deviation.

Refer to the trial operations manual for further guidance on proper collection of sputum specimens at each scheduled visit.

Susceptibility testing (DST) for anti-TB medications will be performed on positive *M tuberculosis* isolates from Day -1 cultures. If Day -1 culture is not positive for *M tuberculosis*, testing will be performed on Day 1 or Week 1 cultures. Susceptibility testing will also be performed on the end of treatment sputum specimen, if positive for *M tuberculosis* (Week 17 for Arms 1, 2, and 3, or Week 26 for Arm 4), as well as on positive cultures from participants suspected of relapse. Susceptibility testing may also be performed at other timepoints at the request of the principal investigator or the sponsor.

To determine if the participant has relapsed or has a new infection of *M tuberculosis*, genotyping will be performed on the baseline culture (Day −1 and/or Day 1, or Week 1, if necessary) and the first of any subsequent positive culture during the post treatment follow-up period. In addition, the sponsor may request genotyping as needed.

Positive *M tuberculosis* isolates will be stored long-term for confirmatory testing and potential additional characterization of the bacteria, as necessary.

8.1.2 Signs and Symptoms of Tuberculosis

An aggregate of clinical signs and symptoms of pulmonary TB will be assessed at screening and throughout the trial. Signs and symptoms will be assessed by the investigator (cough, hemoptysis, dyspnea, chest pain, loss of appetite, feeling feverish, and night sweats); from vital signs and physical examination (weight, body mass index, body temperature, respiratory rate and heart rate); from clinical laboratory tests (hemoglobin, hematocrit, c-reactive protein, and serum albumin); and from chest radiograph. A chest radiograph must be taken during the screening period, and an official interpretation must be available, and the assessment of its compatibility with pulmonary TB must be made, prior to randomization. An end of treatment chest radiograph will be taken at Week 17 (for participants in Arms 1, 2, and 3) or Week 26 (for participants in Arm 4) and at Month 12.

8.2 Pharmacokinetic Assessments

Sparse sampling will be performed; population PK analysis will be used to analyze PK characteristics of DLM and metabolite DM-6705, BDQ and its metabolite M2, and OPC-167832, as applicable.

For participants randomized to Arm 1, Arm 2, and Arm 3, PK samples will be taken on Day 1 (predose to DLM and postdose), and then Week 2 (predose), Week 4 (predose), Week 8 (predose and postdose), Week 13 (predose), and Week 17/ET (predose and postdose) (9 samples per participant). One or 2 blood samples for OPC-167832, DLM and its metabolite DM-6705, and BDQ and its metabolite M2 will be taken from each participant at each PK sampling timepoint. (The predose blood draw should be drawn 15 minutes prior to the daily dose of OPC-167832/DLM/BDQ. The postdose blood draw should be done at a flexible time approximately 2-8 hours postdose). If a PK blood sample cannot be drawn at the designated time, a window of ± 15 minutes for each blood draw is acceptable, with the exact time recorded.

Participants randomized to treatment Arm 4 will not have PK sampling.

8.2.1 Pharmacokinetic Blood/Serum/Plasma Samples

Blood samples will be collected to determine the concentrations of DLM and its metabolite DM-6705, and for OPC 167832. Blood samples also will be collected to determine the concentrations of BDQ and its metabolite M2.

The concentrations of additional metabolites that are not identified in the protocol may also be determined, if needed. In addition, PK samples may be used for the investigation of a bioanalytical method, if needed.

Blood samples for PK analysis will be collected at the time points as shown in Table 1.3-1 (schedule of assessments). These samples should be collected within 15 minutes (ie, ± 15 minutes) of the nominal sampling window. Predose samples should be collected within 15 minutes before IMP administration.

When vital signs or ECGs are scheduled at the same nominal time as PK sample collections, vital signs should be measured and ECGs should be performed before PK samples are collected.

The actual date and time of the PK sample collection will be recorded in the eCRF.

After processing into plasma, aliquots will be placed into appropriately labeled tubes and will be placed in a freezer set at −70°C.

All plasma samples will be shipped to the central repository. Additional information will be provided in the Operations Manual.

8.3 Pharmacodynamic Assessments

8.3.1 Pharmacodynamic Laboratory Tests

Sputum culture using MGIT: culture conversion and time-to-detection.

8.4 Pharmacogenomic Assessments

8.4.1 Pharmacogenomic Samples

Not applicable.

8.5 Exploratory Biomarker Assessments

Exploratory biomarkers include positron emission tomography/computerized axial tomography (PET/CT) imaging, RS ratio, and transcriptomic signatures from whole blood. The type of PET/CT in this trial is positron emission tomography with 2-[^{18}F]-fluoro-2-deoxy-D-glucose.

Whole blood samples to longitudinally assess transcriptomic signatures previously associated with TB cure will be collected using Paxgene ribonucleic acid tubes at the time points described in the schedule of assessments (Table 1.3-1). Genomic ribonucleic acid will be extracted from a whole blood sample and stored until shipped to the designated laboratory for analysis. Additional information will be provided in the Laboratory Manual.

Results from exploratory biomarker assessments will be reported separately from the main clinical study report.

CCI [redacted]

CCI [redacted]

8.7 Safety Assessments

Details pertaining to the definitions, collection, reporting, and follow-up of AEs are described in Section 8.8.

8.7.1 Clinical Laboratory Assessments

Clinical laboratory samples will be collected at the time points described in the schedule of assessments (Table 1.3-1) to perform the clinical laboratory assessments described in Section 10.2. Refer to Section 8.8.5 for potential serious hepatotoxicity. The Division of AIDS (DAIDS) table criteria will be used for identifying laboratory values of potential clinical relevance.[8] The total volume of blood to be collected during the trial will be documented in the ICF form.

8.7.2 Physical Examination

Either a complete or targeted physical examination will be performed at the time points described in the schedule of assessments (Table 1.3-1). Visual assessments to be completed during screening will consist of Snellen and Ishihara testing and will be repeated during the trial if the investigator suspects abnormalities.

8.7.3 Vital Signs

Vital signs include systolic and diastolic blood pressure (mmHg), heart rate (bpm), respiratory rate (rpm), body temperature (C), height (cm) (only at screening), weight, and body mass index (BMI). Vital signs will be obtained prior to PK blood draws and ECGs at the nominal time points, where applicable. At screening, blood pressure and heart rate will be taken with the participant in the supine (performed first) and sitting position after remaining for ≥ 3 minutes in each position and temperature and respiratory rate will be taken with the participant in the supine position. At all other time points, vital signs will be obtained after the participant has been at rest in the supine position for at least 1 minute.

Vital signs will be collected at the trial visits described in the schedule of assessments (Table 1.3-1). Participants should be monitored for potentially clinically significant vital signs values. The DAIDS table criteria will be used for identifying vital signs of potential clinical relevance.[8]

8.7.4 Electrocardiogram

One standard 12-lead ECG will be performed during screening, plus 3 ECGs at baseline (Day −1) spaced 5 to 10 minutes apart, and 3 ECGs at all subsequent visits during the treatment and follow up periods. Where multiple ECGs are required in a visit, the participant should be recumbent for 10 minutes prior, if possible, and ECGs should be collected 5 to 10 minutes apart after the dose of IMP and prior to PK blood draws.

Electrocardiograms will be performed at the trial visits described in the schedule of assessments (Table 1.3-1). Participants should be monitored for potentially clinically significant ECG results. The DAIDS table criteria will be used for identifying ECGs of potential clinical relevance.[8]

8.7.5 Suicidality Monitoring

Not applicable.

8.7.6 Other Safety Variables

A PET/CT scan will be performed on participants that consent to this assessment at the trial visits described in the schedule of assessments (Table 1.3-1). If the PET/CT scan is unavailable, the participant is permitted to continue in the trial.

8.8 Adverse Events

8.8.1 Definitions

An AE is defined as any untoward medical occurrence in a clinical trial participant administered an IMP or trial treatment and which does not necessarily have a causal relationship with this treatment. Adverse events would not include information recorded as medical history at screening for preplanned procedures for which the underlying condition was known and no worsening occurred. An adverse reaction is any untoward and unintended response to an IMP related to any dose administered.

A suspected adverse reaction is any AE for which there is a reasonable possibility that the IMP caused the AE. For the purpose of Investigational New Drug (IND) safety reporting, “reasonable possibility” means there is evidence to suggest a causal relationship between the IMP or trial treatment and the AE. Suspected adverse reaction implies a lesser degree of certainty about causality.

TEAEs are defined as AEs with an onset date on or after the start of treatment. In more detail, TEAEs are all AEs which started after the start of IMP treatment; or if the event was continuous from baseline and was worsening, serious, IMP related, or resulted in death, discontinuation, interruption, or reduction of IMP.

An SAE includes any event that results in any of the following outcomes:

- Death
- Life-threatening; ie, the participant was, in the opinion of the investigator, at immediate risk of death from the event as it occurred. It does not include an event that, had it occurred in a more severe form, might have caused death.
- Persistent or significant incapacity/disability or substantial disruption of the ability to conduct normal life functions.
- Requires inpatient hospitalization or prolongs hospitalization.
  - Hospitalization itself should not be reported as an SAE; whenever possible the reason for the hospitalization should be reported.
  - Hospitalizations or prolonged hospitalizations for social admissions (ie, those required for reasons of convenience or other nonmedical need) are not considered SAEs.
  - Prescheduled hospitalization to address a condition that has existed prior to the signing of the ICF should not be considered an SAE.
- Congenital anomaly/birth defect.
- Other medically significant events that, based upon appropriate medical judgment, may jeopardize the participant and may require medical or surgical intervention to prevent one of the outcomes listed above; eg, allergic bronchospasm requiring intensive treatment in an emergency room or home, blood dyscrasias or convulsions that do not result in hospitalization, or the development of drug dependency or drug abuse.

Nonserious AEs are all AEs that do not meet the criteria for a "serious" AE.

Adverse Events of Special Interest (AESIs): A noteworthy event for the particular product/IMP or class of products that a sponsor may wish to monitor carefully. All AESIs are to be reported as immediately reportable event (IREs). No AESIs have been identified for the IMP to be administered during this trial. There are no AESIs in this trial.

Immediately Reportable Event (IRE):

- Any SAE.
- Any AE related to occupational exposure.
- Potential serious hepatotoxicity (see Section 8.8.5).
- Pregnancies are also defined as IREs. Although normal pregnancy is not an AE, it will mandate IMP discontinuation and must be reported on an IRE form and the Clinical Trial Pregnancy and Breastfeeding Form(s) to the sponsor. This includes pregnancy of the participant or the partner of the participant. Pregnancy will only be documented on the AE eCRF if the pregnancy occurs in a female participant and there is an abnormality or complication.

Clinical Laboratory Test Value Changes: It is the investigator's responsibility to review the results of laboratory tests for each individual participant as they become available. This review will be documented by the investigator's dated signature on the laboratory report. The investigator may repeat the laboratory test or request additional tests to verify the results of the original laboratory tests. If this laboratory value is considered medically relevant (ie, clinically significant) by the investigator (participant is symptomatic, requiring corrective treatment or further evaluation), or if the laboratory value leads to discontinuation, and/or fulfills a seriousness criterion, this is considered an AE.

Severity: Adverse events will be graded on a 3-point scale and reported as indicated in the eCRF. The severity of an adverse experience is defined as follows:

**1 = Mild:**	Discomfort noticed, but no disruption to daily activity.
**2 = Moderate:**	Discomfort sufficient to reduce or affect normal daily activity.
**3 = Severe:**	Inability to work or perform normal daily activity.

Note: In addition, all AEs will be graded on a 5-point scale according to the "DAIDS Table for Grading the Severity of Adult and Pediatric AEs".[8]

IMP Causality: Assessment of causal relationship of an AE to the use of the IMP is defined as follows:

**Related**:	There is a reasonable possibility of a temporal and causal relationship between the IMP and the AE.
**Not Related**:	There is no temporal or causal relationship between the IMP and the AE.

8.8.2 Eliciting and Reporting Adverse Events

The investigator will regularly assess participants for the occurrence of AEs. To avoid bias in eliciting AEs, participants should be asked the nonleading question: "How have you felt since your last visit?" All AEs (serious and nonserious) reported by the participant must be recorded on the source documents and eCRF provided by the sponsor. Adverse event collection will begin after a participant signs the ICF, and will continue until the follow up visit 12 months postrandomization. All AEs must be reported after participant informed consent has been obtained, including screening failures due to AEs, irrespective of IMP administration.

Medical terminology should be used for AE reporting. Adverse events should be reported as a single unifying diagnosis whenever possible or, in the absence of a unifying diagnosis, as individual signs or symptoms.

Exacerbation or disease progression should be reported as an AE only if there are unusual or severe clinical features that were not present, or experienced earlier, or not expected based on the course of the condition.

In addition, the sponsor must be notified immediately by telephone, fax, or email of any IREs according to the procedure outlined below, in Section 8.8.3. Special attention should be paid to recording hospitalization and concomitant medications.

The adverse event, start date, end date, seriousness, severity, relationship to trial treatment (IMP causality), action taken with trial treatment, and outcome will be recorded on the source documents and in the eCRF.

8.8.3 Immediately Reportable Events

The investigator must immediately report (within 24 hours), using an IRE form, after he/she or site personnel become aware of any IRE (SAE, AE related to occupational exposure, potential serious hepatotoxicity, or confirmed pregnancy), by telephone, fax, or email to the sponsor or designee using the contact information on the cover page of this protocol (please note that the IRE form is NOT the AE eCRF). Subject confidentiality must be protected and contact information such as name, address, phone number or any other protected health information as determined by applicable local regulation must be redacted when forwarding Safety Information and supporting documentation. Details regarding the follow-up of IREs are included in Section 8.8.7.2.

8.8.4 Adverse Events of Special Interest

Not applicable.

8.8.5 Potential Serious Hepatotoxicity

For a participant who experiences an elevation in AST or ALT that is ≥ 3 times the upper limit of normal (ULN), a total bilirubin level should also be evaluated. If the total bilirubin is ≥ 2 times the ULN, complete an IRE form with all values listed and also report as an AE in the eCRF.

8.8.6 Procedure for Breaking the Blind

Except for the microbiology laboratory being blinded to the treatment assignment of each participant, this trial does not use blinding procedures.

8.8.7 Follow-up of Adverse Events

8.8.7.1 Follow-up of Nonserious Adverse Events

Nonserious AEs that are identified at any time during the trial must be recorded on the AE eCRF with the current status (ongoing or resolved/recovered) noted. All nonserious events (that are not IREs) that are ongoing at the last scheduled contact will be recorded as ongoing in the eCRF. For any AE having been identified throughout the trial, during analysis, additional relevant medical history information may be requested by the sponsor to further ascertain causality (including, but not limited to, information such as risk-related behavior, family history, and occupation).

8.8.7.2 Follow-up of Immediately Reportable Events

This trial requires that participants be actively monitored for IREs up to 12 months after randomization, or the last scheduled visit.

Immediately reportable events that are **identified or ongoing at the last scheduled contact** must be recorded as such on the AE eCRF page and the IRE form. If updated information (eg, resolved status) on IRE status becomes available after a participant's last scheduled contact (up to last in-clinic visit for the entire trial), this must be reported to the sponsor and recorded on the AE eCRF page and the IRE form, according to the appropriate reporting procedures described in Section 8.8.3.

It is expected that the investigator will provide or arrange appropriate supportive care for the participant and will provide prompt updates on the participant's status to the sponsor. The investigator will follow IREs until the events are:

- Resolved,
- Recovered/Resolved with sequelae
- The participant is lost to follow-up, or
- Has died.

Resolution means that the participant has returned to the baseline state of health and stabilized means that the investigator does not expect any further improvement or worsening of the participant's condition. The investigator will continue to report any significant follow-up information to the sponsor up to the point the event has resolved or stabilized, or the participant is lost to follow-up, or has died.

Refer to Section 10.3 for additional information regarding the follow-up period for participants that become pregnant or for pregnant partners of male participants.

8.8.7.3 Follow-up and Reporting of Immediately Reportable Events Occurring After Last Scheduled Contact

Any new IREs reported to the investigator which occur after the last scheduled contact and are determined by the investigator to be reasonably associated with the use of the IMP, should be reported to the sponsor according to the procedures outlined in Section 8.8.3.This may include IREs that are captured on follow-up telephone contact or at any other time point after the defined trial period and continue to report any significant follow-up information to the sponsor until the events are resolved or stabilized, or the participant is lost to follow-up or has died.

8.9 Treatment of Overdose

For treatment of overdose, please refer to the IB for DLM or OPC-167832, or the package insert for BDQ and RHEZ.

8.10 Participant Assessment Recording

Not applicable.

8.11 Other Assessments

Not applicable.

9 Statistical Considerations

9.1 Sample Size

It is planned to enroll enough participants so there will be 20 participants in DLM + BDQ + 10 mg OPC-167832, 40 participants each in DLM + BDQ + 30 mg OPC-167832, and DLM + BDQ + 90 mg OPC-167832, and 20 participants in RHEZ group. This is a pilot trial and exploratory in nature. Thus 80% CIs between proportion differences will be calculated to compare with noninferiority margin. Assuming that the sputum culture conversion (SCC) rate for the primary endpoint is 93% for the RHEZ group, and assuming the SCC rate of the investigational arms is identical to the RHEZ group, with a noninferiority margin of 12%, sample size of 120 participants (100 for pooled DLM + BDQ + OPC-167832 and 20 for RHEZ) will have about 84% power to reject the null hypothesis with a one-sided alpha level of 0.1, ie, the lower limit of the two-sided 80% CI of the difference in proportions of sputum conversion between the pooled investigational arms and RHEZ is larger than -0.12. The Miettinen and Nurminen method to construct 80% CI was used in the sample size calculation.[7] Assuming 11% randomized participants will not demonstrate at least 1 positive culture result by Week 1 and susceptibility to rifampin and isoniazid, approximately 135 participants will be

randomized in 1:2:2:1 ratio to obtain 120 participants in the modified intent-to-treat (mITT) analysis set.

9.2 Datasets for Analysis

The PK analysis set will include all participants with adequate data for the population PK analysis.

The safety analysis set includes all participants who receive any dose of IMP or RHEZ. Safety analysis will be conducted based on the safety analysis set.

The mITT analysis set includes all randomized participants who take at least 1 dose of IMP or RHEZ, have at least 1 postbaseline culture result, and demonstrate positive culture results by Week 1 and susceptibility to rifampin and isoniazid (from the screening specimen).

9.3 Handling of Missing Data for Primary and Secondary Endpoint Analysis

Missing data caused by participant discontinuation will be handled by a composite variable strategy according to International Council for Harmonisation (ICH) E9 (R1) Addendum.[9] Participants who discontinue early from the trial during the treatment period without an SCC outcome will be considered as no SCC.

9.4 Statistical Analyses

9.4.1 Efficacy Analyses

9.4.1.1 Primary Efficacy Endpoint Analysis

The primary efficacy endpoint of the trial is the proportion of participants who have SCC and no subsequent positive culture by the end of treatment. Sputum culture conversion occurs when a participant has the first of 2 visits of at least 1 week apart with sputum cultures negative and without a positive sputum culture result in between.

The 3 DLM + BDQ + OPC-167832 arms will be pooled, and noninferiority with RHEZ will be established if the lower limit of 80% CI for the difference of proportion of participants with SCC between the pooled DLM + BDQ + OPC-167832 group is larger than -0.12. The Miettinen and Nurminen CI of common risk difference stratified by HIV status and cavitation status prior to randomization will be used.[7] If there is a stratum of very small size, strata may be collapsed. The SAP will describe the details. The 80% CI of the proportion difference between each dose level of DLM + BDQ + OPC-167832 arm and RHEZ will be provided. In addition, Cochran-Armitage test will be used to detect trend in proportion of SCC with increasing OPC-167832 dose.

9.4.1.2 Secondary Efficacy Endpoint Analysis

The secondary efficacy endpoints of this protocol are:

- The proportion of participants in each arm who achieve SCC in MGIT by 8 weeks of treatment.
- Time to detection of MGIT cultures.
- The proportion of participants in each arm who convert sputum LAM to negative by 8 weeks of treatment and by end of treatment.
- Proportion of participants who develop drug resistance.
- Time to SCC.

For a proportion endpoint, the same analysis methodology specified for the primary endpoint will be used. A 12% noninferiority margin will be used if the goal is to establish noninferiority.

The Kaplan-Meier curve of time to SCC will be provided for each group and relationship between time to SCC and PK parameters will be studied.

To identify the optimal dose of OPC-167832, a PK/PD analysis will be performed (see Section 9.4.3.4).

Control of Experiment-wise Type 1 Error is not applicable to this trial.

9.4.1.3 Exploratory Biomarker Endpoint Analysis

Exploratory biomarker endpoints are:

- Longitudinally assess PET/CT imaging response over the course of treatment.
- Longitudinally evaluate the RS ratio decline in sputum.
- Longitudinally assess whole blood transcriptomic signatures previously associated with TB cure from serum.

Results from exploratory biomarker assessments will be reported separately from the main clinical study report.

9.4.2 Safety Analysis

Safety endpoints will be summarized using descriptive statistics (number, median, mean, standard deviation, minimum, maximum) for continuous values, and by frequency and percentage for discrete variables. Percentage and frequency of AEs and clinical laboratory test results by grades will be analyzed.

9.4.2.1 Adverse Events

All AEs will be coded by system organ class and Medical Dictionary for Regulatory Activities preferred term. The incidence of the following events will be summarized by treatment group:

- TEAEs
- TEAEs by severity
- TEAEs potentially causally related to the IMP or trial medication
- TEAEs with an outcome of death or trial medication
- Serious TEAEs
- TEAEs leading to discontinuation of the IMP or trial medication

In addition, TEAEs by DAIDS grades will be summarized.

9.4.2.2 Clinical Laboratory Data

For clinical laboratory tests data, baseline is defined as the last nonmissing value obtained at a scheduled visit on or before Day −1. If the Day −1 measurement is missing, the baseline value will be the last nonmissing measurement from the screening visit (the previous scheduled visit). Clinical laboratory test parameters and mean changes from baseline will be summarized by treatment. The incidence of potentially clinically significant changes and abnormalities in clinical laboratory test parameters will be summarized. Clinical laboratory test data will be presented in listings.

9.4.2.3 Physical Examination and Vital Signs Data

Physical examination data will be presented in listings.

Vital sign parameters and mean changes from baseline will be summarized by treatment arm. The incidence of potentially clinically significant changes and abnormalities in vital sign parameters will be summarized. Vital sign data will be presented in listings.

9.4.2.4 Electrocardiogram Data

Electrocardiogram measurements and mean changes from baseline will be summarized by treatment. The baseline ECG is defined as Day −1. If the measurement from Day −1 is missing, the screening ECG is taken as baseline. The incidence of potentially clinically significant changes and abnormalities in ECG evaluations will be summarized. Electrocardiogram data will be presented in listings.

9.4.3 Other Analyses

9.4.3.1 Analysis of Demographic and Baseline Characteristics

Summaries of demographics, TB medical history, TB disease severity, and sputum specimen results at baseline will be provided. Continuous variables will be summarized with mean, standard deviation, minimum, median, and maximum.

9.4.3.2 Pharmacokinetic Analysis

Plasma concentration of OPC-167832, DLM and its metabolite DM-6705, and BDQ and its metabolite M2 are planned to be utilized to perform population PK analyses, as applicable. A population PK analysis will be provided in a separate report.

9.4.3.3 Pharmacodynamic Analysis

Not applicable.

9.4.3.4 Exposure-Response Analysis

Pharmacokinetics/PD and/or exposure-response analysis will be used to analyze the relationship of OPC-167832 plasma exposure alone or combined with DLM and BDQ with the efficacy and safety endpoints, as applicable. The efficacy endpoints including but not limited to LAM, proportion of participants without SCC, time to detection and time to SCC may be explored. A PK/PD and/or exposure-response analysis will be provided in a separate report.

9.4.3.5 Pharmacogenomic Analysis

Transcriptomic signatures previously associated with TB cure from serum will be longitudinally assessed. Details will be included in the approved SAP.

CCI

[redacted]

CCI [redacted]

9.5 Interim Analysis and Adaptive Design

Interim PK/PD analyses will be performed after participants have completed the end of treatment response time point (4 months of treatment in the OPC-167832 arm).

In addition, an early assessment of dose-response and toxicity may be conducted after the first 24 randomized participants (ie, approximately 4:8:8:4 in each arm) complete the end of treatment response time point. If an OPC-167832 arm is dropped, subsequently enrolled participants would then be allocated to the remaining OPC-167832 arms. Further details will be provided in the SAP.

9.5.1 Data Monitoring Committee

An independent Data Monitoring Committee (DMC) will be contracted by Otsuka to review safety and efficacy data during the conduct of the trial. The DMC will convene and operate based on an established charter. The DMC will be permitted to seek additional independent expertise, as needed. The primary responsibility of the DMC will be to monitor safety and to advise the sponsor of the clinical project regarding whether or not the safety concerns merit stopping the trial. Towards this objective the DMC will meet on a predetermined schedule to review safety and available efficacy data.

10 Supporting Documentation and Operational Considerations

10.1 Appendix 1: Regulatory, Ethical, and Trial Oversight Considerations

10.1.1 Ethics and Responsibility

This trial must be conducted in compliance with the protocol, applicable ICH GCP guidance, international ethical principles derived from the Declaration of Helsinki and Council for International Organizations of Medical Science guidelines, and applicable local laws and regulations. Each trial site will seek approval/favorable opinion by an IRB or IEC according to regional requirements, and the investigator will provide that documentation to the sponsor. The IRB/IEC will evaluate the ethical, scientific, and medical appropriateness of the trial. Further, in preparing and handling the eCRF, IRE and any safety information, the investigator, subinvestigator, and their staff will take measures to ensure adequate care in protecting participant privacy. To this end, a participant ID will be used to identify each participant.

Financial aspects, participant insurance, and the publication policy for the trial will be documented in the agreement between the sponsor and the investigator.

10.1.2 Informed Consent

Informed consent will be freely obtained from all participants (or their guardian or legally acceptable representative, as applicable for local laws). The ICF will be approved by the same IRB/IEC that approves this protocol.

Each ICF will comply with the ICH (International Council for Harmonisation) GCP Guidelines, and local regulatory requirements. The investigator will ensure that the sponsor reviews and authorizes any written site-specific ICF used in the trial before submission to the IRB/IEC.

Investigators may discuss trial availability and the possibility for entry with a potential participant without first obtaining consent. However, informed consent must be obtained and documented before initiation of any procedures that are performed solely for the purpose of determining eligibility for this trial, including withdrawal from current medication(s).

Potential participants are free to refuse entry into the trial, or withdraw from the trial at any time, without justification, and there will be no consequences to their further care.

Once appropriate essential information has been provided and fully explained in layman's language to the participant by the investigator (or a qualified designee), and it

has been documented that the participant has had the opportunity to ask questions and have those questions answered, the IRB/IEC-approved paper ICF will be signed and dated by both the participant and the person obtaining consent (investigator or designee), as well as by any other parties required by the IRB/IEC. The participant will receive a copy of the signed ICF; the original shall be kept on file by the investigator. Participants may be asked to sign additional ICFs if the protocol is amended and the changes to the protocol result in additional information that needs to be provided to the participants, so that they can make a knowledgeable and voluntary decision on continued trial participation. Female partners of male participants who become pregnant during the course of the trial may be asked to sign additional ICFs in order to collect additional information regarding the non-participant partner and fetus.

CCI [redacted]

10.1.3 Confidentiality

All information generated in this trial will be considered confidential and will not be disclosed to anyone not directly concerned with the trial without the sponsor's prior written permission. Subject confidentiality requirements of the region(s) where the trial is conducted will be met. However, authorized regulatory officials and sponsor personnel (or their representatives) may be allowed full access to inspect and copy the records, consistent with local requirements. All IMPs, participant bodily fluids, and/or other materials collected shall be used solely in accordance with this protocol, unless otherwise agreed to in writing by the sponsor.

Participants will be identified only by unique participant ID in the eCRF. If further participant ID is required, participants' full names may be made known to a regulatory agency or other authorized officials if necessary, sub participant ject to local regulations.

10.1.4 Quality Control and Quality Assurance

10.1.4.1 Monitoring

The sponsor has ethical, legal, and scientific obligations to follow this trial in accordance with established research principles, the applicable ICH GCP guidance, and applicable regulatory requirements and local laws. As part of a concerted effort to fulfill these obligations (maintain current personal knowledge of the progress of the trial), the sponsor's monitors will visit the site during the trial, as well as communicate frequently via telephone, email, and written communications. In addition, all investigators and trial

site personnel will undergo initial and ongoing training for this particular trial, and this training will be clearly documented.

10.1.4.2 Auditing

The sponsor's Quality Assurance Unit (or representative) may conduct trial site audits. Audits will include, but are not limited to, IMP supply, presence of required documents, the informed consent process, site operations, delegation of authority and training, and a review of the eCRF with source documents, as applicable. The investigator will agree to cooperate and participate with audits.

Regulatory authorities may inspect the investigator site during or after the trial. The investigator will cooperate with such inspections and will contact the sponsor immediately if such an inspection occurs.

10.1.5 Protocol Deviations

In the event of a significant deviation from the protocol due to an emergency, accident, or mistake (eg, violation of informed consent process, IMP dispensing or participant dosing error, treatment assignment error, participant enrolled in violation of eligibility criteria or concomitant medication criteria), the investigator or designee will contact the sponsor or designee at the earliest possible time by telephone or via email. The investigator and sponsor (or designee) will come as quickly as possible to a joint decision regarding the participant's continuation in the trial. This decision will be documented by the investigator and the sponsor (or designee) and reviewed by the site monitor.

Any major protocol deviation will be recorded in the eCRF along with the start date and details of the deviation.

10.1.6 Records Management

10.1.6.1 Source Documents

Source documents are defined as the results of original observations and activities of a clinical investigation. Source documents will include but are not limited to medical records, electronic data, logs, and recorded data from automated instruments or applications. All source documents pertaining to this trial will be maintained by the investigators and made available for direct inspection by authorized persons.

Investigator(s)/institution(s) will permit trial-related monitoring, audits, IRB/IEC review, and regulatory inspection(s) by providing direct access to source data/documents by authorized persons as defined in the ICF. In all cases, participant confidentiality must be maintained in accordance with local regulatory requirements.

10.1.6.2 Data Collection

During each participant's visit to the site, an investigator or their designee participating in the trial will record information to document all significant observations. At a minimum, these notes will contain:

- Documentation of the informed consent process, including any revised consents;
- Documentation of the investigator's decision to enroll the participant into the trial, the review of all inclusion/exclusion criteria prior to IMP administration, and confirmation of the participant's actual participation in the trial;
- The date of the visit and the corresponding Visit or Day in the trial schedule;
- General participant status remarks, including any *significant* medical findings. The severity, frequency, duration, action taken, and outcome of any AEs and the investigator's assessment of relationship to IMP must also be recorded;
- Any changes in concomitant medications or dosages;
- A general reference to the procedures completed, including dosing and IMP compliance;
- The signature (or initials) and date of the investigator (or designee) who made an entry in the medical record.

In addition, any contact with the participant via telephone or other means that provides significant clinical information will also be documented in the progress notes as described above.

Information from medical records and other source documents will be entered by investigative site personnel onto eCRFs in the sponsor's electronic data capture (EDC) system that is 21 Code of Federal Regulations Part 11 compliant. Changes to the data will be captured by an automatic audit trail in the EDC system.

Electronic data not entered on eCRF, such as data received from central laboratories and central ECG readers, will be reconciled using key data fields by the sponsor or the clinical research organization with the eCRF data to ensure consistency.

10.1.6.3 File Management at the Trial Site

The investigator will ensure that the trial site file is maintained in accordance with applicable ICH GCP guidance and as required by applicable local regulations. The investigator/institution will take measures to prevent accidental or premature destruction of these documents.

10.1.6.4 Records Retention at the Trial Site

Regulatory requirements for the archival of records for this trial necessitate that participating investigators maintain detailed clinical data for the longest of the following 3 periods:

- A period of at least 2 years after the date on which approval to market the drug is obtained (or if IMP development is discontinued, the date regulatory authorities were notified of discontinuation); OR
- A period of at least 3 years after the sponsor notifies the investigator that the final report has been filed with regulatory authorities.
- Longer, region-specific storage requirements, if applicable.

The investigator must not dispose of any records relevant to this trial without either (1) written permission from the sponsor or (2) provision of an opportunity for the sponsor to collect such records. The investigator will be responsible to maintain adequate and accurate electronic or hard copy source documents of all observations and data generated during this trial including any data clarification forms received from the sponsor. Such documentation is participant to inspection by the sponsor and relevant regulatory authorities. If the investigator withdraws from the trial (eg, due to relocation or retirement), all trial-related records should be transferred to a mutually agreed-upon designee within a sponsor-specified timeframe. Notice of such transfer will be given to the sponsor in writing.

10.1.6.5 Publication Authorship Requirements

Authorship for any Otsuka-sponsored publications resulting from the conduct of this trial will be based on International Committee of Medical Journal Editors (ICMJE) authorship criteria (http://www.icmje.org/recommendations). According to ICMJE guidelines, one may be considered an author only if the following criteria are met:

1) Substantial contributions to the conception or design of the work; or the acquisition, analysis, or interpretation of data for the work; AND
2) Drafting the work or revising it critically for important intellectual content; AND
3) Final approval of the version to be published; AND
4) Agreement to be accountable for all aspects of the work in ensuring that questions related to the accuracy or integrity of any part of the work are appropriately investigated and resolved.

All authors must meet the above criteria, and all who qualify for authorship based on the above criteria should be listed as authors.

Investigators or other trial participants who do not qualify for authorship may be acknowledged in publications resulting from the trial. By agreeing to participate in the trial, investigators or other trial participants consent to such acknowledgement in any publications resulting from its conduct.

10.2 Appendix 2: Clinical Laboratory Tests

The tests detailed in Table 10.2-1 will be performed.

Table 10.2-1 Clinical Laboratory Assessments

Hematology:<br>Hemoglobin<br>Hematocrit<br>Mean corpuscular hemoglobin concentration<br>Mean corpuscular volume<br>Red blood cell (RBC) count<br>White blood cell (WBC) count (absolute and differential)<br>Platelets<br>Coagulation (prothrombin time [PT], partial thromboplastin time [PTT], international normalized ratio [INR])<br>CD4 count<br><br>Urinalysis:<br>Specimen appearance<br>Color<br>Occult blood<br>Glucose<br>Microscopic analysis, if indicated, WBC/RBC counts per high powered field<br>pH<br>Protein<br>Specific gravity<br><br>Additional Tests:<br>Urine pregnancy for FOCBP<br>Serology (hepatitis B surface antigen [HbsAg], hepatitis C antibodies [anti-HCV], HIV), SARS-CoV-2 polymerase chain reaction test or antigen test (where appropriate)<br>HIV-viral load<br>HbA1c	Serum Chemistry:<br>Alkaline phosphatase<br>Alanine aminotransferase<br>Aspartate aminotransferase<br>Bilirubin<br>Blood urea nitrogen<br>Calcium<br>Cholesterol<br>Creatinine/eGFR<br>Gamma glutamyl transferase<br>Glucose<br>Lactate dehydrogenase<br>Magnesium<br>Potassium<br>Protein<br>Sodium<br>Triglycerides<br>Serum albumin<br>C-reactive protein<br><br>Drug Screen (all items in urine except where noted)<br>Alcohol (urine or blood test)<br>Amphetamines<br>Barbiturates<br>Benzodiazepines<br>Cannabinoids<br>Cocaine<br>Methadone<br>Opiates<br>Phencyclidine

10.3 Appendix 3: Contraceptive Guidance and Collection of Pregnancy Information

Females of childbearing potential (FOCBP) are females whose menstruation has started and who are not documented as sterile (eg, have had a bilateral oophorectomy, or hysterectomy, or who have been postmenopausal for at least 12 months). Females of nonchildbearing potential do not meet definition of FOCBP.

For males and FOCBP, or their partners, who are sexually active, there must be a documented agreement that the participant and their partner will take effective measures (ie, 2 different approved methods of birth control or abstinence) to prevent pregnancy during the course of the trial and for 12 weeks after the last dose of IMP or RHEZ. Unless the participant is sterile (ie, females who have had a bilateral oophorectomy, have had a hysterectomy, or have been postmenopausal for at least 12 consecutive months; or males who have had a bilateral orchiectomy) and for 12 weeks after the last dose of IMP of RHEZ, 2 of the following approved methods of birth control must be used: vasectomy, tubal ligation, intrauterine device, birth control pill, birth control implant, birth control depot injection, birth control patch, condom, sponge, or occlusive cap (vaginal diaphragm or cervical/vault cap). Any single method of birth control, including vasectomy and tubal ligation, may fail, leading to pregnancy. The contraceptive method will be documented in the eCRF or source document. Male participants must also agree not to donate sperm from trial screening through 12 weeks after the last dose of IMP or RHEZ.

Before enrolling males and females in this clinical trial, investigators must review the below information about trial participation as part of the ICF process. The topics should generally include:

- General information
- Informed consent form
- Pregnancy prevention information
- Drug interactions with hormonal contraceptives
- Contraceptives in current use
- Follow-up of a reported pregnancy

Before trial enrollment, males and FOCBP must be advised of the importance of avoiding pregnancy during trial participation and the potential risk factors for an unintentional pregnancy. Participants must sign the ICF confirming that the above-mentioned risk factors and the consequences were discussed.

A urine or serum pregnancy test for human chorionic gonadotropin will be performed at screening on all FOCBP and female participants ≥ 12 years of age, if menstruation has

started. If a urine test is performed and is positive, the investigator will follow-up with a confirmatory serum test.

During the trial, all FOCBP should be instructed to contact the investigator immediately if they suspect they might be pregnant (eg, missed or late menstrual cycle). Male participants must be instructed to contact the investigator immediately, during the trial, if their partner suspects that they might be pregnant (eg, missed or late menstrual cycle).

If a participant is suspected to be pregnant before she receives IMP or RHEZ, the IMP or RHEZ administration must be withheld until the results of serum pregnancy tests are available. If the pregnancy is confirmed, the participant must not receive the IMP or RHEZ and must not be enrolled in the trial. If pregnancy is suspected while the participant is taking IMP or RHEZ, the IMP or RHEZ must be withheld immediately (if reasonable, taking into consideration any potential withdrawal risks) until the result of the pregnancy test is known. If pregnancy is confirmed, the IMP will be permanently discontinued in an appropriate manner (eg, dose tapering if necessary for participant safety) but the participant will not be automatically withdrawn from the trial. Pregnant participants will be encouraged to remain in the trial and continue with all visit procedures as scheduled other than dispensation of IMP unless contraindicated by pregnancy.

The investigator must immediately notify the sponsor (within 24 hours) of any pregnancy associated with IMP or RHEZ exposure during the trial and for at least 12 weeks after the last dose of IMP or RHEZ, and record the event on the IRE form and forward it to the sponsor. The sponsor will forward the Clinical Trial Breastfeeding and Pregnancy form in addition to the IRE form to the investigator for monitoring the outcome of the pregnancy.

Protocol required procedures for trial discontinuation and follow-up must be performed on the participant unless contraindicated by pregnancy (eg, x-ray studies). Other appropriate pregnancy follow-up procedures should be considered if indicated. In addition, the investigator must report to the sponsor, on the Clinical Trial Breastfeeding and Pregnancy Form(s), follow-up information regarding the course of the pregnancy, including perinatal and neonatal outcome. Infants will be followed for a minimum of 6 months from the date of birth. Local regulatory requirements must be followed for follow-up and reporting on pregnancy cases and/or infants.

10.4 Appendix 4: List of Abbreviations

Abbreviation	Definition
AE	Adverse event
AESI	Adverse event of special interest
ALT	Alanine aminotransferase
Anti-HCV	Hepatitis C antibodies
AST	Aspartate aminotransferase
AUC	Area under the concentration-time curve
AUC_{24h}	Area under the concentration-time curve from zero to 24 hours
BDQ	Bedaquiline
BID	Twice daily
BMI	Body mass index
CFR	Cumulative fraction of response
CFU	Colony-forming unit
CI	Confidence interval
C_{max}	Maximum (peak) plasma concentration
CYP	Cytochrome P450
DAIDS	Division of AIDS
DprE1	Decaprenylphosphoryl-β-D-ribose 2'-oxidase
DLM	Delamanid
DMC	Data Monitoring Committee
DOT	Directly observed therapy
DS-TB	Drug-susceptible tuberculosis
EBA	Early bactericidal activity
ECG	Electrocardiogram
eCRF	Electronic case report form
EDC	Electronic data capture
eGFR	Estimated glomerular filtration rate
ET	Early termination
CCI [redacted]	
FOCBP	Female of childbearing potential
GCP	Good Clinical Practice
HbA1c	Glycosylated hemoglobin
HBsAg	Hepatitis B surface antigen
HCV	Hepatitis C virus
HFS-TB	Hollow fiber system of tuberculosis
HIV	Human immunodeficiency virus
IB	Investigator's Brochure
ICF	Informed consent form
ICH	International Council for Harmonisation
ICMJE	International Committee of Medical Journal Editors
ID	Identification
IEC	Independent Ethics Committee
IMP	Investigational medicinal product
IND	Investigational New Drug

Abbreviation	Definition
INR	International normalized ratio
IRB	Institutional Review Board
IRE	Immediately reportable event
LAM	Lipoarabinomannan
MAD	Multiple ascending dose
MDR-TB	Multidrug-resistant tuberculosis
MGIT	Mycobacteria Growth Indicator Tube®
MIC	Minimum inhibitory concentration
mITT	Modified intent-to-treat
NOAEL	No observed adverse effect level
OBR	Optimized background regimen
Otsuka	Otsuka Pharmaceutical Co, Ltd
PET/CT	Positron emission tomography/computerized axial tomography
PK	Pharmacokinetics
PK/PD	Pharmacokinetics/pharmacodynamics
PQC	Product quality complaint
PT	Prothrombin time
PTT	Partial thromboplastin time
QD	Once daily
QTc	QT interval corrected
QTcF	QT interval corrected by Fridericia's method
RBC	Red blood cell
RHEZ	Rifampin, isoniazid, ethambutol, and pyrazinamide
RS ratio	Ribosomal ribonucleic acid synthesis ratio
SAE	Serious adverse event
SAP	Statistical analysis plan
SARS-CoV-2	Severe acute respiratory syndrome coronavirus
SCC	Sputum culture conversion
TB	Tuberculosis
TEAE	Treatment-emergent adverse event
TIW	Thrice-weekly
ULN	Upper limit of normal
WBC	White blood cell

10.5 Appendix 5: Schedule of Sputum Collections for All Treatment Arms

**Sample:**		**Sputum - MGIT Culture/ LAM**	**Sputum - MGIT Culture/ LAM**	**Sputum - RS Ratio**
**Collected by:**		Participant at home	Site staff	Site staff
Day	-14 to -2	-	X (eligibility assessment only)	-
	-1	-	X	X
	1	-	X	X
Week	1	X	X	X
	2	X	X	X
	3	X	X	X
	4	X	X	X
	5	X	X	X
	6	X	X	X
	7	X	X	X
	8	X	X	X
	10	X	X	X
	12	X	X	X
	13	X	X	X
	15	X	X	X
	17	X	X	X
	19	X	X	X
	21	X	X	X
	23	X	X	X
	26	X	X	X
	28	X	X	X
Week	30 to 52/ET	X	X	X

10.6 Appendix 6: Completer Category Information

Treatment completer, trial completer	Randomized participant who has taken at least one dose of IMP/RHEZ<br>Does not discontinue treatment in the treatment period (17weeks for IMP/26 weeks for RHEZ)<br>Has at least some assessments at the 17 week/26week end-of-treatment visit (completes end-of-treatment visit)<br>Continues on trial till the end-of-study visit at 12 months post-randomization; completes end-of-study assessments
Treatment non-completer, trial completer	Randomized participant who has taken at least one dose of IMP/RHEZ<br>Discontinues treatment prior to the end of the treatment period (17 weeks for IMP/26 weeks for RHEZ)<br>Continues on trial till the end-of-study visit at 12 months post-randomization; completes end-of-study assessments
Treatment completer, trial non-completer	Randomized participant who has taken at least one dose of IMP/RHEZ<br>Does not discontinue treatment in the treatment period (17 weeks for IMP/26 weeks for RHEZ)<br>Has at least some assessments at the 17week /26week end-of-treatment visit (completes end-of-treatment visit)<br>May or may not continue on trial after end-of-treatment visit.<br>Does not continue till the end-of-study visit at 12 months post-randomization; does not complete end-of-study assessments<br>Includes participants who do not complete 12 months of post-randomization follow-up for any reason:<br>participants who die after end-of-treatment visit;<br>participants LTFU after end-of-treatment visit;<br>participants who withdraw/are withdrawn from the study after end-of-treatment.
Treatment non-completer, trial non-completer	Randomized participant<br>not treated with IMP/RHEZ<br>OR<br>at least one dose of IMP/RHEZ<br>If study treatment was started, discontinues treatment prior to the end of the treatment period (17 weeks for IMP/26 weeks for RHEZ)<br>May or may not continue on trial after discontinuing treatment.<br>If continues on study, discontinues prior to end-of-study visit at 12 months post-randomization; does not complete end-of-study assessments at the 12-month timepoint.<br>Includes participants who:<br>participants who die/are LTFU/withdraw/are withdrawn prior to 17/26 weeks treatment period<br>participants who die/are LTFU/withdraw/are withdrawn after discontinuing treatment (prior to 17/26 weeks) but prior to 12 months post-randomization

10.7 Appendix 7: Protocol Amendments

The investigator will not make any changes to this protocol without the sponsor's prior written consent and subsequent approval/favorable opinion by the IRB/IEC. Any permanent change to the protocol, whether an overall change or a change for specific trial site(s), must be handled as a protocol amendment. Any amendment will be written by the sponsor. Each amendment will be submitted to the IRB/IEC, as required by local regulations. Except for "administrative" or "nonsubstantial" amendments, investigators will wait for IRB/IEC approval/favorable opinion of the amended protocol before implementing the change(s). Administrative amendments are defined as having no effect on the safety of participants, conduct or management of the trial, trial design, or the quality or safety of IMP(s) used in the trial. A protocol change intended to eliminate an apparent immediate hazard to participants should be implemented immediately after agreement by the sponsor and investigator, followed by IRB/IEC notification within local applicable timelines. The sponsor will submit protocol amendments to the applicable regulatory agencies within local applicable timelines.

When the IRB/IEC, investigators, and/or the sponsor conclude that the protocol amendment substantially alters the trial design and/or increases the potential risk to the participant, the currently approved written ICF will require similar modification. In such cases, after approval/favorable opinion of the new ICF by the IRB/IEC, repeat written informed consent will be obtained from participants enrolled in the trial before expecting continued participation and before the amendment-specified changes in the trial are implemented.

10.7.1 Protocol Amendment(s)/Administrative Change(s)

Please see Appendix 1 and Appendix 2.

11 References

[1] World Health Organization. Global tuberculosis report 2019. Geneva: World Health Organization; 2020.

[2] Sirturo (bedaquiline) [prescribing information]. Titusville, NJ: Janssen Therapeutics, Division of Janssen Products, LP; May 2020.

[3] Otsuka Pharmaceutical Co., Ltd. OPC-67683 Investigator’s Brochure, Edition 16. Otsuka Report, issued 07 Apr 2020.

[4] Gumbo T, Chapagain M, Magombedze G, Srivastava S, Deshpande D, Pasipanodya JG, et al. Novel tuberculosis combination regimens of two and three-months therapy duration. Submitted, Mar 2020.

[5] Otsuka Pharmaceutical Development and Commercialization, Inc. OPC-167832 Investigator’s Brochure, Edition 6, Otsuka Report, issued 20 Apr 2021.

[6] Aventis Pharma (Pty) Ltd. Rifafour e-275 tablets Package Insert. South African Electronic Package Inserts. Published 11 Feb 2002.

[7] Miettinen O, Nurminen M. Comparative analysis of two rates. Stat Med. 1985;4(2):213–226.

[8] United States Department of Health and Human Services, Division of AIDS, National Institute of Allergy and Infectious Diseases, National Institutes of Health. Division of AIDS (DAIDS) Table for Grading the Severity of Adult and Pediatric Adverse Events; 2017.

[9] European Medicines Agency, Science Medicines Health. ICH E9 (R1) addendum on estimands and sensitivity analysis in clinical trials to the guideline on statistical principles for clinical trials. London, England: European Medicines Agency; 2017.

Appendix 1 Protocol Amendment 1/Administrative Change(s)

Amendment Number: 1

Issue Date: 05 Aug 2021

PURPOSE:

The purpose of amending the Protocol 323-201-00006, issued 12 Jul 2021, was to:

- Allow for the recruitment of patients with HIV and TB into the trial.

BACKGROUND:

Changes to Protocol 323-201-00006 were made for clarification of details regarding participant eligibility for patients with HIV.

MODIFICATIONS TO PROTOCOL:

Description of Change	Rationale for Change	Sections Affected by Change
Evaluation of PK antiretroviral parameters has been removed	Expensive and under-powered to test for antiretroviral PK samples	Section 1.1 Synopsis<br>Section 3 Objectives and Endpoints
Removed sputum sample for eligibility at Day -1	No longer required for participant inclusion	Table 1.3 Schedule of Assessments
Testing for HIV viral load added to screening	To evaluate eligibility of HIV participants with TB to enter the trial	Table 1.3 Schedule of Assessments<br>Section 10.2 Clinical Laboratory Tests
Having a negative HIV status at screening was removed	To allow for the recruitment of HIV patients into this trial	Section 5.2.1 Inclusion Criteria
Having a history of HIV was removed	To allow for the recruitment of HIV patients into this trial	Section 5.2.2 Exclusion Criteria
Participants with HIV co infection who are on antiretroviral drugs during screening or with CD4 count < 350 cells/mm3 are excluded	Update exclusion criteria to clarify the enrollment of HIV patients	Section 5.2.2 Exclusion Criteria
The only antiretroviral therapy allow for this trial is tenofovir, emtricitabine/ lamivudine, dolutegravir. Furthermore, participants must be on a stable regimen for at least 3 months prior to screening.	Provide clarity on prohibited medication used during the trial	Section 6.5.1 Prohibited Medications or Therapies
Neurological exam and psychiatric assessment description have been removed	To clarify what assessments will take place during the physical exams	Section 8.7.2

ADDITIONAL RISK TO THE SUBJECT:

There is no additional risk to the participants.

A complete redline version of this amendment showing all changes from the previous version will be produced and will be available upon final approval of this amendment.

Appendix 2 Protocol Amendment 2/Administrative Change(s)

Amendment Number: 2

Issue Date: 08 Oct 2021

PURPOSE:

The purpose of amending the Protocol 323-201-00006, issued 12 Jul 2021, was to:

- Address site and investigator comments to the protocol
- Change "subjects" to "participants" per the request of the health authority.

BACKGROUND:

Changes to Protocol 323-201-00006 were made for clarification of details regarding the definition of completed participants individual participant discontinuation, and general clarity of the document.

MODIFICATIONS TO PROTOCOL:

Description of Change	Rationale for Change	Sections Affected by Change
Changed "subject" to "participant" throughout the document	At the request of the sites and health authority	Global change to protocol amendment
Removed PK endpoint from secondary objective	Population PK analysis will be reported separately	Section 1.1 Synopsis<br>Section 3 Objectives and Endpoints
Separated out exploratory and biomarker efficacy endpoints in trial objectives	To be clear about the use of biomarker exploratory efficacy endpoints	Section 1.1 Synopsis<br>Section 3 Objectives and Endpoints<br>Section 9.4.3
Updated language around definitions of trial completers versus non-completers	To be clear on which participants are considered completers and which are not	Section 4.5 Definition of Completed Participants
Updated text for individual participant discontinuation	To be clear on which participants are discontinued from the trial	Section 7.3 Individual Participant Discontinuation
Updated text for pregnancy information	To clarify the procedure for pregnant participants during the trial	Section 10.3 Appendix 3
General strategic updates to text based on investigator or site feedback	To align with peer reviewed comments to the document	Global change to protocol amendment

ADDITIONAL RISK TO THE SUBJECT:

There is no additional risk to the participants.

A complete redline version of this amendment 2 showing all changes from the previous version will be produced and will be available upon final approval of this amendment.

Agreement

I, the undersigned principal investigator, have read and understand the protocol (including the Investigator's Brochure) and agree that it contains all the ethical, legal and scientific information necessary to conduct this trial in accordance with the principles of Good Clinical Practices and as described herein and in the sponsor's (or designee's) Clinical Trial Agreement.

I will provide copies of the protocol to all physicians, nurses, and other professional personnel to whom I delegate trial responsibilities. I will discuss the protocol with them to ensure that they are sufficiently informed regarding the investigational new drug, OPC-167832, the concurrent medications, the efficacy and safety parameters and the conduct of the trial in general. I am aware that this protocol must be approved by the Institutional Review Board (IRB) or receive a favorable opinion by the Independent Ethics Committee (IEC) responsible for such matters in the clinical trial facility where OPC-167832 will be tested prior to commencement of this trial. I agree to adhere strictly to the attached protocol (unless amended in the manner set forth in the sponsor's Clinical Trial Agreement, at which time I agree to adhere strictly to the protocol as amended).

I understand that this IRB/IEC-approved protocol will be submitted to the appropriate regulatory authority/ies by the sponsor. I agree that clinical data entered on eCRF by me and my staff will be utilized by the sponsor in various ways, such as for submission to governmental regulatory authorities and/or in combination with clinical data gathered from other research sites, whenever applicable. I agree to allow sponsor and designee monitors and auditors full access to all medical records at the research facility for participants screened or enrolled in the trial.

I agree to await IRB/IEC approval before implementation of any substantial amendments to this protocol. If, however, there is an immediate hazard to participants, I will implement the amendment immediately, and provide the information to the IRB/IEC within the required local applicable timelines. Administrative changes to the protocol will be transmitted to the IRB/IEC for informational purposes only, if required by local regulations.

I agree to provide all participants with informed consent forms, as required by the applicable regulations and by ICH guidelines. I agree to report to the sponsor any adverse experiences in accordance with the terms of the sponsor's Clinical Trial Agreement and the relevant regional regulation(s) and guideline(s). I further agree to provide all required information regarding financial certification or disclosure to the sponsor for all investigators and subinvestigators in accordance with the terms of the relevant regional regulation(s). I understand that participation in the protocol involves a commitment to publish the data from this trial in a cooperative publication before publication of efficacy and safety results on an individual basis may occur, and I consent to be acknowledged in any such cooperative publications that result.

Principal Investigator Print Name	Signature	Date

Otsuka Pharmaceutical Development & Commercialization, Inc

Investigational New Drug quabodepistat (OPC-167832)

Protocol No. 323-201-00006
IND No. 129303

A Multicenter, Phase 2b/c, Open-label, Randomized, Dose-finding Trial to Evaluate the Safety and Efficacy of a 4-month Regimen of OPC-167832 in Combination with Delamanid and Bedaquiline in Participants with Drug-susceptible Pulmonary Tuberculosis in Comparison with Standard Treatment

Statistical Analysis Plan
Version 2: Final
Date: 4 June 2024
Protocol Date: 08 Oct 2021

Confidential
May not be used, divulged, published, or otherwise disclosed without the prior written consent of Otsuka Pharmaceutical Development & Commercialization, Inc.

Table of Contents

Title Page 1
Table of Contents 2
List of Tables 6
List of Figures 7
List of Appendices 8
List of Abbreviations and Definition of Terms 9
1 Introduction 11
2 Trial Objectives 11
2.1 Primary Objective 11
2.2 Secondary Objectives 11
2.3 Exploratory Objective - Biomarkers 11
2.4 Exploratory Objective - Efficacy 12
3 Trial Design 12
3.1 Type/Design of Trial 12
3.2 Trial Treatments 12
3.2.1 Treatment Descriptions 13
3.3 Trial Population 14
3.4 Trial Visit Window 14
4 Sample Size 14
5 Statistical Analysis Datasets 15
5.1 All Participants Set 15
5.2 Randomized Analysis Set 15
5.3 Modified Intent-to-Treat Analysis Set 15
5.4 Safety Analysis Set 16
5.5 Completers or Partial Completers Analysis Set 16
5.5.1 Completers or Potential Completers Analysis Set (CPC) 17
5.5.2 Completers or Potential Completers_8 Analysis Set (CPC_8) 17
5.6 Per Protocol Analysis Set 17
5.7 Pharmacokinetic Analysis Set 18

6 Handling of Missing Data 18

6.1 Missing Data for Sputum Culture Conversion 18

7 General Considerations for Statistical Analyses 19

7.1 Continuous Variables 19

7.2 Categorical Variables 19

7.3 Stratification Factors/Stratum 19

8 Primary and Secondary Outcome Variables 20

8.1 Primary Outcome Variables 20

8.1.1 Primary Efficacy Variable 20

8.1.2 Primary Safety Variables 22

8.1.2.1 Adverse Events 23

8.1.2.2 Clinical Laboratory Data 24

8.1.2.3 Vital Signs 24

8.1.2.4 Electrocardiogram Data 24

8.1.2.5 Physical Examination Data 25

8.1.2.6 Rate of All-Cause Treatment Discontinuation 25

8.2 Secondary Outcome Variables 25

8.2.1 Secondary Efficacy Variables 25

8.2.1.1 Sputum Culture Conversion in MGIT by 8 Weeks 26

8.2.1.2 Time to Positivity/Time to Detection of MGIT Cultures 26

8.2.1.3 Sputum Lipoarabinomannan Concentration 27

8.2.1.4 Drug Resistance 28

8.2.1.5 Time to Sputum Culture Conversion (SCC) 28

8.3 Exploratory Outcome Variables 29

8.3.1 Exploratory Efficacy Variables 29

8.3.1.1 Favorable Outcome 29

8.3.1.2 Relapse 30

8.3.2 Exploratory Biomarker Variables 30

9 Disposition and Demographic Analysis 31

9.1 Participant Disposition 31

9.2 Reasons for Discontinuation 31

9.3 Demographic and Baseline Characteristics 32

9.4 Medical History 32
9.5 Treatment Exposure and Compliance 32
9.6 Prior and Concomitant Medication 33
9.7 Rescue Treatment/Medications 34
9.8 Protocol Deviations 34
10 Efficacy Analyses 35
10.1 Primary Efficacy Analyses 35
10.2 Secondary Efficacy Analyses 36
10.2.1 Sputum Culture Conversion in MGIT by 8 Weeks 36
10.2.2 Time to Positivity/Time to Detection of MGIT Cultures 36
10.2.3 Sputum Lipoarabinomannan 36
10.2.4 Drug Resistance 36
10.2.5 Time to Sputum Conversion 37
10.3 Exploratory Efficacy Analyses 37
10.4 Exploratory Biomarker Analyses 38
10.5 Sensitivity Analyses 38
10.6 Subgroup Analyses 39
11 Safety Analyses 39
11.1 Extent of Exposure 39
11.2 Adverse Events 39
11.3 Clinical Laboratory Data 40
11.4 Vital Sign Data 40
11.5 Electrocardiogram Data 41
11.6 Physical Examination Data 41
11.7 Clinical Signs and Symptoms of Tuberculosis 41
11.8 Other Safety Data 42
12 Pharmacokinetic and Pharmacodynamic Analyses 42
13 Pharmacogenomic Analyses 42
14 Bioavailability Analyses 42
15 Interim Analyses 42
16 Data Monitoring Committee Analyses 43

17 Changes in Planned Analysis 43
18 Document History 44
19 References 47
20 Appendices 48

List of Tables

Table 3.2.1-1 Rifinah Dosing Table..14
Table 8.1.1-1 Baseline Visit and Baseline Visit Result Determination 20
Table 8.1.1-2 Interpretation of (Regular) MGIT Sputum Culture Results 22

List of Figures

Figure 3-1 Trial Design Schematic..12

List of Appendices

Appendix 1 Definition of Grades in Serum Chemistry, Hematology and Urinalysis Lab Test Results in the DAIDS Table for Grading the Severity of Adult and Pediatric Adverse Events 49

Appendix 2 Definition of Grades for Vital Signs in the DAIDS Table for Grading the Severity of Adult and Pediatric Adverse Events 53

Appendix 3 Sponsor Provided Criteria for Identifying Vital Sign Measurements of Potential Clinical Relevance 54

Appendix 4 Sponsor Provided Criteria for Identifying ECG Measurements of Potential Clinical Relevance 55

Appendix 5 Completer Category Information..58

Appendix 6 Missing Data for Adverse Events/Concomitant Medications 60

Appendix 7 SAS Code...62

Appendix 8 Determination of 6-month Post End-of-Treatment Favorable/Unfavorable/Not Assessable Outcome.........................63

List of Abbreviations and Definition of Terms

Abbreviation	Definition
ACD	actual cumulative dose
ADI	actual dose intensity
AE	adverse event
BDQ	Bedaquiline
BMI	body mass index
CI	confidence interval
CPC	completers or potential treatment completers
CRF	case report form
CSR	clinical study report
CT	computerized axial topography
DAIDS	Division of AIDS
DBO	delamanid, bedaquiline, OPC-167832 (quabodepistat)
DCO	data cutoff date
DLM	Delamanid
DMC	data monitoring committee
DOT	directly observed therapy
DS-TB	drug-susceptible pulmonary tuberculosis
ECG	Electrocardiogram
FO	favorable outcome
HEENT	head, eyes, ears, nose and throat
HIV	human immunodeficiency virus
IA	interim analysis
ICH	International Council for Harmonisation
IMP	investigational medicinal product (includes any delamanid, bedaquiline and quabodepistat [OPC-167832] and any rifampin, isoniazid, ethambutol, pyrazinamide) provided to a participant as study medication
IRT	interactive response technology
KM	Kaplan-Meier
LAM	Lipoarabinomannan
LTFU	lost to follow-up
MGIT	Mycobacteria Growth Indicator Tube®
MIC	minimum inhibitory concentration
mITT	modified intent-to-treat
MTB/MTb	Mycobacterium tuberculosis
PCR	potential completers
PCS	potentially clinically relevant
PDI	planned dose intensity
PET	positron emission tomography
PK	Pharmacokinetic
PP	per protocol
QD	Daily
RDI	relative dose intensity
RHEZ	rifampin, isoniazid, ethambutol, pyrazinamide
RS	rRNA synthesis ratio
SAE	serious adverse event
SAP	statistical analysis plan
SCC	sputum culture conversion
SD	standard deviation

Abbreviation	Definition
SLC	sputum lipoarabinomannan conversion
TB	Tuberculosis
TIW	three times weekly
TEAE	treatment-emergent adverse event
TTD	time to detection
TTP	time to positivity
w	With
wo	Without
wwom	with and without mitigation

1 Introduction

This Statistical Analysis Plan (SAP) describes the statistical methodology and data analysis algorithms and conventions to be applied for statistical analysis and reporting of safety and efficacy data of Trial 323-201-00006. This SAP is based on Amendment 2 of the Protocol dated 08 Oct 2021 (Amendment 3 primarily intended to change eligibility to increase enrolment, but since enrolment was completed in the study it was rescinded and not implemented).

Trial 323-201-00006 is a phase 2 trial for quabodepistat (OPC-167832) that is being developed for the treatment of pulmonary tuberculosis, an infection caused by *Mycobacterium tuberculosis*. The statistical analyses and summary tabulations described in this SAP will provide the basis for all the analyses conducted during the course of the trial and also for the final analysis outputs with the results and appendices of the clinical study report (CSR) for this study.

2 Trial Objectives

2.1 Primary Objective

The primary objective of this trial is to assess the safety and efficacy of quabodepistat (OPC-167832) combined with delamanid (DLM) and bedaquiline (BDQ) in participants with drug-susceptible pulmonary tuberculosis (DS-TB) administered for 4 months compared to standard of care administered for 6 months (RHEZ).

2.2 Secondary Objectives

- To evaluate the efficacy using sputum Mycobacteria Growth Indicator Tube® (MGIT) culture and lipoarabinomannan (LAM) concentration for quabodepistat (OPC-167832) combined with DLM and BDQ administered for 4 months as compared to RHEZ.
- To evaluate the development of resistance to anti-tuberculosis (TB) drugs in all treatment arms.
- To inform dose selection of quabodepistat (OPC-167832) combined with DLM and BDQ.

2.3 Exploratory Objective - Biomarkers

- To evaluate various novel biomarkers for association with microbiological and clinical response.

CCI [redacted]

3 Trial Design

3.1 Type/Design of Trial

Trial 323-201-00006 is a multicenter, phase 2b/c, open-label, randomized, dose-finding trial in participants with DS-TB. The microbiology laboratory will be blinded to treatment assignments. Following a screening period of up to 14 days, eligible participants will be randomized on Day −1 or predose Day 1 in a ratio of 1:2:2:1 to one of the four treatment arms (Figure 3-1). Randomization will be stratified by human immunodeficiency virus (HIV) status (yes or no) and presence of bilateral cavitation on screening chest x-ray (yes or no). At the end of the treatment period, participants will be followed until 12 months post randomization.

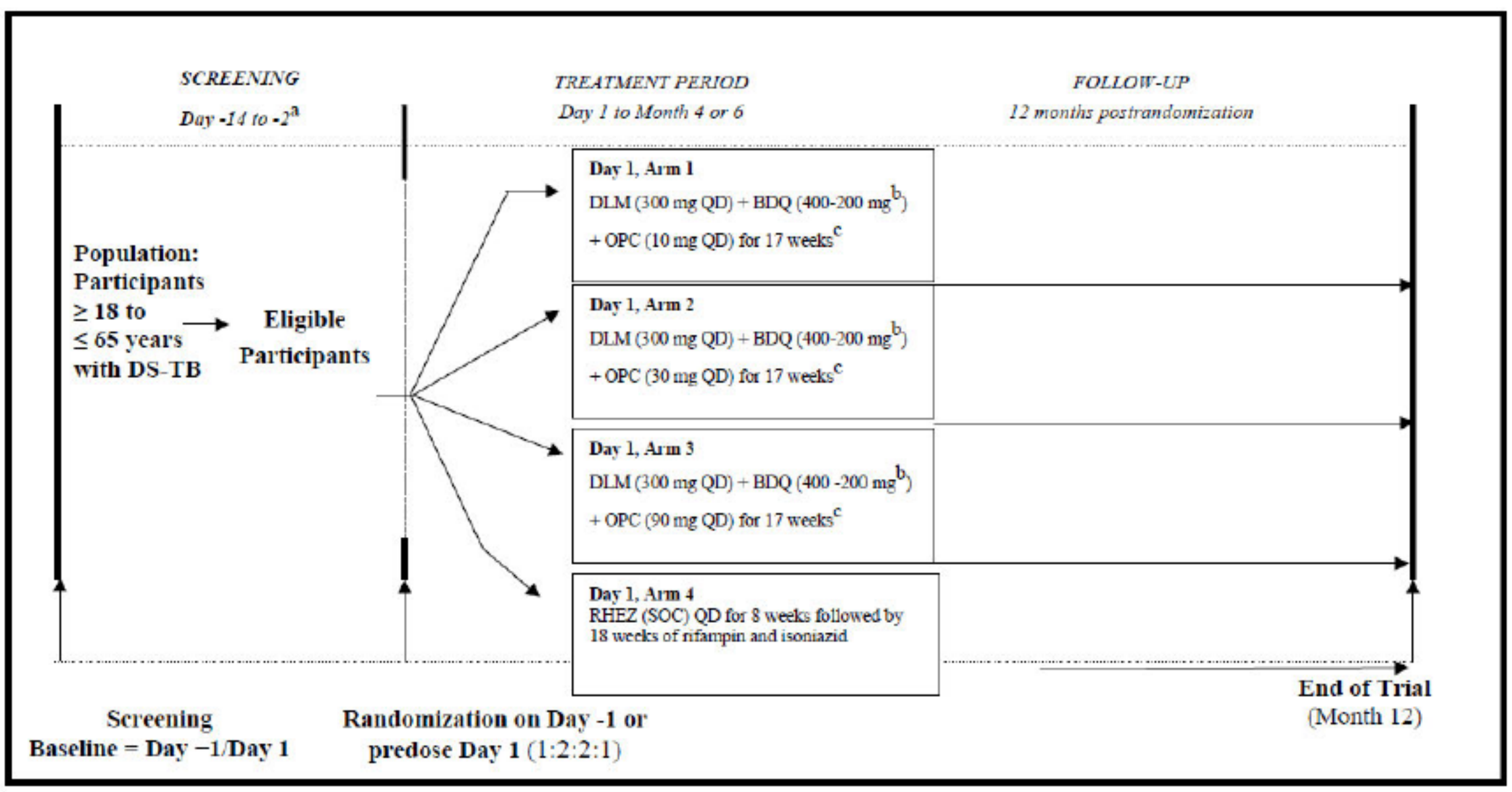

Figure 3-1 Trial Design Schematic

3.2 Trial Treatments

Treatment Arms 1, 2, and 3:

Investigational medicinal product (IMP) (i.e. quabodepistat [OPC-167832], DLM, or BDQ) in Arms 1-3 must be administered after the completion of a meal.

Bedaquiline should be administered as follows: 400 mg daily (QD) for the first 2 weeks of treatment and 200 mg three times weekly (TIW) thereafter with at least 48 hours between doses for the remainder of the trial (i.e., a total of 17 weeks [Arm 1 or Arm 2 or Arm 3] of treatment).

IMP will be administered using the locally accepted adherence monitoring techniques which could include directly observed therapy (DOT), Wisepill, video DOTs, or other adherence monitoring techniques. In addition, all doses of IMP given during trial visits will be observed via the hand-and-mouth procedure. IMP will be administered with approximately 240 mL of still (noncarbonated) water.

Treatment Arm 4:

Participants randomized to treatment Arm 4 will receive RHEZ orally QD for 8 weeks followed by 18 weeks of rifampin and isoniazid per the local standard of care. RHEZ will be administered using the locally accepted adherence monitoring techniques which could include DOT, Wisepill, video DOTs, or other adherence monitoring techniques. In addition, all doses of RHEZ given during trial visits will be observed via the hand-and-mouth procedure. RHEZ will be administered with approximately 240 mL of still (noncarbonated) water. The RHEZ will be taken 1 hour prior to or 2 hours after a meal.

3.2.1 Treatment Descriptions

Arm 1: DLM (300 mg once daily [QD]) + BDQ (400 mg QD × 2 weeks, then 200 mg thrice - weekly [TIW]) + OPC-167832 (10 mg QD) for 17 weeks

Arm 2: DLM (300 mg QD) + BDQ (400 mg QD × 2 weeks, then 200 mg TIW) + OPC-167832 (30 mg QD) for 17 weeks

Arm 3: DLM (300 mg QD) + BDQ (400 mg QD × 2 weeks, then 200 mg TIW) + OPC-167832 (90 mg QD) for 17 weeks

Arm 4: RHEZ for 8 weeks followed by 18 weeks of rifampin and isoniazid (for a total of 26 weeks)

Dosing of Rifafour® (RHEZ) will be determined by the participant's weight assessed at each visit where vital signs and weight are assessed.

Weight will be rounded to the next whole number (e.g., 1.1-1.4 = 1; 1.5-1.9 = 2).

Dosing will be based on the participant's weight:

- Participants weighing 30 to 37 kg will receive 2 tablets per day.
- Participants weighing 38 to 54 kg will receive 3 tablets per day.
- Participants weighing 55 to 70 kg will receive 4 tablets per day.

- Participants weighing > 70 kg will receive 5 tablets per day.

Dispense sufficient RHEZ tablets into a clearly labelled container for the subject to complete treatment for the next week and allow for the window period. The label should contain name RHEZ, protocol number, the site name and address, instructions for use, route of administration, and appropriate precautionary statements.

Dosing of Rifinah will be determined by the participant's weight assessed at each visit where vital signs and weight are assessed (see Table 3.2.1-1). Weight will be rounded to the next whole number (e.g., 1.1-1.4 = 1; 1.5-1.9 = 2).

Table 3.2.1-1 Rifinah Dosing Table

Participant weight	Number of tablets daily	
	RIFINAH 150/75	RIFINAH 300FC
30 – 37 kg	2	-
38 – 54 kg	3	-
≥55 kg	-	2

3.3 Trial Population

The trial population will consist of participants who are ≥ 18 years to ≤ 65 years at screening with body weight of ≥ 35.0 kg and who have newly diagnosed pulmonary DS-TB. Approximately 120 participants (including no more than 15% with HIV-coinfection) will be enrolled at approximately 8 sites in South Africa.

3.4 Trial Visit Window

During treatment for Week 1 through Week 26 (Visits 3 through 20), the visit can be target day ± 2 days. During the follow-up period of Week 28 through Week 52 (Visits 21 through 27), the visit can be target day ± 7 days.

4 Sample Size

It is planned to enroll enough participants so that there will be 20 participants in the DLM + BDQ + 10 mg OPC-167832 arm, 40 participants each in the DLM + BDQ + 30 mg OPC-167832, and DLM + BDQ + 90 mg OPC-167832 arms, and 20 participants in the RHEZ group. This is a pilot trial and exploratory in nature. Thus, 80% CIs between proportion differences will be calculated to compare with noninferiority margin. Assuming that the sputum culture conversion (SCC) rate at the end-of-treatment for the primary endpoint is 93% for the RHEZ group, and assuming the SCC rate of the quabodepistat (OPC-167832) arms is identical to the RHEZ group, with a noninferiority margin of 12%, a sample size of 120 participants (100 for pooled the DLM + BDQ + OPC-167832 arms and 20 for RHEZ) will have about 84% power to reject the null

hypothesis with a one-sided alpha level of 0.1, i.e., the lower limit of the two-sided 80% CI of the difference in proportions of sputum culture conversion between the pooled investigational arms and RHEZ is larger than −0.12. The Miettinen and Nurminen method to construct 80% CI was used in the sample size calculation. Assuming 11% randomized participants will not demonstrate at least 1 positive culture result by Week 1 and susceptibility to rifampin and isoniazid, approximately 135 participants will be randomized in 1:2:2:1 ratio to obtain 120 participants in the modified intent-to-treat (mITT) analysis set.

5 Statistical Analysis Datasets

5.1 All Participants Set

The All Participants set will include all enrolled participants, that is, participants who have given signed informed consent. Screen failures includes participants who signed informed consent but were not randomized into the trial either because they did not meet the eligibility criteria or for any other reason (such as the study recruitment was complete). This analysis set will be used for inclusion/exclusion criteria, and screen failures.

5.2 Randomized Analysis Set

This analysis set comprises all enrolled participants who are randomized into the trial. Participants are considered randomized when they are assigned a randomization number and are assigned to a treatment arm.

This will be used for protocol deviations, participant disposition and medical history.

5.3 Modified Intent-to-Treat Analysis Set

The modified intent-to-treat (mITT) analysis set includes randomized participants that have met all the below:

- have taken at least 1 dose of IMP or RHEZ,
- have at least 1 postbaseline culture result,
- demonstrate positive culture results by Week 1,
- demonstrate susceptibility to rifampin and isoniazid (from the screening specimen).

In the case where Week 1 is the baseline for MGIT cultures, then a post baseline culture will be defined as Week 2 or later. The mITT analysis set will be used for efficacy analyses. Tables on the mITT analysis set are presented according to the arm to which a participant was randomized (the planned treatment), regardless of actual treatment received.

5.4 Safety Analysis Set

The safety analysis set includes all randomized participants who take any dose of IMP (Arms 1 – 4). Participants will only be excluded from this population if the participant did not receive any IMP. Safety analysis will be presented by actual treatment received.

Safety analysis will be conducted based on the safety analysis set.

5.5 Completers or Partial Completers Analysis Set

Definition of Completed Participants

The treatment period is defined as the time period during which participants are evaluated for primary and/or secondary objectives of the trial irrespective of whether or not the participant actually took all doses of the IMP. For the purpose of this trial, treatment completers will be randomized participants who take IMP through either Week 17 (for Arm 1, Arm 2, and Arm 3) or Week 26 (for Arm 4) and complete at least some of the end-of-treatment assessments. Trial completers will be randomized participants who remain on the study till the 12 months post randomization visit.

Completer categories are as follows:

- Treatment completer and trial completer
- Treatment non-completer and trial completer
- Treatment completer, trial non completer
- Treatment non-completer and trial non-completer.

Details are as in Appendix 5 Completer Category Information

Note that "treatment completer" and "trial completer" are definitions requiring the participant to have been on the study for the entire treatment duration and trial duration respectively. Participants who discontinue or die would not be entered as "completed" in the CRF disposition page. The participant disposition table will be based on the disposition entered for the participant in the CRF, and the treatment or trial completer status as assessed by the criteria listed in Appendix 5 Completer Category Information

5.5.1 Completers or Potential Completers Analysis Set (CPC)

This analysis set will be used for analyses prior to final database lock, where a data cutoff has to be applied. Participants with ongoing treatment at data cutoff are excluded from the Completers or Potential Completers (CPC) analysis set.

The CPC analysis set comprises of mITT participants who are also treatment completers or potential treatment completers. Treatment completers are participants who have been exposed to IMP and do not discontinue treatment during the treatment period (17 weeks for quabodepistat (OPC-167832) arms/26 weeks for RHEZ arm). Potential completers are participants who could have completed treatment (by data cutoff as applicable for Data Monitoring Committee [DMC]/interim analysis [IA]), had they not discontinued treatment, died or become lost-to-follow-up prior to completing treatment.

Note that for data cutoffs that are post end-of- treatment, (or potential end-of-treatment for discontinuing participants) for all participants, reports on the CPC analysis set will not be generated.

5.5.2 Completers or Potential Completers_8 Analysis Set (CPC_8)

This analysis set will be used for the "8 weeks" analyses prior to final database lock, where a data cutoff has to be applied.

The CPC_8 analysis set comprises of mITT participants who are also 8-week treatment completers or potential 8-week treatment completers. 8-week treatment completers are participants who have been exposed to IMP and do not discontinue treatment in the first 8 weeks of the treatment period. Potential 8-week completers are participants who could have completed 8 weeks of treatment by data cutoff, had they not discontinued treatment, died or become lost-to-follow-up prior to completing the 8-weeks on treatment. Only participants who have not completed 8-weeks of treatment by data cutoff, that is, are ongoing participants at data cutoff, are excluded from the CPC_8 analysis set.

Note that for data cutoffs that are post 8-weeks treatment (or potential 8-week treatment completion) for all participants, reports on the CPC_8 analysis set will not be generated.

5.6 Per Protocol Analysis Set

The per protocol (PP) analysis set comprises of participants from mITT analysis set excluding those satisfying any of the criteria mentioned below:

- patients who do not meet eligibility criteria.
- participants who are found to have less than or equal to 80% adherence of any of the IMP or discontinued IMP for a reason other than death, TB or TB-related

disease, or are who are determined to have major treatment adherence protocol violations.

- patients with major protocol deviations that impact primary efficacy analyses.

The final decision on the PP analysis set will be taken by the medical lead, clinical operations lead, and statistician. Participants will be analyzed according to actual treatment received.

The PP analysis set will be used for sensitivity analyses of select efficacy data.

Reasons for excluding a participant from the PP analysis set will be summarized and listed.

In addition to select efficacy analyses, analyses such as disposition, demographics and baseline characteristics, and exposure tables will be generated for comparability of treatment arms for the PP analysis set.

5.7 Pharmacokinetic Analysis Set

The pharmacokinetic analysis set will include all participants with adequate data for the population pharmacokinetic (PK) analysis.

6 Handling of Missing Data

Unless specified in this SAP, all data will be evaluated as observed, and no imputation method for missing values will be used. If values are imputed for missing data, these will be flagged in listings. Situations in which data may be imputed is specified in the handling of missing data Section 6.1 and efficacy analysis Section 10.

Missing dates for dates of Concomitant Medications and Adverse Events will be imputed as described in

Appendix 6 Missing Data for Adverse Events/Concomitant Medications

6.1 Missing Data for Sputum Culture Conversion

All randomized participants will be sputum culture positive at baseline.

Modified intent-to-treat (mITT) participants who discontinue or die prior to treatment completion, will be assessed based on the MGIT sputum culture assessments till the date of discontinuation or death. mITT participants who remain on the trial till the end-of-treatment but have two or more missing consecutive sputum culture assessments at the

end of treatment, will be considered as not having achieved SCC (SCC = “No”) for the end-of-treatment endpoint.

If there are missing cultures between 2 negative sputum cultures, the definition of conversion is still met, and the participant will be counted as having SCC.

Contaminated sputum cultures and Mycobacterium other than Tuberculosis will be defined as missing culture assessments for the primary analyses.

7 General Considerations for Statistical Analyses

General considerations for descriptive statistics and presentation for continuous and categorical data are given below.

All analyses will be presented by the 4 treatment arms and overall. In addition, for a few select efficacy tables, summaries for the pooled quabodepistat (OPC-167832) arms may also be included.

7.1 Continuous Variables

Continuous data will be described using descriptive statistics: number of observations (n), mean, standard deviation, median, minimum, and maximum.

Means, medians, and CIs will be reported to one decimal place more than the data reported on the CRF or by the vendor. Standard deviation (SD) will be reported to two decimal places more than the data reported on the CRF or by the vendor. Minimum and maximum will be reported to the same number of decimal places displayed on the CRF or by the vendor. P-values will be reported to 4 decimal places. Heart rates and ECG will be presented without any decimals for all summary statistics. The average of triplicate ECGs will be presented rounded to the nearest integer.

7.2 Categorical Variables

For categorical variables, the counts and proportions (as percentages) of each category for each parameter will be calculated. Percentages will be presented with one decimal place. The denominator for all percentages, unless otherwise specified, will be the number of participants in the specified treatment arm, overall group or cohort (e.g., subgroup) in the specified analysis population.

7.3 Stratification Factors/Stratum

Randomization will be stratified by HIV status (yes or no) and presence of bilateral cavitation on screening chest x-ray (yes or no). If there is a stratum of very small size, strata may be collapsed. For instance, if there are less than 5 HIV participants randomized

into the study in the OPC-167832 arms combined, or in the RHEZ arm, then the analysis will not use this stratification variable to adjust the estimates, similarly for bilateral cavitation status.

Descriptive analyses may be presented by stratification factors wherever needed. When statistical models are applied, the analysis may be done with and without adjustment for stratification factors (or collapsed stratification factors, as the case may be). Analytical methods will be specified in presentations.

Strata identification will be based on CRF data.

8 Primary and Secondary Outcome Variables

8.1 Primary Outcome Variables

8.1.1 Primary Efficacy Variable

Efficacy will be assessed by the proportion of participants achieving SCC in Mycobacteria Growth Indicator Tube® (MGIT) by the end of the treatment period. The treatment period is 17 weeks for arms with quabodepistat (OPC-167832) and 26 weeks for the RHEZ arm. Participants in the OPC-167832 arms will be pooled and compared to those in the RHEZ arm. The baseline for sputum culture will be Day 1 if the Day 1 result is positive. If the Day 1 result is negative, then the Day −1 result will be used as baseline. If the Day −1 result is also negative then the Week 1 result will be used as baseline, which in this case, must be positive. The treatment period will be from baseline to the end of the treatment period (last dose date of IMP). Below is an example decision table to determine the baseline visit result.

Note that SCC is a binary endpoint.

Table 8.1.1-1 Baseline Visit and Baseline Visit Result Determination

Visit	Results							
Day −1	Positive	Negative	Negative	Negative	Positive	Positive	Positive	Negative
Day 1	Negative	Positive	Negative	Positive	Negative	Positive	Positive	Negative
Week 1	Negative	Negative	Positive	Positive	Positive	Negative	Positive	Negative
**Baseline Visit**	**Day −1**	**Day 1**	**Week 1**	**Day 1**	**Day −1**	**Day 1**	**Day 1**	**-**
**Baseline Visit Result**	**Positive**	**Positive**	**Positive**	**Positive**	**Positive**	**Positive**	**Positive**	**-**

A participant with negative sputum culture results at all three timepoints (last column) will be deemed ineligible for the mITT analysis set used for the efficacy analysis.

As per protocol, up to 3 MGIT samples will be collected at each visit except on Day −1 and Day 1, when only two samples will be collected. Only two of the samples are used for MGIT analysis. The third sample is used for rRNA synthesis ratio (RSA). MGIT culture will be performed on sputum specimen without (wo) any mitigation method applied and also with (w) a mitigation method applied.

For primary analysis of SCC, time to detection (TTD) or Kaplan-Meier (KM) plots, only the two MGIT samples without mitigation will be considered, i.e., (regular) MGIT. A sensitivity analyses will be performed using all 4 MGIT samples with and without mitigation (wwom).

For the primary analyses the following rules will be used with regular MGIT samples, at each visit:

- Both samples have a result, and this result is not "contaminated" or "other" as recorded on the CRF:
    - both results are positive for *Mycobacterium tuberculosis* (MTB) complex, the SCC result will be positive.
    - both results are negative for MTB complex, the SCC result will be negative.
    - one result for MTB complex is positive and one is negative, the result will be positive.
- If both samples are missing or both are contaminated/other, the result will be missing.
- If one sample is "contaminated"/"other"/or is missing, and the other is positive, the result will be positive.
- If one sample is "contaminated"/"other"/or is missing, and the other is negative, the result will be negative.

See Table 8.1.1-2 for interpretation of each sample.

Sensitivity analyses interpreting CRF results differently from what is presented in Table 8.1.1-2, may be considered to assess worst case and best-case scenarios. Details of these analyses will be specified in programming specifications.

Definition of sputum culture conversion (SCC):

SCC occurs when a participant has the first of 2 consecutive visits of at least 7 days apart with sputum culture assessment interpretations as “negative”, without a positive sputum culture result in between and no subsequent positive sputum culture during the treatment period (either end-of-treatment or 8 weeks). The date of collection for the first sample out of these 2 consecutive samples that is negative is considered as the date of conversion. If there are missing or contaminated/other culture results between 2 negative sputum culture assessments, the definition of conversion is still met.

The numerator of the proportion of participants will be the number of mITT participants who have achieved SCC by the end of the treatment period within the quabodepistat (OPC-167832) arms or the RHEZ arm. The denominator will be the total number of mITT participants within the arms being compared. See Section 6.1 for handling of missing data for SCC.

The MGIT cultures will be interpreted as follows:

Table 8.1.1-2 Interpretation of (Regular) MGIT Sputum Culture Results

CRF Results	Interpretation
Positive for MTB Complex	Positive
Positive for MTB Complex with Contamination	Positive
Mycobacteria other than MTB	Negative
Negative for MTB Complex	Negative
Contaminated	Missing
Missing results	Missing
Other	Missing

8.1.2 Primary Safety Variables

Safety and tolerability will be assessed based on the following data:

- Incidence of adverse events (AE), serious adverse events (SAE), treatment-emergent adverse events (TEAE), serious treatment emergent adverse events, AEs leading to discontinuation and TEAEs leading to discontinuation, Related TEAEs, TEAEs leading to Death, TEAEs by Severity (described in Section 8.1.2.1).
- The incidence of Potentially Clinically Relevant abnormal findings in clinical laboratory tests (serum chemistry, hematology, urinalysis, and coagulation) (described in Section 8.1.2.2).
- Potentially Clinically Relevant findings and abnormalities in vital signs (described in Section 8.1.2.3).
- Incidence of Potentially Clinically Relevant Abnormalities in Electrocardiogram Parameters (described in Section 8.1.2.4).
- Electrocardiograms (ECGs) (described in Section 8.1.2.4).
- Physical examinations (described in Section 8.1.2.5).

- Incidence of TEAEs with a DAIDS Toxicity grade >= 3 (described in Section 8.1.2.1).
- Rate of all-cause treatment discontinuation (described in Section 8.1.2.6).

8.1.2.1 Adverse Events

AEs will be collected from the signing of informed consent to the end of trial visit after the follow-up period or till complete withdrawal of consent. Serious adverse events are followed until resolution, stabilization, lost to FU or death.

All AEs will be coded by Medical Dictionary for Regulatory Activities (MedDRA; Version 25.0 or newest available) system organ class and preferred term.

Treatment-emergent AEs (TEAE) are defined as AEs with an onset date on or after the start of treatment with the IMP. In more detail, TEAEs are all AEs which started after the start of IMP treatment; or if the event was continuous from baseline and was worsening, serious, IMP related, or resulted in death, discontinuation, interruption, or reduction of IMP.

A serious adverse event is defined as an AE that leads to death, is life threatening, requires or prolongs hospitalization, significant disability, congenital anomaly or birth defect, and other medically important serious advent.

Non-serious AEs are AEs not classified as “Serious”.

Causality to IMP is classified as: related; not related.

AEs are graded in two ways. First, on a severity 3-point scale as follows: Mild; Moderate; Severe. In addition, all AEs will be graded on a 5-point scale according to DAIDS Table for Grading the Severity of Adult and Pediatric AEs[1] as follows: Grade 1 – Mild; Grade 2 – Moderate; Grade 3 – Severe; Grade 4 – Life Threatening; Grade 5 – Death.

Outcome of AE is classified as: Not recovered/not resolved; Recovered/resolved; Recovering/resolving; Recovered/resolved with sequelae; Fatal; Unknown.

Action taken with Study Treatment is classified as: Dose not changed; Dose reduced; Drug interrupted; Drug withdrawn; Not applicable.

Reporting of Adverse Events is described in Section 11.2.

8.1.2.2 Clinical Laboratory Data

Clinical hematology, chemistry, coagulation and urinalysis are tested at: Screening, Day −1, Week 2, Week 4, Week 6, Week 8, Week 13, Week 17, Week 21, Week 26, Week 52, and end of study.

For clinical laboratory tests data, baseline is defined as the last non-missing value obtained at a scheduled visit on or before Day −1. If the Day −1 measurement is missing, the baseline value will be the non-missing measurement from the screening visit (the previous scheduled visit). Unscheduled visits will only be presented in the listings.

Reporting of Clinical Laboratory Data is described in Section 11.3.

8.1.2.3 Vital Signs

Vital signs are taken at: Screening, Day −1, Week 2, Week 4, Week 6, Week 8, Week 10, Week 12, Week 13, Week 15, Week 17, Week 21, Week 26, Week 28, Week 30, Week 34, Week 39, Week 43, Week 47, Week 52, and end of study.

Vital signs include systolic and diastolic blood pressure (mmHg), heart rate (bpm), respiratory rate (rpm), body temperature (C), height (cm) (only at screening), weight, and body mass index (BMI). Baseline is defined as the last non-missing value obtained at a scheduled visit on or before Day −1. If the Day −1 measurement is missing, the baseline value will be the non-missing measurement from the screening visit (the previous scheduled visit).

Vital signs will be obtained prior to PK blood draws and ECGs. At screening, blood pressure and heart rate will be taken with the participant in the supine (performed first) and sitting position after remaining for ≥ 3 minutes in each position and temperature and respiratory rate will be taken with the participant in the supine position. At all other time points, vital signs will be obtained after the participant has been at rest in the supine position for at least 1 minute. Unscheduled visits will only be presented in the listings.

Reporting of Vital Signs is described in Section 11.4.

8.1.2.4 Electrocardiogram Data

ECG measurements are taken at: Screening, Day −1, Week 2, Week 4, Week 6, Week 8, Week 10, Week 12, Week 13, Week 15, Week 17, Week 21, Week 26, Week 30, Week 34, Week 39, Week 43, Week 47, Week 52, and end of study.

One standard 12-lead ECG will be performed during screening, plus 3 ECGs at baseline (Day −1) spaced 5 to 10 minutes apart, and 3 ECGs at all subsequent visits during the treatment and follow-up periods. Baseline is defined as the last non-missing value

obtained at a scheduled visit on or before Day −1. Findings detected by the investigator will also be categorized as baseline using the last non-missing value obtained at a scheduled visit on or before Day −1. Where multiple ECGs are required in a visit, the participant should be recumbent for 10 minutes prior, if possible, and ECG should be collected 5 to 10 minutes apart after the dose of IMP and prior to PK blood draws. If additional ECGs were obtained at a time point at a site, all of the ECGs obtained at the time point will be averaged to provide for a more accurate point estimate of ECG intervals at that time point. If there are multiple ECG interpretation records, if one is Abnormal then the overall classification is Abnormal. The number of records with abnormal interpretation will be included in parentheses (x/n) where x is the number of "abnormal" interpretation records and n is the total number of records. This will be included in listings of ECG data. Unscheduled visits will only be presented in the listings.

Reporting of ECG data is described in Section 11.5. Refer to Section 7.1 for handling of decimal values in ECG.

8.1.2.5 Physical Examination Data

Visual assessments to be completed during screening, Week 17 or Week 26 will consist of Snellen and Ishihara testing and will be repeated during the trial if the investigator suspects abnormalities.

Reporting of Physical Examination Data is described in Section 11.6.

Visual assessments will occur at screening, Week 17 (for participants in Arm 1, Arm 2, and Arm 3), or Week 26 (for participants in Arm 4). They will consist of Snellen and Ishihara testing and will be repeated during the trial if the investigator suspects abnormalities.

8.1.2.6 Rate of All-Cause Treatment Discontinuation

All-cause treatment discontinuation will be summarized by treatment arm and overall. Reasons for treatment discontinuation are: adverse event, physician decision, protocol deviation, withdrawal of consent by the participant and other. The "other" category has an "other specify" option, and these reasons, collected in the free text field, will also be tabulated.

All-cause reasons for discontinuation will be listed.

8.2 Secondary Outcome Variables

8.2.1 Secondary Efficacy Variables

The secondary efficacy endpoints are as follows:

- The proportion of participants in each arm who achieve SCC in MGIT by 8 weeks of treatment.
- Time to detection of MGIT cultures.
- The proportion of participants in each arm who convert sputum LAM to negative by 8 weeks of treatment and by end-of-treatment.
- Proportion of participants who develop drug resistance.
- Time to SCC.

8.2.1.1 Sputum Culture Conversion in MGIT by 8 Weeks

The proportion of participants who achieve SCC in MGIT by 8 weeks of treatment will be assessed using the same methods as the primary endpoint. The analysis period is from baseline to the Week 8 visit sputum MGIT culture assessment date.

SCC analyses in MGIT by 8 weeks analysis will be performed using the two MGIT assessments without mitigation (regular). A sensitivity analysis, with and without mitigation analysis (wwom), will be performed using all four samples (two each with and without mitigation).

The statistical analysis for SCC in MGIT by 8 weeks is described in Section 10.2.1

8.2.1.2 Time to Positivity/Time to Detection of MGIT Cultures

Time to positivity (TTP)/Time to detection of MGIT cultures will be performed using the two MGIT assessments without mitigation. A sensitivity analysis will be performed using all four samples (two each with and without mitigation).

Note that the term “time to positivity” is used interchangeably with the term “time to detection of a positive signal (TTD)”.

The TTP values in Days will be calculated as “days + hours/24” to be used in deriving the analysis values of TTP. If the TTP value is 42 days and x hours, (with x > 0) or more than 42 days, (say 44 days), then TTD will be capped at 42 days.

TTP values from MGIT cultures with the following qualitative results will be checked for consistency. For MGIT culture results below, the TTP value should be classified as missing:

- Contaminated
- Positive for MTB complex and contaminated
- No TB growth but positive for other mycobacteria
- Unknown

The TTP value summarized will be derived as follows:

TTP_tabulated = minimum (time to positivity over all records with a non-missing value for TTP for a participant-visit both with and without mitigation).

Baseline TTP will be derived as follows:

Day 1 value = minimum (over all non-missing TTP records for participant-Day 1) [there are 2 records].

Day −1 value = minimum (over all non-missing TTP records for participant-Day −1) [there are 2 records].

Week n value = minimum (over all non-missing TTP records for participant-Week n records) [there will typically be 4 records] for n >= 1.

Similar to baseline values for sputum culture results, for TTP:

Baseline is the Day 1 value (as derived above). If Day 1 is missing, or TTP ≥ 42 (negative MGIT sputum culture result), then it is the Day −1 values. If both Day 1 and Day −1 values are missing, or TTP >= 42 on both these days, then it is the Week 1 value (as derived above).

The statistical analysis for time to detection of MGIT cultures is described in Section 10.2.2.

8.2.1.3 Sputum Lipoarabinomannan Concentration

Sputum LAM concentrations are measured on up to 3 samples collected at baseline and then at every scheduled visit. Sputum conversion in LAM occurs when a participant has the first of 2 visits of at least 1 week apart with sputum LAM negative (below the lower limit of quantification) and without a positive sputum LAM result in between. The proportion of participants who convert sputum LAM to negative will be assessed at end-of-treatment and at 8 weeks of treatment.

Sputum LAM will be assessed using the same methods as the primary endpoint.

For categorical analyses < 10 pg/mL will be considered Negative. All values ≥ 10 pg/mL will be considered positive. The character result variable will contain "< 10" for values below 10, and the numerical result will be missing for these records.

There are two values for LAM per subject-visit. Only non-missing results will be used. If one is positive, overall result will be positive. Only when both results (or the only result if there is only one) are negative will the result at that visit be negative overall.

SCC for LAM will be considered to have been achieved if there are negative results at two consecutive visits with non-missing results.

LAM values may be analyzed as continuous numerical values.

The statistical analysis for Sputum LAM is described in Section 10.2.3.

8.2.1.4 Drug Resistance

Acquired resistance is defined as a post-baseline resistant result at any time point after a baseline susceptible result. Resistance data is collected for streptomycin, isoniazid, rifampicin, ethambutol, pyrazinamide, bedaquiline and delamanid. The baseline for drug resistance will be Day −1. If Day −1 is missing, then Day 1 will be used. If Day 1 is missing, then Week 1 will be used.

Susceptibility testing (DST) for anti-TB medications will be performed on positive *M tuberculosis* isolates from Day −1 cultures. If Day −1 culture is not positive for *M tuberculosis*, testing will be performed on Day 1 or Week 1 cultures. Susceptibility testing will also be performed on the end-of-treatment sputum specimen, if positive for *M tuberculosis* (Week 17 for Arms 1, 2, and 3, or Week 26 for Arm 4), as well as on positive cultures from participants suspected of relapse. Susceptibility testing may also be performed at other timepoints at the request of the principal investigator or the sponsor.

The presentation of Drug Resistance is described in Section 10.2.4.

MIC testing will be conducted for quabodepistat (OPC-167832). If MIC testing for DBQ and DLM is performed, it will reported out separately. All MIC testing results will be in a separate report.

8.2.1.5 Time to Sputum Culture Conversion (SCC)

Time to SCC will be calculated using MGIT cultures without the mitigation method measures, i.e., regular (MGIT). A sensitivity analysis will be performed with and without the mitigation method, i.e., MGIT (wwom). Each participant that has converted will have one time to SCC.

If a participant has a positive MGIT culture result throughout the study period, then he/she is considered as not having achieved SCC within the specific period under consideration. The time to SCC in this case is censored (censor variable = 1), and time to SCC will be taken to be from first dose date to end of follow-up (EOT date) or data cut-off date or Week 8 (only for CPC_8 analysis) as applicable in number of days, whichever is earlier. If the participant discontinued from the study, and did not achieve SCC, the time to SCC would be till the date of discontinuation if the date of discontinuation was prior to data cutoff date (DCO), but till DCO if otherwise. If the participant is lost to

follow-up (LTFU), then the time to SCC will be taken till the last MGIT culture assessment date if this is prior to the DCO date, and till the DCO date if the last MGIT culture assessment date is later. Participants who continue on the study beyond DCO, have missing SCC results prior to DCO, and have not achieved SCC within the analytic period, will be censored at their last non-missing SCC assessment date.

If a participant has a negative MGIT culture at 2 consecutive recorded visits more than 1 week apart, then the participant is considered to have had an SCC. The participant must not have a positive MGIT culture result till the end of the analysis period. This time to SCC value is uncensored (censor variable = 0). The time to SCC in this case is the time from first dose date of IMP to the first visit date of the 2 consecutive visit dates at which SCC was observed.

The statistical analysis for time to SCC is described in Section 10.2.5.

8.3 Exploratory Outcome Variables

CCI [redacted]

8.3.1.1 Favorable Outcome

A participant is considered to have a **favorable** outcome if, during the post randomization (post baseline) evaluation period, the participant is alive, completed the post randomization evaluation, is assessable, and otherwise, did not meet the definition of an unfavorable outcome. A participant will not be assessable if he/she does not have any efficacy or safety assessments beyond Week 1.

A participant is considered to have an **unfavorable** outcome if one of the following occurs:

- Clinical progression of pulmonary disease during treatment or the need for an unanticipated surgical intervention for TB (e.g., lobectomy).
- Any growth of *M tuberculosis* from an extrapulmonary site during the post-treatment period (Note: Excludes reinfection with a new strain of *M tuberculosis*).
- Signs and symptoms of active tuberculosis during the follow-up period (i.e., after the end-of-treatment), including radiographic worsening compared to baseline

findings, resulting in re-initiation of anti-mycobacterial therapy (note: excludes a re-initiation that is subsequently found to be due to a misdiagnosis and/or alternative diagnosis).

- Death during treatment or follow-up. This is all-cause death.
- A sputum culture with growth of *M tuberculosis* at certain time points as follows:
  - Failure to achieve SCC at the end of the treatment period that result in a change in anti-mycobacterial therapy.
  - Microbiologic relapse during the follow-up period (i.e., after the end of treatment) (Note: Excludes reinfection and requires more than 1 positive sputum culture with a new strain of *M tuberculosis*).

Favorable outcomes will be determined at the timepoints 12 months post randomization, and at 6 months post-end-of-treatment.

Favorable outcome at 12 months post-randomization is an Investigator-reported assessment and will be reported as collected in the data. The 6-months-post-end of treatment favorable outcomes are derived using the rules specified in Appendix 8.

8.3.1.2 Relapse

To determine if the participant has relapsed or has a new infection of *M tuberculosis*, genotyping will be performed on the baseline culture (Day −1 and/or Day 1, or Week 1, if necessary) and the first of any subsequent positive culture during the post treatment follow-up period. In addition, the sponsor may request genotyping as needed.

Relapse is defined as the same strain of *M tuberculosis* (by genotyping) identified prior to treatment that is causing the recurrence of an active TB disease after a successful treatment (achieved SCC) in a participant. If genotyping identifies an unrelated strain that caused the recurrence, it is considered as a reinfection but not a relapse.

Relapse will be determined at the timepoints 12 months post randomization, and at 6 months post-end-of-treatment.

8.3.2 Exploratory Biomarker Variables

Exploratory biomarker endpoints are:

- Longitudinally assess positron emission tomography/computerized axial tomography (PET/CT) imaging over the course of treatment.
- Longitudinally evaluate the ribosomal ribonucleic acid synthesis ratio (RS ratio) decline in sputum.
- Longitudinally assess whole blood transcriptomic signatures previously associated with TB cure from serum.

The analysis of these exploratory biomarker outcomes will be presented in a separate biomarker report.

9 Disposition and Demographic Analysis

9.1 Participant Disposition

Participant disposition will be summarized by treatment arm and stratification factors (when applicable) for below categories:

- Screened Participants
- Randomized
- Treated
- Randomized and Treated
- Discontinued Treatment
- Discontinued Study
- Completed Treatment
- Completed Study
- Safety analysis set
- mITT analysis set
- PP analysis set
- Treatment completer; Trial completer
- Treatment non-completer; Trial completer
- Treatment completer; Trial non-completer
- Treatment non-completer; Trial non-completer

9.2 Reasons for Discontinuation

Reason for discontinuation of treatment will be summarized by treatment arm and overall.

Reason for Discontinuation from Study will also be summarized by treatment arm and overall.

9.3 Demographic and Baseline Characteristics

Baseline is defined as the last non-missing value obtained at a scheduled visit on or before Day −1.

Demographic and baseline characteristics variables include:

- Age (years)
- Sex (Male/Female)
- Race
- Ethnicity
- TB Treatment History
- TB Treatment History – Site of Disease
- Height (cm)
- Weight (kg)
- BMI (kg/m^2)
- HIV Status
- Cavitation status
- Karnofsky Score (%)
- Sputum specimen results (without mitigation)
- Sputum specimen results (with mitigation)

9.4 Medical History

Medical history will be presented by the frequency and proportion (expressed in percent) for each system organ class and preferred term and for each treatment arm. Medical history preferred terms will be coded with MedDRA v25.0 or higher. The summary will be based on the randomized analysis set.

9.5 Treatment Exposure and Compliance

The time and dose of IMP administration during the trial will be recorded in eCRF and be listed. The relative dose intensity (RDI) will be used to assess the treatment compliance. The RDI is defined as:

RDI (%) = 100 × ADI (mg/day) / PDI (mg/day),

where ADI and PDI are the actual dose intensity and planned dose intensity, respectively:

PDI (mg/day) = planned cumulative dose (mg) / duration of exposure (days),

ADI (mg/day) = actual cumulative dose (mg) / duration of exposure (days).

The planned cumulative dose is the planned daily dose amount multiplied by the planned duration of exposure, while the actual cumulative dose is the sum of actual total daily dose amount over the duration of exposure.

The actual total daily dose is the dosage taken during a day by each participant of each of the different IP medications computed separately: delamanid (DLM), bedaquiline (BDQ) and quabodepistat (OPC-167832). The actual total daily dose will need information from Missed Doses, Overdose and Dose Administration.

The Actual Cumulative Dose (ACD) is computed for each participant as the sum of their actual total daily dose over the duration in the study accounting for overdoses and missed doses.

Summary of exposure will be generated for the safety analysis set and include ACD, ADI, PDI and RDI; and RDI categories (>80%, <=80%; >95%, <=95%).

The duration of exposure is defined as:

Duration of exposure (days) = (Date of last dose – Date of first dose) + 1, regardless of unplanned intermittent discontinuations. Summary statistics (N, mean, standard deviation, minimum, maximum) will be presented for duration of exposure by treatment arm and overall.

A summary table will be generated for the randomized analysis set presenting descriptive statistics on duration of exposure and the number and percentage of participants with exposure: No exposure, 0 to ≤ 4 Weeks, > 4 to ≤ 8 Weeks, > 8 to ≤ 12 Weeks, 12 to ≤ 16 Weeks, > 16 to ≤ 18 Weeks, > 18 to ≤ 20 Weeks, > 20 to ≤ 25 Weeks, > 25 to ≤ 27 Weeks, > 27 Weeks.

A listing will be generated for exposure information.

9.6 Prior and Concomitant Medication

The frequency and r proportion (expressed as percent) of participants taking concomitant medications will be tabulated by drug classification using the WHO drug dictionary WHODRUG B3G Mar 2023 (or newer version) for safety analysis set Medications taken prior to the study medication treatment period, concomitant medications taken during study medication treatment period and medications taken after the study treatment period, will be tabulated. The study medication treatment period is defined as day of first IMP treatment to the last day of IMP treatment. “After” study treatment refers to the period following the last day of IMP treatment to the end of the follow-up period. In instances where a participant may be followed up for a longer time beyond the 1-year post

randomization end of study, all medications taken in this period will also be included in the “after” study treatment period.

Prior medications that are not Anti-TB medications were collected for only 30 days prior to the first screening visit, while prior Anti-TB medications were those used at any time before starting study treatment.

The proportion of participants taking medications will be tabulated for all safety analysis sets prior to trial medication. The proportion of participants taking concomitant medications will be tabulated for the safety analysis set during and after the trial medication. Anti-TB concomitant medications and other general concomitant medications will be summarized separately.

In addition, listings of concomitant mediations will be provided.

9.7 Rescue Treatment/Medications

Rescue treatment consisting of the standard of care treatment, i.e., RHEZ is provided in the following circumstances:

1) If a participant is sputum culture positive for *M tuberculosis* at the end of treatment,
2) If a participant has a microbiologic relapse subsequent to the end of treatment, or
3) If, in the opinion of the investigator, a participant is clinically deteriorating, based on any combination of signs, symptoms, or chest radiograph data.

Note: Standardized rescue treatment consisting of RHEZ is to be used for Arm 1, Arm 2, and Arm 3 not responding to therapy. However, participants in Arm 4 will receive individualized care according to local guidelines.

The frequency and proportion (expressed as percent) of participants taking rescue medications will be tabulated in the safety analysis set.

A listing will display all rescue medications used in the study along with the reason for rescue medication.

9.8 Protocol Deviations

Major protocol deviations are entered into the CRF. These will be summarized by type of deviations (e.g., deviations in entry criteria, dosing, concomitant medication, procedural, etc.) by site. In addition, a listing of the major protocol deviations will be provided.

10 Efficacy Analyses

10.1 Primary Efficacy Analyses

The primary efficacy analysis will be based on the mITT population for (regular) MGIT. The primary efficacy endpoint of the trial is the proportion of participants who have achieved SCC by the end of treatment. SCC occurs when a participant has the first of 2 visits of at least 1 week apart with sputum cultures negative and without a positive sputum culture result in between, and without a positive culture by the end of treatment. If there are missing or contaminated culture results between two negative sputum culture assessments, the definition of conversion is still met.

Participants who discontinue treatment but continue to remain on the study will be included in this analysis.

The arms 1, 2 and 3 will be pooled. Noninferiority with RHEZ will be established if the lower limit of the 80% CI for the difference of proportion of participants with SCC in the pooled arms 1, 2 and 3, and the proportion of participants in the RHEZ arm, is larger than −0.12. In addition, each treatment arm will be compared separately to RHEZ.

The Miettinen and Nurminen method will be used to obtain the 80% CI of difference of proportions.

The SAS code for this analysis is provided in **Appendix 7 SAS Code**

The 80% and 95% CIs of the proportion difference between each arm and the standard-of-care arm, RHEZ, will be provided. In addition, Cochran-Armitage test will be used to detect trend in proportion of SCC with increasing quabodepistat (OPC-167832) dose.

Descriptive analyses will be conducted on the primary efficacy endpoint on the CPC analysis set and PP analysis sets.

For the CPC analysis, only completers and potential completers are included in the analysis. Participants ongoing treatment at data cutoff, will be excluded from the analysis. The proportion of participants achieving SCC within each treatment arm, pooled over OPC-167832 arms, and overall will be tabulated. No imputation is applied for potential completers, SCC is determined based on available assessments only. For completers, the end of treatment visit must be prior to or on the data cutoff date. For participants who achieve SCC within the analysis period (from first dose date to last dose date), SCC is set to “Yes”. For participants who do not achieve SCC within the analysis period, but for whom the last non-missing assessment within this period is negative, SCC will be determined by taking into account a next available visit assessment after the last non-missing on-treatment assessment, allowing for SCC to occur prior to or on data

cutoff. The denominator will be all CPC participants, and the numerator will be the number of participants achieving SCC (SCC = Yes), expressed as a percentage among CPC participants.

A sensitivity analysis will be conducted on the primary efficacy endpoint on the mITT analysis set using sputum culture assessments from both with and without mitigation (wwom) MGIT cultures similar to the regular MGIT analyses described above.

10.2 Secondary Efficacy Analyses

The secondary efficacy endpoints of this protocol are as stated in Section 8.2.1.

10.2.1 Sputum Culture Conversion in MGIT by 8 Weeks

The same analysis methodology specified for the primary endpoint will be presented for the secondary endpoint of SCC by 8 weeks.

10.2.2 Time to Positivity/Time to Detection of MGIT Cultures

Time to positivity/Time to detection of MGIT cultures and change from baseline will be summarized by treatment arm and visit, for each stratum separately and overall. This summary will be generated for the two populations, CPC analysis set and mITT analysis set. A separate sensitivity analysis will be conducted for TTP for with and without the mitigation method cultures.

10.2.3 Sputum Lipoarabinomannan

Numeric LAM data will be converted to a binary variable using the rule: if the LAM result is less than 10 pg/mL then it is interpreted as a negative result. If a LAM result is greater than or equal to 10 pg/mL then it is interpreted as positive result. Refer to Section 8.2.1.3. Sputum LAM conversion (SLC) will be assessed using the same definitions and rules as for SCC. The proportion of participants in each arm who convert sputum LAM to negative (i.e., achieve SLC) by 8 weeks of treatment and by the end of treatment will be analyzed and presented following the same methods as for SCC.

If data comes in character format “<10” with the corresponding numeric value missing, then the character format “<10” will be interpreted as a numeric value less than 10 pg/mL.

10.2.4 Drug Resistance

The number and proportion of participants who develop resistance to each of streptomycin, isoniazid, rifampicin, ethambutol, pyrazinamide, and bedaquiline will be

tabulated. Resistance data for streptomycin, isoniazid, rifampicin, ethambutol, pyrazinamide and bedaquiline is collected in the CRF and will be reported as collected for both the mITT analysis set and for the safety analysis set. by stratification variables and overall. Resistance results for each IMP will be presented by visit and overall. If a participant developed resistance to an IMP at scheduled or unscheduled timepoint, then they will be reported as having developed resistance overall within the study period.

10.2.5 Time to Sputum Conversion

For the final analysis, time to SCC will be presented on the mITT analysis set. For interim analyses where a data cutoff date is applied, the analysis will be presented on the CPC and CPC_8 analysis sets. The time to SCC analysis will be performed on both the regular and wwom MGIT data.

Censoring for the time to SCC endpoint has been described in Section 8.2.1.5.

Time to SCC will be analyzed using the Kaplan-Meier (KM) method. KM time to SCC curves for each treatment arm will be presented graphically and median time (displayed in days) to conversion will be estimated and presented together with the 25th and 75th percentiles. The Participants at risk will be summarized by treatment group at Week 2 (Day 14), Week 4 (Day 28), Week 6 (Day 42), Week 8 (Day 56), Week 10 (Day 70), Week 15 (Day 105), Week 17 (Day 119), Week 23 (Day 161) and Week 26 (Day 182).

Summaries of the number and percentage of participants experiencing an event, and number and percentage of participants censored, will be provided for each treatment group.

For both end of treatment and 8-weeks analyses, listings will be produced, including whether the participant had the event or, date of event/censored date, reasons for censoring, the start date, and the time to event (days).

CCI [redacted]

CCI

[redacted]

10.4 Exploratory Biomarker Analyses

The exploratory biomarkers specified in Section 8.3.2 will be described and analysed in a separate report.

10.5 Sensitivity Analyses

Sensitivity analyses have been described in various sections above. They are also summarized below:

- The PP analysis set will be used for sensitivity analyses of select efficacy data.

- For the primary endpoint, sensitivity analyses for determination of SCC may be performed for the IA. Sensitivity analysis of the primary endpoint will be performed using all 4 samples (wwom).
- Sensitivity analysis of TTP and Time to SCC will be performed using all 4 samples (wwom).

Select sensitivity analyses may be performed for the IA.

10.6 Subgroup Analyses

Subgroup analyses may be conducted.

11 Safety Analyses

Safety endpoints will be summarized using descriptive statistics (number, median, mean, standard deviation, minimum, maximum) for continuous values, and by frequency and proportion (expressed as percent) for discrete variables. Frequency and proportion (expressed as percent) of AEs and clinical laboratory test results by severity and toxicity grades will be presented.

11.1 Extent of Exposure

Extent of exposure to the trial medication for safety analysis set will be summarized using descriptive statistics (n, percentage) by treatment arm, pooled treatment arm and overall.

For each participant, duration of exposure to study drug will be defined as (date of last dose − date of first dose + 1). Participants' total days of exposure is defined as the sum of the number of exposure days for all participants within a group. Total days of exposure will be presented by treatment arm and overall.

11.2 Adverse Events

The frequency and percentage or proportion of participants with the following events will be summarized and tabulated by SOC, MedDRA preferred term, severity and treatment arm:

- AEs
- TEAEs
- Severe TEAEs
- TEAEs by DAIDS grade where grade >= 3
- TEAEs related to IMP
- TEAEs Leading to death

- Serious AEs
- Serious TEAEs
- TEAEs Leading to Discontinuations

The total number of AEs in each of these categories will be summarized.

All AEs will be presented in a listing. The listing of death due to AE, serious AE and TEAE leading to discontinuation will also be provided. In addition, the frequency and percentage or proportion of TEAE incidences will be summarized for serious TEAE, serious study medication related TEAE and non-serious TEAE.

11.3 Clinical Laboratory Data

Clinical laboratory test parameters and mean changes from baseline will be summarized by treatment arm for each scheduled visit. The incidence of potentially clinically significant (PCS) changes and abnormalities in clinical laboratory test parameters will be summarized using the DAIDS criteria (all grades greater than or equal to 1). The PCS events will include unscheduled assessments. Clinical laboratory test data will be presented in listings. For laboratory tests with different ranges for males and females, the results will be presented both separately by sex, and combined, in the Tables. Unscheduled laboratory assessments will be listed.

Laboratory data will be presented in SI units. Laboratory values that are received in character format with the corresponding numeric format missing, will be reviewed and assigned a numeric value for using in generating the summary tables.

11.4 Vital Sign Data

Vital sign parameters and mean changes from baseline will be summarized by treatment arm for each scheduled visit.

The incidence of potentially clinically significant changes and abnormalities in vital sign parameters will be summarized. Data from all visits will be included in the clinically significant result tables and included in all listings.

The DAIDS table criteria will be used for identifying vital signs of potential clinical significance and these values will be tabulated. These will be all values assigned a DAIDs grade greater than or equal to 1. The sponsor provided criteria from Appendix 3 Sponsor Provided Criteria for Identifying Vital Sign Measurements of Potential Clinical Relevance will also be tabulated separately.

Multiple numerical vital sign examination results for the same visit will be averaged to present a single value at a particular visit. Vital sign examinations marked by the investigator as repeat examinations will replace original results.

Unscheduled vital sign examinations will not enter summary tables and will only be reported in listings. However, unscheduled vital sign data determined to be potentially clinically relevant (PCR) will be summarized in the table for PCR vital signs.

11.5 Electrocardiogram Data

Electrocardiogram measurements and mean changes from baseline will be summarized by treatment over all scheduled visits. The following ECG parameters will be collected: ECG Mean Heart Rate (Beats/Min), PR Interval (msec), QRS Duration (msec), QT Interval (msec), QTCB Interval (msec), QTCF Interval (msec), RR Interval (msec). The average of triplicate ECGs will be presented rounded to the nearest integer. Refer to Section 8.1.2.4 for details.

Electrocardiogram data will be presented in listings. Both scheduled and unscheduled visit assessments will be listed. Potentially clinically significant ECG findings will be identified using the sponsor defined criteria in Appendix 4 *Sponsor Provided Criteria for Identifying ECG Measurements of Potential Clinical Relevance*

and will also be summarized.

11.6 Physical Examination Data

Physical examination findings for HEENT (head, eyes, ears, nose, and throat), Thorax, Abdomen, Skin and Mucosae, Neurological, Extremities, and Urogenital will be listed by participant for all visits including unscheduled. Clinical Significance assessments will be included in the listing on physical examination.

A listing of visual assessment based on Snellen and Ishihara testing will be presented.

11.7 Clinical Signs and Symptoms of Tuberculosis

An aggregate of clinical signs and symptoms of pulmonary TB will be assessed at screening and throughout the trial. Signs and symptoms will be assessed by the investigator (cough, hemoptysis, dyspnea, chest pain, loss of appetite, feeling feverish, and night sweats); from vital signs (body temperature, respiratory rate and heart rate), and physical examination (including weight and body mass index) from clinical laboratory tests (hemoglobin, hematocrit, c-reactive protein, and serum albumin); and from chest radiograph. A chest radiograph must be taken during the screening period, and an official interpretation must be available, and the assessment of its compatibility with pulmonary

TB must be made, prior to randomization. An end of treatment chest radiograph will be taken at Week 17 (for participants in Arms 1, 2, and 3) or Week 26 (for participants in Arm 4) and at Month 12.

A summary table will present the number and percent of participants with: persistent cough, fever, weight loss, findings on recent chest radiograph consistent with TB, hemoptysis, dyspnea, chest pain, night sweats and loss of appetite.

A listing will display individual participant data including those recording other signs and symptoms.

11.8 Other Safety Data

There are no other safety data to be analyzed.

12 Pharmacokinetic and Pharmacodynamic Analyses

Plasma concentration of quabodepistat (OPC-167832), DLM and its metabolite DM-6705, and BDQ and its metabolite M2 are planned to be utilized to perform population PK analyses, as applicable. A population PK analysis will be provided in a separate report.

Pharmacokinetics/PD and/or exposure-response analysis will be used to analyze the relationship of quabodepistat (OPC-167832) plasma exposure alone or combined with DLM and BDQ with the efficacy and safety endpoints, as applicable. The efficacy endpoints including but not limited to LAM, proportion of participants without SCC, time to detection and time to SCC may be explored. A PK/PD and/or exposure-response analysis will be provided in a separate report.

13 Pharmacogenomic Analyses

The assessment of transcriptomic signatures previously associated with TB cure from serum will be included as a part of Exploratory Biomarker Analyses as described earlier. No other pharmacogenomic analyses will be conducted.

14 Bioavailability Analyses

There will be no bioavailability analyses in this trial.

15 Interim Analyses

Interim PK/PD analyses will be performed after participants have completed the end of treatment response time point (4 months of treatment in the quabodepistat [OPC-167832] arm). In addition, an early assessment of dose-response and toxicity may be conducted

after the first 24 randomized participants (i.e., approximately 4:8:8:4 in each arm) complete the end of treatment response time point. If a quabodepistat (OPC-167832) arm is dropped, subsequently enrolled participants would then be allocated to the remaining OPC-167832 arms.

Other interim analyses may be performed for internal development purpose. The interim analysis will not result in stopping the study unless it is due to safety concern.

Analyses methods for interim analyses, unless otherwise indicated, will apply the rules as specified in this SAP. No statistical comparisons will be performed at the Interim, hence no adjustments for multiple testing need to be made in this study.

During the interim analysis, efficacy analyses will not test for non-inferiority. Only exact Clopper-Pearson 95% CIs will be generated for proportions in each treatment arm.

The interim analysis of efficacy data will primarily focus on the CPC population; however, tables on mITT may also be generated.

Select sensitivity analyses will be performed for the IA.

16 Data Monitoring Committee Analyses

An independent Data Monitoring Committee (DMC) will be contracted by Otsuka to review safety and efficacy data during the conduct of the trial. The DMC will convene and operate based on an established charter; the schedule of DMC review and contents/scope of DMC reports are discussed in the DMC charter.

The DMC will be permitted to seek additional independent expertise, as needed. The primary responsibility of the DMC will be to monitor safety and to advise the sponsor of the clinical project regarding whether or not the safety concerns merit stopping the trial. Towards this objective, the DMC will meet on a predetermined schedule to review safety and available efficacy data. The DMC and the study team will decide on the list of outputs that the DMC will review, and these will be presented at each DMC meeting.

17 Changes in Planned Analysis

Not applicable.

18 Document History

Dates/Author	Description of Updates	Version	Document Updates
15 May 2023/ PPD	Original version	1.0	not applicable
11 November 2023/ PPD PPD	Added text that Protocol Amendment 3 was not implemented, and SAP is based on Amendment 2.	1.1	Section 1 Introduction
	Added additional description on RHEZ including dosing		Section 3.2.1 Treatment Description
	Additional text added for those whose baseline MGIT is at Week 1, postbaseline will be Week 2 or later		Section 5.3 Modified-intent-to-treat analysis set
	Section on Per Protocol (PP) analysis set definition added.		Section 5.6 Per Protocol analysis set
	Added text Contaminated sputum cultures and Mycobacterium Other than Tuberculosis will be defined as missing culture assessments for the primary analyses		Section 6.1 Missing Data for Sputum Culture Conversion
	Text added on presenting analysis by pooled quabodepistat (OPC-167832) arms		Section 7 General Considerations for Statistical Analyses
	Added text Analyses of stratification will use IRT data, based on which the randomization was performed		Section 7.3 Stratification Factors/Stratum
	Included clarification that primary analysis of SCC, TID, K-M plots will be based only on 2 MGIT samples without mitigation. Details of scenarios of the two samples were included. Sensitivity Analysis will use all 4 samples without and with mitigation.<br>Updated scenarios for SCC result derivation in Primary analysis. For Sensitivity analysis added consideration for date of conversion.		Section 8.1.1 Primary Efficacy Variable
	Added test on two methods to determine severity of TEAEs		Section 8.1.2.1 Adverse Events
	Text added on Snellen and Ishihara visual assessment at select weeks		Section 8.1.2.5 Physical Examination Data
	Added text for free text reasons under other category		8.1.2.6 Rate of All-Cause Treatment Discontinuation
	Additional details added on methodology to calculate SCC at 8 weeks or EOT.		Section 8.2.1.1 SCC in MGIT at 8 Weeks
	Included clarification that analysis of Time to Positivity (TTP)/Time to Detection of MGIT will be based only on		Section 8.2.1.2 Time to Positivity (TTP)/Time to

Dates/Author	Description of Updates	Version	Document Updates
	2 MGIT samples without mitigation. Sensitivity Analysis will use all 4 samples without and with mitigation.		Detection of MGIT Cultures
	Additional details added on analysis of Sputum LAM concentration		Section 8.2.1.3 Sputum LAM Concentration
	Added text on MIC determination may also be carried out for BDQ and DLM		Section 8.2.1.4 Drug Resistance
	Removed text < 42 days for calculation of Censored subjects and added clarifications on censoring for participants who continue beyond data cutoff date		Section 8.2.1.5 Time to Sputum Culture Conversion (SCC)
	Updated section as per protocol section 9.4.3.6		Section 8.3.1.1 Favorable Outcome
	Added section on relapse		Section 8.3.1.2 Relapse
	Updated categories to display and in order suggested		Section 9.1 Participant Disposition
	Added section on reasons for discontinuations		Section 7.2 Reasons for discontinuation
	Updated categories to include Karnofsky Score (%), Sputum specimen results, etc.		Section 9.3 Demographic and Baseline Characteristics
	Additional details added on methodology to calculate treatment exposure and compliance		Section 9.5 Treatment Exposure and Compliance
	Section on analysis of Rescue Medications added		Section 9.7 Rescue Treatment/Medications
	Added section on rescue treatment/medications		Section 9.7 Rescue Treatment/Medications
	Added details for analysis to be presented in plots & tables, scale to consider for Participants at Risk, handling of missing Week 6, 7, 8 data in CPC_8 analysis set for subjects who did not achieve SCC.		Section 10.2.5 Time to Sputum Culture Conversion
	Added description of analysis of favorable outcomes		Section 10.3 Exploratory Efficacy Analysis
	Added Sections on Sensitivity and Subgroup Analyses		Section 10.5 Sensitivity Analyses<br>Section 10.6 Subgroup Analyses
	Added additional details on computation of total days of exposure		Section 11.1 Extent of Exposure
	Text added that laboratory data with different ranges for Males and Females will be presented separately		Section 11.3 Clinical Laboratory Data
	Section on Clinical Signs and Symptoms of Tuberculosis added		Section 11.7 Clinical Signs and Symptoms of Tuberculosis
	Clubbed Pharmacokinetic & Pharmacodynamic Analyses sections under Section 10		Section 12 Pharmacokinetic and Pharmacodynamic Analyses

Dates/Author	Description of Updates	Version	Document Updates
	Added text that Interim analysis will not test for inferiority.<br>Added text 80% and 95% CI For Primary pooled analysis and for rest of the analysis 95% CI will be calculated.		Section 10.1 on Primary Efficacy Analyses and Section 15 on Interim Analysis
	Appendix for detection of electrocardiogram was updated based on assessments being performed by ECG vendor		Appendix 4 Sponsor Provided Criteria for Identifying ECG Measurements of Potential Clinical Relevance
	Additional Abbreviations Added, Grammatical corrections and updates to text for clarity		Throughout protocol
4 June 2024/<br>PPD	Clarified text on Screen Failures		Section 5.1
	Minor clarifying text added throughout the document		Throughout document, including updates in Sections 12 and 13
	Added definition of relapse		Section 8.3.1.2
	Added clarification on Not Assessable category for favorable outcome		Section 10.3
	Added Appendix 8 for determination of favorable/unfavorable/not assessable outcomes at 6-months post-end-of-treatment.		Appendix 8
	Added that a separate report for MIC data would be prepared.		Section 8.2.1.4

19 References

1 United States Department of Health and Human Services, Division of AIDS, National Institute of Allergy and Infectious Diseases, National Institutes of Health. Division of AIDS (DAIDS) Table for Grading the Severity of Adult and Pediatric Adverse Events; 2017.

20 Appendices

Appendix 1 Definition of Grades in Serum Chemistry, Hematology and Urinalysis Lab Test Results in the DAIDS Table for Grading the Severity of Adult and Pediatric Adverse Events

The table below contains some of the criteria provided in the DAIDs table. Please see pages 25-31 of the DAIDs v2.1 tables[1] for the complete list for Chemistry, Hematology, and Urinalysis.

Definition of Grades in Serum Chemistry, Hematology and Urinalysis Lab Test Results in the DAIDS Table for Grading the Severity of Adult and Pediatric Adverse Events								
							Clinically Significant	
**LAB GROUP**	**TEST**	**UNITS**	**SEX**	**AGE GROUP**	**GRADE 1 MILD**	**GRADE 2 MODERATE**	**GRADE 3 SEVERE**	**GRADE 4 POTENTIALLY LIFE-THREATENING**
Chemistry	Albumin, Low	g/dL	Male/Female	All	3.0 to < LLN	≥ 2.0 to < 3.0	< 2.0	NA
Chemistry	Albumin, Low	g/L	Male/Female	All	30 to < LLN	≥ 20 to < 30	< 20	NA
Chemistry	Alkaline Phosphatase, High	-	Male/Female	All	1.25 to < 2.5 × ULN	2.5 to < 5.0 × ULN	5.0 to < 10.0 × ULN	≥ 10.0 × ULN
Chemistry	ALT or SGPT, High	-	Male/Female	All	1.25 to < 2.5 × ULN	2.5 to < 5.0 × ULN	5.0 to < 10.0 × ULN	≥ 10.0 × ULN
Chemistry	AST or SGOT, High	-	Male/Female	All	1.25 to < 2.5 × ULN	2.5 to < 5.0 × ULN	5.0 to < 10.0 × ULN	≥ 10.0 × ULN
Chemistry	Total Bilirubin, High	-	Male/Female	> 28 days of age	1.1 to < 1.6 × ULN	1.6 to < 2.6 × ULN	2.6 to < 5.0 × ULN	≥ 5.0 × ULN
Chemistry	Calcium, High	mg/dL	Male/Female	≥ 7 days of age	10.6 to < 11.5	11.5 to < 12.5	12.5 to < 13.5	≥ 13.5
Chemistry	Calcium, High	mmol/L	Male/Female	≥ 7 days of age	2.65 to < 2.88	2.88 to < 3.13	3.13 to < 3.38	≥ 3.38
Chemistry	Calcium, Low	mg/dL	Male/Female	≥ 7 days of age	7.8 to < 8.4	7.0 to < 7.8	6.1 to < 7.0	< 6.1
Chemistry	Calcium, Low	mmol/L	Male/Female	≥ 7 days of age	1.95 to < 2.10	1.75 to < 1.95	1.53 to < 1.75	< 1.53
Chemistry	Creatinine, High	-	Male/Female	All	1.1 to 1.3 × ULN	> 1.3 to 1.8 × ULN	> 1.8 to < 3.5 × ULN	≥ 3.5 × ULN
Chemistry	Creatinine Clearance or eGFR, Low	ml/min or ml/min/1.73m^2	Male/Female	All	N/A	< 90 to 60 ml/min or ml/min/1.73 m2 OR 10 to < 30% decrease from participant's baseline	< 60 to 30 ml/min or ml/min/1.73 m2 OR 30 to < 50% decrease from participant's baseline	< 30 ml/min or ml/min/1.73 m2 OR ≥ 50% decrease from participant's baseline or dialysis needed

**Definition of Grades in Serum Chemistry, Hematology and Urinalysis Lab Test Results in the DAIDS Table for Grading the Severity of Adult and Pediatric Adverse Events**								
							**Clinically Significant**	
**LAB GROUP**	**TEST**	**UNITS**	**SEX**	**AGE GROUP**	**GRADE 1 MILD**	**GRADE 2 MODERATE**	**GRADE 3 SEVERE**	**GRADE 4 POTENTIALLY LIFE-THREATENING**
Chemistry	Glucose, Fasting, High	mg/dL	Male/Female	All	110 to 125	> 125 to 250	> 250 to 500	≥ 500
Chemistry	Glucose, Fasting, High	mmol/L	Male/Female	All	6.11 to < 6.95	6.95 to < 13.89	13.89 to < 27.75	≥ 27.75
Chemistry	Glucose, Fasting, Low	mg/dL	Male/Female	≥ 1 month of age	55 to 64	40 to < 55	30 to < 40	< 30
Chemistry	Glucose, Fasting, Low	mmol/L	Male/Female	≥ 1 month of age	3.05 to < 3.55	2.22 to < 3.05	1.67 to < 2.22	< 1.67
Chemistry	Glucose, Non Fasting, High	mg/dL	Male/Female	All	116 to 160	> 160 to 250	> 250 to 500	≥ 500
Chemistry	Glucose, Non Fasting, High	mmol/L	Male/Female	All	6.44 to < 8.89	8.89 to < 13.89	13.89 to < 27.75	≥ 27.75
Chemistry	Cholesterol, High	mg/dL	Male/Female	All	200 to < 240	240 to < 300	≥ 300	NA
Chemistry	Cholesterol, High	mmol/L	Male/Female	All	5.18 to < 6.19	6.19 to < 7.77	≥ 7.77	NA
Chemistry	Inorganic Phosphorus, Low	mg/dL	Male/Female	> 14 years of age	2.0 to < LLN	1.4 to < 2.0	1.0 to < 1.4	< 1.0
Chemistry	Inorganic Phosphorus, Low	mmol/L	Male/Female	> 14 years of age	0.65 to < LLN	0.45 to < 0.65	0.32 to < 0.45	< 0.32
Chemistry	Inorganic Phosphorus, Low	mg/dL	Male/Female	< 1 to 14 years of age	3.0 to < 3.5	2.5 to < 3.0	1.5 to < 2.5	< 1.5
Chemistry	Inorganic Phosphorus, Low	mmol/L	Male/Female	< 1 to 14 years of age	0.97 to < 1.13	0.81 to < 0.97	0.48 to < 0.81	< 0.48
Chemistry	Magnesium, Low	mEq/L	Male/Female	All	1.2 to < 1.4	0.9 to < 1.2	0.6 to < 0.9	< 0.6
Chemistry	Magnesium, Low	mmol/L	Male/Female	All	0.60 to < 0.70	0.45 to < 0.60	0.30 to < 0.45	< 0.30
Chemistry	Potassium, High	mEq/L	Male/Female	All	5.6 to < 6.0	6.0 to < 6.5	6.5 to < 7.0	≥ 7.0
Chemistry	Potassium, High	mmol/L	Male/Female	All	5.6 to < 6.0	6.0 to < 6.5	6.5 to < 7.0	≥ 7.0
Chemistry	Potassium, Low	mEq/L	Male/Female	All	3.0 to < 3.4	2.5 to < 3.0	2.0 to < 2.5	< 2.0
Chemistry	Potassium, Low	mmol/L	Male/Female	All	3.0 to < 3.4	2.5 to < 3.0	2.0 to < 2.5	< 2.0
Chemistry	Sodium, High	mEq/L	Male/Female	All	146 to < 150	150 to < 154	154 to < 160	≥ 160
Chemistry	Sodium, High	mmol/L	Male/Female	All	146 to < 150	150 to < 154	154 to < 160	≥ 160
Chemistry	Sodium, Low	mEq/L	Male/Female	All	130 to < 135	125 to < 130	121 to < 125	≤ 120
Chemistry	Sodium, Low	mmol/L	Male/Female	All	130 to < 135	125 to < 130	121 to < 125	≤ 120
Chemistry	Triglycerides, High	mg/dL	Male/Female	All	150 to 300	> 300 to 500	> 500 to < 1,000	> 1,000
Chemistry	Triglycerides, High	mmol/L	Male/Female	All	1.71 to 3.42	> 3.42 to 5.7	> 5.7 to 11.4	> 11.4
Chemistry	Uric Acid, High	mg/dL	Male/Female	All	7.5 to < 10.0	10.0 to < 12.0	12.0 to < 15.0	≥ 15.0

**Definition of Grades in Serum Chemistry, Hematology and Urinalysis Lab Test Results in the DAIDS Table for Grading the Severity of Adult and Pediatric Adverse Events**								
							**Clinically Significant**	
**LAB GROUP**	**TEST**	**UNITS**	**SEX**	**AGE GROUP**	**GRADE 1 MILD**	**GRADE 2 MODERATE**	**GRADE 3 SEVERE**	**GRADE 4 POTENTIALLY LIFE-THREATENING**
Chemistry	Uric Acid, High	mmol/L	Male/Female	All	0.45 to < 0.59	0.59 to < 0.71	0.71 to < 0.89	≥ 0.89
Hematology	Activated Partial Thromboplastin Time, High	-	Male/Female	All	1.1 to < 1.66 × ULN	1.66 to < 2.33 × ULN	2.33 to < 3.00 × ULN	≥ 3.00 × ULN
Hematology	Prothrombin Time, High	-	Male/Female	All	1.1 to < 1.25 × ULN	1.25 to < 1.50 × ULN	1.50 to < 3.00 × ULN	≥ 3.00 × ULN
Hematology	Partial Thromboplastin Time, High	-	Male/Female	All	1.1 to < 1.66 × ULN	1.66 to < 2.33 × ULN	2.33 to < 3.00 × ULN	≥ 3.00 × ULN
Hematology	International Normalized Ratio (INR), High	-	Male/Female	All	1.1 to < 1.5 × ULN	1.5 to < 2.0 × ULN	2.0 to < 3.0 × ULN	≥ 3.00 × ULN
Hematology	Absolute CD4+ Count, Low	$cell/mm^3$	Male/Female	> 5 years of age (not HIV infected)	300 to < 400	200 to < 300	100 to < 200	< 100
Hematology	Absolute CD4+ Count, Low	cells/L	Male/Female	> 5 years of age (not HIV infected)	300 to < 400	200 to < 300	100 to < 200	< 100
Hematology	Absolute Lymphocytes, Low	$cell/mm^3$	Male/Female	> 5 years of age (not HIV infected)	600 to < 650	500 to < 600	350 to < 500	< 350
Hematology	Absolute Lymphocytes, Low	cells/L	Male/Female	> 5 years of age (not HIV infected)	0.600×10^9 to $< 0.650 \times 10^9$	0.500×10^9 to $< 0.600 \times 10^9$	0.350×10^9 to $< 0.500 \times 10^9$	$< 0.350 \times 10^9$
Hematology	Absolute Neutrophil Count, Low	$cell/mm^3$	Male/Female	> 7 days of age	800 to 1,000	600 to 799	400 to 599	< 400
Hematology	Absolute Neutrophil Count, Low	cells/L	Male/Female	> 7 days of age	0.800×10^9 to 1.000×10^9	0.600×10^9 to 0.799×10^9	0.400×10^9 to $< 0.599 \times 10^9$	$< 0.400 \times 10^9$
Hematology	Hemoglobin, Low	g/dL	Male	≥ 13 years of age	10.0 to 10.9	9.0 to < 10.0	7.0 to < 9.0	< 7.0
Hematology	Hemoglobin, Low	mmol/L	Male	≥ 13 years of age	6.19 to 6.76	5.57 to < 6.19	4.34 to < 5.57	< 4.34
Hematology	Hemoglobin, Low	g/dL	Female	≥ 13 years of age	9.5 to 10.4	8.5 to < 9.5	6.5 to < 8.5	< 6.5
Hematology	Hemoglobin, Low	mmol/L	Female	≥ 13 years of age	5.88 to 6.48	5.25 to < 5.88	4.03 to < 5.25	< 4.03
Hematology	Hemoglobin, Low	g/dL	Male/Female	57 days of age to < 13 years of age	9.5 to 10.4	8.5 to < 9.5	6.5 to < 8.5	< 6.5

**Definition of Grades in Serum Chemistry, Hematology and Urinalysis Lab Test Results in the DAIDS Table for Grading the Severity of Adult and Pediatric Adverse Events**								
							**Clinically Significant**	
**LAB GROUP**	**TEST**	**UNITS**	**SEX**	**AGE GROUP**	**GRADE 1 MILD**	**GRADE 2 MODERATE**	**GRADE 3 SEVERE**	**GRADE 4 POTENTIALLY LIFE-THREATENING**
Hematology	Hemoglobin, Low	mmol/L	Male/Female	57 days of age to < 13 years of age	5.88 to 6.48	5.25 to < 5.88	4.03 to < 5.25	< 4.03
Hematology	Platelet Count, Decreased	$cells/mm^3$	Male/Female	All	100,000 to < 125,000	50,000 to < 100,000	25,000 to < 50,000	< 25,000
Hematology	Platelet Count, Decreased	cells/L	Male/Female	All	$100,000 \times 10^9$ to $< 125.000 \times 10^9$	$50,000 \times 10^9$ to $< 100,000 \times 10^9$	$25,000 \times 10^9$ to $< 50,000 \times 10^9$	$< 25,000 \times 10^9$
Hematology	White Blood Cell, Decreased	$cells/mm^3$	Male/Female	> 7 days of age	2,000 to 2,499	1,500 to 1,999	1,000 to 1,499	< 1,000
Hematology	White Blood Cell, Decreased	cells/L	Male/Female	> 7 days of age	$2,000 \times 10^9$ to $2,499 \times 10^9$	$1,500 \times 10^9$ to $1,999 \times 10^9$	$1,000 \times 10^9$ to $1,499 \times 10^9$	$< 1,000 \times 10^9$
Urinalysis	Urine Glucose	-	Male/Female	All	Trace to 1+ or ≤ 250 mg	2+ or > 250 to ≤ 500 mg	> 2+ or > 500 mg	NA
Urinalysis	Urine Blood	-	Male/Female	All	6 to < 10 RBCs per high power field	≥ 10 RBCs per high power field	Gross OR Presence of RBC casts OR Intervention indicated	Life-threatening consequences
Urinalysis	Urine Protein	-	Male/Female	All	1+	2+	3+ or higher	NA

LLN = lower limit of normal; NA = not applicable; RBC = red blood cell; ULN=upper limit of normal

Appendix 2 Definition of Grades for Vital Signs in the DAIDS Table for Grading the Severity of Adult and Pediatric Adverse Events

The table below contains some of the criteria provided in the DAIDs table. Please see pages of the DAIDs v2.1 tables[1] for blood pressure abnormalities (page 7), unintentional weight loss (page 27), fever (page 21).

Definition of Grades for Vital Sign in the DAIDS Table for Grading the Severity of Adult Adverse Events

Abnormality	AGE GROUP	GRADE 1 MILD	GRADE 2 MODERATE	GRADE 3 SEVERE	GRADE 4 POTENTIALLY LIFE-THREATENING
Hypertension (with the lowest reading taken after repeat testing during a visit) Male/Female	≥ 18 years of age	140 to < 160 mmHg systolic OR 90 to < 100 mmHg diastolic	≥ 160 to < 180 mmHg systolic OR ≥ 100 to < 110 mmHg diastolic	≥ 180 mmHg systolic OR ≥ 110 mmHg diastolic	Life-threatening consequences in a participant not previously diagnosed with hypertension (e.g., malignant hypertension) OR Hospitalization indicated

Definition of Grades for Vital Sign in the DAIDS Table for Grading the Severity of Pediatric Adverse Events

Abnormality	GRADE 1 MILD	GRADE 2 MODERATE	GRADE 3 SEVERE	GRADE 4 POTENTIALLY LIFE- THREATENING
Unintentional Weight Loss (excludes postpartum weight loss)	NA	5% to < 9% loss in body weight from baseline	≥ 9% to < 20% loss in body weight from baseline	≥ 20% loss in body weight from baseline OR Aggressive intervention indicated (e.g., tube feeding, total parenteral nutrition)
Fever (non-axillary temperatures only)	38.0°C to < 38.6°C or 100.4°F to < 101.5°F	≥ 38.6°C to < 39.3°C or ≥ 101.5°F to < 102.7°F	≥ 39.3°C to < 40.0°C or ≥ 102.7°F to < 104.0°F	≥ 40.0°C or ≥ 104.0°F

Appendix 3 Sponsor Provided Criteria for Identifying Vital Sign Measurements of Potential Clinical Relevance

Sponsor Provided Criteria for Identifying Vital Sign Measurements of Potential Clinical Relevance		
**Variable**	**PCR Criterion Value**	**Change Relative to Baseline**
Systolic BP	> 160 mmHg	> 20 mmHg increase
	< 90 mmHg	> 20 mmHg decrease
Diastolic BP	≥ 100 mmHg	> 15 mmHg increase
	< 50 mmHg	> 15 mmHg decrease
Heart Rate	> 120 bpm	> 15 bpm increase
	< 50 bpm	> 15 bpm decrease
Respiratory Rate	≥ 25 breaths/min	-
	< 12 breaths/min	-
Temperature	< 35ºC	-
	> 38.0ºC	-
Weight	-	> 5% increase
	-	> 5% decrease

Appendix 4 Sponsor Provided Criteria for Identifying ECG Measurements of Potential Clinical Relevance

Sponsor Provided Criteria for Identifying EC Measurements of Potential Clinical Relevance		
**Category**<br>**Diagnosis**	**Criterion Value[a]**	**Change Relative to Baseline[a]**
**Interval**		
RR High	> 1200 msec	
RR Low	< 600 msec	
**Rhythm**		
Sinus tachycardia[b]	(HR_Mean > 100) AND (PR_Mean is Not Null)	
Sinus tachycardia[b]	(HR_Mean > 119) and (PR_Mean is Not Null)	
Sinus bradycardia[c]	(HR_Mean < 50 bpm) and (PR_Mean is Not Null)	
Sinus bradycardia[c]	(HR_Mean < 41) and (PR_Mean is Not Null)	
Artificial Pacemaker	All	not present → present
Atrial fibrillation	All	not present → present
Atrial flutter	All	not present → present
Atrial Pacing	All	not present → present
Atrial Tachycardia	All	not present → present
Ectopic Supraventricular Rhythm	All	not present → present
Idioventricular Rhythm	All	not present → present
Junctional Rhythm	All	not present → present
Junctional Tachycardia	All	not present → present
Sinus Pauses	All	not present → present
Sinus Bradycardia	All	not present → present
Sinus Tachycardia	All	not present → present
Supraventricular tachycardia	All	not present → present
Ventricular Fibrillation	All	not present → present
Other	All	not present → present
**Ectopy**	All	not present → present
Atrial Bigeminy	All	not present → present
Atrial Premature Complexes	All	not present → present
Frequent Atrial Premature Complexes (>3)	All	not present → present
Ventricular Premature Complexes	All	not present → present
Frequent Ventricular Premature Complexes (>2)	All	not present → present
Ventricular Bigeminy	All	not present → present
Ventricular Couplets	All	not present → present

Sponsor Provided Criteria for Identifying EC Measurements of Potential Clinical Relevance		
**Category**<br>**Diagnosis**	**Criterion Value[a]**	**Change Relative to Baseline[a]**
Ventricular Trigeminy	All	not present → present
Non-sustained Ventricular Tachycardia	All	not present → present
Premature Junctional Complexes	All	not present → present
Junctional Escape Complexes	All	not present → present
Other	All	not present → present
**Conduction**		
First Degree AV Block	PR > 0.20 s	
2:1 AV Block	All	not present → present
AV Mobitz I	All	not present → present
AV Mobitz II	All	not present → present
Complete Heart Block	All	not present → present
First Degree AV Block	All	not present → present
Incomplete Left Bundle Branch Block	All	not present → present
Incomplete Right Bundle Branch Block	All	not present → present
Intraventricular Conduction Defect	All	not present → present
Left Anterior Hemiblock	All	not present → present
Left bundle-branch block	All	not present → present
Left Posterior Hemiblock	All	not present → present
Prolonged QTc	All	not present → present
Right bundle-branch block	All	not present → present
Wolff-Parkinson-White	All	not present → present
Other	All	not present → present
**ST Segment**		
Depressed	All	not present → present
Elevated	All	not present → present
Other	All	not present → present
**T Waves**		
Biphasic	All	not present → present
Flat	All	not present → present
Inverted	All	not present → present
Notched	All	not present → present
Other	All	not present → present
**U Waves**		
Abnormal	All	not present → present
T-U Fusion	All	not present → present
Other	All	not present → present
**Myocardial Infarction**		
High Lateral MI 1, AVL	All	not present → present
Septal MI V1, V2, (V3)	All	not present → present
Antero Lateral MI V3-V6	All	not present → present
Inferior MI (2), 3, F	All	not present → present
Extensive Anterior MI 1, L, V1-V6	All	not present → present
Antero Septal MI V1-V4	All	not present → present
Lateral MI 1, L, V5, V6	All	not present → present
Anterior MI V3, V4	All	not present → present
Posterior MI	All	not present → present

Sponsor Provided Criteria for Identifying EC Measurements of Potential Clinical Relevance		
**Category**<br>**Diagnosis**	**Criterion Value[a]**	**Change Relative to Baseline[a]**
Other	All	not present → present
**Morphology**		
Left Atrial Abnormality	All	not present → present
Left Ventricular Hypertrophy	All	not present → present
Low Voltage	All	not present → present
Right Atrial Abnormality	All	not present → present
Right Ventricular Hypertrophy	All	not present → present
Brugada Syndrome	All	not present → present
Other	All	not present → present
**QT Interval**		
Increase in QT	QT > 450 msec (males and females)	
	QT > 480 msec (males and females)	
	QT > 500 msec (males and females)	
Increase in QTcF	QTcF > 450 msec (males and females)	
	QTcF > 480 msec (males and females)	
	QTcF > 500 msec (males and females)	
		Increase of ≥ 30 msec and ≤ 60 msec
		Increase of > 60 msec
Increase in QTcB	QTcB > 450 msec (males and females)	
	QTcB > 480 msec (males and females)	
	QTcB > 500 msec (males and females)	
		Increase of ≥ 30 msec and ≤ 60 msec
		Increase of > 60 msec

[a]In order to be identified as potentially clinically relevant, an on-treatment value must meet the “Criterion Value” OR represent a change from the participant’s baseline value of at least the magnitude shown in the “Change Relative to Baseline” column.

[b]No current diagnosis of supraventricular tachycardia, ventricular tachycardia, atrial fibrillation, atrial flutter, or other rhythm abnormality.

[c]No current diagnosis of atrial fibrillation, atrial flutter, or other rhythm abnormality.

Appendix 5 Completer Category Information

Completer Category Information	
Treatment completer; trial completer	• Randomized participant who has taken at least one dose of quabodepistat (OPC-167832), DLM, or BDQ, RHEZ<br>• Does not discontinue treatment in the treatment period (17 weeks for quabodepistat (OPC-167832), DLM, or BDQ /26 weeks for RHEZ)<br>• Has at least some assessments at the 17 week/26 week end-of-treatment visit (completes end-of-treatment visit)<br>• Continues on trial till the end of study visit at 12 months post randomization; completes end of study assessments
Treatment non-completer; trial completer	• Randomized participant who has taken at least one dose of quabodepistat (OPC-167832), DLM, or BDQ, RHEZ<br>• Discontinues treatment prior to the end of the treatment period (17 weeks for OPC-167832, DLM, or BDQ/26 weeks for RHEZ)<br>• Continues on trial till the end of study visit at 12 months post randomization; completes end of study assessments
Treatment completer; trial non-completer	• Randomized participant who has taken at least one dose of quabodepistat (OPC-167832), DLM, or BDQ, RHEZ<br>• Does not discontinue treatment in the treatment period (17 weeks for quabodepistat (OPC-167832), DLM, or BDQ/26 weeks for RHEZ)<br>• Has at least some assessments at the 17 week/26 week end-of-treatment visit (completes end-of-treatment visit)<br>• May or may not continue on trial after end-of treatment visit<br>• Does not continue till the end of study visit at 12 months post randomization; does not complete end of study assessments<br>• Includes participants who do not complete 12 months of post randomization follow-up for any reason: participants who die after end-of-treatment visit; participants lost to follow-up (LTFU) after end-of-treatment visit; participants who withdraw/are withdrawn from the study after end-of-treatment.

Completer Category Information	
Treatment non-completer; trial non-completer	• Randomized participant not treated with quabodepistat (OPC-167832), DLM, or BDQ, RHEZ<br>• OR at least one dose of quabodepistat (OPC-167832), DLM, or BDQ, RHEZ.<br>• If study treatment was started, discontinues treatment prior to the end of the treatment period (17 weeks for quabodepistat (OPC-167832), DLM, or BDQ /26 weeks for RHEZ).<br>• May or may not continue on trial after discontinuing treatment.<br>• If continues on study, discontinues prior to end-of study visit at 12 months post randomization; does not complete end of study assessments at the 12-month timepoint.<br>• Includes participants who: participants who die/are LTFU/withdraw/are withdrawn prior to 17 weeks/26 weeks treatment period, participants who die/are LTFU/withdraw/are withdrawn after discontinuing treatment (prior to 17 weeks/26 weeks) but prior to 12 months post randomization.

BDQ = bedaquiline; DLM = delamanid; LTFU = lost to follow-up; RHEZ = rifampin, isoniazid, ethambutol, pyrazinamide.

Appendix 6 Missing Data for Adverse Events/Concomitant Medications

In general, imputation of partial missing dates will be made for AE onset date, AE resolution date, death date, start and end dates of concomitant therapy, and date of initial diagnosis. Completely missing dates will not be imputed.

In general, if related information on the missing date exists in any other dataset that may help in determining the missing information, it will be used for imputations. Final decisions on key dates may be taken by key members of the study team and must include the study statistician.

Partial AE onset dates will be imputed as follows:

- If only day is missing, it will be set to:
  - First day of the month that the AE occurred, if month/year of the onset of AE is different than the month/year of first dose date.
  - The day of first dose date, if the month/year of the onset of AE is the same as month/year of the first dose date and month/year of the AE resolution date is different.
  - The day of first dose date or day of AE resolution date, whichever is earliest, if month/year of the onset of AE and month/year of the first dose date and month/year of the AE resolution date are same.
- If both day and month are missing, it will be set to the earliest of:
  - January 1 of the year of onset, as long as this date is after the first dose date;
  - Month and day of the first dose date, if this date is the same year that the AE occurred;
  - Last day of the year if the year of the AE onset is prior to the year of the first dose date;
  - AE resolution date.

Partial AE resolution dates not marked as ongoing will be imputed as follows:

- If missing day only, it will be set to the earlier of (last day of the month of resolution, the day of the date of death if death occurred in that month);
- If missing both day and month, it will be set to the earlier of (December 31 of the year, the day and month of the date of death if death occurred in that year).

For partial start and end dates of concomitant therapies, the AE imputation rules listed above will be applied accordingly.

For partial date of death and date of initial diagnosis, the following rule will be applied:

- If year is missing, no imputation will be made;
- If only year is present, but month and day are missing, June 30th will be used;
- If only day is missing but year and month are present, the 15th of the month will beused.

The above imputations will be modified by the following rules as needed:

- If imputed date for initial diagnosis is >= study consent date, consent date – 1 will be used;

If the imputed death date is < the last known alive date, later date + 1 will be used.

Appendix 7 SAS Code

CCI

[redacted]

Appendix 8 Determination of 6-month Post End-of-Treatment Favorable/Unfavorable/Not Assessable Outcome

Unfavorable Outcome Criteria	Date to Use	Rationale
1) Clinical progression of pulmonary disease <u>*during treatment*</u> or the need for an unanticipated surgical intervention for TB (e.g., lobectomy).<br><br>*Progression of Pulmonary Disease*	Stop date of IMP	If participant has clinical progression while on IMP, investigators would stop the IMP and start the participant with rescue meds.
2) Any growth of M tuberculosis from an extrapulmonary site during the post-treatment period (Note: excludes reinfection with a new strain of M tuberculosis).<br><br>*Growth of M Tuberculosis*	AE start date<br><br>Check for terms provided for extrapulmonary AEs	If participant developed TB from extrapulmonary site, this would be reported as AE and the start date would be the date of sample collection that confirmed positive for MTb.
3) Signs and symptoms of active tuberculosis during the follow-up period (i.e., after the end of treatment), including radiographic worsening compared to baseline findings, resulting in re-initiation of anti-mycobacterial therapy (Note: excludes a re-initiation that is subsequently found to be due to a misdiagnosis and/or alternative diagnosis).<br><br>*Signs and Symptoms of Tuberculosis*	Start Date of Rescue Meds	Rescue Medication criteria 3: "If, in the opinion of the investigator, a participant is clinically deteriorating, based on any combination of signs, symptoms, or chest radiograph data. Participants in Arm 4 will be managed according to local guidelines if not responding to therapy."<br><br>Start date of rescue medication: This confirms PI decision of unfavorable outcome due to clinical progression during post treatment period.
4) Death during treatment or follow-up<br><br>*Death During Treatment or Follow-up*	Date of Death	Date of death reflects time of event that is death.
5) A sputum culture with growth of M tuberculosis at certain time points as follows:<br><br>a) Failure to achieve SCC by the end of the treatment period that results in a change in anti-mycobacterial therapy;<br><br>*Failure to Achieve SCC*<br><br>b) Microbiologic relapse during the follow-up period (i.e., after the end of treatment) (Note: excludes reinfection and requires more than 1 positive sputum culture with a new strain of M tuberculosis).<br><br>*Microbiologic Relapse During Follow-up*	a) Date of sputum collection at end of treatment;<br><br>b) Date of first relapse (regular mgit) from the genotyping data	a) Date of sputum collection at Week 17 or Week 26 that confirms participant is still positive for MTb, hence decision to start rescue meds;<br><br>b) Date of first relapse (based on regular mgit only) as collected in the genotyping CRF page

AE = adverse event; CRF = case report form ; IMP = investigational medicinal product;TB = tuberculosis; mgit = Mycobacteria Growth Indicator Tube®;MTb = Mycobacterium tuberculosis; SCC = sputum culture conversion.

For each criterion, an unfavorable outcome is indicated by a date for the occurrence of that criterion prior to the 6-months post-end-of-treatment date. The overall unfavorable outcomes 6-months post-end-of-treatment occurs at the earliest of the dates identified in above table. If all dates are missing, then the participant has a favorable outcome 6-months post-end-of-treatment.

If a participant does not have sputum culture assessments at Weeks 34 and 39, he will be considered as “Not assessable” for the 6-months-post-end-of-treatment favorable outcome.

Otsuka

This page is a manifestation of an electronically captured signature

SIGNATURE PAGE

Document Name: P32320100006_SAP_4June2024

Document Number: [redacted]

Signed by	Meaning of Signature	Server Date (dd-MMM-yyyy hh:min) - UTC timezone
PPD	Clinical Approval	20-Jun-2024 12:31:08
	Biostatistics Approval	14-Jun-2024 12:54:19
	Clinical Pharmacology Approval	14-Jun-2024 03:04:52
