# Supplementary material for: Efficacy and safety of a 4-month quabodepistat, delamanid, and bedaquiline regimen for drug-susceptible pulmonary tuberculosis: a multicentre, open-label, randomised, proof-of-concept, non-inferiority, phase 2b/c trial
Source: Lancet Infect Dis. 2026 Oct;26(10):1010–21. doi: 10.1016/S1473-3099(26)00143-X (PMC13601027; doi:10.1016/S1473-3099(26)00143-X)
Supplement: Supplementary file 2 [file mmc2.pdf]

THE LANCET
Infectious Diseases

Supplementary appendix 2

This Equitable Partnership Declaration (EPD) was submitted by the authors, and we reproduce it as supplied. It has not been peer reviewed. *The Lancet*'s editorial processes have not been applied to the EPD.

Supplement to: Dawson R, Diacon AH, Variava E, et al. Efficacy and safety of a 4-month quabodepistat, delamanid, and bedaquiline regimen for drug-susceptible pulmonary tuberculosis: a multicentre, open-label, randomised, proof-of-concept, non-inferiority, phase 2b/c trial. *Lancet Infect Dis* 2026; published online May 29. https://doi.org/10.1016/S1473-3099(26)00143-X.

Equitable Partnership Declaration

If any questions do not apply to your study, please indicate “N/A” for “not applicable.”
For more information on how to complete this form, see the Information for Authors document.

Researcher considerations

1. Please detail the involvement that researchers who are based in the country or countries of study had during a) study design; b) clinical study processes, such as processing blood samples, prescribing medication, or patient recruitment; c) data interpretation; and d) manuscript preparation, commenting on all aspects. If they were not involved in any of these aspects, please explain why.

   This should include a thorough description of their leadership roles in the study. Are local researchers named in the author list or the acknowledgements, or are they not mentioned at all (and, if not, why)? Please also describe the involvement of early career researchers based in the location of the study. Some of this information might be repeated from the Contributors section in the manuscript. Note: we adhere to [ICMJE authorship criteria] for naming authors on a paper.

**a) Study design:**<br><br>This was a trial conducted in a single country, the Republic of South Africa, and at 6 clinical research sites. The lead in country principal investigators (PI) (1st author, Rodney Dawson from the University of Cape Town, South Africa and 2nd author, Andreas Diacon, Stellenbosch University, South Africa) were involved in the conceptualization of the project, study design, and final approval of the protocol. The investigators from all sites also had opportunity to provide comments on the protocol and study design during their respective IRB and regulatory submissions. Additionally, all principal investigators participated in the final review of the protocol and subsequent publication.<br><br>The protocol was previously published in *Trials (Dawson, R., Diacon, A.H., Takuva, S. et al. Quabodepistat in combination with delamanid and bedaquiline in participants with drug-susceptible pulmonary tuberculosis: protocol for a multicenter, phase 2b/c, open-label, randomized, dose-finding trial to evaluate safety and efficacy. Trials* ***25****, 70 (2024). https://doi.org/10.1186/s13063-024-07912-5)*.<br><br>During the conduct of the trial, the sponsor had periodic meetings with site investigators to solicit input with regard to improvements in study design and feasibility. Investigator input was further incorporated in preparing for the second amendment of the protocol.
**b) Clinical study processes:**<br><br>The PIs and their research teams at the different clinical sites and participating laboratories were responsible for all clinical study processes, such as processing blood samples, prescribing medication, and patient recruitment. The PIs oversaw the entire conduct of the study at their site, ensuring it complied with the protocol, ethical standards, Good Clinical Practice (GCP) guidelines (per ICH E6), and applicable regulations (South African Health Products Regulatory Authority).<br><br>The core responsibilities of the PIs were as follows:

• Oversee trial conduct per the approved protocol; document and report any deviations.<br>• Protect participants' rights, safety, and welfare, including proper informed consent, medical care for trial-related issues, and prompt reporting of serious adverse events.<br>• Ensure qualifications of self and staff; adequately supervise and train all delegated personnel (while retaining final responsibility).<br>• Secure and maintain IRB/IEC approval, permit monitoring, audits, and inspections.<br>• Manage investigational product (storage, dispensing, accountability).<br>• Ensure accurate, complete, and timely data collection, record-keeping, and reporting.<br>• Comply with all applicable regulations and sponsor requirements.<br><br>Professor Rodney Dawson (University of Cape Town), the first author, was the overall country PI assuming leadership of the study. The country PI served as the main point of contact for the sponsor regarding country-specific issues (eg, local ethics/IRB approvals, regulatory filings, community engagement, recruitment challenges, or cultural/contextual adaptations).<br><br>Sub-investigators at the different research sites conducted the day-to-day clinical research operations under the oversight of the PIs. Sub-investigators are early career researchers approved to conduct clinical studies as investigators.<br><br>The different clinical site PIs and sub-investigators are listed as co-authors, and the clinical research sites are acknowledged in the relevant section.
**c) Data interpretation:**<br><br>An interim and final analysis were conducted. The results were shared with the PIs and sub-investigators during investigator meetings and scheduled calls, and these were discussed. In preparation of the conference presentations and manuscripts, the different site PIs and sub-investigators were invited to review the abstract, presentation, and/or manuscript, and they had access to the tables and figures of results.<br><br>Every month during the conduct of the study, the sponsor clinical and operations teams had update meetings with country PIs on study conduct and safety data.
**d) Manuscript preparation:**<br><br>Editorial support was provided by The Medicine Group, LLC (New Hope, PA, United States). The listed co-authors constituting the PIs, sub-investigators, and sponsor teams were invited to participate in the review of the data and drafting and reviews of the different versions of the manuscript. All co-authors approved the final version.

2. How was funding used to remunerate and enhance the skills of researchers in the countries of study? And how was funding used to improve research infrastructure at the study sites?

   Potentially effective investments into long-term skills and opportunities within local institutions could include training or mentorship in analytical techniques and manuscript writing, opportunities to lead all or specific aspects of the study, financial remuneration rather than requiring volunteers, and other professional development and educational opportunities.

Improvements to research infrastructure could include funding extended trial designs (eg, platform trials), establishment of long-term contracts for research staff, building research facilities, and setting up local control of funding allocation.

**Skills:**<br><br>Funding for the study was primarily used to support the clinical trial activities conducted under the site's standard clinical trial agreement. While no additional formal training was required for the clinical staff beyond routine study procedures, the project did contribute to skills development in specific areas. In particular, the microbiology laboratory personnel gained hands-on expertise in using the LAM Pathfast Machine, which enhanced their technical capacity in diagnostic testing and contributed to improved laboratory competency beyond the scope of this single study.<br><br>Although the study did not introduce new financial remuneration structures or extensive professional development programs for site researchers, the engagement in the trial provided opportunities for staff to participate in study implementation, data collection, and laboratory operations—strengthening their practical experience in clinical research. These activities support long-term capability by exposing local teams to study protocols, quality standards, and operational workflows typical of sponsored clinical trials.
**Research infrastructure:**<br><br>With respect to infrastructure, the funding helped ensure the availability and use of essential study equipment, most notably the LAM Pathfast Machine within the microbiology laboratory. This investment supported diagnostic capacity during the trial. While the study did not involve construction of new research facilities, expansion of trial platforms, or establishment of long-term research contracts, the equipment and operational experience gained contribute to the laboratory's broader ability to participate in future research.

3. How did you safeguard the researchers who implemented the study?

Please describe how you guaranteed safe working conditions for study staff, including provision of appropriate personal protective equipment, protection from violence, and prevention of overworking.

Research staff were safeguarded through a standard clinical-trial operational structure, supported by comprehensive Operations Manuals outlining all study procedures, including the proper handling and disposal of biohazard materials. The sponsor ensured that each site met infection-control requirements appropriate for a tuberculosis study, and staff were provided with the necessary personal protective equipment consistent with institutional and study-specific biosafety protocols. Although the study did not involve elevated security risks, site processes ensured that personnel had a secure working environment and clear reporting mechanisms for any safety concerns. Together, these measures ensured safe, compliant, and ethically responsible working conditions for all study personnel.

Benefits to the communities and regions of study

4. How does the study address the research and policy priorities of its location?

   How were the local priorities determined and then used to inform the research question? Who decided which priorities to take forward? Which elements of the study address those priorities?

> The study directly addresses South Africa's research and policy priorities for tuberculosis by focusing on improved diagnostic and treatment strategies within a setting that carries one of the highest tuberculosis and drug-resistant tuberculosis burdens globally. South African tuberculosis patients represent a population that stands to benefit most from advances such as regimen shortening and novel drug combinations, making the country an essential setting for generating clinically meaningful evidence. Local priorities were identified through national tuberculosis strategic objectives and the expertise of South African investigators, who jointly determined that evaluating improved diagnostic tools and approaches would best support national needs. The country's diverse population also provides valuable pharmacokinetic and pharmacodynamic insights applicable to wider global populations. Decisions on which priorities to pursue were made collaboratively by the sponsor and experienced South African tuberculosis clinicians who served as study investigators. The study's use of South Africa's strong clinical trial infrastructure including established microbiology, diagnostic, pharmacology, and radiology capacities ensured that the research was well aligned with national capabilities. Ultimately, the evidence generated will benefit not only South African patients but also tuberculosis-affected communities across other low- and middle-income countries with similarly high disease burdens.

5. How will research products be shared in the community of study?

   For instance, will you be providing written or oral layperson summaries for non-academic information sharing? Will study data be made available to institutions in the region(s) of study? The Lancet Global Health *encourages authors to translate the summary (abstract) into relevant languages after paper editing; do you intend to translate your summary?*

> Research outputs from the study will be shared through established public-access mechanisms, including the posting of results on ClinicalTrials.gov, where they are accessible to the broader scientific and community audience. Study data will be shared with collaborating institutions in accordance with sponsor policies and applicable data-sharing agreements.
>
> As the study population and research partners operate primarily in English, translation of the publication summary is not required.

6. How were individuals, communities, and environments protected from harm?

a) *How did you ensure that sensitive patient data were handled safely and respectfully? Was there any potential for stigma or discrimination against participants arising from any of the procedures or outcomes of the study?*

Individuals, communities, and the environment were protected through adherence to established biosafety and ethical standards throughout the study. All biohazard materials were managed and disposed of by qualified third-party vendors who operated under approved standard operating procedures for hazardous-waste handling, thereby minimizing environmental and community risk. Sensitive participant data were coded and stored according to institutional data-protection requirements to ensure confidentiality and respectful handling of personal information. To limit stigma and minimize any burden on participants, the study reduced the need for in-person Directly Observed Therapy (DOT) by using MERMS to monitor adherence and engaged relatives to assist with DOT only when necessary and with the participant's consent. These approaches reduced visibility of tuberculosis treatment-related procedures, thereby lowering the potential for inadvertent disclosure, stigma, or discrimination. Overall, the combination of robust biosafety practices, secure data-management processes, and participant-centered adherence strategies provided comprehensive protections throughout the study.

b) *Might any of the tests be experienced as invasive or culturally insensitive?*

To protect individuals from harm and ensure cultural sensitivity, the study team revised the baseline safety procedures after sites expressed concerns about the acceptability of urogenital examinations. Although the original protocol included a urogenital exam as part of the holistic baseline assessment, this requirement was replaced with an exhaustive, investigator-led medical history focused on the urogenital system. Participants were referred to an appropriate specialist only when clinically warranted based on this assessment. This approach preserved participant safety while avoiding unnecessary examinations that could be perceived as invasive or culturally insensitive. By prioritizing participant comfort, respecting cultural norms, and limiting sensitive examinations to situations of genuine medical need, the study minimized the potential for distress, discomfort, or unintended harm within the participating communities.

c) *How did you determine that work was sensitive to traditions, restrictions, and considerations of all cultural and religious groups in the study population?*

Cultural and religious considerations were evaluated during site initiation and through direct consultation with site investigators, who provided guidance on community norms and sensitivities relevant to study procedures. This process informed modifications to protocol requirements such as replacing routine urogenital examinations with a detailed medical history and specialist referral only when clinically indicated to avoid procedures that could be considered invasive or culturally inappropriate. All participant interactions were conducted by trained staff who followed GCP and institutional ethics-committee guidance to ensure respect for personal dignity, privacy, and autonomy. In addition, biohazard materials were managed by certified disposal vendors in accordance with local regulations to protect surrounding communities and the environment. Through these combined measures, the

study ensured that all procedures were culturally sensitive, ethically justified, and aligned with the expectations and values of the population involved.

d) *Were biowaste and radioactive waste disposed of in accordance with local laws?*

All biological waste generated during the study was managed by qualified third-party vendors who operated under approved standard operating procedures and in full compliance with local South African laws and environmental regulations. The sponsor verified these safeguards by confirming the vendors' certifications and reviewing their waste-management SOPs, and additional oversight was provided through audits conducted at the microbiology laboratory to ensure proper containment, handling, and disposal practices. These combined measures ensured that biowaste was safely managed throughout the study and that no risks were posed to study participants, site personnel, surrounding communities, or the environment.

e) *Were any structures built that would have impacted members of the community or the environment (such as handwashing facilities in a public space)? If so, how did you ensure that you had appropriate community buy-in?*

The study did not involve construction of any new structures or facilities that could impact local communities or the environment. Instead, efforts focused on ensuring that existing site infrastructure met appropriate infection-control standards to prevent cross-contamination and minimize the risk of tuberculosis transmission. Because no structural modifications or community-facing installations were implemented, community buy-in processes related to construction were not required. All infection-control measures were implemented within the established clinical environment and in accordance with local regulatory and institutional requirements, ensuring that study activities were contained within the site and posed no environmental or community impact.

f) *How might the study have impacted existing health-care resources (such as staff workloads, use of equipment that is typically employed elsewhere, or reallocation of public funds)?*

The study was structured to minimize any impact on existing health-care resources by ensuring that all trial activities were fully supported through the sponsor and managed within the site's established research infrastructure. Investigator and staff compensation followed local guidelines and was formalized through the clinical trial agreement, which ensured that staffing allocations dedicated to the study did not draw from routine clinical care. During participants' transition periods to local standard of care, they were encouraged to remain under study follow-up for safety assessments rather than shifting immediately to community clinics. Investigators maintained communication with local clinics to update them on participant status, thereby reducing additional workload for public-sector facilities and preserving local health-care resources. Through these measures, the study avoided placing undue demands on clinical staff, services, or equipment.

7. Confirm that local ethics review was sought, and please provide the approval number. If not sought, please explain why.

The list of local ethics committees that conducted the review and granted approval for the study has been provided separately (manuscript appendix) and includes the associated ethics approval numbers.

Secondary analyses

8. Have the data analysed in your study been extracted from another source, such as a national survey, rather than being directly collected by the authors of this paper?

N/A

If the authors of this paper were not involved in data collection, how were the findings interpreted with sufficient contextual knowledge?
The Lancet Global Health *believe contextual understanding is crucial for informed data analysis and interpretation.*

N/A

---

9. Finally, please provide the title (eg, Dr/Prof, Mr/Mrs/Ms/Mx), name, and email address of an author who can be contacted about this statement.

Name: Dr. Simbarashe Takuva
Email: STakuva@otsuka-onpg.com
